# National, regional, and state-level burden of *Streptococcus pneumoniae* and *Haemophilus influenzae* type b disease in children in India: modelled estimates for 2000–15

**DOI:** 10.1016/S2214-109X(19)30081-6

**Published:** 2019-05-13

**Authors:** Brian Wahl, Apoorva Sharan, Maria Deloria Knoll, Rajesh Kumar, Li Liu, Yue Chu, David A McAllister, Harish Nair, Harry Campbell, Igor Rudan, Usha Ram, Molly Sauer, Anita Shet, Robert Black, Mathuram Santosham, Katherine L O'Brien, Narendra K Arora

**Affiliations:** a International Vaccine Access Center, Johns Hopkins Bloomberg School of Public Health, Baltimore, MD, USA; b Institute for International Programs, Johns Hopkins Bloomberg School of Public Health, Baltimore, MD, USA; c Department of International Health, and Department of Population, Family and Reproductive Health, Johns Hopkins Bloomberg School of Public Health, Baltimore, MD, USA; d INCLEN Trust International, New Delhi, India; e School of Public Health, Post Graduate Institute of Medical Education and Research, Chandigarh, India; f Institute of Health and Wellbeing, University of Glasgow, Glasgow, UK; g Centre for Global Health Research, Usher Institute of Population Health Sciences and Informatics, Medical School, University of Edinburgh, Edinburgh, UK; h Public Health Foundation of India, New Delhi, India; i Department of Public Health and Mortality Studies, International Institute for Population Sciences, Mumbai, India

## Abstract

**Background:**

India accounts for a disproportionate burden of global childhood illnesses. To inform policies and measure progress towards achieving child health targets, we estimated the annual national and state-specific childhood mortality and morbidity attributable to *Streptococcus pneumoniae* and *Haemophilus influenzae* type b (Hib) between 2000 and 2015.

**Methods:**

In this modelling study, we used vaccine clinical trial data to estimate the proportion of pneumonia deaths attributable to pneumococcus and Hib. The proportion of meningitis deaths attributable to each pathogen was derived from pathogen-specific meningitis case fatality and bacterial meningitis case data from surveillance studies. We applied these proportions to modelled state-specific pneumonia and meningitis deaths from 2000 to 2015 prepared by the WHO Maternal and Child Epidemiology Estimation collaboration (WHO/MCEE) on the basis of verbal autopsy studies from India. The burden of clinical and severe pneumonia cases attributable to pneumococcus and Hib was ascertained with vaccine clinical trial data and state-specific all-cause pneumonia case estimates prepared by WHO/MCEE by use of risk factor prevalence data from India. Pathogen-specific meningitis cases were derived from state-level modelled pathogen-specific meningitis deaths and state-level meningitis case fatality estimates. Pneumococcal and Hib morbidity due to non-pneumonia, non-meningitis (NPNM) invasive syndromes were derived by applying the ratio of pathogen-specific NPNM cases to pathogen-specific meningitis cases to the state-level pathogen-specific meningitis cases. Mortality due to pathogen-specific NPNM was calculated with the ratio of pneumococcal and Hib meningitis case fatality to pneumococcal and Hib meningitis NPNM case fatality. Census data from India provided the population at risk.

**Findings:**

Between 2000 and 2015, estimates of pneumococcal deaths in Indian children aged 1–59 months fell from 166 000 (uncertainty range [UR] 110 000–198 000) to 68 700 (44 600–86 000), while Hib deaths fell from 82 600 (52 300–112 000) to 15 600 (9800–21 500), representing a 58% (UR 22–78) decline in pneumococcal deaths and an 81% (59–91) decline in Hib deaths. In 2015, national mortality rates in children aged 1–59 months were 56 (UR 37–71) per 100 000 for pneumococcal infection and 13 (UR 8–18) per 100 000 for Hib. Uttar Pradesh (18 900 [UR 12 300–23 600]) and Bihar (8600 [5600–10 700]) had the highest numbers of pneumococcal deaths in 2015. Uttar Pradesh (9300 [UR 5900–12 700]) and Odisha (1100 [700–1500]) had the highest numbers of Hib deaths in 2015. Less conservative assumptions related to the proportion of pneumonia deaths attributable to pneumococcus indicate that as many as 118 000 (UR 69 000–140 000) total pneumococcal deaths could have occurred in 2015 in India.

**Interpretation:**

Pneumococcal and Hib mortality have declined in children aged 1–59 months in India since 2000, even before nationwide implementation of conjugate vaccines. Introduction of the Hib vaccine in several states corresponded with a more rapid reduction in morbidity and mortality associated with Hib infection. Rapid scale-up and widespread use of the pneumococcal conjugate vaccine and sustained use of the Hib vaccine could help accelerate achievement of child survival targets in India.

**Funding:**

Bill & Melinda Gates Foundation.

## Introduction

*Streptococcus pneumoniae* (pneumococcus) and *Haemophilus influenzae* type b (Hib) are serious causes of pneumonia, meningitis, and other severe childhood infections in India and worldwide.^1^ Highly efficacious vaccines that provide protection from these pathogens have been used in developed and developing countries for many years; global data on vaccine introduction and vaccine programme implementation can be visualised on VIEW-hub, an interactive platform developed by the International Vaccine Access Center (IVAC). The Hib-containing pentavalent vaccine was introduced in the immunisation programmes of Tamil Nadu and Kerala in December, 2011.^2^ It is now used in the routine immunisation programme of all states in India. The 13-valent pneumococcal conjugate vaccine (PCV) was introduced subnationally in 2017. There are plans to expand its use in India in the coming years.

**Research in context**

**Evidence before this study**

We searched PubMed for national and subnational estimates of pneumococcal and *Haemophilus influenzae* type b (Hib) morbidity and mortality in children in India from 2000 to 2017. Estimates of national pneumococcal and Hib morbidity and mortality for 2000 were published in 2009 by the Child Health Epidemiology Reference Group (CHERG) collaboration (now the WHO Maternal and Child Epidemiology Estimation collaboration). Subnational estimates of pneumococcal pneumonia morbidity and mortality for India in 2010 were published in 2015. Based on these estimates, there were approximately 564 000 cases of pneumococcal pneumonia and 105 000 deaths from pneumococcal pneumonia in 2010. No subnational disease burden estimates for Hib were identified in the literature.

**Added value of this study**

We present, to our knowledge, the first comprehensive subnational estimates of pneumococcal and Hib morbidity and mortality in children aged 1–59 months in India, due to all syndromes associated with these pathogens (ie, pneumonia, meningitis, and other invasive diseases). This study is also the first to report on the annual subnational pneumococcal and Hib disease burden from 2000 to 2015. These estimates are based on recently published subnational cause of death estimates for India and incorporate new data from 67 studies published between 2006 and 2014, including three studies from India. These studies report on pathogen-specific meningitis case fatality and the distribution of meningitis case estimates by cause. Our pneumococcal and Hib meningitis morbidity and mortality models used updated methods that are based on estimates of all-cause meningitis deaths. The methods used in this study differ from previously published subnational pneumococcal disease burden estimates.

**Implications of all the available evidence**

Our estimates provide insight into progress towards reducing the morbidity and mortality associated with these two pathogens in young children. These data can be used to inform policies around expanded use of the pneumococcal conjugate vaccine in India.

Efforts to improve access to primary maternal and child health services in India have contributed to progress towards reducing child mortality. Many of these efforts have been concentrated in less socioeconomically developed states, including those that comprise the Empowered Action Group (EAG). Although India narrowly missed the Millennium Development Goal child mortality target of 44 deaths per 1000 livebirths by 2015,^3^ implementation and widespread use of new child health interventions, including new vaccines that protect children from some of the leading causes of mortality, could accelerate achievement of the Sustainable Development Goal of 25 child deaths per 1000 livebirths or lower in India by 2030. Since around 20% of child deaths occur in India, global progress towards reducing child mortality will depend on progress in India.

Disease burden is an important decision-making factor for the introduction and sustained use of new vaccines. The technical challenges associated with doing observational surveillance studies for pneumococcal and Hib infections in resource-poor settings have been extensively documented.^4^, ^5^ Models can provide complementary insight into disease burden when observational studies are absent, inconsistent, or unable to provide such information for epidemiological reasons. For large and diverse countries such as India, subnational disease burden estimates are particularly important for policy making and programme planning, as national burden estimates can mask substantial variation in morbidity and mortality across large subnational populations.

Researchers have estimated that there were around 105 000 deaths from pneumococcal pneumonia and 564 000 cases of severe pneumococcal pneumonia in 2010 in children younger than 5 years in India.^6^ There are no published subnational burden estimates for clinical pneumococcal pneumonia, pneumococcal meningitis, or other invasive pneumococcal diseases. There are also no published subnational burden estimates for Hib infection. In light of widespread Hib vaccine use, the anticipated expansion of PCV use in India, and reductions in all-cause mortality since 2010, we aimed to prepare state-level pneumococcal and Hib disease burden estimates for India from 2000 to 2015.

## Methods

### Overview

In this modelling study, we estimated the pathogen-specific burden in children aged 1–59 months at the state level separately for each of the three clinical syndromes associated with pneumococcus and Hib (ie, pneumonia, meningitis, and invasive non-pneumonia, non-meningitis [NPNM]) using methods briefly described here and in detail elsewhere.^1^ NPNM is characterised by the isolation of pneumococcus or Hib from normally sterile body fluid but without the clinical findings of pneumonia or meningitis. Input data for each model are described in table 1.^7^, ^8^, ^9^, ^10^, ^11^, ^12^, ^13^, ^14^, ^15^

**Table 1:** State-specific model parameters and sources

	**Sources of data from India**	**Sources of data from outside India or approach for missing data, or both**
**Pathogen-specific pneumonia**		
All-cause pneumonia deaths	Modelled estimates based on verbal autopsy studies from India^7^	NA
Pneumonia cases	Modelled estimates based on risk factor prevalence data from India^8^	NA
Proportion of pneumonia cases and deaths attributable to each pathogen	NA	Used global estimate with clinical trial data^1^
**Pathogen-specific meningitis**		
All-cause meningitis deaths*	Modelled estimates based on verbal autopsy studies from India^7^	NA
Proportion of meningitis cases attributable to each pathogen	Meningitis surveillance: 2 studies^9^, ^10^	Meningitis surveillance from Asia: 33 studies†
Pneumococcal meningitis CFR	Pneumococcal meningitis surveillance from low mortality settings: NA; medium mortality settings: NA; high mortality settings: NA, very high mortality settings: NA	Pneumococcal meningitis surveillance from low mortality settings: 36 studies; medium mortality setting: 13 studies; high mortality settings: 8 studies; very high mortality settings: 7 studies
Hib meningitis CFR	Hib meningitis surveillance from low mortality settings: NA; medium mortality settings: 2 studies;^11^, ^12^ high mortality settings: 1 study;^13^ very high mortality settings: NA	Hib meningitis surveillance from low mortality settings: 31 studies; medium mortality settings: 16 studies; high mortality settings: 4 studies; very high mortality settings: 10 studies
**Pathogen-specific NPNM**		
Pneumococcal NPNM case multiplier	Severe pneumococcal disease surveillance from low or medium mortality settings: NA; high or very high mortality settings: 2 studies;^14^, ^15^ non-severe pneumococcal disease surveillance: NA	Severe pneumococcal disease surveillance from low or medium mortality settings: 26 studies; high or very high mortality settings: 4 studies; non-severe pneumococcal disease surveillance: 2 studies
Hib NPNM case multiplier	Hib disease surveillance from low or medium mortality settings: NA; high or very high mortality settings: NA	Hib disease surveillance from low or medium mortality settings: 26 studies; high or very high mortality settings: 3 studies
Pneumococcal NPNM CFR multiplier	Pneumococcal disease surveillance: NA	Pneumococcal disease surveillance: 2 studies
Hib NPNM CFR multiplier	Hib disease surveillance: NA	Hib disease surveillance: 5 studies
**Population at risk and demographic model parameters**		
Child mortality	Unpublished modelled data based on SRS data from India	NA
Child population	2001 and 2011 India census data	Interpolated and extrapolated with annual census growth rates estimates
Access to care for meningitis	NFHS 2, 3, and 4‡	Linear interpolation and extrapolation
HIV prevalence	UNAIDS unpublished data	Apportioned HIV burden to states based on population
Hib vaccine coverage	Inferred from DTP3 coverage estimates from AHS, DLHS, and NFHS	Linear interpolation or extrapolation

NA=not available. CFR=case fatality ratio. NPNM=non-pneumonia, non-meningitis. Hib=*Haemophilus influenzae* type b. SRS=Sample Registration System. NFHS=National Family and Household Survey. DTP3=third dose of diphtheria-tetanus-pertussis vaccine. AHS=Annual Health Survey. DLHS=District Level Household Survey.

* All-cause meningitis cases and all-cause NPNM cases and deaths are not used; pathogen-specific meningitis cases and pathogen-specific NPNM cases and deaths are based on pathogen-specific meningitis deaths.

† Full list of citations for non-Indian studies provided in the appendix (pp 5–9).

‡ Used data from question related to care seeking for pneumonia symptoms as a proxy for care seeking for meningitis.

As previously reported,^1^ we updated a systematic review of pneumococcal and Hib invasive disease^16^ with published and unpublished data up to and including 2014 for the pathogen-specific meningitis and NPNM models. We searched six global databases (ie, PubMed, Embase, Biosis, Cochrane, Global Health, and Pascal) and five regional databases (ie, IMEMR, IMSEAR, LILACS, WHOLIS, and WPRIM) and followed the same quality assessment criteria described in the original literature review.^16^

All analyses were done with Stata, version 14. Our analyses are compliant with the Guidelines for Accurate and Transparent Health Estimates Reporting (GATHER) statement (appendix p 4).^17^

### Pathogen-specific pneumonia model

To estimate pathogen-specific pneumonia deaths and cases, we applied estimates of the proportion of pneumonia deaths and cases attributable to each pathogen to all-cause pneumonia mortality and morbidity estimates. All-cause pneumonia deaths and cases were prepared by the WHO Maternal and Child Epidemiology Estimation collaboration (WHO/MCEE). State-level all-cause pneumonia deaths were modelled for each year (2000–15) with verbal autopsy data from India.^7^ State-level, all-cause clinical, and severe pneumonia cases were prepared for 2000 and 2015 with a previously described risk factor prevalence model (appendix pp 10–12).^8^

We used data from randomised controlled vaccine trials with various pneumonia endpoints in the probe approach to estimate the attributable fraction for each pathogen.^1^ Since PCV and Hib vaccine trials did not assess vaccine efficacy against pneumonia mortality, we used efficacy against radiograph-confirmed, primary endpoint pneumonia as a proxy for this value. The efficacy values against clinical and severe pneumonia were used to estimate pathogen-specific pneumonia morbidity.

PCV and Hib vaccines do not prevent all pneumonia deaths or cases caused by pneumococcus and Hib, and so additional adjustments to vaccine efficacy values are required. To account for incomplete vaccine efficacy, the observed efficacy against vaccine-type invasive pneumococcal disease was used as a proxy for the efficacy against vaccine-type pneumococcal pneumonia. Efficacy for vaccine-type pneumococcal pneumonia has not been measured in childhood PCV efficacy trials. PCV efficacy values were also adjusted to account for the proportion of vaccine-type invasive pneumococcal disease in the control group and for Hib vaccine use, since all trials were done in settings with routine use of this vaccine. Parameters used for these adjustments are unique to each study, and so we made these adjustments for each trial before combining estimates across trials in random-effects meta-analyses. Given the uncertainty around the proportion of pneumonia deaths attributable to pneumococcus, we applied the same sensitivity analyses used in the estimation of global pneumococcal pneumonia deaths.^1^

The primary source of uncertainty in this approach is the between-study variability in vaccine efficacy estimates. Therefore, uncertainty bounds for the pathogen-specific pneumonia mortality and morbidity model were based on a jackknife analysis, in which one study is omitted from the analysis at a time, for the upper and lower bounds of the pneumococcal and Hib pneumonia fractions.^1^ Other sources of uncertainty were not included in the pneumonia mortality and morbidity models.

### Pathogen-specific meningitis model

Pathogen-specific meningitis deaths for each state were estimated by applying the proportion of meningitis deaths caused by pneumococcus and Hib to all-cause meningitis deaths prepared by the WHO/MCEE collaboration. We estimated the proportion of meningitis deaths attributable to each pathogen in each state using summary estimates of the proportion of meningitis cases attributable to each pathogen and adjusted pathogen-specific meningitis case fatality ratios (CFR), both from values reported in the literature (appendix pp 5–9). Reported CFR estimates represent only children with access to care. Therefore, we adjusted state pathogen-specific meningitis CFR estimates using data from the National Family and Household Survey (NFHS) on the proportion of children seeking care for pneumonia symptoms, as a proxy for care seeking for meningitis (table 1). For years missing access to care values, we assumed linear trends between available values. A 5-year moving average was used to smooth pathogen-specific CFR estimates within states. State pathogen-specific meningitis mortality estimates were divided by pathogen-specific meningitis CFR estimates to derive pneumococcal and Hib meningitis morbidity estimates.

We followed previously published methods for ascertaining uncertainty bounds for meningitis morbidity and mortality; we ascertained the most conservative (ie, widest) uncertainty for aetiological proportions based on a jackknife, leave-one-study-out analysis and the reported confidence intervals from the random-effects meta-analysis.^1^ As with the pneumonia models, uncertainty for the meningitis mortality and morbidity models did not account for other sources of uncertainty.

### Pathogen-specific invasive non-pneumonia, non-meningitis model

We used previously described methods to estimate pathogen-specific morbidity from other NPNM invasive syndromes (eg, sepsis).^1^ Briefly, we abstracted from published studies the ratio of pathogen-specific NPNM cases to pathogen-specific meningitis cases, stratified them by all-cause child mortality, and within those strata we combined ratios using random-effects meta-analyses. For each state, we applied the mortality-specific ratio from the meta-analysis to the pneumococcal or Hib meningitis cases estimates to infer the pathogen-specific NPNM cases. Pneumococcal NPNM cases were stratified by severe and non-severe cases by use of different NPNM case definitions from published studies. Pathogen-specific NPNM deaths were estimated with the ratio of pathogen-specific meningitis CFR to pathogen-specific NPNM CFR from the literature and by applying this value to pathogen-specific NPNM cases. Uncertainty ranges for pathogen-specific NPNM morbidity and mortality were based on those for pathogen-specific meningitis.^1^

### Population data

Sources of demographic and population data for each state are described in table 1. We used census data for India from 2001 and 2011 to estimate annual populations at risk. For years where child population data were not available, we used census data to estimate annual population growth rates to interpolate and extrapolate the state-level child population. We then normalised population data to sum to the total national UN population estimates for India. The Sample Registration System (SRS) for India was used to prepare annual child mortality estimates for each state.^7^ Since no state-level paediatric HIV prevalence estimates are available, we assumed the same unpublished annual national HIV prevalence from UNAIDS for each state (Mahy M, UNAIDS, personal communication).

### Adjustment for Hib vaccine use

Pathogen-specific burden estimates were calculated assuming no vaccine use. We then adjusted these estimates using estimates of Hib vaccine efficacy against invasive Hib disease^18^ and state-specific vaccine coverage estimates to account for the impact of Hib vaccine use. Since no subnational estimates of Hib vaccine coverage are publicly available, we used coverage estimates for the third dose of diphtheria-tetanus-pertussis vaccine (DTP3) from subnational surveys, including the NFHS and the Annual Health Survey (AHS), as proxies for coverage with three doses of Hib vaccine. In the year of Hib vaccine introduction, we prorated coverage estimates to account for the month when the Hib vaccine was introduced. Linear interpolation was used when data were missing for relevant years. Our model accounts for indirect protection afforded by the Hib vaccine in the first years following introduction using the same methods previously reported.^18^ We assumed no children in India received PCV before 2015, since its use in the national immunisation programme began only in 2017 in selected states.

### Reporting

India is a large and sociodemographically diverse country, comprising 29 states and seven union territories. For the purpose of this analysis, we used six geographically contiguous and socioeconomically distinct regions: north, east, northeast, central, west, and south. For state-level reporting, union territories, except for Delhi, and states with fewer than 1500 total child deaths, except for Goa, have been grouped as “northeast” and “other” on the basis of their location (table 2). Telangana separated from Andhra Pradesh in June, 2014. We projected population and mortality data for Andhra Pradesh and modelled Hib and pneumococcal disease burden for Andhra Pradesh and Telangana together for 2014 and 2015. We also report on the pathogen-specific burden estimates together for five regions and eight states that are socioeconomically less developed than others in India (Empowered Action Group [EAG] states) plus Assam (table 2). Health indicators in Assam are often reported together with EAG states given its large size and similar development challenges.

**Table 2:** Indian states by region

	**States**
Central	Chhattisgarh, Madhya Pradesh, Rajasthan, and Uttar Pradesh
East	Bihar, Jharkhand, Odisha, and West Bengal
North	Chandigarh, Delhi, Haryana, Himachal Pradesh, Jammu and Kashmir, Punjab, and Uttarakhand
Northeast	Arunachal Pradesh, Assam, Manipur, Meghalaya, Mizoram, Nagaland, Sikkim, and Tripura
South	Andaman and Nicobar Islands, Andhra Pradesh, Karnataka, Kerala, Lakshadweep, Puducherry, and Tamil Nadu
West	Dadra and Nagar Haveli, Daman and Diu, Goa, Gujarat, and Maharashtra
Empowered Action Group and Assam	Assam, Bihar, Chhattisgarh, Jharkhand, Madhya Pradesh, Odisha, Rajasthan, Uttar Pradesh, and Uttarakhand

Pathogen-specific mortality and incidence per 100 000 children aged 1–59 months for each year are reported. Reported results are rounded and reported with three significant digits or fewer; precision was not provided for fewer than 100 cases or deaths. Higher precision is provided in the appendix (pp 22–41). Morbidity and mortality estimates, unless otherwise specified, are given for children without HIV infection. Mortality and incidence estimates associated with pneumococcal and Hib disease among children with HIV infection are provided in the appendix (pp 22–41).

### Role of the funding source

The sponsor of this study had no role in study design, data collection, data analysis, data interpretation, writing of the report, or the decision to submit for publication. All authors had full access to all the data used in the study and the corresponding author had final responsibility for the decision to submit for publication.

## Results

Our literature review yielded only two studies that reported on the distribution of pathogens among children admitted to hospital with meningitis.^9^, ^10^ An additional 33 studies from Asia providing data about the distribution of meningitis cases by cause were identified. We found no studies reporting on pneumococcal meningitis case fatality in India and only three studies reporting on Hib meningitis case fatality in India.^11^, ^12^, ^13^ Several additional studies from epidemiologically relevant settings contributed data to estimates of pathogen-specific meningitis case fatality (appendix pp 5–8). The literature review also yielded two studies with data about the ratio of pneumococcal NPNM cases to pneumococcal meningitis cases from India.^14^, ^15^

In 2015, 68 700 (uncertainty range [UR] 44 600–86 000) pneumococcal deaths and 15 600 (9800–21 500) Hib deaths were estimated to have occurred in children aged 1–59 months in India. In this age group, among deaths categorised as being related to HIV/AIDS in India, we estimate that 1000 (UR 600–1200) deaths were caused by pneumococcal infection and fewer than 100 by Hib infection. At the national level, pneumococcal deaths declined by 58% (UR 22–78) since 2000, when there were 166 000 (UR 110 000–198 000) estimated pneumococcal deaths. Hib deaths declined by 81% (UR 59–91) during the same time period; in 2000, there were an estimated 82 600 (UR 52 300–112 000) Hib deaths. Despite substantial reductions in mortality, pneumococcus accounted for 14% (UR 9–17) and Hib for 3% (UR 2–4) of all deaths among children aged 1–59 months in India.

**Figure 1:**
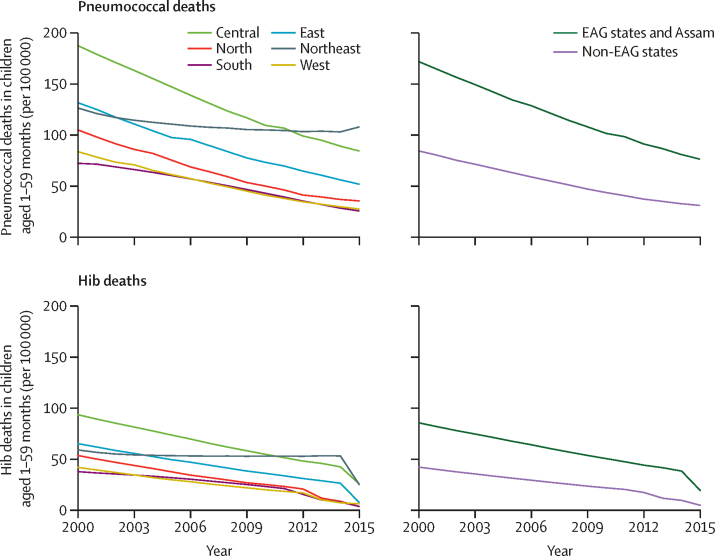
Mortality rates for *Streptococcus pneumoniae* and *Haemophilus influenzae* type b for 2000–15, by region and EAG status* EAG=Empowered Action Group. *EAG states: Bihar, Chhattisgarh, Jharkhand, Madhya Pradesh, Odisha, Rajasthan, Uttarakhand, and Uttar Pradesh. Assam was included with EAG states in this analysis.

There were regional differences in pneumococcal and Hib mortality in India in 2015. Approximately 50% of pneumococcal deaths (34 100 [UR 22 100–42 700]) and 65% of Hib deaths (10 300 [6500–14 100]) were estimated to have occurred in the central region, which accounted for only 33% of the child population in that year. 75% of pneumococcal deaths (51 600 [33 400–64 600]) and 83% of Hib deaths (13 000 [8100–17 800]) in 2015 occurred in the eight EAG states and Assam, which together account for 55% of the child population younger than 5 years. The northeast region had the highest estimated pneumococcal mortality in 2015, with approximately 108 (UR 69–137) deaths per 100 000 children aged 1–59 months, more than four times the pneumococcal mortality in the south region (26 [17–33]), which had the lowest pneumococcal mortality rate in 2015. The central (26 [UR 16–35]) and northeast (25 [15–34]) regions had the highest Hib mortality in 2015. Pneumococcal mortality rates decreased in all regions between 2000 and 2015; however, compared with all other regions, the northeast region had the smallest declines (figure 1). Hib mortality rates in all regions declined by between 57% (northeast region) and 94% (north region) between 2000 and 2015 (figure 1).

**Figure 2:**
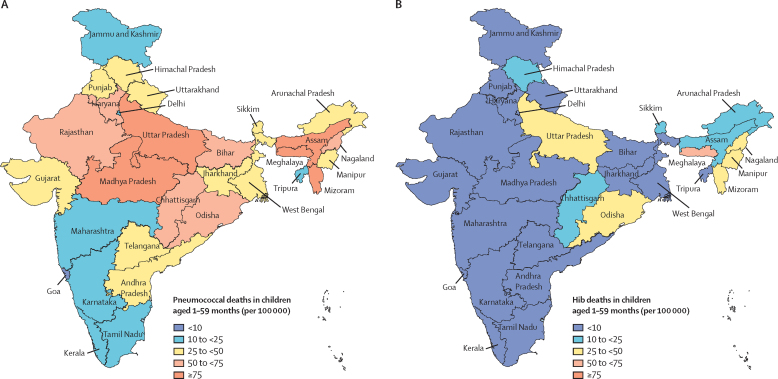
Mortality rates for *Streptococcus pneumoniae* (A) and *Haemophilus influenzae* type b (B) in 2015 by state

**Figure 3:**
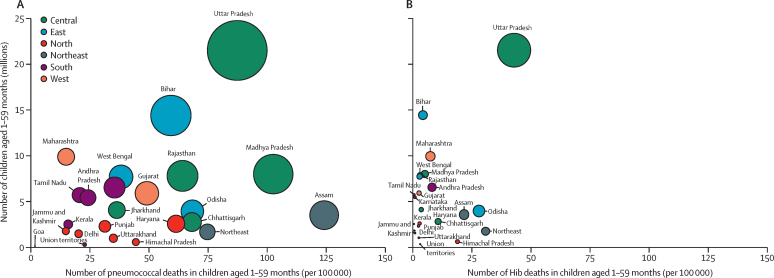
Burden of *Streptococcus pneumoniae* (A) and *Haemophilus influenzae* type b (B) deaths and mortality by state in 2015 Size of bubble indicates absolute number of pathogen-specific deaths. Y-axis shows number of children aged 1–59 months living in each state.

State-level pathogen-specific deaths and mortality rates in 2015 are provided for pneumococcal infection in table 3 and figure 2A, and figure 3, and for Hib in table 4, figure 2B, and figure 3. Uttar Pradesh, the most populous state in India, accounting for around 18% of the population younger than 5 years, had the highest number of pneumococcal and Hib deaths each year between 2000 and 2015. An estimated 18 900 (UR 12 300–23 600) pneumococcal deaths occurred in Uttar Pradesh in 2015, representing 28% of all pneumococcal deaths in that year, while 9300 (5900–12 700) Hib deaths occurred in the state, representing 60% of all Hib deaths in India in 2015. In 2000, Uttar Pradesh had the highest pneumococcal mortality (201 [UR 136–237] per 100 000 children aged 1–59 months) and Madhya Pradesh had the highest Hib mortality (99 [61–137] per 100 000 children aged 1–59 months). However, pneumococcal mortality rates in Meghalaya were higher than in all other states from 2007 onwards, as was Hib mortality from 2009 onwards, and Meghalaya continued to have the highest mortality rates for both pathogens through to 2015.

**Table 3:** Streptococcus pneumoniae mortality in Indian children aged 1–59 months without HIV infection* in 2015

	**NPNM deaths (UR)**	**NPNM mortality rate (UR)**	**Meningitis deaths (UR)**	**Meningitis mortality rate (UR)**	**Pneumonia deaths (UR)**	**Pneumonia mortality rate (UR)**	**Total pneumococcal deaths (UR)**	**Total pneumococcal mortality rate (UR)**
**States**								
Andhra Pradesh	200 (100–500)	4 (2–7)	300 (100–500)	4 (2–8)	1800 (1300–1900)	28 (20–29)	2300 (1500–2900)	36 (23–45)
Assam	500 (200–900)	13 (5–27)	500 (200–1100)	14 (6–30)	3400 (2400–3500)	97 (69–101)	4400 (2800–5500)	124 (79–158)
Bihar	900 (400–1800)	6 (3–12)	1000 (500–2000)	7 (3–14)	6700 (4700–6900)	46 (33–48)	8600 (5600–10 700)	60 (39–74)
Chhattisgarh	200 (90–400)	7 (3–14)	200 (100–400)	8 (4–16)	1500 (1000–1500)	53 (38–55)	1900 (1200–2400)	68 (44–86)
Delhi	<100	2 (1–5)	<100	3 (1–5)	200 (200–200)	15 (11–16)	300 (200–400)	21 (13–26)
Goa	<100	<1 (0–0)	<100	0 (0–1)	<100	2 (1–2)	<100	2 (1–3)
Gujarat	300 (100–600)	5 (2–10)	400 (200–700)	6 (3–11)	2300 (1600–2400)	38 (27–40)	2900 (1900–3600)	49 (32–61)
Haryana	200 (100–300)	7 (3–12)	200 (100–400)	8 (4–14)	1200 (900–1300)	47 (34–49)	1600 (1000–1900)	62 (41–75)
Himachal Pradesh	<100	5 (3–8)	<100	6 (3–9)	200 (100–200)	34 (24–35)	300 (200–300)	45 (30–53)
Jammu and Kashmir	<100	2 (1–3)	<100	2 (1–3)	200 (100–200)	12 (8–12)	300 (200–300)	15 (10–19)
Jharkhand	200 (100–300)	4 (1–8)	200 (100–400)	4 (2–9)	1200 (800–1200)	29 (20–30)	1500 (1000–1900)	37 (23–47)
Karnataka	100 (100–300)	3 (1–5)	200 (100–300)	3 (2–5)	1000 (700–1100)	19 (13–20)	1300 (900–1600)	24 (16–30)
Kerala	<100	2 (1–4)	<100	2 (1–4)	300 (200–300)	12 (9–13)	400 (300–500)	16 (11–20)
Madhya Pradesh	900 (300–1800)	11 (4–22)	1000 (400–2000)	12 (5–25)	6400 (4600–6700)	80 (57–84)	8200 (5300–10 400)	103 (66–130)
Maharashtra	200 (100–400)	2 (1–4)	200 (100–400)	2 (1–4)	1200 (800–1200)	12 (8–12)	1500 (1000–2000)	15 (10–20)
Northeast	<100	5 (2–10)	200 (100–300)	9 (4–19)	1000 (700–1100)	61 (43–64)	1300 (800–1600)	75 (49–93)
Odisha	300 (100–600)	7 (3–15)	300 (100–600)	8 (3–17)	2100 (1500–2200)	54 (38–56)	2700 (1700–3400)	69 (44–87)
Punjab	<100	4 (2–7)	<100	4 (2–8)	500 (400–600)	24 (17–25)	700 (500–900)	32 (21–40)
Rajasthan	500 (200–1000)	7 (3–13)	600 (300–1200)	8 (3–15)	3900 (2800–4100)	50 (35–52)	5000 (3300–6300)	64 (42–80)
Tamil Nadu	100 (100–300)	2 (1–5)	100 (100–300)	3 (1–6)	900 (700–1000)	16 (11–17)	1200 (800–1600)	21 (13–28)
Union territories	<100	3 (1–5)	<100	3 (2–6)	<100	17 (12–18)	<100	23 (15–28)
Uttar Pradesh	2000 (900–3900)	9 (4–18)	2200 (1000–4400)	10 (5–20)	14 700 (10 400–15 300)	68 (48–71)	18 900 (12 300–23 600)	88 (57–109)
Uttarakhand	<100	4 (1–7)	<100	4 (2–8)	300 (200–300)	27 (19–29)	300 (200–400)	35 (23–45)
West Bengal	300 (200–600)	4 (2–8)	400 (200–700)	5 (2–9)	2300 (1600–2400)	30 (21–31)	3000 (2000–3600)	38 (25–47)
**Regions**								
Central	3600 (1600–7100)	9 (4–18)	4000 (1800–8000)	10 (4–20)	26 500 (18 800–27 600)	66 (47–69)	34 100 (22 100–42 700)	85 (55–106)
East	1700 (800–3300)	6 (2–11)	1900 (800–3700)	6 (3–12)	12 200 (8700–12 700)	40 (29–42)	15 800 (10 300–19 600)	52 (34–65)
North	400 (200–700)	4 (2–7)	400 (200–800)	5 (2–8)	2700 (1900–2800)	27 (19–28)	3500 (2300–4300)	36 (24–44)
Northeast	500 (200–1100)	10 (4–21)	700 (300–1400)	13 (5–26)	4400 (3100–4600)	85 (61–89)	5600 (3600–7100)	108 (69–137)
South	600 (300–1100)	3 (1–6)	600 (300–1300)	3 (1–6)	4100 (2900–4300)	20 (14–21)	5300 (3400–6700)	26 (17–33)
West	500 (200–1000)	3 (1–6)	500 (200–1100)	3 (2–7)	3400 (2400–3600)	21 (15–22)	4500 (2900–5600)	28 (18–35)
National	7200 (3200–14 300)	6 (3–12)	8200 (3600–16 200)	7 (3–13)	53 300 (37 800–55 500)	44 (31–46)	68 700 (44 600–86 000)	56 (37–71)

Rates are cases or deaths per 100 000 children aged 1–59 months. NPNM=non-pneumonia, non-meningitis. UR=uncertainty range.

* Burden among children with HIV infection included in the appendix (pp 22–41).

**Table 4:** *Haemophilus influenzae* type b mortality in Indian children aged 1–59 months without HIV infection* in 2015

	**NPNM deaths (UR)**	**NPNM mortality rate (UR)**	**Meningitis deaths (UR)**	**Meningitis mortality rate (UR)**	**Pneumonia deaths (UR)**	**Pneumonia mortality rate (UR)**	**Total pneumococcal deaths (UR)**	**Total pneumococcal mortality rate (UR)**
**States**								
Andhra Pradesh	<100		100 (100–200)	2 (1–3)	400 (300–500)	6 (4–8)	500 (300–700)	8 (5–11)
Assam	<100	0 (0–0)	200 (100–300)	6 (2–9)	600 (400–700)	16 (11–21)	800 (500–1100)	22 (13–30)
Bihar	<100	0 (0–0)	200 (100–200)	1 (0–2)	500 (300–600)	3 (2–4)	600 (400–900)	4 (3–6)
Chhattisgarh	<100	0 (0–0)	<100	3 (1–4)	200 (200–300)	8 (6–11)	300 (200–400)	11 (7–15)
Delhi	<100	0 (0–0)	<100	0 (0–0)	<100	1 (1–1)	<100	1 (1–2)
Goa	<100	0 (0–0)	<100	0 (0–0)	<100	0 (0–0)	<100	0 (0–0)
Gujarat	<100	0 (0–0)	<100	1 (0–1)	100 (100–200)	2 (1–3)	200 (100–200)	3 (2–4)
Haryana	<100	0 (0–0)	<100	1 (0–1)	<100	2 (2–3)	<100	3 (2–4)
Himachal Pradesh	<100	0 (0–0)	<100	5 (3–7)	<100	14 (10–19)	100 (100–100)	19 (13–26)
Jammu and Kashmir	<100	0 (0–0)	<100	0 (0–0)	<100	1 (0–1)	<100	1 (1–1)
Jharkhand	<100	0 (0–0)	<100	1 (0–2)	100 (100–200)	3 (2–4)	200 (100–200)	4 (2–5)
Karnataka	<100	0 (0–0)	<100	0 (0–0)	<100	1 (1–1)	<100	1 (1–1)
Kerala	<100	0 (0–0)	<100	0 (0–0)	<100	1 (0–1)	<100	1 (0–1)
Madhya Pradesh	<100	0 (0–0)	100 (0–200)	1 (1–2)	300 (200–400)	4 (3–5)	400 (300–600)	5 (3–7)
Maharashtra	<100	0 (0–0)	200 (0–300)	2 (0–3)	600 (400–800)	6 (4–8)	800 (500–1100)	8 (5–11)
Northeast	<100	0 (0–0)	100 (0–200)	8 (3–13)	400 (300–500)	23 (16–30)	500 (300–700)	31 (19–43)
Odisha	<100	0 (0–0)	300 (0–400)	7 (3–11)	800 (600–1100)	21 (15–27)	1110 (700–1500)	28 (17–39)
Punjab	<100	0 (0–0)	<100	1 (0–1)	<100	2 (1–3)	<100	3 (2–4)
Rajasthan	<100	0 (0–0)	<100	1 (0–1)	200 (100–200)	2 (2–3)	300 (200–300)	3 (2–4)
Tamil Nadu	<100	0 (0–0)	<100	0 (0–0)	<100	1 (0–1)	<100	1 (0–1)
Union Territories	<100	0 (0–0)	<100	1 (0–1)	<100	3 (2–3)	<100	3 (2–5)
Uttar Pradesh	<100	0 (0–0)	2400 (1000–3600)	11 (5–17)	6900 (4900–9100)	32 (22–42)	9300 (5900–12 700)	43 (27–59)
Uttarakhand	<100	0 (0–0)	<100	1 (0–1)	<100	2 (1–3)	<100	3 (2–4)
West Bengal	<100	0 (0–0)	<100	1 (0–1)	200 (100–200)	2 (2–3)	200 (200–300)	3 (2–4)
**Regions**								
Central	<100	0 (0–0)	2600 (1100–4000)	7 (3–10)	7600 (5400–10 000)	19 (13–25)	10 300 (6500–14 100)	26 (16–35)
East	<100	0 (0–0)	500 (200–900)	2 (1–3)	1600 (1100–2100)	5 (4–7)	2100 (1300–2900)	7 (4–10)
North	<100	0 (0–0)	<100	1 (0–1)	200 (200–300)	2 (2–3)	300 (200–400)	3 (2–4)
Northeast	<100	0 (0–0)	300 (100–500)	6 (2–10)	1000 (700–1300)	18 (13–24)	1300 (800–1780)	25 (15–34)
South	<100	0 (0–0)	200 (100–300)	1 (0–1)	500 (300–700)	2 (2–3)	700 (400–900)	3 (2–5)
West	<100	0 (0–0)	200 (100–400)	1 (0–2)	700 (500–900)	4 (3–6)	900 (600–1300)	6 (4–8)
National	<100	0 (0–0)	4000 (1600–6100)	3 (1–5)	11 600 (8200–15 300)	10 (7–13)	15 600 (9800–21 500)	13 (8–18)

Rates are cases or deaths per 100 000 children aged 1–59 months. NPNM=non-pneumonia, non-meningitis. UR=uncertainty range.

* Burden among children with HIV infection included in the appendix (pp 22–41).

Of the three syndromes associated with pneumococcal and Hib infection, pneumonia accounted for the greatest burden of pathogen-specific mortality in India and in every state between 2000 and 2015. Pneumonia accounted for 78% (53 300 [UR 37 800–55 500]) of the all-syndrome pneumococcal deaths estimated in 2015, whereas meningitis accounted for 12% (8200 [3600–16 200]) and NPNM accounted for 11% (7200 [3200–14 500]). To be able to compare our findings with other published pneumococcal mortality estimates, we estimated there were 76 700 (UR 54 400–79 900) pneumococcal pneumonia deaths, 11 800 (5600–22 700) meningitis deaths, and 7200 (3400–13 800) NPNM deaths in children aged 1–59 months in 2010. Of the estimated Hib deaths in the same year, 74% (11 600 [UR 8200–15 300]) were due to pneumonia, 25% (4000 [1600–6100]) due to meningitis, and less than 1% (<100) due to NPNM.

As previously reported,^1^ we estimated pneumococcus to account for 34% (UR 24–36) and Hib to account for 21% (15–28) of radiograph-confirmed pneumonia cases; we used those case-derived proportions as a proxy for the fraction of pneumonia deaths attributable to these two pathogens, in settings where PCV and Hib vaccine are not used.^1^ Our sensitivity analysis found that the proportion of radiograph-confirmed pneumonia that is pneumococcus could be as high as 51% (UR 15–87), in which case there could have been as many as 102 000 (UR 62 200–110 000) estimated pneumococcal pneumonia deaths and therefore 118 000 (69 000–140 000) pneumococcal deaths in 2015. State-level pneumococcal pneumonia deaths based on this sensitivity analysis are provided in the appendix (pp 42–57).

We estimated 1·6 million (UR 1·2–1·9) cases of severe pneumococcal disease (ie, severe pneumococcal pneumonia, pneumococcal meningitis, and severe NPNM) in 2015. There were 2·7 million (UR 2·0–3·1) estimated severe pneumococcal cases in 2000, representing a reduction of almost 40%. Uttar Pradesh (367 000 [UR 271 000–428 000]), Bihar (238 000 [177 000–276 000]), and Maharashtra (149 000 [111 000–171 000]) had the highest numbers of estimated severe pneumococcal cases in 2015. Madhya Pradesh (1767 [UR 1304–2068]), Uttar Pradesh (1697 [1254–1982]), and Bihar (1648 [1223–1912]) had the highest incidence of severe pneumococcal disease per 100 000 children aged 1–59 months in 2015. Severe pneumococcal disease in India manifests primarily as severe pneumonia. There were 1·6 million (UR 1·2–1·8) estimated cases of severe pneumococcal pneumonia in 2015, accounting for more than 97% of all severe pneumococcal disease.

By 2015, severe Hib disease had declined in India to 236 000 cases (UR 138 000–468 000), a substantial decline from the 883 000 (517 000–1 750 000) estimated cases in 2000. More than 73% of severe Hib cases in 2015 occurred in three states that together only accounted for 30% of the child population in that same year: Uttar Pradesh (UR 120 000 [703 000–238 000]), Maharashtra (39 000 [22 900–78 200]), and Odisha (13 600 [7900–27 100]).

## Discussion

We estimated the comprehensive (ie, including all relevant syndromes) subnational pneumococcal and Hib morbidity and mortality burden for children aged 1–59 months in India. Our analysis found that, even before introduction of the PCV or Hib vaccine, pneumococcal and Hib deaths in children had declined substantially since 2000. Despite substantial reductions in mortality, pneumococcus accounted for 14% (UR 9–17) and Hib for 3% (UR 2–4) of all deaths among children aged 1–59 months in India. These deaths are largely preventable with specific interventions such as vaccines that prevent the occurrence of cause-specific disease and with early and effective treatment. These estimates can be used to monitor progress towards achieving child survival targets and inform the impact of disease prevention and treatment strategies.

The reduction in deaths from these two pathogens before the introduction and widespread use of the PCV and Hib vaccine reflects overall child survival trends in India during this period.^19^ The launch of the National Rural Health Mission (NRHM) in 2005, now the National Health Mission (NHM), has helped to expand and improve primary maternal and child health services in the country, which is likely to have contributed to improvements in overall child survival.^20^, ^21^ Our model quantified the accelerated decline in Hib deaths after 2012, during which the Hib vaccine was introduced in many states. Uttar Pradesh, which accounted for approximately 60% of Hib deaths, was the last state to initiate routine use of the Hib vaccine in December, 2015.

A disproportionate number of pneumococcal and Hib deaths continue to occur in states located in the eastern and central regions. The states in these two regions, excluding West Bengal, comprise the EAG states. Disparities between EAG states (inclusive of Assam) and non-EAG states, represented by the difference between pneumococcal and Hib mortality between these two groups, diminished between 2000 and 2015. The NHM's efforts have been most intensive in EAG states and in states in the northeast region, including Assam. This is likely to have contributed to a reduction in these disparities over time. The estimates imply that introduction of the Hib vaccine in many EAG states and Assam throughout 2014 and 2015 has contributed to further minimisation of the differences in Hib mortality rates between these two groups of states (figure 1). The recent subnational introduction of PCV-13 in 2017 and its projected national rollout over the coming years is expected to further reduce differences in pneumococcal and all-cause mortality.

Similar to global burden estimates, pneumonia dominated the syndromic distribution of deaths associated with pneumococcus and Hib in India. As a result, disease burden estimates for both pathogens are highly sensitive to changes in estimates for all-cause pneumonia mortality and the fraction of pneumonia deaths associated with each pathogen. In the probe approach, we used estimates of the proportion of radiograph-confirmed pneumonia cases caused by pneumococcus and Hib as proxy values for the proportion of pneumonia deaths associated with each pathogen. This approach results in the inherent inference that the case fatality estimates for radiograph-confirmed pneumonia caused by pneumococcus, Hib, and non-pneumococcus or non-Hib are identical, which is likely to be incorrect. Without access to timely and effective antibiotics, the case fatality for bacterial causes of pneumonia is likely to be higher than that for non-bacterial pneumonia. Most deaths from pneumonia in India occur in the community, rather than in health-care facilities, indicating that many individuals who develop pneumonia probably do not have access to antibiotics.^22^ As a result, our approach has a direction of bias that is likely to underestimate state-level mortality rates and deaths from pneumococcal and Hib pneumonia.

The vaccine probe approach also requires adjustment for incomplete vaccine efficacy. As a proxy for vaccine-type pneumococcal pneumonia efficacy in children, we used efficacy against vaccine-type invasive pneumococcal diseases, which is likely to be higher than vaccine-type pneumococcal pneumonia efficacy. If true, this approach also underestimates the contribution of pneumococcus to deaths from pneumonia. We did a sensitivity analysis using data from a PCV clinical trial with a pathogen-specific pneumonia efficacy outcome done in an elderly population in the Netherlands^23^ to assess the magnitude of potential underestimation. We found that deaths from pneumococcal pneumonia could be as much as 50% higher than our current estimates.^1^ Others have included this adjustment in their base case model for global pneumococcal pneumonia mortality estimates;^24^ we report the results of our sensitivity analysis at the state level in the appendix (pp 42–57).

Our subnational model allows us to account for differences in vaccine coverage between states. Without accounting for subnational differences in vaccine coverage, the impact of new vaccines on pathogen-specific disease would be biased. In the case of the Hib vaccine, which was first used in states with low child mortality, accounting for vaccine use at the state level restricts the degree to which the effects of the Hib vaccine are overestimated in the years following the introduction of vaccine. We used state-level DTP3 coverage from two different surveys as a proxy for state-level Hib vaccine coverage. In the routine immunisation programme in India, the Hib vaccine is delivered together with four other antigens (ie, DTP and hepatitis B) as a pentavalent vaccine.

The 13-valent PCV was introduced in 2017 in three states with relatively high child mortality. Researchers who didan invasive pneumococcal disease surveillance study between 2011 and 2015 in India concluded that the 13-valent PCV product includes serotypes responsible for 74% of invasive pneumococcal disease nationwide.^25^ Although subnational PCV coverage estimates in the private sector have been published for 2012,^26^ we did not use these estimates in our model as the overall national coverage was reported to be lower than 1%. Additionally, those who received PCV in the private sector are probably already at low risk of pneumococcal disease and mortality than those without access to PCV in the private sector.

Subnational pneumococcal pneumonia morbidity and mortality burden estimates for 2010 have been published.^6^ We prepared annual estimates of subnational pneumococcal and Hib deaths by syndrome between 2000 and 2015 so that we could compare our estimates with the published literature. In 2010, we estimated there were 76 700 (UR 54 400–79 900) deaths from pneumococcal pneumonia in Indian children aged 1–59 months (appendix pp 29–34), whereas Farooqui and colleagues^6^ estimated 105 100 (UR 91 100–120 000) deaths from pneumococcal pneumonia. Although there are quantitative differences between our estimates and those of Farooqui and colleagues^6^ at the state level, the rank order of states based on deaths from pneumococcal pneumonia is similar across the two models (ie, the four states with the greatest burden are the same in both models), indicating that the use of either set of estimates for state-specific PCV prioritisation would lead to the same conclusions. The conceptual approaches used to prepare estimates of pneumococcal pneumonia deaths differ between the two models, which largely account for the quantitative discrepancies between the estimates prepared by Farooqui and colleagues and our estimates. A state-level comparison of results from both models and the details of the different approaches is provided in the appendix (p 20).

India has previously introduced new vaccines into the national immunisation programme in a phased manner. The approach for state selection has differed by vaccine, and decision makers might consider a range of factors for which subnational data are available, including disease burden, potential vaccine impact, indicators of equity, health-system readiness, and routine immunisation coverage. India has usually initiated introductions in states and union territories with relatively strong health systems. However, the introduction of PCV marked a shift in this approach, prioritising on the basis of child mortality rather than health-system strength or vaccine coverage. The availability of subnational estimates enables evidence-based strategies for state prioritisation; in particular, such estimates allow India to develop equity-focused and impact-focused approaches to PCV rollout and subsequent new vaccine introductions.

Policy makers increasingly consider mortality and morbidity burden estimates together when making decisions about the introduction of new vaccines. Morbidity burden estimates are also important variables in vaccine cost-effectiveness analyses.^27^ Until now, subnational morbidity burden estimates for pneumococcus have only been available for severe pneumonia. Although this syndrome accounts for a large fraction of severe pneumococcal diseases, bacterial meningitis cases in India often result in severe, long-term neurological impairments.^28^, ^29^ These morbidity burden estimates are necessary for cost-of-illness assessments. To contribute to these efforts, we prepared subnational pneumococcal and Hib morbidity estimates for each syndrome associated with these pathogens.

In addition to the challenges associated with the vaccine probe approach described above, our approach for estimating the subnational burden of pneumococcal and Hib disease in India has other limitations. Although our estimates inherently account for state-level disease risk factors and access to care differences, additional disparities within states and populations remain. Our models do not account for these disparities. All-cause child mortality estimates are now available with higher spatial resolution (ie, at the district level).^30^ These estimates could serve as the basis for future pathogen-specific mortality estimates at the district level in India. However, such estimates would require additional district-level input data, including PCV and Hib vaccine coverage. This information would be useful for future pneumococcal disease burden estimates, given the district-wise introduction strategy for PCV.

Another limitation of our model is the continued reliance on verbal autopsy data for all-cause pneumonia and meningitis deaths.^7^ Verbal autopsy data are susceptible to misclassification and other challenges that have been described extensively.^31^, ^32^ These challenges are especially relevant for deaths from non-specific syndromes such as pneumonia. Nationally representative cause-of-death data that have been directly measured, as opposed to modelled estimates, are now available.^33^ Pneumococcal and Hib disease burden estimates based on these cause-of-death data could complement the estimates provided here. Additionally, strengthened vital registration systems and medical cause of death reporting in India would help improve confidence in cause-specific disease burden estimates.

We used data from India or its regional neighbours wherever possible. Population-at-risk and demographic data came largely from Indian Government sources. The pathogen-specific pneumonia models use data from vaccine clinical trials done in several countries around the world. The implications, assumptions, and possible consequential underestimation or overestimation from the pathogen-specific component of the model are discussed elsewhere.^1^ Another limitation of our estimates is the absence of data from India about pathogen-specific meningitis and NPNM. We therefore relied on data from studies done in geographically and epidemiologically relevant settings. Additional pathogen-specific data from observational studies done in India would help improve disease burden estimates.

Our subnational estimates of mortality and morbidity caused by pneumococcus and Hib suggest that India has made substantial progress in reducing mortality and morbidity from these diseases in the past 15 years and that the recent Hib vaccine introductions have further accelerated these reductions. The introduction and expanded use of these vaccines as planned by the government has the potential to substantially accelerate the reduction of childhood pneumococcal and Hib morbidity and mortality towards meeting the Sustainable Development Goal child survival targets by 2030.

For more on **VIEW-hub** see http://www.view-hub.org/
