# Supplementary material for: National, regional, and state-level burden of Streptococcus pneumoniae and Haemophilus influenzae type b disease in children in India: modelled estimates for 2000–15
Source: Lancet Glob Health. 2019 May 13;7(6):e735–47. doi: 10.1016/S2214-109X(19)30081-6 (PMC6527518; doi:10.1016/S2214-109X(19)30081-6)
Supplement: Supplementary appendix [file mmc1.pdf]

THE LANCET
Global Health

Supplementary appendix

This appendix formed part of the original submission and has been peer reviewed. We post it as supplied by the authors.

Supplement to: Wahl B, Sharan A, Knoll MD, et al. National, regional, and state-level burden of *Streptococcus pneumoniae* and *Haemophilus influenzae* type b disease in children in India: modelled estimates for 2000–15. *Lancet Glob Health* 2019; **7:** e735–47

The state-level burden of *Streptococcus pneumoniae* and *Haemophilus influenzae* type b disease in children 1-59 months of age in India from 2000-2015

Brian Wahl,[*1] Apoorva Sharan,[*2], Maria Deloria Knoll,[1] Rajesh Kumar,[3] Li Liu,[4,5] Yue Chu,[4] David A McAllister,[6] Harish Nair,[7,8] Harry Campbell,[7] Igor Rudan,[7] Usha Ram,[9] Molly Sauer,[1] Anita Shet,[1] Robert Black,[4] Mathuram Santosham,[1] Katherine L O'Brien,[1**] Narendra K Arora,[2**]

[1] International Vaccine Access Center (IVAC), Department of International Health, Johns Hopkins Bloomberg School of Public Health, Baltimore, Maryland, USA
[2] The INCLEN Trust, New Delhi, India
[3] School of Public Health, Post Graduate Institute of Medical Education and Research, Chandigarh, India
[4] Institute for International Programs, Department of International Health, Johns Hopkins Bloomberg School of Public Health, Baltimore, Maryland, USA
[5] Department of Population, Family and Reproductive Health, Johns Hopkins Bloomberg School of Public Health, Baltimore, Maryland, USA
[6] Institute of Health and Wellbeing, University of Glasgow, Glasgow, Scotland, UK
[7] Centre for Global Health Research, Usher Institute of Population Health Sciences and Informatics, Medical School, The University of Edinburgh, Edinburgh, Scotland, UK
[8] Public Health Foundation of India, New Delhi, India
[9] Department of Public Health & Mortality Studies, International Institute for Population Sciences, Mumbai, India

Correspondence: Brian Wahl, International Vaccine Access Center (IVAC), Department of International Health, Johns Hopkins Bloomberg School of Public Health, 415 N Washington Street, Floor 5, Baltimore, MD 21231, USA, bwahl@jhu.edu

भारत में बच्चों में *स्ट्रेप्टोकोकस न्यूमोनिये* और *हीमोफिलस इन्फ्लुएंजे* टाइप बी प्रकार के रोग के राष्ट्रीय, क्षेत्रीय, और राज्य-स्तर का बोझ: 2000-2015 के लिए मॉडल किए गए अनुमान

ब्रायन वाहल पी.एच.डी.,* [1] अपूर्वा शरन एस.सी.एम.,* [2] मारिया डेलोरिया नोल पी.एच.डी.,[1] प्रो० राजेश कुमार एम.डी.,[3] ली लियू पी.एच.डी., [4,5] युए चू एम.एस.पी.एच., [4] डेविड ए मैकलिस्टर एम.डी., [6] प्रो० हरीश नायर पी.एच.डी., [7,8] प्रो० हैरी कैंपबेल एम.डी., [7] प्रो० इगोर रुदन एम.डी., [7] प्रो० ऊषा राम पी.एच.डी., [9] मौली सौएर एम.पी.एच., [1] अनीता शेट एम.डी., [1] प्रो० रॉबर्ट ब्लैक एम.डी., [4] प्रो० मथुराम संतोषम एम.डी., [1] प्रो० कैथरीन एल ओ'ब्रायन एम.डी., [1] ** प्रो० नरेंद्र के अरोड़ा एम.डी. [2] **

[1] अंतर्राष्ट्रीय वैक्सीन प्रवेश केंद्र (आइवाक), अंतर्राष्ट्रीय स्वास्थ्य विभाग, जॉन्स हॉपकिंस ब्लूमबर्ग स्कूल ऑफ पब्लिक हेल्थ, बाल्टीमोर, मैरीलैंड, यू.एस.ए.
[2] इन्क्लेन ट्रस्ट इंटरनेशनल, नई दिल्ली, भारत
[3] स्कूल ऑफ पब्लिक हेल्थ, पोस्ट ग्रेजुएट इंस्टीट्यूट ऑफ मेडिकल एजुकेशन एंड रिसर्च, चंडीगढ़, भारत
[4] अंतर्राष्ट्रीय कार्यक्रमों के लिए संस्थान, अंतर्राष्ट्रीय स्वास्थ्य विभाग, जॉन्स हॉपकिंस ब्लूमबर्ग स्कूल ऑफ पब्लिक हेल्थ, बाल्टीमोर, मैरीलैंड, यू.एस.ए.
[5] जनसंख्या, परिवार और प्रजनन स्वास्थ्य विभाग, जॉन्स हॉपकिंस ब्लूमबर्ग स्कूल ऑफ पब्लिक हेल्थ, बाल्टीमोर, मैरीलैंड, यू.एस.ए.
[6] स्वास्थ्य और कल्याण संस्थान, ग्लासगो विश्वविद्यालय, ग्लासगो, स्कॉटलैंड, यू.के.
[7] ग्लोबल हेल्थ रिसर्च सेन्टर, उशर इंस्टीट्यूट ऑफ पॉपुलेशन हेल्थ साइंसेज एंड इंफॉर्मेटिक्स, मेडिकल स्कूल, द यूनिवर्सिटी ऑफ एडिनबर्ग, एडिनबर्ग, स्कॉटलैंड, यू.के.
[8] पब्लिक हेल्थ फाउंडेशन ऑफ इंडिया, नई दिल्ली, भारत
[9] पब्लिक हेल्थ और मृत्यु दर विभाग, अंतर्राष्ट्रीय जनसंख्या विज्ञान संस्थान, मुंबई, भारत

पत्राचार: Brian Wahl, International Vaccine Access Center (IVAC), Department of International Health, Johns Hopkins Bloomberg School of Public Health, 415 N Washington Street, Floor 5, Baltimore, MD 21231, USA, bwahl@jhu.edu

पृष्ठभूमि: भारत का दुनिया में बचपन की बीमारियों के लिए अधिकतर योगदान है। नीतियों को सूचित करने और बाल स्वास्थ्य लक्ष्यों को प्राप्त करने की दिशा में प्रगति को मापने के लिए , हमने 2000 और 2015 के बीच *स्ट्रेप्टोकोकस न्यूमोनिये* और *हीमोफिलस इन्फ्लुएंजे* टाइप बी के लिए वार्षिक राष्ट्रीय और राज्य-विशिष्ट बचपन मृत्यु दर और रोगों की तरफ़ योगदान का अनुमान लगाया।

विधियाँ: वैक्सीन क्लिनिकल परीक्षण डेटा का उपयोग न्यूमोकॉकस और हिब के कारण निमोनिया से होने वाली मौतों के अनुपात का अनुमान लगाने के लिए किया गया था। मेनिन्जाइटिस से होने वाली मौतों में जिम्मेदार प्रत्येक रोगाणु के लिए का अनुपात रोगाणु-विशेष मेनिन्जाइटिस मामले की घातकता और जीवाणु मेनिन्जाइटिस मामले की स्तिथि के कारणों के तरीके के (एटिओलॉजी) डेटा को निगरानी अध्ययनों से लिया गया था। हमने विश्व स्वास्थ्य संगठन (WHO) मातृ एवं शिशु महामारी विज्ञान आकलन (MCEE) सहयोग (WHO/MCEE) द्वारा भारत में मौखिक शव परीक्षण अध्ययनों के आधार पर तैयार किए गए 2000 से 2015 तक मॉडल-विशेष निमोनिया और मेनिन्जाइटिस से होने वाली मौतों के अनुपातों को लागू किया। न्यूमोकोकस और हिब के कारण नैदानिक (क्लीनिकल) और गंभीर निमोनिया के मामलों का बोझ टीका नैदानिक परीक्षण डेटा का उपयोग करके और भारत से जोखिम कारक प्रसार डेटा का उपयोग करके डब्ल्यूएचओ/एमसीईई द्वारा तैयार राज्य-विशेष सभी कारण निमोनिया मामलों का अनुमान निर्धारित किया गया था। रोगाणु-विशेष मेनिन्जाइटिस के मामलों को राज्य-स्तरीय मॉडल-विशेष मेनिन्जाइटिस से हुई मौतों और राज्य-स्तरीय मैनिंजाइटिस के मामलों के घातक अनुमानों से लिया गया था। गैर-निमोनिया, मेनिनजाइटिस (एनपीएनएम) के कारण न्यूमोकोकल और हिब रोगाणु आक्रामक लक्षणों को रोगाणु-विशेष एनपीएनएम (NPNM) से रोगाणु-विशेष मेनिन्जाइटिस मामलों को राज्य स्तर रोगाणु-विशेष मेनिन्जाइटिस मामलों के अनुपात को लागू कर प्राप्त किया गया। रोगाणु-विशेष NPNM के कारण मृत्यु की गणना न्यूमोकोकल और हिब मैनिंजाइटिस मामले की मृत्यु दर के अनुपात का उपयोग करके न्यूमोकोकल और हिब मैनिंजाइटिस NPNM मामले की मृत्यु दर के साथ की गई। भारत जनगणना के मुहैया आंकड़े जनसंख्या को जोखिम में दर्शाते हैं।

निष्कर्ष: भारतीय बच्चों में (1-59 महीने) न्यूमोकोकल और हिब के मामलों में मृत्यु का अनुमान 166,000 से गिर कर (अनिश्चितत रेंज [यूआर]: 110,000-198,000) और 82,600 (UR: 52,300-112,000) मृत्यु 2000 से 68,700 (UR: 44,600-86,000) और 15,600 (UR: 9,800-21,500) मृत्यु 2015 में क्रमश: था। यह न्यूमोकॉकल मौतों में 58% (UR: 22-78%) की गिरावट को दर्शाता है और हिब से हुई मौतों में 81% (UR: 59-91%) की गिरावट है। 2015 के राष्ट्रीय न्यूमोकोकल और हिब मृत्यु दर 56 (यूआर: 37-71) और 13 (यूआर: 8-18) प्रति 100,000 बच्चों (1-59 महीने) की मृत्यु क्रमशः थी। 2015 में उत्तर प्रदेश (18,900 [यूआर: 12,300-23,600]) और बिहार (8,600 [5,600-10,700]) में सबसे ज्यादा न्यूमोकोकल मौतें हुईं। उत्तर प्रदेश (9,300 [यूआर: 5,900-12,700) और ओडिशा (1,100 [यूआर: 700) -1,500]) में 2015 में सबसे अधिक हिब मौतें हुई। न्यूमोकॉकस के कारण निमोनिया से होने वाली मौतों के अनुपात से संबंधित रूढ़िवादी धारणाएं बताती हैं कि भारत में 2015 में कुल निमोनिया से 118,000 (UR: 69,000-140,000) मौतें हो सकती हैं।

व्याख्या: संयुग्म (कोंजूगेट) टीके के राष्ट्रव्यापी कार्यान्वयन से पहले ही 2000 के बाद से भारत में 1-59 महीने के बच्चों में न्यूमोकोकल और हिब से मृत्यु दर में गिरावट आई है। कई राज्यों में हिब वैक्सीन (टीका) की शुरूआत से हिब रोग से जुड़ी रुग्णता और मृत्यु दर में अधिक तेजी से कमी आई। न्यूमोकोकल कंजुगेट वैक्सीन (पीसीवी टीका) का तेजी से और व्यापक पैमाने पर उपयोग और हिब वैक्सीन (टीका) का निरंतर उपयोग भारत में पीड़ित बच्चों के स्वास्थ्य लक्ष्यों को तेजी से प्राप्त करने में मदद कर सकता है।

Webappendix 1: GATHER checklist

Table 1: GATHER Checklist

GATHER checklist item	Section(s) or sources providing information
**Objectives and funding**	
1. Define the indicators, populations, and time periods for which estimates were made.	Indicators, populations, and time periods provided in methods section.
2. List the funding sources for the work.	Funding source provided in summary section.
**Data inputs**	
3. Describe how the data were identified and how the data were accessed.	Data identification and collection approaches provided in methods section and in previous publications.[1 2]
4. Specify the inclusion and exclusion criteria. Identify all ad-hoc exclusions.	Inclusion and exclusion criteria provided will be in webappendix and are in previous publications.[1 2]
5. Provide information about all included data sources and their main characteristics. For each data source used, report reference information or contact name/institution, population represented, data collection method, years of data collection, sex and age range, diagnostic criteria or measurement method, and sample size, as relevant.	Data sources and characteristics provided in webappendix and in online open access database.
6. Identify and describe any categories of input data that have potentially important biases (e.g., based on characteristics listed in item 5).	Potentially important biases provided in discussion.
7. Describe and give sources for any other data inputs.	Other sources of data described in methods.
8. Provide all data inputs in a file format from which data can be efficiently extracted, including all relevant meta-data listed in item 5. For any data inputs that cannot be shared because of ethical or legal reasons provide a contact name or the name of the institution that retains the right to the data.	Data inputs available in Excel spreadsheet in online open access database when appropriate. Data inputs that cannot be shared will be described in the webappendix with institutional contacts for potentially obtaining these data.
**Data analysis**	
9. Provide a conceptual overview of the data analysis method. A diagram may be helpful.	Conceptual model provided in Figure 1 of previous publication.[3]
10. Provide a detailed description of all steps of the analysis, including mathematical formulae. This description should cover, as relevant, data cleaning, data pre-processing, data adjustments and weighting of data sources, and mathematical or statistical models.	Detailed description of analysis provided in methods, appendix, and previous publications.[1 3-5]
11. Describe how candidate models were evaluated and how the final models were selected.	Discussion of candidate model considerations provided in methods and discussed in detail in previous publication.[3]
12. Provide the results of an evaluation of model performance, if done, as well as the results of any relevant sensitivity analysis.	Results of model performance at country-level provided in results and in the webappendix.
13. Describe methods of calculating uncertainty of the estimates. State which sources of uncertainty were, and were not, accounted for in the uncertainty analysis.	Uncertainty methods and sources provided in methods.
14. State how analytic or statistical source code used to generate estimates can be accessed.	Access to source code provided in online open access database.
**Results and discussion**	
15. Provide published estimates in a file format from which data can be efficiently extracted.	Results in Excel spreadsheet provided in online open access database.
16. Report a quantitative measure of the uncertainty of the estimates.	Uncertainty intervals provided with all results in manuscript, will be in the webappendix, and online open access database.
17. Interpret results in light of existing evidence. If updating a previous set of estimates, describe the reasons for changes in estimates.	Discussion of results relevant to existing research provided in research in context, results, and discussion.
18. Discuss limitations of the estimates. Include a discussion of any modelling assumptions or data limitations that affect interpretation of the estimates.	Limitations of data and models provided in discussion.

Webappendix 2: Data sources

Table 2: Studies contributing to estimates of pathogen-specific meningitis case fatality ratio estimates in India stratified by child mortality setting

a. Streptococcus pneumoniae

Low child mortality settings (<30 deaths per 1000 live births)

- Al Awaidy, et al. "Epidemiology of invasive pneumococcal infection among hospitalized children aged less than 5 years in Oman." *Vaccine* 30 (2012): G7-G10.
- Al Ayed, et al. "Retrospective review of invasive pediatric pneumococcal diseases in a military hospital in the southern region of Saudi Arabia." *Annals of Saudi Medicine* 31.5 (2011): 469.
- Al Musawi, M., et al. "A retrospective epidemiological study of invasive pneumococcal infections in children aged 0-5 years in Bahrain from 1 January 1999 to 31 December 2003." *Vaccine*. 2012. 30 Suppl 6:G2-6.
- Andrade, A. L., et al. "Population-based surveillance for invasive pneumococcal disease and pneumonia in infants and young children in Goiania, Brazil." *Vaccine*. 2012. 30:1901-9
- Batuwanthudawe, R., et al. "Surveillance of invasive pneumococcal disease in Colombo, Sri Lanka." *Clinical Infectious Diseases* 48.Supplement 2 (2009): S136-S140.
- Benavides, J. A., et al. "Population-based surveillance for invasive pneumococcal disease and pneumonia in infants and young children in Bogota, Colombia." *Vaccine*. 2012. 30:5886-92
- Biaukula, V. L., et al. "Meningitis in children in Fiji: etiology, epidemiology, and neurological sequelae." *International Journal of Infectious Diseases* 16.4 (2012): e289-e295.
- Camou, T., et al. "Invasive pneumococcal diseases in Uruguayan children: comparison between serotype distribution and conjugate vaccine formulations." *Vaccine* 21.17 (2003): 2093-2096.
- Chan, Iris Mei-Ching, et al. "Invasive pneumococcal disease in Hong Kong children." J *Pediatr Respirology Critic Care* 9.2 (2013): 4-8.
- Chavez, P., et al. "Meningitis bacteriana aguda: experiencia de 10 años." *Rev Chil Infectol*;11(2):92-98, 1994
- Davidson, M., et al. "The Epidemiology of Invasive Pneumococcal Disease in Alaska, 1986-1990 Ethnic Differences and Opportunities for Prevention." *Journal of Infectious Diseases* 170.2 (1994): 368-376.
- Dickinson M., et al., "Las meningoencefalitis bacterianas en la población infantil cubana: 1998-2000." *Revista Cubana de Pediatría* 74.2 (2002): 106-114.
- Fortnum, H. M. and A. C. Davis. "Epidemiology of bacterial meningitis." *Archives of Disease in Childhood* 68.6 (1993): 763-767.
- Grzesiowski, P., et al. "Invasive pneumococcal disease in children up to 5 years of age in Poland." *Eur J Clin Microbiol Infect Dis.* 2008. 27:883-5
- Hanna J. N., et al. "Bacterial meningitis in children under five years of age in Western Australia. *Medical Journal of Australia*." 1991.;155:3,160-164
- Harboe, Z. B., et al. "Pneumococcal serotypes and mortality following invasive pneumococcal disease: a population-based cohort study." *PLoS Med* 6.5 (2009): e1000081.
- Ishikawa, T., et al. "Epidemiology of bacterial meningitis in children: Aichi prefecture, Japan, 1984-1993." *Pediatric Neurology*. 1996 Apr;14(3):244-250
- Jansen, A. G., et al. " Invasive pneumococcal disease in the Netherlands: Syndromes, outcome and potential vaccine benefits." *Vaccine*. 2009. 27:2394-401
- Johnson, A. P., et al. "Morbidity and mortality of pneumococcal meningitis and serotypes of causative strains prior to introduction of the 7-valent conjugant pneumococcal vaccine in England." *Journal of Infection* 55.5 (2007): 394-399.
- Lagos, R., et al., "Age- and serotype-specific pediatric invasive pneumococcal disease: insights from systematic surveillance in Santiago, Chile, 1994--2007." *J Infect Dis.* 2008. 198:1809-17
- Lagos, Z. R., et al. "Epidemiología de las enfermedades invasoras causadas por *Streptococcus pneumoniae* en niños chilenos: proyecciones clínicas y de salud pública." *Revista chilena de infectología* 18 (2001): 15-21.
- Lim, L. H., et al. "Childhood invasive pneumococcal disease: a hospital-based study from Malaysia." *J Paediatr Child Health*. 2007. 43:366-9
- Lucey, J. M., et al. "Pneumococcal meningitis: clinical outcomes in a pre-vaccine era at a Dublin paediatric hospital, 1999–2007." *Irish Journal of Medical Science* 180.1 (2011): 47-50.
- McIntyre, P. B., et al., "A population based study of the impact of corticosteroid therapy and delayed diagnosis on the outcome of childhood pneumococcal meningitis." *Archives of Disease in Childhood*. 2005. 90:391-396
- Memish, Z. A., et al., "Epidemiology of invasive pneumococcal infection in children aged five years and under in Saudi Arabia: a five-year retrospective surveillance study." *Int J Infect Dis*. 2010. 14:e708-12
- Miller, E., et al. "Epidemiology of invasive and other pneumococcal disease in children in England and Wales 1996–1998." *Acta Paediatrica* 89.s435 (2000): 11-16.
- Namani, S. R. K., et al. "Causative pathogens of bacterial meningitis in children and their susceptibility to antibiotics." *The Internet JInfects Dis* 9 (2010): 1-6.
- Rendi-Wagner, P., et al. "Prospective surveillance of incidence, serotypes and antimicrobial susceptibility of invasive *Streptococcus pneumoniae* among hospitalized children in Austria." *Journal of Antimicrobial Chemotherapy*. 2004;53(5):826-831
- Ríos, A. M., et al. "Impacto de la resistencia a antimicrobianos y de serotipos de *Streptococcus pneumoniae* en la mortalidad de niños menores de 5 años con enfermedad invasora." (1999).
- Robinson, K. A., et al. "Epidemiology of invasive *Streptococcus pneumoniae* infections in the United States, 1995-1998: Opportunities for prevention in the conjugate vaccine era." *Jama* 285.13 (2001): 1729-1735.
- Trotman, H., et al. "Pneumococcal meningitis in Jamaican children." *West Indian Medical Journal* 58.6 (2009): 585-588.

- Von Kries, R., et al. "Prediction of the potential benefit of different pneumococcal conjugate vaccines on invasive pneumococcal disease in German children." *Pediatric Infectious Disease Journal* 21.11 (2002): 1017-1023.
- Voss, L., et al. "Invasive pneumococcal disease in a pediatric population, Auckland, New Zealand." *Pediatric Infectious Disease Journal*. 1994;13(10):873-878

Medium child mortality settings (30-<75 deaths per 1000 live births)

- Abucejo-Ladesma, E., et al. "Serious community-acquired paediatric infections in rural Asia (Bohol Island, Philippines): bacterial meningitis in children less than 5 years of age." *Scandinavian Journal of Infectious Diseases* 39.11-12 (2007): 983-989.
- Asturias, E. J., et al. "Meningitis and pneumonia in Guatemalan children: the importance of *Haemophilus influenzae* type b and *Streptococcus pneumoniae*." *Revista Panamericana de Salud Pública* 14.6 (2003): 377-384.
- Berkley, J. A., et al. "Bacteremia among children admitted to a rural hospital in Kenya." New England Journal of Medicine 352.1 (2005): 39-47.
- Capeding, Maria Rosario, et al. "Prospective surveillance study of invasive pneumococcal disease among urban children in the Philippines." Pediatric Infectious Disease Journal 32.10 (2013): e383-e389.
- Chhetri, U. D., et al. "Clinical profile of invasive pneumococcal disease in Patan Hospital, Nepal." *Kathmandu University Medical Journa*l 9.1 (2012): 45-49.
- El Mdaghri, N., et al. "Epidemiological profile of invasive bacterial diseases in children in Casablanca, Morocco: antimicrobial susceptibilities and serotype distribution." *Eastern Mediterranean Health Journal* 18.11 (2012): 1097.
- Lovera, D., et al., "Risk factors for mortality in Paraguayan children with pneumococcal bacterial meningitis." *Tropical Medicine & International Health* 10.12 (2005): 1235-1241.
- Reis, J. N., et al. "Population-based survey of antimicrobial susceptibility and serotype distribution of Streptococcus pneumoniae from meningitis patients in Salvador, Brazil." *Journal of Clinical Microbiology* 40.1 (2002): 275-277.
- Shah, A. S., et al. "Invasive pneumococcal disease in Kanti Children's Hospital, Nepal, as observed by the South Asian Pneumococcal Alliance network." *Clinical Infectious Diseases* 48.Supplement 2 (2009): S123-S128.
- Williams, E. J., et al. "Hospital-based surveillance of invasive pneumococcal disease among young children in urban Nepal." *Clinical Infectious Diseases* 48. Supplement 2 (2009): S114-S122.

High child mortality settings (75-<150 deaths per 1000 live births)

- Al Khorasani, A., et al., "Bacterial profile and clinical outcome of childhood meningitis in rural Yemen: a 2-year hospital-based study." *Journal of Infection* 53.4 (2006): 228-234.
- Ba, O., et al. "Hospital surveillance of childhood bacterial meningitis in Senegal and the introduction *of Haemophilus influenzae* type b conjugate vaccine." *American Journal of Tropical Medicine and Hygiene* 83.6 (2010): 1330-1335.
- Iriso, R., et al. "Bacterial meningitis following introduction of Hib conjugate vaccine in northern Uganda." *Annals of Tropical Paediatrics* 28.3 (2008): 211-216.
- Khowaja, A. R., et al. "Mortality and neurodevelopmental outcomes of acute bacterial meningitis in children aged< 5 years in Pakistan." The Journal of Pediatrics 163.1 (2013): S86-S91.
- Kisakye, A., et al. "Surveillance for *Streptococcus pneumoniae* meningitis in children aged< 5 years: implications for immunization in Uganda." *Clinical Infectious Diseases* 48.Supplement 2 (2009): S153-S161.
- Menezes, Ana Paula de O., et al. "Serotype distribution and antimicrobial resistance of *Streptococcus pneumoniae* prior to introduction of the 10-valent pneumococcal conjugate vaccine in Brazil, 2000–2007." Vaccine 29.6 (2011): 1139-1144.
- Zaidi, A. K. M., et al. "Surveillance of pneumococcal meningitis among children in Sindh, southern Pakistan." *Clinical Infectious Diseases* 48.Supplement 2 (2009): S129-S135.

Very high child mortality settings (>=150 deaths per 1000 live births)

- Bercion, R., et al. "Acute bacterial meningitis at the ‘Complexe Pédiatrique’of Bangui, Central African Republic." *Journal of Tropical Pediatrics* 54.2 (2008): 125-128.
- Bernard-Bonnin, A. C., et al. "Purulent meningitis in children in Yaounde: epidemiological and prognostic aspects." *Annales de la Societe Belge de Medecine Tropicale*. Vol. 65. No. 1. 1984.
- Falade, A. G., et al. "Invasive pneumococcal disease in children aged< 5 years admitted to 3 urban hospitals in Ibadan, Nigeria." *Clinical Infectious Diseases* 48.Supplement 2 (2009): S190-S196.
- Tall, F. R., et al. "*Haemophilus influenzae* meningitis in a hospital in Burkina-Faso." *Medecine et Maladies Infectieuses*. 1992;22(12):1173-1177
- Yaro, S., et al. "The epidemiology of Haemophilus influenzae type b meningitis in Burkina Faso." *Pediatric Infectious Disease Journal* 25.5 (2006): 415-419.

b. *Haemophilus influenzae* type b (Hib)

Low child mortality settings (<30 deaths per 1000 live births)

- Almuneef, M., et al. "Childhood bacterial meningitis in Saudi Arabia." *Journal of Infection* 36.2 (1998): 157-160.
- Anh, Dang D., et al. "*Haemophilus influenzae* type B meningitis among children in Hanoi, Vietnam: epidemiologic patterns and estimates of H. Influenzae type B disease burden." *American Journal of Tropical Medicine and Hygiene* 74.3 (2006): 509-515.
- Anh, D. D., et al. "Surveillance of pneumococcal-associated disease among hospitalized children in Khanh Hoa Province, Vietnam." *Clinical Infectious Diseases* 48.Supplement 2 (2009): S57-S64.
- Barton-Forbes, M. A., et al., "Epidemiology of *Haemophilus influenzae* invasive disease in Jamaica, 1990-1993." *West Indian Medical Journal* 49.3 (2000): 200-204.
- Batuwanthudawe, R., et al. "Surveillance of invasive pneumococcal disease in Colombo, Sri Lanka." *Clinical Infectious Diseases* 48.Suppl. 2 (2009): S136-S140.

- Batuwanthudawe, R., et al. "Incidence of childhood *Haemophilus influenzae* type b meningitis in Sri Lanka." *International Journal of Infectious Diseases* 14.5 (2010): e372-e376.
- Boehme, C., et al. "Three years of acute bacterial meningitis in the pediatric service at the Temuco Regional Hospital." *Revista Medica de* Chile 121.6 (1993): 633-638.
- Booy, R., et al. "Invasive *Haemophilus influenzae* type b disease in the Oxford region (1985-91)." *Archives of Disease in Childhood* 69.2 (1993): 225-228.
- Bower, C., et al. "Sequelae of *Haemophilus influenzae* type b meningitis in Aboriginal and non-Aboriginal children under 5 years of age." *Journal of Paediatrics and Child Health* 30.5 (1994): 393-397.
- Chavez, P., et al. "Meningitis bacteriana aguda: experiencia de 10 años." *Rev. Chil. Infectol* 11.2 (1994): 92-8.
- Chen, M. K., et al. "Prospective surveillance of children with invasive *Haemophilus influenzae* disease in Taiwan." *Journal of Microbiology, Immunology, and Infection* 32.4 (1999): 257-260.
- Dagan, R., et al. "Epidemiology of pediatric meningitis caused by *Haemophilus influenzae* type b, *Streptococcus pneumoniae*, and Neisseria meningitidis in Israel: a 3-year nationwide prospective study." *Journal of Infectious Diseases* 169.4 (1994): 912-916.
- De Jonghe, M. and G. Glaesener. "Type B *Haemophilus influenzae* infections. Experience at the pediatric hospital of Luxembourg." *Bulletin de la Société des Sciences Médicales du Grand-Duché de Luxembourg* 132.2 (1995): 17.
- Dickinson, M., et al., "Las meningoencefalitis bacterianas en la población infantil cubana: 1998-2000." *Revista Cubana de Pediatría* 74.2 (2002): 106-114.
- Fortnum, H. M. and A. C. Davis. "Epidemiology of bacterial meningitis." Archives of disease in childhood 68.6 (1993): 763-767.
- Gilbert, G. L., et al., "Clinical manifestations and outcome of *Haemophilus influenzae* type b disease." *Journal of Paediatrics and Child Health* 31.2 (1995): 99-104.
- Halfon-yaniv, I., et al., "Epidemiology of invasive *Haemophilus influenzae* type b infections in Bedouins and Jews in Southern Israel." *Pediatric Infectious Disease Journal* 9.5 (1990): 321-325.
- Ishikawa, T., et al. "Epidemiology of bacterial meningitis in children: Aichi Prefecture, Japan, 1984–1993." *Pediatric Neurology* 14.3 (1996): 244-250.
- Kamiya, Hitoshi, et al. "Childhood bacterial meningitis in Japan." *Pediatric Infectious Disease Journal* 17.9 (1998): S183-S185.
- Kristensen, K., et al. "Epidemiology of *Haemophilus influenzae* type b infections among children in Denmark in 1985 and 1986." *Acta Paediatrica* 79.6-7 (1990): 587-592.
- McIntyre, P. B., et al. "Invasive *Haemophilus influenzae* type b disease in Sydney children 1985-1987: a population-based study." *Medical Journal of Australia* 154.12 (1991): 832-837.
- McIntyre, P., et al. "The outcome of childhood *Haemophilus influenzae* meningitis. A population based study." *Medical Journal of Australia* 159.11-12 (1992): 766-772.
- Muangchana, C., et al. "Bacterial meningitis incidence in Thai children estimated by a rapid assessment tool." *Southeast Asian Journal of Tropical Medicine and Public Health* 40.3 (2009): 553.
- Namani, S., et al. "Causative pathogens of bacterial meningitis in children and their susceptibility to antibiotics." *Journal of Infectious Disease* 9 (2010): 1-6.
- Rauter, L., and I. Mutz. "*Haemophilus influenzae* meningitis 1983 to 1992--epidemiology and sequelae of the disease." *Wiener Klinische Wochenschrift* 106.7 (1993): 187-192.
- Reinert, P., et al. "Epidemiology of *Haemophilus influenzae* type b disease in France." *Vaccine* 11 (1993): S38-S42.
- Schoendorf, K. C., et al. "National trends in *Haemophilus influenzae* meningitis mortality and hospitalization among children, 1980 through 1991." *Pediatrics* 93.4 (1994): 663-668.
- Zaki, M., et al. "Childhood bacterial meningitis in Kuwait." *Journal of Tropical Medicine and Hygiene* 93.1 (1990): 7-11.

Medium child mortality settings (30-<75 deaths per 1000 live births)

- Abucejo-Ladesma, E., et al. "Serious community-acquired paediatric infections in rural Asia (Bohol Island, Philippines): bacterial meningitis in children less than 5 years of age." *Scandinavian Journal of Infectious Diseases* 39.11-12 (2007): 983-989.
- Ansari, I., et al. "Culture proven bacterial meningitis in children: agents, clinical profile and outcome." *Kathmandu Univ Med J.* 2011. 9:36-40
- Asturias, Edwin J., et al. "Meningitis and pneumonia in Guatemalan children: the importance of *Haemophilus influenzae* type b and *Streptococcus pneumoniae*." *Revista Panamericana de Salud Pública* 14.6 (2003): 377-384.
- Basualdo, W., et al. "Invasive *Haemophilus influenzae* type b infections in children in Paraguay." *Archives of Medical Research* 35.2 (2004): 126-133.
- El Mdaghri, N., et al. "Epidemiological profile of invasive bacterial diseases in children in Casablanca, Morocco: antimicrobial susceptibilities and serotype distribution." *Eastern Mediterranean Health Journal* 18.11 (2012): 1097.
- Hussey, G., et al. "Epidemiology of invasive *Haemophilus influenzae* infections in Cape Town, South Africa." *Annals of Tropical Paediatrics* 14.2 (1994): 97-103.
- Limcangco, M. R. T., et al. "Epidemiology of *Haemophilus influenzae* type b meningitis in Manila, Philippines, 1994 to 1996." *Pediatric Infectious Disease Journal* 19.1 (2000): 7-11.
- Luby, S. P., et al. "A low-cost approach to measure the burden of vaccine preventable diseases in urban areas." *Vaccine* 28.31 (2010): 4903-4912.
- Mendsaikhan, Jamsran, et al. "Childhood bacterial meningitis in Ulaanbaatar, Mongolia, 2002–2004." *Clinical Infectious Diseases* 48.Supplement 2 (2009): S141-S146.
- Minz, S., et al. "Incidence of *Haemophilus influenzae* type b meningitis in India." *Indian Journal of Medical Research* 128.1 (2008): 57.

- Ribeiro, G. S., et al. "Prevention of *Haemophilus influenzae* type b (Hib) meningitis and emergence of serotype replacement with type a strains after introduction of Hib immunization in Brazil." *Journal of Infectious Diseases* 187.1 (2003): 109-116.
- Saha, S. K., et al. "The increasing burden of disease in Bangladeshi children due to *Haemophilus influenzae* type b meningitis." *Annals of Tropical Paediatrics* 17.1 (1997): 5-8.
- Shah, A. S., et al. "Invasive pneumococcal disease in Kanti Children's Hospital, Nepal, as observed by the South Asian Pneumococcal Alliance network." *Clinical Infectious Diseases* 48.Supplement 2 (2009): S123-S128.
- Simões, L. L. P., et al. "Impact of *Haemophilus influenzae* b (Hib) vaccination on meningitis in Central Brazil." *Revista de Saude Publica* 38.5 (2004): 664-670.
- Torres, A., et al. "Infecciones invasivas por *Haemophilus influenzae* tipo b (Hib) en Tucumán-Argentina." *Arch. Argent. Pediatr* 93.4 (1995): 238-44.
- Weiss, D. P.L., et al. "Epidemiology of bacterial meningitis among children in Brazil, 1997-1998." *Revista de Saude Publica* 35.3 (2001): 249-255.

High child mortality settings (75-<150 deaths per 1000 live births)

- Adegbola, R. A., et al. "*Haemophilus influenzae* type b disease in the western region of The Gambia: background surveillance for a vaccine efficacy trial." *Annals of Tropical Paediatrics* 16.2 (1996): 103-111.
- Invasive Bacterial Infections Surveillance (IBIS) Group of the International Clinical Epidemiology Network. "Are *Haemophilus influenzae* infections a significant problem in India? A prospective study and review." *Clinical Infectious Diseases* 34.7 (2002): 949-957.
- Khowaja, A. R., et al. "Mortality and neurodevelopmental outcomes of acute bacterial meningitis in children aged< 5 years in Pakistan." *Journal of Pediatrics* 163.1 (2013): S86-S91.
- Koko, J., et al. "Bacterial meningitis in children in Libreville, Gabon. Epidemiologic, therapeutic and prognostic features." *Medecine et Maladies Infectieuses* 30.1 (2000): 50-56.
- Lewis, R. F., et al. "Action for child survival: elimination of *Haemophilus influenzae* type b meningitis in Uganda." *Bulletin of the World Health Organization* 86.4 (2008): 292-301.
- Zaidi, A. K. M., et al. "Surveillance of pneumococcal meningitis among children in Sindh, southern Pakistan." *Clinical Infectious Diseases* 48.Supplement 2 (2009): S129-S135.

Very high child mortality settings (>=150 deaths per 1000 live births)

- Bercion, R., et al. "Acute bacterial meningitis at the 'Complexe Pédiatrique'of Bangui, Central African Republic." *Journal of Tropical Pediatrics* 54.2 (2008): 125-128.
- Bernard-Bonnin, A. C., et al. "Purulent meningitis in children in Yaounde: epidemiological and prognostic aspects." *Annales de la Societe Belge de Medecine Tropicale*. Vol. 65. No. 1. 1984.
- Bijlmer, H. A., et al. "The epidemiology of *Haemophilus influenzae* meningitis in children under five years of age in The Gambia, West Africa." *Journal of Infectious Diseases* 161.6 (1990): 1210-1215.
- Campagne, G., et al. "Epidemiology of bacterial meningitis in Niamey, Niger, 1981-96." *Bulletin of the World Health Organization* 77.6 (1999): 499.
- Lagunju, I. A., et al. "Childhood bacterial meningitis in Ibadan, Nigeria--antibiotic sensitivity pattern of pathogens, prognostic indices and outcome." *African Journal of Medicine and Medical sciences* 37.2 (2008): 185-191.
- Molyneux, E., et al. "Acute bacterial meningitis in children admitted to the Queen Elizabeth Central Hospital, Blantyre, Malawi in 1996–97." *Tropical Medicine & International Health* 3.8 (1998): 610-618.
- Muhe, L., et al. "Pneumococcal and *Haemophilus influenzae* meningitis in a children's hospital in Ethiopia: serotypes and susceptibility patterns." *Tropical Medicine & International Health* 4.6 (1999): 421-427.
- Tall, F. R., et al. "*Haemophilus influenzae* meningitis in a hospital in Burkina-Faso." *Medecine et Maladies Infectieuses*. 1992;22(12):1173-1177
- Yaro, S., et al. "The epidemiology of Haemophilus influenzae type b meningitis in Burkina Faso." *Pediatric Infectious Disease Journal* 25.5 (2006): 415-419.

Table 3: Studies contributing to the distribution of meningitis cases in India

Distribution of meningitis cases by etiology
**Asia**

- Abanamy, A., et al. "Childhood bacterial meningitis in Riyadh." *Annals of Saudi Medicine* 11.6 (1991): 628-632.
- Abomelha A. et al., "Childhood bacterial meningitis"
- Al Khorasani, A., et al., "Bacterial profile and clinical outcome of childhood meningitis in rural Yemen: a 2-year hospital-based study." *Journal of Infection* 53.4 (2006): 228-234.
- al-Jurayyan N. A., et al., "Childhood bacterial meningitis in Al-Baha province, Saudi Arabia"
- Al-Mazrou, Y. Y., et al. "*Haemophilus type* B meningitis in Saudi children under 5 years old." *Journal of Tropical Pediatrics* 50.3 (2004): 131-136.
- Almuneef, M., et al. "Childhood bacterial meningitis in Saudi Arabia." *Journal of Infection* 36.2 (1998): 157-160.
- Ansari, I., et al. "Culture proven bacterial meningitis in children: agents, clinical profile and outcome." *Kathmandu University Medical Journal* 9.1 (2012): 36-40.
- Azubuike J. C., et al., "Childhood bacterial meningitis in Tabuk, Saudi Arabia"
- Batuwanthudawe, R., et al. "Incidence of childhood *Haemophilus influenzae* type b meningitis in Sri Lanka." *International Journal of Infectious Diseases* 14.5 (2010): e372-e376.
- Batuwanthudawe, R., et al. "Surveillance of invasive pneumococcal disease in Colombo, Sri Lanka." *Clinical Infectious Diseases* 48.Supplement 2 (2009): S136-S140.
- Ceyhan, M., "A Prospective Study of Etiology of Childhood Acute Bacterial Meningitis, Turkey-Volume 14, Number 7—July 2008-Emerging Infectious Disease journal-CDC." (2008).
- Chinchankar, Nandita, et al. "Diagnosis and outcome of acute bacterial meningitis in early childhood." Indian pediatrics 39.10 (2002): 914-921.
- Choo, K. E., et al. "Pyogenic meningitis in hospitalized children in Kelantan, Malaysia." *Annals of Tropical Paediatrics* 10.1 (1990): 89-98.
- Dagan, R., et al. "Effectiveness of a nationwide infant immunization program against *Haemophilus influenzae* b." *Vaccine* 17.2 (1999): 134-141.
- Fujii, R., et al. "Trends in childhood bacterial meningitis in Japan (1979-1984).(Part 1). On the causative organisms." Kansenshōgaku zasshi. *Journal of the Japanese Association for Infectious Diseases* 60.6 (1986): 592.
- Gratten, M., et al. "The Bacteriology of Acute Pneumonia and Meningitis in Children in Papua New Guinea: Assumptions, Facts and Technical Strategies." *Papua New Guinea Medical Journal* 48.1/2 (2005): 73.
- Gurley, E. S., et al. "Etiologies of bacterial meningitis in Bangladesh: results from a hospital-based study." *American Journal of Tropical Medicine and Hygiene* 81.3 (2009): 475-483.
- Kim, Kyung-Hyo, et al. "The Causative Organisms of Bacterial Meningitis in Korean Children." *J Korean Med Sci* 13 (1998): 60-4.
- Kim, S. A., et al. "An expanded age range for meningococcal meningitis: molecular diagnostic evidence from population-based surveillance in Asia." *BMC Infectious Diseases* 12.1 (2012): 310.
- Lehmann, D., et al. "Aetiology and clinical signs of bacterial meningitis in children admitted to Goroka Base Hospital, Papua New Guinea, 1989–1992." *Annals of Tropical Paediatrics* 19.1 (1999): 21-32.
- Liu, C. C., et al. "Bacterial meningitis in infants and children in southern Taiwan: emphasis on *Haemophilus influenzae* type B infection." *Journal of the Formosan Medical Association Taiwan* 92.10 (1993): 884-888.
- Mahmood Y. A., et al., "Neonatal and childhood bacterial meningitis at Saddam general hospital in Tikrit." *Medical Journal of Tikrit* University (1997);3:39-45
- Mahmoud, R., et al. "Pattern of meningitis in Al-Ain medical district, United Arab Emirates—a decadal experience (1990–99)." *Journal of Infection* 44.1 (2002): 22-25.
- Mani, R., et al. "Bacteriological profile of community acquired acute bacterial meningitis: a ten-year retrospective study in a tertiary neurocare centre in South India." *Indian Journal of Medical Microbiology* 25.2 (2007): 108.
- Mao, Fei-Fei, et al. "Aetiological spectrum and antibiotic susceptibility pattern of bacterial meningitis in infants and children in Hangzhou, China." *Acta Paediatrica* 94.8 (2005): 1162-1163.
- Mendsaikhan, Jamsran, et al. "Childhood bacterial meningitis in Ulaanbaatar, Mongolia, 2002–2004." *Clinical Infectious Diseases* 48.Supplement 2 (2009): S141-S146.
- Moïsi, J. C., et al. "Enhanced diagnosis of pneumococcal meningitis with use of the Binax NOW immunochromatographic test of *Streptococcus pneumoniae* antigen: a multisite study." *Clinical Infectious Diseases* 48.Supplement 2 (2009): S49-S56.
- Saha, S. K., et al. "The increasing burden of disease in Bangladeshi children due to *Haemophilus influenzae* type b meningitis." *Annals of Tropical Paediatrics* 17.1 (1997): 5-8.
- Sakata, H., et al. "A study of bacterial meningitis in Hokkaido between 1994 and 1998." Kansenshogaku zasshi. *The Journal of the Japanese Association for Infectious Diseases* 74.4 (2000): 339-344.
- Sung, R. Y. T., et al. "Meningitis in Hong Kong children, with special reference to the infrequency of haemophilus and meningococcal infection." *Journal of Paediatrics and Child Health* 33.4 (1997): 296-299.
- Sutinen, J., et al. "Etiology of central nervous system infections in the Philippines and the role of serum C-reactive protein in excluding acute bacterial meningitis." *International Journal of Infectious Diseases* 3.2 (1999): 88-93.
- Yang, Y., et al. "Acute bacterial meningitis in children in Hefei, China 1990-1992." *Chinese Medical Journal* 109.5 (1996): 385-388.
- Zaidi, Anita KM, et al. "Surveillance of pneumococcal meningitis among children in Sindh, southern Pakistan." *Clinical Infectious Diseases* 48.Supplement 2 (2009): S129-S135.

Webappendix 3: Methods for estimating state-level, all-cause clinical and severe pneumonia cases

We used methods that have been previously described elsewhere to estimate the incidence of community-based pneumonia and severe pneumonia in low- and middle-income countries.[6] We treated states in India as countries in this model. The detailed methods for all developing countries, including subnational estimates in India, are described below.

Step 1
A systematic literature review was conducted to identify published data on the incidence of pneumonia from community-based longitudinal studies. From the literature, an estimate of the proportion of pneumonia cases that are severe was also estimated. Only studies that met the minimum quality criteria we included. These were: (1) community-based surveillance of a defined population of children had to have been carried out for a minimum period of one year and in multiples of 12 months because clinical pneumonia has such a markedly seasonal nature; (2) the study had to use a strategy of active case detection—the surveillance interval had to be two weeks or shorter to minimize recall bias; and (3) case definitions had to be clearly defined and consistently applied.

Step 2
We conducted a systematic literature review to identify the risk factors for pneumonia in children aged 0-4 years. We selected risk factors which in meta- analyses had strong statistically significant associations with pneumonia. These were:

- Malnutrition (weight for age <-2SD): odds ratio (OR) 4.5 (2.1-9.5)
- Low birth weight (<2500g): OR 3.6 (0.8-16.3)
- Non-exclusive breastfeeding (4 months): OR 2.7 (1.7-4.4)
- Indoor air pollution: OR 1.6 (1.1-2.3)
- HIV: OR 6.51 (5.86-7.24)
- Crowding (more than 5 persons per household): OR 1.9 (1.5-2.5)
- Incomplete immunisation (no measles immunisation at 12 months): OR 1.8 (1.3-2.5)

Step 3
We obtained data on the prevalence of all risk factors other than HIV from representative household surveys with large sample sizes—the demographic and health surveys (DHS). DHS surveys are generally carried out every 2 to 5 years and provide data on demography and health indicators. The state-level prevalence data (other than HIV) in each state were obtained from the National Family Health Survey (NFHS). The HIV data were obtained from the United Nations Programme on HIV/AIDS (UNAIDS) estimates for 2013. For states in India, the prevalence of HIV in children was estimated using the proportion of children in each state with mothers infected with HIV and the UNAIDS estimate of the odds ratio for HIV infection in children born to mothers infected with HIV. We ensured that the definitions of risk factors were the same in the studies estimating the risk ratios as in the surveys that measured prevalence of exposure to these risk factors.

Specifically, we defined each risk factor using one or more DHS/NFHS variables. Then, using individual patient data from the standard DHS/NFHS survey (excluding special surveys) for each country or state at the survey version closest to 2000 and 2015 respectively, we calculated the proportion of participants with each combination of risk factors.

Step 4
Apart from HIV, all variables were contained within the DHS survey for India, and so the joint distributions were observed. For HIV, we corrected the DHS-derived estimates of HIV to match the UNAIDS survey estimates for India. First, we calculated the odds ratio for the difference in maternal HIV for each child to the childhood HIV prevalence estimates at the whole-India level. Next, we applied this odds ratio to the state-level data to estimate the prevalence with HIV for each combination of the other risk factors.

Step 5
The overall incidence (of clinical pneumonia in community) and hospitalization rate (for physician diagnosed pneumonia) for developing countries and Indian states were obtained from the Poisson regression models as described elsewhere.[6]

10,000 samples for each prediction were obtained. For the proportion of cases which were severe (i.e. with lower chest wall indrawing), the estimate from random effects meta-analysis (using Stata 11.1) was represented as a beta distribution, from which 10,000 samples were obtained using R. The incidence of severe pneumonia was calculated as the incidence of clinical pneumonia in the community times the proportion with severe pneumonia.

The overall incidence (of clinical and hospitalized pneumonia respectively) in a population is the sum of each stratum specific rate, weighted by the proportion in each stratum where there are j mutually exclusive strata:

$$Total\ rate = \frac{\sum x_j}{\sum n_j} = \sum_j \left(\frac{x_j}{n_j} \cdot \frac{n_0}{x_0} \cdot \frac{x_0}{n_0} \cdot \frac{n_j}{\sum n_j} \right)$$

where x is the number of cases in a stratum, n is the total population in a stratum and where $j = 0$ indicates the stratum which has no exposures (i.e., the unexposed) and $j = 1$ to $j = 127$ indicate the strata for each unique combination of risk factors. This equation can be re-written as:

$$Total\ rate = \frac{x_0}{n_0} \times \sum j \left(\frac{x_j}{n_j} \cdot \frac{n_0}{x_0} \cdot \frac{n_j}{\sum n_j} \right)$$

where $\frac{x_0}{n_0}$ is the rate in the unexposed, $\frac{x_j}{n_j} \cdot \frac{n_0}{x_0}$ is the rate ratio, and $\frac{n_j}{\sum n_j}$ is the proportion in each stratum.

Therefore, using the regional estimates (for developing countries or Indian states) of the total rate, the combined rate ratio for each stratum, and the proportion in each stratum, we can calculate the incidence in unexposed people for the region (all developing countries and Indian states). If we then assume that the rate in the unexposed is consistent across the region, we can then calculate the incidence rate for each country or Indian state:

$$Country\ or\ Indian\ state\ specific\ rate = \frac{\sum x_j^c}{\sum n_j^c} = \frac{\sum x_j^s}{\sum n_j^s} \cdot \frac{\sum_j (\frac{x_j}{n_j} \cdot \frac{n_0}{x_0} \cdot \frac{n_j^r}{\sum n_j^r})}{\sum_j (\frac{x_j}{n_j} \cdot \frac{n_0}{x_0} \cdot \frac{n_j^s}{\sum n_j^s})}$$

where c indicates a country- or Indian state-specific value and r indicates a regional value.

<u>Step 6</u>

Assumptions

- As with attributable fraction calculations, we assume that the pooled risk ratios obtained from a range of case-control/cohort studies in the developing region were applicable across each developing country or Indian state. As described in Step 7 we added additional uncertainty to our estimates to relax this assumption somewhat. We assumed that odds ratios were the same as relative risks.
- As in our previous analysis for HIV and pneumonia, we assumed that there was an *n*-way interaction between each risk factor (i.e., 1.25-fold reduction) such that rate ratios combined sub-multiplicatively (e.g., for the group with malnutrition and low birth weight, $RR_{malnutrition} = 4.5$, and $RR_{low\ birth\ weight} = 3.6$, $RR_{both} = (4.5 \times 3.6) / 1.25 = 12.96$ rather than 4.5 x 3.6 = 16.2; and for the group with malnutrition, low birth weight, and non-exclusive breastfeeding, $RR_{non\text{-}exclusive\ breast\ feeding} = 2.7$, $RR_{all\ three} = (4.5 \times 3.6 \times 2.7)/1.25^2 = 27.99$ rather than 4.5 x 3.6 x 2.7 = 43.74). None of the primary studies included in the meta-analysis reported interaction/heterogeneity of effect measure estimates on a multiplicative scale. However, although we noted that the majority of studies did not report testing for interaction (presumably because of an actual or perceived lack of statistical power) it seemed unlikely to us that all risks combined fully multiplicatively.

Therefore, we added a weak interaction term to shrink the combined rate ratios towards the null. This would have the effect of causing the country specific estimates to be attenuated towards the overall estimate for the developing region.

- In order to calculate the uncertainty for the proportion of the population in each country or Indian state within each of the j strata, we sampled from a Dirichlet distribution, a multivariate extension of the beta distribution. Using this approach, we did not need to assume independence of risk factors.
- Finally, we assumed that the rate among the unexposed (i.e., the risk in the absence of these seven risk factors) came from the same distribution for all countries and Indian states. This is the same as assuming that there was no residual confounding.

Step 7

Using a simulation-based approach, we performed the calculation described in Step 5 to estimate the country- and Indian state-specific rates along with uncertainty estimates as uncertainty ranges (UR). This was done by performing this calculation for each of the 10,000 samples from the distributions representing the incidence rates, disease severity proportion, odds ratios and risk factor proportions (methods used to obtain each of these were obtained as described above) within R. Full analysis code and data are available from the authors on request.

Step 7

In a similar manner to equation 3 within step 5, we calculated the incidence of pneumonia in HIV-uninfected children by summing those strata where children were HIV negative regardless of the presence/absence of other risk factors. Similarly, we calculated the incidence of pneumonia in HIV-infected children.

Step 8

Having obtained estimates of the total rate and rate in HIV-uninfected children in each country or Indian state, we used the standard formula for attributable fraction:

$$Attributable\ fraction = \frac{I_e - I_0}{I_e}$$

Or, equivalently:

$$Attributable\ fraction = \frac{RR\ -1}{RR}$$

where I_e is the incidence in HIV-infected and I_0 is the incidence in HIV-uninfected in each country or Indian state; and RR is the risk ratio for pneumonia in HIV-infected (compared to HIV-uninfected). We then applied the attributable fraction to the rate among the HIV-infected (and the UR) in order to obtain the rate attributable to HIV with the UR. As we had already incorporated uncertainty in the HIV risk factor estimates in calculating the total country rates, we did not add additional uncertainty at this stage.

Webappendix 4: Summary estimates for pathogen-specific pneumonia morbidity and mortality

4.1: Probe approach

We used the probe approach to estimate the proportion of pneumonia cases and deaths attributable to pneumococcus.[3 7] We first identified efficacy values from PCV and Hib vaccine randomized control trials (RCT) and effectiveness results from case-control studies with random or systematic allocation of vaccine. We used data from trials and studies that used case definitions that aligned closely with WHO pneumonia case definitions.[8-10] Since vaccine trials and studies did not assess efficacy against pneumonia mortality, we used values of efficacy and effectiveness against radiography-confirmed, primary end-point pneumonia (i.e., consolidation),[10] with relevant adjustments, as a proxy for the proportion of pneumonia deaths caused by each pathogen. Arithmetically inherent in using this proxy is that the pneumococcal pneumonia case fatality ratio (CFR) is equivalent to the Hib pneumonia CFR, and they are both equal to the CFR for non-pneumococcal, non-Hib pneumonia cases. The direction of the bias introduced by using this proxy has been described previously—it most likely underestimates pneumococcal and Hib deaths in high-mortality settings and may overestimate deaths in low-mortality settings.[3] We used values of PCV and Hib vaccine efficacy and effectiveness against WHO-defined clinical and severe pneumonia[8 9] to estimate the proportions of clinical and severe pneumonia cases associated with each pathogen.

4.2: Adjustments

PCV and Hib vaccine efficacy and effectiveness values were adjusted to account for imperfect efficacy against vaccine-type (VT) pneumococcal pneumonia. Since true efficacy values for these parameters are not known, we used as a proxy the observed efficacy against VT invasive pneumococcal disease (IPD) on a trial-by-trial basis. We estimated the degree of bias introduced by this proxy measure for pneumococcus using two sensitivity analyses described previously.[3] PCV efficacy values were also adjusted to account for the proportion of pneumonia that was VT in the control group. As this value is not available, we used as a proxy the proportion of IPD cases that were VT from the control group. For pneumococcus, PCV efficacy values were also adjusted to account for the proportion of pneumonia cases caused by Hib, since all PCV trials were conducted in the context of Hib vaccine use. Below are the formulas for calculating the proportion of deaths and cases due to pneumococcus and Hib:

$$\%\ Pneumonia\ cases_{Spn} = \frac{VE_{Pneumonia,PCV}}{[(VE_{IPD,PCV}) \times (VT_{Control}) \times (1 - \%\ Pneumonia\ cases_{Hib})]}$$

$$\%\ Pneumonia\ cases_{Hib} = \frac{VE_{Pneumonia,Hib}}{[VE_{IHD,Hib}]}$$

Where VE is PCV or Hib vaccine efficacy against a pneumonia or invasive disease, IPD is invasive pneumococcal disease, IHD is invasive Hib disease, $VT_{Control}$ is the proportion of pneumococcal disease caused by vaccine serotypes in the control group of the RCT.

Summary Estimates

Adjusted PCV and Hib vaccine efficacy and effectiveness estimates were each combined in a random effects meta-analysis to determine the pathogen-specific fraction of pneumonia cases for each pneumonia case definition (i.e., clinical, severe and radiography-confirmed pneumonia). We used the delta method to calculate standard errors for each trial. Random effects meta-analyses were used throughout the pathogen-specific pneumonia modeling because fixed effects models do not incorporate between study variability. In addition, fixed effects models produce biased results in most cases.[11] The value of random effects meta-analysis methods has been called into question. However, we determined that this was the most appropriate way to combine information from different studies into a single input parameter value with associated uncertainty.

Uncertainty

The primary sources of uncertainty in the pneumonia models were the between-study variation in vaccine efficacy estimates. For pneumococcal pneumonia, the Gambia trial[12] significantly pushes the meta-analysis upward. In contrast, the Lombok trial[13] pulls the overall estimate toward the null for Hib pneumonia. As we assume all Hib pneumonia deaths occur only in children less than two years, the upper limit uncertainty bounds, reflect the possibility that there may be deaths in older children. Because of this between-study variation, we chose to use a

jackknife, leave-one-study-out approach for the upper and lower bounds of the pneumococcal and Hib pneumonia fractions. A limitation of using the jackknife method with small sample sizes is that standard errors are not reliable, which is why we chose to use the upper and lower bounds for estimating uncertainty. The uncertainty bounds account for both sampling and non-sampling error. Assessing non-sampling error has been a limitation of disease burden models. This approach underscores the uncertainty arising from limited sample sizes, population variability, and the paucity of data that is usually available to estimate disease burden. We were unable to account these sources of uncertainty as these values (e.g., vaccine coverage, population estimates) were not available. Nevertheless, excluding all sources of model uncertainty leads to uncertainty ranges that are narrower than the true uncertainty.

Figure 4: Proportion of radiography-confirmed pneumonia attributable to pneumococcus

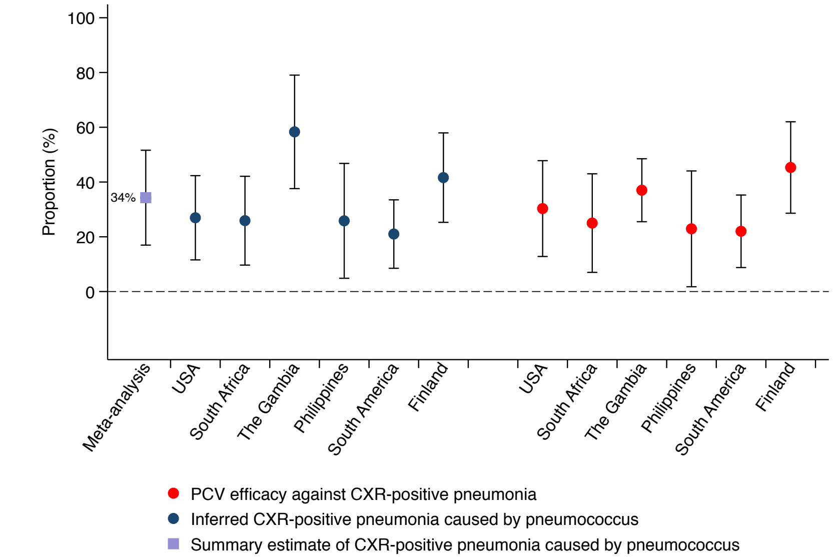

Figure 5: Proportion of severe pneumonia attributable to pneumococcus

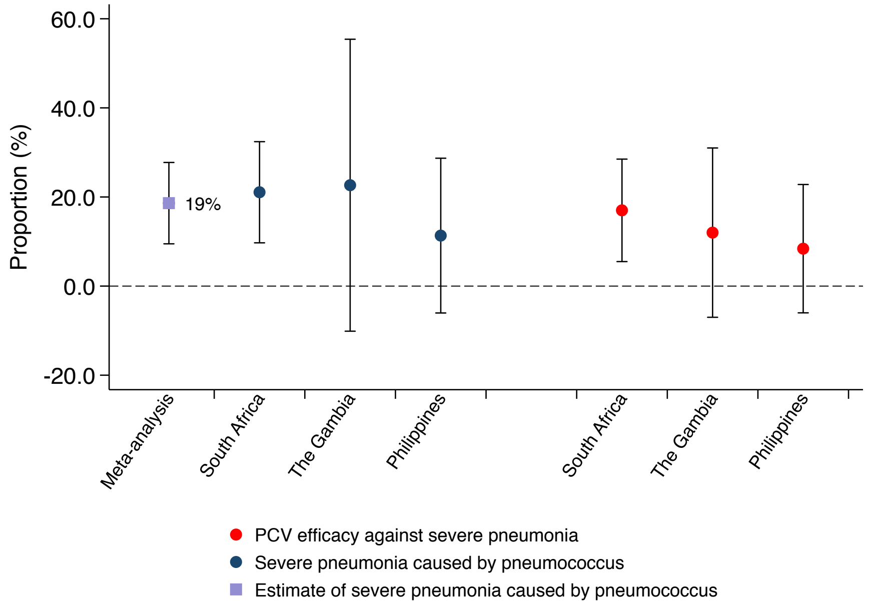

Figure 6: Proportion of clinical pneumonia attributable to pneumococcus

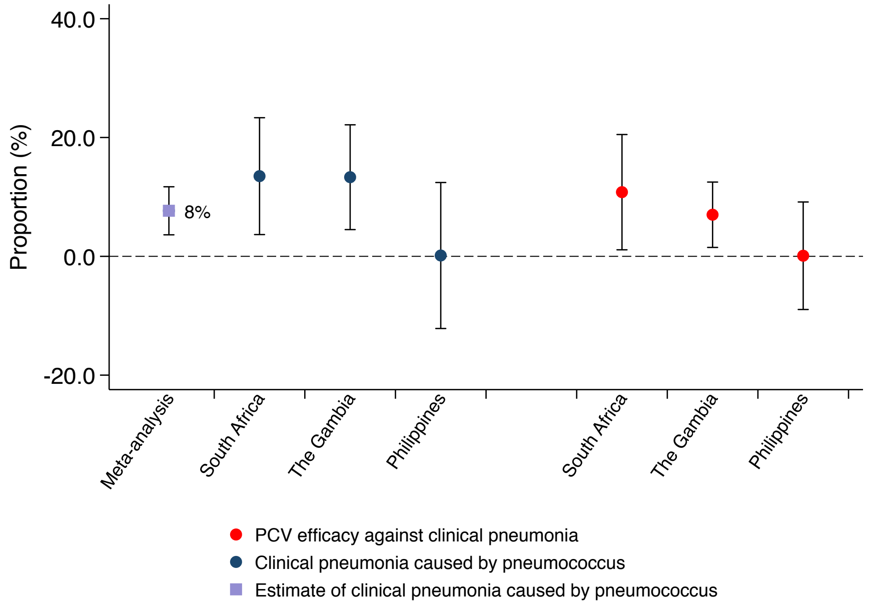

Figure 7: Proportion of radiography-confirmed pneumonia attributable to Hib

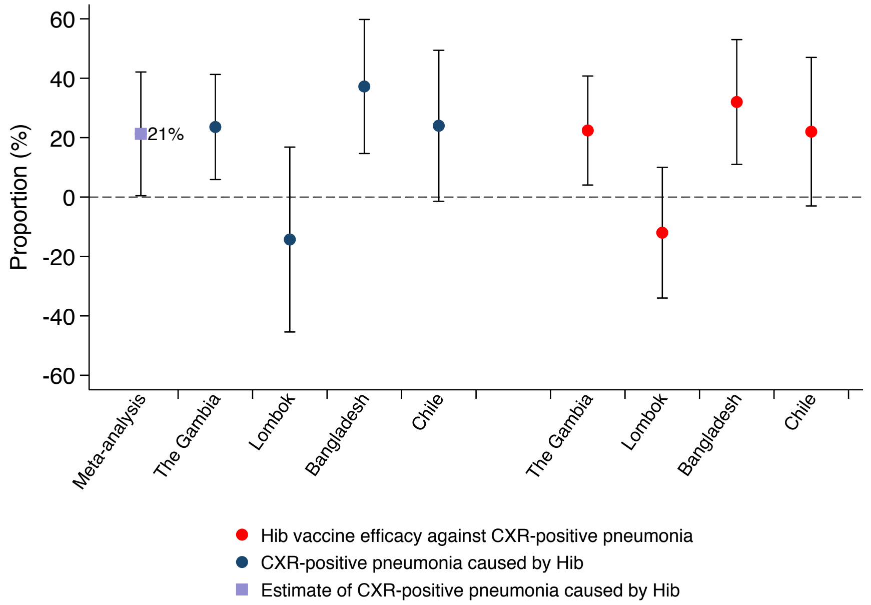

Figure 8: Proportion of severe pneumonia attributable to Hib

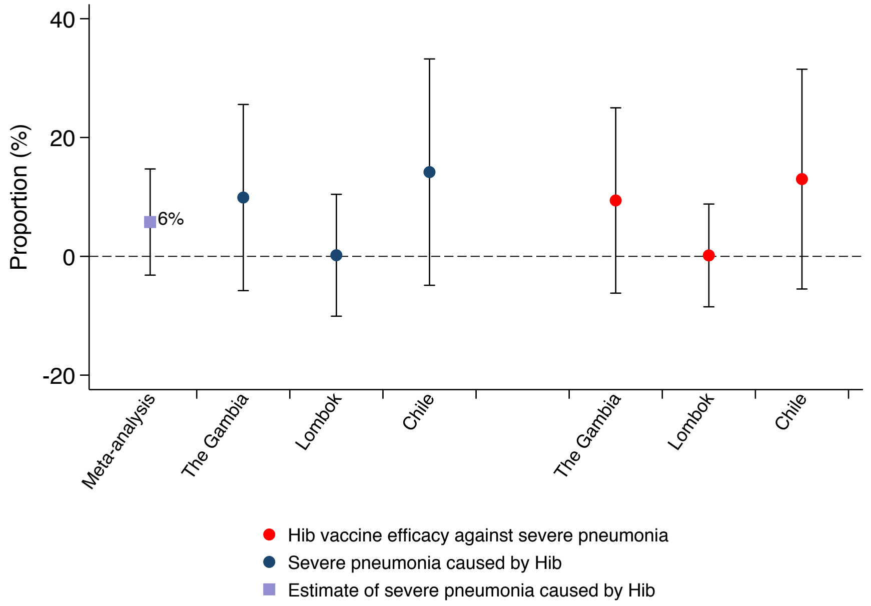

Figure 9: Proportion of clinical pneumonia attributable to Hib

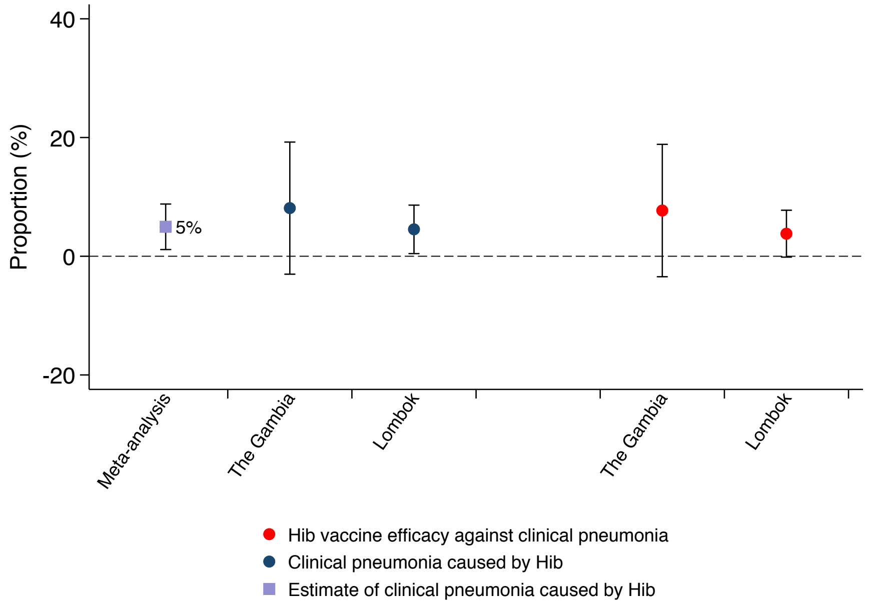

Webappendix 5: Estimates of pathogen-specific meningitis morbidity and mortality.

We used data from studies reporting on the distribution of meningitis cases and adjusted these estimates by the relative pathogen-specific meningitis CFR to calculate the proportion of meningitis deaths due to pneumococcus and Hib. We constrained the total contribution of the most common causes of bacterial meningitis (i.e., pneumococcus, Hib, and *Neisseria meningitidis*) to all-cause meningitis cases and deaths to 83%, based on an analysis of studies reporting on multiple meningitis etiologies using diagnostic techniques with equivalent sensitivity for all pathogens. To derive the proportion of all-cause meningitis deaths attributable to each pathogen, we used the following equation.

$$\%\, Meningitis\ deaths_x = \frac{Meningitis\ CFR_x \times \%\, Meningitis\ cases_x}{\sum_i^z (Meningitis\ CFR_i \times \%\, Meningitis\ cases_i)}$$

where $Meningitis\ CFR_x$ is the case fatality ratio (CFR) for pathogen x, $\%\, Meningitis\ cases_x$ is the proportion of meningitis cases attributable to pathogen x, and the denominator on the right-hand side of the equation is the sum of the products for each pathogen (i.e., i ranges over each of the three pathogens of interest—pneumococcus, Hib, and *Neisseria meningitidis*).

The proportion of meningitis cases attributable to pneumococcus and Hib in the equation above was determined by using data from observational studies identified from the literature rather than the vaccine probe approach was used for estimating pathogen-specific pneumonia cases and deaths. The basis for this decision was that the etiologic diagnostic yield from investigations of meningitis cases is high when conducted with appropriate clinical and microbiologic methods, unlike pneumonia. We included only surveillance studies lasting more than 12 months and conducted in the absence of conjugate vaccine. Data from studies reporting on etiologically-confirmed bacterial meningitis cases attributable to the most common causes of bacterial meningitis (i.e., pneumococcus, Hib, and *Neisseria meningitidis*) were combined in random effects meta-analyses to develop region-specific summary estimates of the relative distribution of meningitis cases by these pathogens.

We estimated pathogen-specific meningitis CFR by random effects meta-analyses of data from the systematic literature review, stratified by child mortality setting: low (<30 deaths per 1,000 live births), medium (30-<75), high (75-<150), and very high (≥150). Because reported CFR values reflect mortality in children who access care at a study site or health facility, we adjusted them to account for the higher CFR (i.e., 90% for all pathogens) assumed for those not accessing care. There is no measure of care seeking for children with meningitis in standardized surveys from India; therefore, we used state-specific values for the proportion of children seeking care for pneumonia symptoms from National Family and Household Survey (NFHS) as a proxy. We assumed 100% access to care in states with child mortality below 30 deaths per 1,000 live births.

Webappendix 6: Methods used to estimate pathogen-specific non-pneumonia, non-meningitis (NPNM) cases and deaths

We estimated pathogen-specific morbidity due to other invasive syndromes (e.g., sepsis) from studies reporting on the ratio of pathogen-specific NPNM cases to pathogen-specific meningitis cases. These estimates were combined using random effects meta-analyses, stratifying the data first by child mortality setting. For pneumococcus, we stratified NPNM cases by severe and non-severe cases. NPNM deaths were estimated by multiplying country-specific NPNM cases (severe cases for pneumococcus) by country-specific NPNM CFR. The latter was derived by estimating a mortality setting-specific ratio of pathogen-specific NPNM CFR and pathogen-specific meningitis CFR, and applying that to the country-specific pneumococcal meningitis CFR.

There are very few studies globally reporting on NPNM CFR (5 each for pneumococcus and Hib) and only two of these studies for pneumococcus were conducted in epidemiologically relevant settings. Because of the paucity of data for pathogen-specific NPNM from relevant settings, we chose to use the ratio from other settings and then anchor our state-specific pathogen NPNM CFR estimates on the state-specific pathogen meningitis CFR estimates. Because we chose to anchor our pathogen NPNM estimates on the pathogen meningitis estimates, we used the pathogen meningitis uncertainty as the primary source of uncertainty in our estimates.

Webappendix 7: Equation used to account for increased risk of Hib and pneumococcal disease in children infected with HIV

We used the following equation to prepare annual mortality and morbidity estimates due to each syndrome (i.e., pneumonia, meningitis, and NPNM) in HIV-infected children for each pathogen:

$$HIV^{+}\ deaths_{pathogen,syndrome} = \frac{HIV\ \%\ \times\ Population_{1-59\ mos.}\ \times\ HIV^{-}\ deaths_{pathogen,syndrome}\ \times\ ID\ Relative\ Risk_{pathogen}}{(1 - HIV\ \%)\ \times\ Population_{1-59\ mos.}}$$

Where $HIV\ \%$ represents the proportion of children infected with HIV and not on HAART, $Population_{1-59\ mos.}$ is the total population of children 1-59 months in a state, $HIV^{-}\ deaths_{pathogen,syndrome}$ is the estimated number of deaths due to the pathogen by syndrome, and $ID\ Relative\ Risk_{pathogen}$ is the relative risk of invasive disease for pneumococcus (40.96) or Hib (7.39) in children infected with HIV.

Webappendix 8: Comparison with previously published India subnational pneumococcal pneumonia estimates

Table 4: Comparison of pneumococcal pneumonia deaths in 2010 comparing estimates Farooqui, et al. and current study

	Farooqui, et al.		Current study		
State or UT	Pneumococcal Pneumonia Deaths	Rank	Pneumococcal Pneumonia Deaths	Rank	Absolute difference
A&N Islands			<100	33	-
Andhra Pradesh	934	13	1,700	12	766
Arunachal Pradesh	0	20	<100	26	70
Assam	2,200	9	3,500	5	1,300
Bihar	23,200	2	9,800	2	13,400
Chandigarh			<100	29	-
Chhattisgarh	2,100	10	1,700	13	400
D&N Haveli			<100	31	-
Daman & Diu			<100	34	-
Delhi	200	18	430	20	230
Goa	0	20	<100	32	10
Gujarat	2,600	8	2,900	7	300
Haryana	1,400	12	1,400	14	0
Himachal Pradesh	200	18	230	23	30
J & K	600	16	320	21	280
Jharkhand	6,300	5	1,800	11	4,500
Karnataka	1,400	12	2,400	9	1,000
Kerala	0	20	290	22	290
Lakshadweep			<100	35	-
Madhya Pradesh	13,400	3	8,100	3	5,300
Maharashtra	2,000	11	2,300	10	300
Manipur	100	19	<100	28	40
Meghalaya	100	19	540	18	440
Mizoram	0	20	110	25	110
Nagaland	100	19	120	24	20
Odisha	3,400	6	3,100	6	300
Puducherry			<100	30	-
Punjab	600	16	800	17	200
Rajasthan	11,900	4	5,700	4	6,200
Sikkim	0	20	<100	31	10
Tamil Nadu	300	17	1,300	15	1,000
Telangana	666	15	1,240	16	574
Tripura	100	19	<100	27	30
Uttar Pradesh	27,800	1	21,500	1	6,300
Uttarakhand	900	14	470	19	430
West Bengal	2,900	7	2,400	8	500

Farooqui, et al. estimated the proportion of severe pneumonia cases caused by pneumococcus using the efficacy of PCV against radiograph-confirmed pneumonia, rather than efficacy against severe pneumonia, from a vaccine trial conducted in the Philippines.[14] The values used to adjust the pneumonia vaccine efficacy value from the Philippines trial for serotype coverage and incomplete vaccine efficacy were from an IPD surveillance study conducted in India[15] and a vaccine clinical trial in South Africa,[16] respectively, rather than applicable data from the Philippines trial. Hib vaccine use was not accounted for. Farooqui, et al. applied this estimate to state-level severe all-cause pneumonia estimates. To derive pneumococcal pneumonia deaths, Farooqui, et al. applied a summary estimate of pneumococcal pneumonia case fatality (16.7%) from two observational studies in India[17 18] to severe pneumococcal pneumonia case estimates. Pathogen-specific pneumonia case fatality is often overestimated, as pathogen isolation is more likely in the most severe cases.

References

1. Wolfson L, O'Brien K, Watt J, et al. Methods to estimate the global burden of disease due to Haemophilus influenzae type b and Streptococcus pneumoniae in children less than 5 years of age. *Lancet Web annex* 2009:893-902.
2. Knoll M, O'Brien K, Henkle E, et al. Global literature review of Haemophilus influenzae type b and Streptococcus pneumoniae invasive disease among children less than five years of age, 1980–2005. *Geneva, Switzerland: World Health Organization* 2009
3. Wahl B, O'Brien KL, Greenbaum A, et al. Burden of Streptococcus pneumoniae and Haemophilus influenzae type b disease in children in the era of conjugate vaccines: global, regional, and national estimates for 2000-15. *Lancet Glob Health* 2018;6(7):e744-e57. doi: 10.1016/S2214-109X(18)30247-X [published Online First: 2018/06/16]
4. O'Brien KL, Wolfson LJ, Watt JP, et al. Burden of disease caused by Streptococcus pneumoniae in children younger than 5 years: global estimates. *Lancet* 2009;374(9693):893-902. doi: 10.1016/S0140-6736(09)61204-6
5. Watt JP, Wolfson LJ, O'Brien KL, et al. Burden of disease caused by Haemophilus influenzae type b in children younger than 5 years: global estimates. *Lancet* 2009;374(9693):903-11. doi: 10.1016/S0140-6736(09)61203-4
6. McAllister DA, Liu L, Shi T, et al. Global, regional, and national estimates of pneumonia morbidity and mortality in children younger than 5 years between 2000 and 2015: a systematic analysis. *The Lancet Global Health* 2018 doi: 10.1016/S2214-109X(18)30408-X
7. Mulholland EK. Use of vaccine trials to estimate burden of disease. *J Health Popul Nutr* 2004;22(3):257-67.
8. World Health Organization. Programme for the control of acute respiratory infections: technical bases for the WHO recommendations on the management of pneumonia in children at first level health facilities. 1991
9. World Health Organization. Handbook IMCI: Integrated management of childhood illness. Geneva2005.
10. Cherian T, Mulholland EK, Carlin JB, et al. Standardized interpretation of paediatric chest radiographs for the diagnosis of pneumonia in epidemiological studies. *Bulletin of the World Health Organization* 2005;83(5):353-9. doi: /S0042-96862005000500011
11. Schmidt FL, Oh IS, Hayes TL. Fixed- versus random-effects models in meta-analysis: model properties and an empirical comparison of differences in results. *The British journal of mathematical and statistical psychology* 2009;62(Pt 1):97-128. doi: 10.1348/000711007x255327 [published Online First: 2007/11/16]
12. Cutts FT, Zaman SM, Enwere G, et al. Efficacy of nine-valent pneumococcal conjugate vaccine against pneumonia and invasive pneumococcal disease in The Gambia: randomised, double-blind, placebo-controlled trial. *Lancet* 2005;365(9465):1139-46. doi: 10.1016/S0140-6736(05)71876-6
13. Gessner BD, Sutanto A, Linehan M, et al. Incidences of vaccine-preventable Haemophilus influenzae type b pneumonia and meningitis in Indonesian children: hamlet-randomised vaccine-probe trial. *Lancet* 2005;365(9453):43-52.
14. Lucero MG, Nohynek H, Williams G, et al. Efficacy of an 11-valent pneumococcal conjugate vaccine against radiologically confirmed pneumonia among children less than 2 years of age in the Philippines: a randomized, double-blind, placebo-controlled trial. *Pediatr Infect Dis J* 2009;28(6):455-62. doi: 10.1097/INF.0b013e31819637af
15. Manoharan A, Manchanda V, Balasubramanian S, et al. Invasive pneumococcal disease in children aged younger than 5 years in India: a surveillance study. *The Lancet Infectious diseases* 2017;17(3):305-12. doi: 10.1016/S1473-3099(16)30466-2
16. Klugman KP, Madhi SA, Huebner RE, et al. A trial of a 9-valent pneumococcal conjugate vaccine in children with and those without HIV infection. *The New England journal of medicine* 2003;349(14):1341-8. doi: 10.1056/NEJMoa035060
17. Gupta M, Kumar R, Deb AK, et al. Multi-center surveillance for pneumonia & meningitis among children (<2 yr) for Hib vaccine probe trial preparation in India. *Indian J Med Res* 2010;131:649-58.
18. Prospective multicentre hospital surveillance of Streptococcus pneumoniae disease in India. Invasive Bacterial Infection Surveillance (IBIS) Group, International Clinical Epidemiology Network (INCLEN). *Lancet* 1999;353(9160):1216-21.

Webappendix 9: Pneumococcal deaths in India by state, year, and HIV status

		Pneumonia Deaths				Meningitis deaths				NPNM deaths			
Year	State	Spn pneumonia deaths (UR)	Spn pneumonia deaths; HIV (UR)	Spn pneumonia mortality rate (UR)	Spn pneumonia mortality rate; HIV (UR)	Spn meningitis deaths (UR)	Spn meningitis deaths; HIV (UR)	Spn meningitis mortality rate (UR)	Spn meningitis mortality rate; HIV (UR)	Spn NPNM deaths (UR)	Spn NPNM deaths; HIV (UR)	Spn NPNM mortality rate (UR)	Spn NPNM mortality rate; HIV (UR)
2000	Andhra Pradesh	5619 (3984-5856)		73 (52-76)		884 (455-1616)		12 (6-21)		278 (143-508)		4 (2-7)	
2001	Andhra Pradesh	5471 (3880-5702)		71 (51-74)		858 (405-1613)		11 (5-21)		764 (360-1436)		10 (5-19)	
2002	Andhra Pradesh	5286 (3748-5508)		69 (49-72)		827 (391-1558)		11 (5-20)		736 (348-1387)		10 (5-18)	
2003	Andhra Pradesh	5066 (3592-5279)		67 (47-70)		791 (375-1494)		10 (5-20)		705 (334-1330)		9 (4-18)	
2004	Andhra Pradesh	4816 (3415-5019)		64 (45-67)		751 (356-1420)		10 (5-19)		669 (317-1264)		9 (4-17)	
2005	Andhra Pradesh	4550 (3227-4742)		61 (43-64)		709 (336-1343)		10 (4-18)		632 (299-1195)		8 (4-16)	
2006	Andhra Pradesh	4280 (3035-4460)		58 (41-60)		667 (315-1263)		9 (4-17)		593 (280-1125)		8 (4-15)	
2007	Andhra Pradesh	3991 (2830-4159)		54 (38-57)		620 (292-1179)		8 (4-16)		552 (260-1049)		8 (4-14)	
2008	Andhra Pradesh	3701 (2624-3857)		51 (36-53)		573 (271-1093)		8 (4-15)		510 (241-973)		7 (3-13)	
2009	Andhra Pradesh	3391 (2405-3534)		47 (33-49)		523 (241-1008)		7 (3-14)		465 (215-897)		6 (3-12)	
2010	Andhra Pradesh	3074 (2180-3203)		43 (31-45)		471 (212-918)		7 (3-13)		419 (189-817)		6 (3-12)	
2011	Andhra Pradesh	2763 (1959-2879)		39 (28-41)		421 (185-830)		6 (3-12)		375 (165-739)		5 (2-11)	
2012	Andhra Pradesh	2440 (1730-2542)		35 (25-37)		370 (159-736)		5 (2-11)		330 (141-656)		5 (2-10)	
2013	Andhra Pradesh	2177 (1544-2269)		32 (23-34)		330 (142-657)		5 (2-10)		294 (126-585)		4 (2-9)	
2014	Andhra Pradesh	1954 (1385-2036)		29 (21-31)		296 (127-590)		4 (2-9)		263 (113-525)		4 (2-8)	
2015	Andhra Pradesh	1818 (1289-1894)	25 (18-27)	28 (20-29)	0 (0-0)	275 (118-549)	4 (2-8)	4 (2-8)	0 (0-0)	245 (105-489)	4 (2-7)	4 (2-7)	0 (0-0)
2000	Arunachal Pradesh	78 (55-81)		50 (35-52)		11 (4-25)		7 (3-16)		10 (4-22)		6 (2-14)	
2001	Arunachal Pradesh	73 (52-76)		46 (33-48)		11 (4-23)		7 (2-15)		9 (3-21)		6 (2-13)	
2002	Arunachal Pradesh	73 (52-76)		46 (33-48)		10 (4-23)		7 (2-14)		9 (3-20)		6 (2-13)	
2003	Arunachal Pradesh	74 (52-77)		46 (33-48)		11 (4-23)		7 (2-15)		9 (3-21)		6 (2-13)	
2004	Arunachal Pradesh	73 (52-76)		46 (33-48)		11 (4-23)		7 (2-14)		10 (3-21)		6 (2-13)	
2005	Arunachal Pradesh	72 (51-75)		45 (32-47)		11 (4-22)		7 (3-14)		9 (4-20)		6 (2-12)	
2006	Arunachal Pradesh	75 (53-78)		47 (33-49)		11 (5-23)		7 (3-14)		10 (4-20)		6 (3-13)	
2007	Arunachal Pradesh	71 (51-74)		44 (31-46)		11 (5-21)		7 (3-13)		10 (5-19)		6 (3-12)	
2008	Arunachal Pradesh	72 (51-75)		45 (32-47)		11 (5-21)		7 (3-13)		10 (5-19)		6 (3-12)	
2009	Arunachal Pradesh	72 (51-75)		45 (32-47)		11 (5-22)		7 (3-14)		10 (4-19)		6 (3-12)	
2010	Arunachal Pradesh	72 (51-75)		45 (32-47)		11 (4-22)		7 (3-14)		9 (4-20)		6 (2-12)	
2011	Arunachal Pradesh	69 (49-72)		43 (31-45)		10 (4-22)		6 (2-14)		9 (3-19)		6 (2-12)	
2012	Arunachal Pradesh	66 (47-69)		42 (30-44)		10 (3-21)		6 (2-13)		9 (3-19)		5 (2-12)	
2013	Arunachal Pradesh	64 (45-66)		41 (29-42)		9 (3-20)		6 (2-13)		8 (3-18)		5 (2-11)	
2014	Arunachal Pradesh	61 (43-63)		39 (28-41)		9 (3-19)		6 (2-12)		8 (3-17)		5 (2-11)	
2015	Arunachal Pradesh	57 (40-59)	1 (1-1)	37 (26-38)	1 (0-1)	8 (3-18)	0 (0-0)	5 (2-12)	0 (0-0)	7 (3-16)	0 (0-0)	5 (2-10)	0 (0-0)
2000	Assam	4199 (2977-4376)		122 (87-127)		620 (264-1274)		18 (8-37)		195 (83-400)		6 (2-12)	
2001	Assam	4056 (2876-4227)		117 (83-122)		599 (255-1230)		17 (7-35)		188 (80-387)		5 (2-11)	
2002	Assam	3943 (2796-4109)		113 (80-117)		583 (248-1196)		17 (7-34)		183 (78-376)		5 (2-11)	
2003	Assam	3865 (2741-4028)		110 (78-114)		571 (243-1172)		16 (7-33)		180 (76-369)		5 (2-10)	
2004	Assam	3806 (2699-3966)		108 (76-112)		564 (239-1154)		16 (7-33)		177 (75-363)		5 (2-10)	
2005	Assam	3757 (2664-3915)		106 (75-110)		558 (240-1135)		16 (7-32)		175 (76-357)		5 (2-10)	
2006	Assam	3706 (2628-3863)		104 (74-108)		552 (241-1116)		15 (7-31)		173 (76-351)		5 (2-10)	
2007	Assam	3678 (2608-3833)		103 (73-107)		549 (243-1104)		15 (7-31)		173 (77-347)		5 (2-10)	
2008	Assam	3657 (2593-3811)		102 (72-106)		547 (242-1098)		15 (7-31)		172 (76-345)		5 (2-10)	
2009	Assam	3609 (2559-3761)		100 (71-105)		540 (239-1083)		15 (7-30)		170 (75-341)		5 (2-9)	
2010	Assam	3576 (2536-3727)		100 (71-104)		535 (237-1073)		15 (7-30)		168 (74-337)		5 (2-9)	
2011	Assam	3544 (2513-3694)		99 (70-103)		530 (235-1064)		15 (7-30)		167 (74-334)		5 (2-9)	
2012	Assam	3487 (2473-3634)		98 (69-102)		521 (231-1047)		15 (6-29)		164 (73-329)		5 (2-9)	
2013	Assam	3481 (2468-3627)		98 (70-103)		520 (230-1045)		15 (7-30)		163 (72-328)		5 (2-9)	
2014	Assam	3442 (2441-3587)		98 (69-102)		513 (228-1033)		15 (6-29)		161 (72-325)		5 (2-9)	
2015	Assam	3403 (2413-3546)	48 (34-50)	97 (69-101)	1 (1-1)	506 (196-1050)	8 (3-16)	14 (6-30)	0 (0-0)	451 (175-935)	7 (3-14)	13 (5-27)	0 (0-0)
2000	Bihar	15229 (10799-15871)		123 (87-129)		2427 (1242-4374)		20 (10-35)		763 (390-1375)		6 (3-11)	
2001	Bihar	14837 (10521-15462)		118 (83-123)		2365 (1210-4261)		19 (10-34)		743 (380-1340)		6 (3-11)	
2002	Bihar	14372 (10191-14978)		112 (79-117)		2290 (1172-4128)		18 (9-32)		720 (368-1298)		6 (3-10)	
2003	Bihar	13877 (9840-14462)		107 (76-111)		2207 (1132-3986)		17 (9-31)		694 (356-1253)		5 (3-10)	
2004	Bihar	13356 (9471-13919)		101 (72-106)		2117 (1089-3836)		16 (8-29)		665 (342-1206)		5 (3-9)	
2005	Bihar	12843 (9107-13385)		96 (68-100)		2024 (1035-3698)		15 (8-28)		636 (325-1163)		5 (2-9)	
2006	Bihar	12339 (8749-12859)		91 (64-95)		1931 (895-3652)		14 (7-27)		1719 (797-3251)		13 (6-24)	
2007	Bihar	11836 (8393-12335)		86 (61-90)		1842 (846-3512)		13 (6-26)		1640 (753-3127)		12 (5-23)	
2008	Bihar	11312 (8022-11789)		81 (58-85)		1754 (809-3357)		13 (6-24)		1561 (720-2989)		11 (5-22)	
2009	Bihar	10721 (7602-11172)		77 (54-80)		1659 (766-3181)		12 (5-23)		1477 (682-2832)		11 (5-20)	
2010	Bihar	10123 (7178-10549)		72 (51-75)		1566 (724-3004)		11 (5-21)		1394 (644-2674)		10 (5-19)	
2011	Bihar	9489 (6728-9889)		67 (47-70)		1468 (678-2816)		10 (5-20)		1307 (604-2507)		9 (4-18)	
2012	Bihar	8736 (6195-9104)		61 (43-64)		1351 (625-2592)		9 (4-18)		1203 (556-2308)		8 (4-16)	
2013	Bihar	8071 (5723-8412)		56 (40-59)		1248 (577-2395)		9 (4-17)		1111 (514-2132)		8 (4-15)	
2014	Bihar	7335 (5201-7644)		51 (36-53)		1134 (524-2177)		8 (4-15)		1010 (467-1938)		7 (3-13)	
2015	Bihar	6659 (4722-6940)	93 (66-97)	46 (33-48)	1 (0-1)	1030 (476-1976)	15 (7-29)	7 (3-14)	0 (0-0)	917 (424-1759)	14 (6-26)	6 (3-12)	0 (0-0)
2000	Chhattisgarh	2850 (2021-2970)		102 (73-107)		423 (183-861)		15 (7-31)		133 (57-271)		5 (2-10)	
2001	Chhattisgarh	2823 (2002-2942)		101 (71-105)		419 (181-853)		15 (6-30)		132 (57-268)		5 (2-10)	
2002	Chhattisgarh	2742 (1944-2857)		97 (69-101)		407 (176-828)		14 (6-29)		128 (55-260)		5 (2-9)	
2003	Chhattisgarh	2646 (1877-2758)		93 (66-97)		394 (170-799)		14 (6-28)		124 (53-251)		4 (2-9)	
2004	Chhattisgarh	2500 (1772-2605)		88 (62-92)		373 (160-755)		13 (6-27)		117 (50-237)		4 (2-8)	
2005	Chhattisgarh	2368 (1679-2468)		83 (59-87)		355 (159-709)		12 (6-25)		112 (50-223)		4 (2-8)	
2006	Chhattisgarh	2292 (1625-2388)		80 (57-84)		346 (161-679)		12 (6-24)		109 (51-214)		4 (2-7)	
2007	Chhattisgarh	2129 (1509-2218)		74 (53-78)		322 (141-641)		11 (5-22)		287 (125-571)		10 (4-20)	
2008	Chhattisgarh	2018 (1431-2103)		71 (50-74)		306 (133-608)		11 (5-21)		272 (119-541)		10 (4-19)	
2009	Chhattisgarh	1876 (1330-1955)		66 (47-69)		285 (124-565)		10 (4-20)		253 (110-503)		9 (4-18)	
2010	Chhattisgarh	1726 (1224-1798)		61 (43-63)		262 (114-520)		9 (4-18)		233 (101-463)		8 (4-16)	
2011	Chhattisgarh	1615 (1145-1683)		57 (40-59)		245 (107-486)		9 (4-17)		218 (95-433)		8 (3-15)	
2012	Chhattisgarh	1495 (1060-1558)		53 (38-55)		227 (99-450)		8 (4-16)		202 (88-401)		7 (3-14)	
2013	Chhattisgarh	1438 (1020-1499)		52 (37-54)		218 (95-433)		8 (3-16)		194 (85-386)		7 (3-14)	
2014	Chhattisgarh	1423 (1009-1483)		51 (36-54)		216 (94-428)		8 (3-15)		192 (84-381)		7 (3-14)	
2015	Chhattisgarh	1462 (1037-1524)	21 (15-21)	53 (38-55)	1 (1-1)	222 (97-440)	3 (1-7)	8 (4-16)	0 (0-0)	197 (86-392)	3 (1-6)	7 (3-14)	0 (0-0)
2000	Delhi	827 (586-862)		53 (37-55)		124 (51-252)		8 (3-16)		110 (46-224)		7 (3-14)	
2001	Delhi	795 (564-828)		50 (36-53)		119 (49-242)		8 (3-15)		106 (44-215)		7 (3-14)	
2002	Delhi	760 (539-792)		48 (34-50)		114 (47-231)		7 (3-15)		101 (42-206)		6 (3-13)	
2003	Delhi	723 (512-753)		46 (32-48)		109 (45-220)		7 (3-14)		97 (40-196)		6 (3-12)	
2004	Delhi	683 (485-712)		43 (31-45)		104 (42-208)		7 (3-13)		92 (38-185)		6 (2-12)	

Spn = *Streptococcus pneumoniae*; npnm = non-pneumonia, non-meningitis; HIV = HIV-infected cases only; UR = uncertainty range

Year	State	Spn pneumonia deaths (UR)	Spn pneumonia deaths; HIV (UR)	Spn pneumonia mortality rate (UR)	Spn pneumonia mortality rate; HIV (UR)	Spn meningitis deaths (UR)	Spn meningitis deaths; HIV (UR)	Spn meningitis mortality rate (UR)	Spn meningitis mortality rate; HIV (UR)	Spn NPNM deaths (UR)	Spn NPNM deaths; HIV (UR)	Spn NPNM mortality rate (UR)	Spn NPNM mortality rate; HIV (UR)
2005	Delhi	644 (457-671)		41 (29-42)		99 (44-192)		6 (3-12)		88 (40-171)		6 (3-11)	
2006	Delhi	605 (429-630)		38 (27-40)		95 (47-177)		6 (3-11)		85 (42-157)		5 (3-10)	
2007	Delhi	565 (400-588)		36 (25-37)		91 (50-161)		6 (3-10)		81 (45-143)		5 (3-9)	
2008	Delhi	525 (372-547)		33 (24-35)		85 (47-150)		5 (3-10)		76 (41-133)		5 (3-8)	
2009	Delhi	484 (343-505)		31 (22-32)		80 (43-138)		5 (3-9)		71 (38-123)		5 (2-8)	
2010	Delhi	443 (314-462)		29 (20-30)		74 (39-126)		5 (3-8)		65 (35-112)		4 (2-7)	
2011	Delhi	402 (285-419)		26 (19-27)		67 (36-115)		4 (2-7)		60 (32-102)		4 (2-7)	
2012	Delhi	356 (252-371)		23 (17-24)		60 (30-121)		4 (2-8)		54 (27-108)		4 (2-7)	
2013	Delhi	314 (222-327)		21 (15-22)		54 (27-107)		4 (2-7)		48 (24-95)		3 (2-6)	
2014	Delhi	269 (191-280)		18 (13-19)		47 (23-92)		3 (2-6)		41 (20-82)		3 (1-5)	
2015	Delhi	228 (162-238)	3 (2-3)	15 (11-16)	0 (0-0)	40 (19-78)	1 (0-1)	3 (1-5)	0 (0-0)	35 (17-69)	1 (0-1)	2 (1-5)	0 (0-0)
2000	Goa	17 (12-18)		15 (10-15)		3 (1-6)		2 (1-5)		3 (1-5)		2 (1-4)	
2001	Goa	15 (10-15)		12 (9-13)		2 (1-5)		2 (1-4)		2 (1-4)		2 (1-4)	
2002	Goa	10 (7-11)		9 (6-9)		2 (1-3)		1 (1-3)		2 (1-3)		1 (1-3)	
2003	Goa	13 (9-13)		11 (8-11)		2 (1-4)		2 (1-4)		2 (1-4)		2 (1-3)	
2004	Goa	13 (9-14)		11 (8-12)		2 (1-5)		2 (1-4)		2 (1-4)		2 (1-3)	
2005	Goa	14 (10-14)		12 (8-12)		2 (1-5)		2 (1-4)		2 (1-4)		2 (1-4)	
2006	Goa	12 (8-12)		10 (7-11)		2 (1-4)		2 (1-4)		2 (1-4)		1 (1-3)	
2007	Goa	8 (6-9)		7 (5-7)		1 (1-3)		1 (0-2)		1 (0-3)		1 (0-2)	
2008	Goa	7 (5-7)		6 (4-6)		1 (0-2)		1 (0-2)		1 (0-2)		1 (0-2)	
2009	Goa	8 (6-8)		7 (5-7)		1 (1-3)		1 (0-2)		1 (0-3)		1 (0-2)	
2010	Goa	8 (6-8)		7 (5-7)		1 (1-3)		1 (0-2)		1 (0-2)		1 (0-2)	
2011	Goa	6 (4-6)		5 (4-6)		1 (0-2)		1 (0-2)		1 (0-2)		1 (0-2)	
2012	Goa	4 (3-5)		4 (3-4)		1 (0-2)		1 (0-1)		1 (0-2)		1 (0-1)	
2013	Goa	3 (2-3)		3 (2-3)		1 (0-1)		1 (0-1)		0 (0-1)		0 (0-1)	
2014	Goa	2 (2-3)		2 (2-2)		0 (0-1)		0 (0-1)		0 (0-1)		0 (0-1)	
2015	Goa	2 (1-2)	0 (0-0)	2 (1-2)	0 (0-0)	0 (0-1)	0 (0-0)	0 (0-1)	0 (0-0)	0 (0-1)	0 (0-0)	0 (0-0)	0 (0-0)
2000	Gujarat	5583 (3959-5819)		93 (66-97)		831 (364-1680)		14 (6-28)		261 (115-528)		4 (2-9)	
2001	Gujarat	5317 (3770-5541)		88 (62-92)		790 (347-1600)		13 (6-27)		248 (109-503)		4 (2-8)	
2002	Gujarat	5063 (3590-5276)		83 (59-87)		751 (330-1524)		12 (5-25)		236 (104-479)		4 (2-8)	
2003	Gujarat	4771 (3383-4972)		78 (56-82)		707 (271-1477)		12 (4-24)		630 (241-1315)		10 (4-22)	
2004	Gujarat	4413 (3129-4599)		72 (51-75)		656 (250-1366)		11 (4-22)		584 (223-1217)		10 (4-20)	
2005	Gujarat	4195 (2974-4371)		69 (49-71)		628 (256-1281)		10 (4-21)		559 (228-1141)		9 (4-19)	
2006	Gujarat	4022 (2852-4192)		66 (46-68)		608 (267-1210)		10 (4-20)		542 (237-1077)		9 (4-18)	
2007	Gujarat	3765 (2670-3924)		61 (43-64)		576 (273-1114)		9 (4-18)		513 (243-992)		8 (4-16)	
2008	Gujarat	3531 (2503-3679)		57 (41-60)		545 (256-1045)		9 (4-17)		485 (228-930)		8 (4-15)	
2009	Gujarat	3275 (2322-3413)		53 (38-56)		508 (237-970)		8 (4-16)		452 (211-863)		7 (3-14)	
2010	Gujarat	3021 (2142-3149)		49 (35-52)		469 (219-894)		8 (4-15)		417 (195-796)		7 (3-13)	
2011	Gujarat	2809 (1992-2928)		46 (33-48)		436 (204-832)		7 (3-14)		388 (181-740)		6 (3-12)	
2012	Gujarat	2617 (1855-2727)		43 (31-45)		406 (190-775)		7 (3-13)		362 (169-690)		6 (3-11)	
2013	Gujarat	2496 (1770-2601)		42 (30-43)		387 (181-739)		6 (3-12)		345 (161-658)		6 (3-11)	
2014	Gujarat	2375 (1684-2475)		40 (28-42)		369 (172-703)		6 (3-12)		328 (153-626)		6 (3-11)	
2015	Gujarat	2257 (1601-2352)	32 (22-33)	38 (27-40)	1 (0-1)	350 (164-668)	5 (2-10)	6 (3-11)	0 (0-0)	312 (146-595)	5 (2-9)	5 (2-10)	0 (0-0)
2000	Haryana	3092 (2192-3222)		120 (85-125)		485 (240-897)		19 (9-35)		152 (76-282)		6 (3-11)	
2001	Haryana	2936 (2082-3060)		113 (80-118)		460 (228-852)		18 (9-33)		145 (72-268)		6 (3-10)	
2002	Haryana	2767 (1962-2884)		106 (75-110)		433 (215-803)		17 (8-31)		136 (68-252)		5 (3-10)	
2003	Haryana	2587 (1834-2696)		98 (70-103)		405 (201-751)		15 (8-29)		127 (63-236)		5 (2-9)	
2004	Haryana	2396 (1699-2497)		91 (64-95)		375 (168-713)		14 (6-27)		334 (150-635)		13 (6-24)	
2005	Haryana	2172 (1540-2264)		82 (58-86)		343 (163-639)		13 (6-24)		305 (145-569)		12 (5-22)	
2006	Haryana	1941 (1376-2022)		73 (52-76)		309 (156-563)		12 (6-21)		275 (139-502)		10 (5-19)	
2007	Haryana	1839 (1304-1916)		69 (49-72)		296 (159-527)		11 (6-20)		263 (142-469)		10 (5-18)	
2008	Haryana	1692 (1200-1764)		64 (45-66)		274 (147-485)		10 (6-18)		244 (130-431)		9 (5-16)	
2009	Haryana	1501 (1065-1565)		57 (40-59)		244 (128-431)		9 (5-16)		217 (114-384)		8 (4-14)	
2010	Haryana	1454 (1031-1515)		55 (39-57)		235 (122-418)		9 (5-16)		210 (109-373)		8 (4-14)	
2011	Haryana	1371 (972-1429)		52 (37-54)		221 (114-396)		8 (4-15)		197 (101-352)		7 (4-13)	
2012	Haryana	1205 (855-1256)		46 (33-48)		194 (99-349)		7 (4-13)		173 (88-311)		7 (3-12)	
2013	Haryana	1223 (868-1275)		47 (33-49)		197 (100-354)		8 (4-14)		175 (89-315)		7 (3-12)	
2014	Haryana	1198 (850-1249)		47 (33-49)		192 (98-347)		7 (4-13)		171 (87-309)		7 (3-12)	
2015	Haryana	1212 (859-1263)	17 (12-18)	47 (34-49)	1 (0-1)	195 (99-351)	3 (1-5)	8 (4-14)	0 (0-0)	173 (88-312)	3 (1-5)	7 (3-12)	0 (0-0)
2000	Himachal Pradesh	544 (386-567)		85 (60-89)		84 (39-162)		13 (6-25)		75 (34-144)		12 (5-23)	
2001	Himachal Pradesh	503 (357-524)		78 (56-82)		78 (36-149)		12 (6-23)		69 (32-133)		11 (5-21)	
2002	Himachal Pradesh	464 (329-484)		72 (51-75)		72 (33-138)		11 (5-22)		64 (29-123)		10 (5-19)	
2003	Himachal Pradesh	429 (304-447)		67 (48-70)		66 (30-127)		10 (5-20)		59 (27-113)		9 (4-18)	
2004	Himachal Pradesh	396 (281-412)		62 (44-65)		61 (28-118)		10 (4-18)		55 (25-105)		9 (4-16)	
2005	Himachal Pradesh	365 (259-380)		58 (41-60)		57 (27-108)		9 (4-17)		51 (24-96)		8 (4-15)	
2006	Himachal Pradesh	337 (239-351)		53 (38-55)		53 (26-99)		8 (4-16)		47 (23-88)		7 (4-14)	
2007	Himachal Pradesh	310 (220-323)		49 (35-51)		49 (25-90)		8 (4-14)		44 (22-80)		7 (3-13)	
2008	Himachal Pradesh	286 (203-298)		46 (32-48)		46 (23-83)		7 (4-13)		41 (20-74)		7 (3-12)	
2009	Himachal Pradesh	263 (186-274)		42 (30-44)		43 (22-76)		7 (4-12)		38 (19-67)		6 (3-11)	
2010	Himachal Pradesh	241 (171-252)		39 (28-41)		40 (21-69)		6 (3-11)		35 (19-61)		6 (3-10)	
2011	Himachal Pradesh	223 (158-232)		37 (26-38)		37 (21-63)		6 (3-10)		33 (18-56)		5 (3-9)	
2012	Himachal Pradesh	207 (147-215)		34 (24-36)		35 (19-58)		6 (3-10)		31 (17-51)		5 (3-9)	
2013	Himachal Pradesh	198 (140-206)		34 (24-35)		34 (18-55)		6 (3-9)		30 (16-49)		5 (3-8)	
2014	Himachal Pradesh	193 (137-201)		33 (24-35)		33 (18-54)		6 (3-9)		29 (16-48)		5 (3-8)	
2015	Himachal Pradesh	194 (138-203)	3 (2-3)	34 (24-35)	0 (0-0)	33 (18-54)	0 (0-1)	6 (3-9)	0 (0-0)	30 (16-48)	0 (0-1)	5 (3-8)	0 (0-0)
2000	Jammu & Kashmir	646 (458-673)		61 (43-64)		112 (59-179)		11 (6-17)		100 (52-159)		9 (5-15)	
2001	Jammu & Kashmir	607 (431-633)		55 (39-57)		106 (55-168)		10 (5-15)		94 (49-150)		9 (4-14)	
2002	Jammu & Kashmir	571 (405-595)		50 (35-52)		99 (52-158)		9 (5-14)		88 (46-141)		8 (4-12)	
2003	Jammu & Kashmir	537 (381-560)		45 (32-47)		93 (49-149)		8 (4-12)		82 (43-133)		7 (4-11)	
2004	Jammu & Kashmir	506 (359-527)		41 (29-43)		86 (46-140)		7 (4-11)		77 (41-125)		6 (3-10)	
2005	Jammu & Kashmir	476 (337-496)		37 (26-39)		79 (44-134)		6 (3-10)		71 (39-120)		5 (3-9)	
2006	Jammu & Kashmir	447 (317-466)		33 (24-35)		73 (38-128)		5 (3-10)		65 (34-114)		5 (3-9)	
2007	Jammu & Kashmir	418 (296-436)		30 (21-31)		68 (33-122)		5 (2-9)		60 (30-108)		4 (2-8)	
2008	Jammu & Kashmir	390 (277-406)		27 (19-28)		62 (31-113)		4 (2-8)		56 (28-101)		4 (2-7)	
2009	Jammu & Kashmir	360 (255-375)		24 (17-25)		57 (29-105)		4 (2-7)		51 (25-93)		3 (2-6)	
2010	Jammu & Kashmir	329 (233-343)		22 (15-22)		52 (26-96)		3 (2-6)		47 (23-85)		3 (2-6)	
2011	Jammu & Kashmir	298 (211-310)		19 (13-20)		47 (24-87)		3 (1-5)		42 (21-77)		3 (1-5)	

Spn = *Streptococcus pneumoniae*; npnm = non-pneumonia, non-meningitis; HIV = HIV-infected cases only; UR = uncertainty range

Year	State	Spn pneumonia deaths (UR)	Spn pneumonia deaths; HIV (UR)	Spn pneumonia mortality rate (UR)	Spn pneumonia mortality rate; HIV (UR)	Spn meningitis deaths (UR)	Spn meningitis deaths; HIV (UR)	Spn meningitis mortality rate (UR)	Spn meningitis mortality rate; HIV (UR)	Spn NPNM deaths (UR)	Spn NPNM deaths; HIV (UR)	Spn NPNM mortality rate (UR)	Spn NPNM mortality rate; HIV (UR)
2012	Jammu & Kashmir	264 (187-275)		16 (12-17)		42 (21-77)		3 (1-5)		37 (19-68)		2 (1-4)	
2013	Jammu & Kashmir	237 (168-247)		14 (10-15)		38 (19-69)		2 (1-4)		34 (17-61)		2 (1-4)	
2014	Jammu & Kashmir	216 (153-225)		13 (9-13)		34 (17-63)		2 (1-4)		31 (15-56)		2 (1-3)	
2015	Jammu & Kashmir	205 (145-214)	3 (2-3)	12 (8-12)	0 (0-0)	33 (16-60)	0 (0-1)	2 (1-3)	0 (0-0)	29 (14-53)	0 (0-1)	2 (1-3)	0 (0-0)
2000	Jharkhand	3098 (2197-3228)		84 (60-88)		451 (190-945)		12 (5-26)		142 (60-297)		4 (2-8)	
2001	Jharkhand	3047 (2161-3176)		81 (58-85)		442 (160-957)		12 (4-26)		394 (142-852)		11 (4-23)	
2002	Jharkhand	2957 (2097-3082)		78 (55-81)		429 (155-928)		11 (4-24)		382 (138-827)		10 (4-22)	
2003	Jharkhand	2902 (2058-3025)		76 (54-79)		420 (152-911)		11 (4-24)		374 (136-811)		10 (4-21)	
2004	Jharkhand	2724 (1932-2839)		70 (50-73)		395 (143-855)		10 (4-22)		351 (127-761)		9 (3-20)	
2005	Jharkhand	2470 (1752-2574)		63 (45-66)		358 (131-774)		9 (3-20)		319 (117-689)		8 (3-18)	
2006	Jharkhand	2438 (1728-2540)		62 (44-64)		354 (131-762)		9 (3-19)		315 (117-678)		8 (3-17)	
2007	Jharkhand	2258 (1601-2353)		57 (40-59)		328 (123-704)		8 (3-18)		292 (109-627)		7 (3-16)	
2008	Jharkhand	2018 (1431-2103)		50 (36-52)		294 (110-630)		7 (3-16)		262 (98-561)		7 (2-14)	
2009	Jharkhand	1790 (1269-1865)		44 (31-46)		261 (97-558)		6 (2-14)		232 (87-497)		6 (2-12)	
2010	Jharkhand	1809 (1283-1886)		45 (32-47)		264 (99-564)		7 (2-14)		235 (88-502)		6 (2-12)	
2011	Jharkhand	1727 (1225-1800)		43 (30-44)		252 (94-539)		6 (2-13)		224 (84-480)		6 (2-12)	
2012	Jharkhand	1606 (1139-1674)		40 (28-41)		234 (87-501)		6 (2-12)		208 (78-446)		5 (2-11)	
2013	Jharkhand	1465 (1039-1527)		36 (26-38)		213 (80-457)		5 (2-11)		190 (71-407)		5 (2-10)	
2014	Jharkhand	1317 (934-1372)		32 (23-34)		192 (72-411)		5 (2-10)		171 (64-366)		4 (2-9)	
2015	Jharkhand	1170 (830-1219)	16 (12-17)	29 (20-30)	0 (0-0)	170 (64-365)	3 (1-5)	4 (2-9)	0 (0-0)	152 (57-325)	2 (1-5)	4 (1-8)	0 (0-0)
2000	Karnataka	4146 (2940-4321)		74 (52-77)		627 (270-1252)		11 (5-22)		558 (240-1115)		10 (4-20)	
2001	Karnataka	3957 (2806-4124)		70 (50-73)		598 (257-1195)		11 (5-21)		533 (229-1064)		9 (4-19)	
2002	Karnataka	3843 (2725-4005)		68 (48-70)		581 (250-1161)		10 (4-20)		517 (222-1033)		9 (4-18)	
2003	Karnataka	3734 (2648-3891)		66 (46-68)		566 (243-1128)		10 (4-20)		504 (216-1004)		9 (4-18)	
2004	Karnataka	3613 (2562-3765)		63 (45-66)		550 (235-1091)		10 (4-19)		489 (209-971)		9 (4-17)	
2005	Karnataka	3469 (2460-3616)		61 (43-63)		531 (236-1039)		9 (4-18)		473 (210-925)		8 (4-16)	
2006	Karnataka	3313 (2349-3453)		58 (41-60)		511 (235-984)		9 (4-17)		455 (209-876)		8 (4-15)	
2007	Karnataka	3122 (2214-3254)		55 (39-57)		485 (232-920)		8 (4-16)		432 (207-819)		8 (4-14)	
2008	Karnataka	2915 (2067-3038)		51 (36-53)		457 (217-859)		8 (4-15)		407 (193-764)		7 (3-13)	
2009	Karnataka	2689 (1906-2802)		47 (34-49)		425 (207-787)		7 (4-14)		378 (184-701)		7 (3-12)	
2010	Karnataka	2446 (1734-2549)		43 (31-45)		390 (195-711)		7 (3-13)		347 (174-633)		6 (3-11)	
2011	Karnataka	2197 (1558-2290)		39 (28-41)		353 (182-635)		6 (3-11)		314 (162-565)		6 (3-10)	
2012	Karnataka	1910 (1354-1991)		34 (24-36)		309 (164-548)		6 (3-10)		275 (146-488)		5 (3-9)	
2013	Karnataka	1625 (1152-1693)		29 (21-31)		264 (140-466)		5 (3-8)		235 (124-415)		4 (2-8)	
2014	Karnataka	1308 (928-1364)		24 (17-25)		213 (113-375)		4 (2-7)		190 (100-334)		3 (2-6)	
2015	Karnataka	1017 (721-1060)	14 (10-15)	19 (13-20)	0 (0-0)	166 (87-292)	2 (1-4)	3 (2-5)	0 (0-0)	148 (78-260)	2 (1-4)	3 (1-5)	0 (0-0)
2000	Kerala	372 (264-388)		12 (8-12)		59 (23-133)		2 (1-4)		53 (20-118)		2 (1-4)	
2001	Kerala	360 (255-375)		11 (8-12)		57 (22-128)		2 (1-4)		51 (19-114)		2 (1-4)	
2002	Kerala	347 (246-362)		11 (8-12)		55 (21-124)		2 (1-4)		49 (19-110)		2 (1-4)	
2003	Kerala	337 (239-351)		11 (8-11)		54 (20-120)		2 (1-4)		48 (18-107)		2 (1-3)	
2004	Kerala	327 (232-340)		11 (8-11)		53 (20-116)		2 (1-4)		47 (18-104)		2 (1-3)	
2005	Kerala	318 (226-332)		11 (7-11)		52 (22-112)		2 (1-4)		46 (19-100)		2 (1-3)	
2006	Kerala	313 (222-326)		10 (7-11)		52 (24-108)		2 (1-4)		47 (21-96)		2 (1-3)	
2007	Kerala	308 (218-321)		10 (7-11)		53 (27-105)		2 (1-4)		47 (24-93)		2 (1-3)	
2008	Kerala	306 (217-319)		11 (7-11)		53 (27-104)		2 (1-4)		47 (24-92)		2 (1-3)	
2009	Kerala	304 (215-316)		11 (8-11)		53 (26-103)		2 (1-4)		47 (23-92)		2 (1-3)	
2010	Kerala	302 (214-315)		11 (8-11)		53 (26-103)		2 (1-4)		47 (23-91)		2 (1-3)	
2011	Kerala	302 (214-315)		11 (8-12)		53 (26-103)		2 (1-4)		47 (23-91)		2 (1-3)	
2012	Kerala	299 (212-312)		11 (8-12)		52 (26-102)		2 (1-4)		47 (23-91)		2 (1-3)	
2013	Kerala	301 (213-313)		12 (8-12)		53 (26-102)		2 (1-4)		47 (23-91)		2 (1-3)	
2014	Kerala	300 (213-313)		12 (8-12)		53 (26-102)		2 (1-4)		47 (23-91)		2 (1-4)	
2015	Kerala	303 (215-316)	4 (3-4)	12 (9-13)	0 (0-0)	53 (26-103)	1 (0-2)	2 (1-4)	0 (0-0)	47 (23-92)	1 (0-1)	2 (1-4)	0 (0-0)
2000	Madhya Pradesh	13860 (9828-14444)		165 (117-172)		2033 (847-4228)		24 (10-50)		639 (266-1329)		8 (3-16)	
2001	Madhya Pradesh	13266 (9407-13825)		157 (111-164)		1946 (811-4047)		23 (10-48)		612 (255-1272)		7 (3-15)	
2002	Madhya Pradesh	12711 (9013-13246)		150 (106-156)		1866 (777-3877)		22 (9-46)		587 (244-1219)		7 (3-14)	
2003	Madhya Pradesh	12156 (8620-12668)		143 (102-149)		1787 (743-3708)		21 (9-44)		562 (234-1166)		7 (3-14)	
2004	Madhya Pradesh	11585 (8215-12073)		137 (97-142)		1709 (708-3534)		20 (8-42)		537 (223-1111)		6 (3-13)	
2005	Madhya Pradesh	11047 (7833-11512)		130 (92-136)		1637 (700-3346)		19 (8-39)		515 (220-1052)		6 (3-12)	
2006	Madhya Pradesh	10513 (7455-10956)		124 (88-129)		1567 (691-3160)		18 (8-37)		493 (217-994)		6 (3-12)	
2007	Madhya Pradesh	9958 (7061-10377)		117 (83-122)		1493 (679-2970)		18 (8-35)		469 (214-934)		6 (3-11)	
2008	Madhya Pradesh	9416 (6677-9812)		111 (79-116)		1417 (642-2808)		17 (8-33)		445 (202-883)		5 (2-10)	
2009	Madhya Pradesh	8863 (6285-9237)		105 (75-110)		1336 (605-2643)		16 (7-31)		420 (190-831)		5 (2-10)	
2010	Madhya Pradesh	8330 (5907-8681)		99 (71-104)		1256 (568-2484)		15 (7-30)		395 (179-781)		5 (2-9)	
2011	Madhya Pradesh	7847 (5564-8177)		94 (67-98)		1180 (535-2340)		14 (6-28)		371 (168-736)		4 (2-9)	
2012	Madhya Pradesh	7362 (5220-7672)		89 (63-93)		1104 (502-2196)		13 (6-27)		347 (158-690)		4 (2-8)	
2013	Madhya Pradesh	7041 (4993-7338)		86 (61-90)		1052 (421-2159)		13 (5-26)		937 (375-1922)		11 (5-24)	
2014	Madhya Pradesh	6708 (4757-6991)		83 (59-87)		1000 (401-2057)		12 (5-25)		890 (357-1831)		11 (4-23)	
2015	Madhya Pradesh	6431 (4560-6702)	90 (64-94)	80 (57-84)	1 (1-1)	956 (385-1972)	14 (6-29)	12 (5-25)	0 (0-0)	851 (343-1756)	13 (5-26)	11 (4-22)	0 (0-0)
2000	Maharashtra	5861 (4156-6108)		54 (38-56)		904 (414-1743)		8 (4-16)		804 (368-1552)		7 (3-14)	
2001	Maharashtra	5469 (3878-5699)		50 (36-52)		843 (386-1627)		8 (4-15)		751 (344-1448)		7 (3-13)	
2002	Maharashtra	5074 (3598-5288)		46 (33-48)		783 (358-1509)		7 (3-14)		697 (319-1344)		6 (3-12)	
2003	Maharashtra	4681 (3320-4879)		43 (30-45)		724 (330-1393)		7 (3-13)		644 (294-1240)		6 (3-11)	
2004	Maharashtra	4299 (3049-4481)		40 (28-41)		667 (303-1279)		6 (3-12)		594 (270-1139)		5 (2-10)	
2005	Maharashtra	3944 (2796-4110)		36 (26-38)		616 (290-1164)		6 (3-11)		548 (258-1036)		5 (2-10)	
2006	Maharashtra	3596 (2550-3748)		33 (24-35)		566 (276-1053)		5 (3-10)		503 (246-938)		5 (2-9)	
2007	Maharashtra	3253 (2306-3390)		30 (21-31)		515 (261-945)		5 (2-9)		459 (232-841)		4 (2-8)	
2008	Maharashtra	2944 (2088-3068)		28 (20-29)		469 (236-855)		4 (2-8)		417 (210-761)		4 (2-7)	
2009	Maharashtra	2630 (1865-2741)		25 (18-26)		420 (211-764)		4 (2-7)		374 (188-680)		4 (2-6)	
2010	Maharashtra	2321 (1646-2419)		22 (16-23)		372 (186-674)		4 (2-6)		332 (166-600)		3 (2-6)	
2011	Maharashtra	2077 (1473-2164)		20 (14-21)		335 (167-603)		3 (2-6)		298 (149-537)		3 (1-5)	
2012	Maharashtra	1805 (1280-1881)		18 (12-18)		294 (128-630)		3 (1-6)		261 (114-561)		3 (1-5)	
2013	Maharashtra	1566 (1110-1632)		15 (11-16)		257 (111-546)		3 (1-5)		228 (99-486)		2 (1-5)	
2014	Maharashtra	1337 (948-1393)		13 (9-14)		220 (95-466)		2 (1-5)		196 (85-415)		2 (1-4)	
2015	Maharashtra	1154 (818-1203)	16 (11-17)	12 (8-12)	0 (0-0)	191 (82-403)	3 (1-6)	2 (1-4)	0 (0-0)	170 (73-358)	3 (1-5)	2 (1-4)	0 (0-0)
2000	Manipur	80 (57-83)		35 (25-36)		11 (4-25)		5 (2-11)		10 (4-22)		4 (2-10)	
2001	Manipur	76 (54-79)		32 (23-34)		11 (4-24)		5 (2-10)		10 (3-21)		4 (1-9)	
2002	Manipur	73 (52-76)		30 (21-31)		10 (4-23)		4 (2-10)		9 (3-20)		4 (1-8)	

Spn = *Streptococcus pneumoniae*; npnm = non-pneumonia, non-meningitis; HIV = HIV-infected cases only; UR = uncertainty range

Year	State	Spn pneumonia deaths (UR)	Spn pneumonia deaths; HIV (UR)	Spn pneumonia mortality rate (UR)	Spn pneumonia mortality rate; HIV (UR)	Spn meningitis deaths (UR)	Spn meningitis deaths; HIV (UR)	Spn meningitis mortality rate (UR)	Spn meningitis mortality rate; HIV (UR)	Spn NPNM deaths (UR)	Spn NPNM deaths; HIV (UR)	Spn NPNM mortality rate (UR)	Spn NPNM mortality rate; HIV (UR)
2003	Manipur	69 (49-72)		28 (20-29)		10 (2-26)		4 (1-11)		9 (2-23)		4 (1-9)	
2004	Manipur	66 (47-69)		26 (19-27)		10 (2-25)		4 (1-10)		8 (2-22)		3 (1-9)	
2005	Manipur	65 (46-67)		25 (18-26)		9 (2-24)		4 (1-10)		8 (2-22)		3 (1-8)	
2006	Manipur	63 (45-66)		24 (17-25)		9 (2-24)		3 (1-9)		8 (2-21)		3 (1-8)	
2007	Manipur	63 (45-65)		23 (17-24)		9 (2-24)		3 (1-9)		8 (2-21)		3 (1-8)	
2008	Manipur	63 (45-66)		23 (16-24)		9 (2-24)		3 (1-9)		8 (2-21)		3 (1-8)	
2009	Manipur	63 (45-66)		23 (16-24)		9 (2-24)		3 (1-9)		8 (2-21)		3 (1-8)	
2010	Manipur	64 (45-66)		23 (16-24)		9 (2-24)		3 (1-9)		8 (2-21)		3 (1-8)	
2011	Manipur	64 (45-67)		22 (16-23)		9 (2-24)		3 (1-8)		8 (2-21)		3 (1-7)	
2012	Manipur	63 (45-66)		22 (16-23)		9 (3-24)		3 (1-8)		8 (2-21)		3 (1-7)	
2013	Manipur	63 (45-65)		21 (15-22)		9 (3-23)		3 (1-8)		8 (2-21)		3 (1-7)	
2014	Manipur	61 (43-64)		21 (15-21)		9 (2-23)		3 (1-8)		8 (2-20)		3 (1-7)	
2015	Manipur	59 (42-61)	1 (1-1)	20 (14-20)	0 (0-0)	9 (2-22)	0 (0-0)	3 (1-7)	0 (0-0)	8 (2-19)	0 (0-0)	3 (1-6)	0 (0-0)
2000	Meghalaya	408 (290-426)		113 (80-118)		64 (32-119)		18 (9-33)		20 (10-37)		6 (3-10)	
2001	Meghalaya	425 (301-443)		114 (81-119)		67 (33-123)		18 (9-33)		21 (10-39)		6 (3-10)	
2002	Meghalaya	442 (313-461)		116 (82-121)		69 (34-128)		18 (9-34)		22 (11-40)		6 (3-11)	
2003	Meghalaya	458 (325-477)		118 (83-123)		72 (35-133)		18 (9-34)		23 (11-42)		6 (3-11)	
2004	Meghalaya	473 (335-493)		119 (84-124)		74 (37-137)		19 (9-35)		23 (12-43)		6 (3-11)	
2005	Meghalaya	487 (345-508)		120 (85-125)		77 (39-141)		19 (10-35)		24 (12-44)		6 (3-11)	
2006	Meghalaya	500 (354-521)		120 (85-125)		79 (41-144)		19 (10-35)		25 (13-45)		6 (3-11)	
2007	Meghalaya	511 (362-533)		121 (85-126)		81 (43-146)		19 (10-34)		26 (13-46)		6 (3-11)	
2008	Meghalaya	525 (372-547)		122 (86-127)		83 (44-150)		19 (10-35)		26 (14-47)		6 (3-11)	
2009	Meghalaya	530 (376-552)		121 (86-126)		83 (41-154)		19 (9-35)		26 (13-48)		6 (3-11)	
2010	Meghalaya	554 (393-578)		124 (88-130)		85 (41-163)		19 (9-37)		27 (13-51)		6 (3-11)	
2011	Meghalaya	573 (406-597)		127 (90-132)		87 (40-170)		19 (9-38)		27 (12-54)		6 (3-12)	
2012	Meghalaya	589 (417-614)		129 (91-134)		89 (39-177)		19 (8-39)		28 (12-56)		6 (3-12)	
2013	Meghalaya	612 (434-637)		132 (94-138)		92 (40-184)		20 (9-40)		29 (13-58)		6 (3-13)	
2014	Meghalaya	631 (447-657)		135 (96-141)		94 (41-190)		20 (9-41)		30 (13-60)		6 (3-13)	
2015	Meghalaya	653 (463-680)	9 (6-10)	138 (98-143)	2 (1-2)	97 (43-196)	1 (1-3)	21 (9-41)	0 (0-1)	31 (13-62)	0 (0-1)	6 (3-13)	0 (0-0)
2000	Mizoram	129 (91-134)		114 (81-119)		19 (8-39)		17 (7-35)		6 (2-12)		5 (2-11)	
2001	Mizoram	124 (88-129)		108 (76-112)		18 (7-38)		16 (6-33)		6 (2-12)		5 (2-10)	
2002	Mizoram	122 (86-127)		103 (73-108)		18 (7-37)		15 (6-32)		6 (2-12)		5 (2-10)	
2003	Mizoram	120 (85-125)		100 (71-104)		18 (7-37)		15 (6-31)		6 (2-12)		5 (2-10)	
2004	Mizoram	119 (84-124)		97 (69-101)		17 (7-36)		14 (6-30)		5 (2-11)		4 (2-9)	
2005	Mizoram	117 (83-122)		94 (67-98)		17 (7-36)		14 (6-29)		5 (2-11)		4 (2-9)	
2006	Mizoram	116 (83-121)		92 (65-96)		17 (7-36)		13 (6-28)		5 (2-11)		4 (2-9)	
2007	Mizoram	116 (82-121)		90 (64-94)		17 (7-35)		13 (6-27)		5 (2-11)		4 (2-9)	
2008	Mizoram	115 (81-120)		88 (62-92)		17 (7-35)		13 (5-27)		5 (2-11)		4 (2-8)	
2009	Mizoram	114 (80-118)		86 (61-90)		17 (7-34)		13 (5-26)		5 (2-11)		4 (2-8)	
2010	Mizoram	112 (79-116)		84 (59-87)		17 (7-34)		12 (5-25)		5 (2-11)		4 (2-8)	
2011	Mizoram	112 (79-117)		83 (59-86)		17 (7-34)		12 (5-25)		5 (2-11)		4 (2-8)	
2012	Mizoram	110 (78-115)		81 (58-85)		16 (7-33)		12 (5-24)		5 (2-10)		4 (2-8)	
2013	Mizoram	110 (78-115)		80 (57-84)		16 (7-33)		12 (5-24)		5 (2-10)		4 (2-8)	
2014	Mizoram	108 (77-113)		79 (56-82)		16 (7-32)		12 (5-24)		5 (2-10)		4 (2-7)	
2015	Mizoram	106 (75-110)	1 (1-2)	76 (54-79)	1 (1-1)	16 (6-33)	0 (0-0)	11 (4-23)	0 (0-0)	14 (6-29)	0 (0-0)	10 (4-21)	0 (0-0)
2000	Nagaland	84 (60-88)		39 (28-41)		12 (4-27)		6 (2-13)		11 (4-24)		5 (2-11)	
2001	Nagaland	89 (63-93)		42 (29-43)		13 (4-28)		6 (2-13)		11 (4-25)		5 (2-12)	
2002	Nagaland	95 (68-99)		44 (31-46)		14 (5-30)		6 (2-14)		12 (4-27)		6 (2-12)	
2003	Nagaland	102 (72-106)		47 (33-49)		15 (5-32)		7 (2-15)		13 (5-29)		6 (2-13)	
2004	Nagaland	108 (76-112)		49 (35-51)		15 (5-34)		7 (2-16)		14 (5-30)		6 (2-14)	
2005	Nagaland	112 (79-117)		51 (36-53)		16 (5-36)		7 (2-16)		14 (5-32)		6 (2-14)	
2006	Nagaland	114 (81-119)		52 (37-54)		16 (5-37)		7 (2-17)		14 (5-33)		7 (2-15)	
2007	Nagaland	116 (82-121)		53 (37-55)		16 (5-37)		7 (2-17)		15 (5-33)		7 (2-15)	
2008	Nagaland	117 (83-122)		53 (38-55)		16 (5-38)		7 (2-17)		15 (5-34)		7 (2-15)	
2009	Nagaland	115 (81-120)		52 (37-54)		16 (5-37)		7 (2-17)		14 (4-33)		6 (2-15)	
2010	Nagaland	119 (85-124)		54 (38-56)		17 (5-39)		8 (2-18)		15 (4-35)		7 (2-16)	
2011	Nagaland	119 (84-124)		54 (38-56)		16 (5-39)		7 (2-18)		15 (4-35)		7 (2-16)	
2012	Nagaland	116 (82-121)		53 (38-55)		16 (5-38)		7 (2-17)		14 (4-34)		7 (2-16)	
2013	Nagaland	112 (80-117)		52 (37-54)		16 (4-37)		7 (2-17)		14 (4-33)		6 (2-15)	
2014	Nagaland	106 (75-110)		49 (35-51)		15 (4-35)		7 (2-16)		13 (4-31)		6 (2-14)	
2015	Nagaland	99 (70-103)	1 (1-1)	46 (33-48)	1 (0-1)	14 (4-33)	0 (0-0)	6 (2-15)	0 (0-0)	12 (3-29)	0 (0-0)	6 (2-14)	0 (0-0)
2000	Odisha	6170 (4375-6430)		152 (108-158)		932 (423-1838)		23 (10-45)		293 (133-578)		7 (3-14)	
2001	Odisha	5830 (4134-6076)		142 (101-148)		881 (400-1737)		22 (10-42)		277 (126-546)		7 (3-13)	
2002	Odisha	5488 (3891-5719)		133 (95-139)		829 (376-1635)		20 (9-40)		261 (118-514)		6 (3-13)	
2003	Odisha	5182 (3674-5400)		126 (89-131)		782 (355-1544)		19 (9-37)		246 (112-485)		6 (3-12)	
2004	Odisha	4878 (3459-5084)		118 (84-123)		736 (334-1453)		18 (8-35)		231 (105-457)		6 (3-11)	
2005	Odisha	4592 (3256-4786)		111 (79-116)		692 (312-1371)		17 (8-33)		218 (98-431)		5 (2-10)	
2006	Odisha	4318 (3062-4500)		104 (74-109)		650 (291-1291)		16 (7-31)		204 (92-406)		5 (2-10)	
2007	Odisha	4021 (2851-4190)		97 (69-101)		604 (269-1204)		15 (6-29)		190 (84-379)		5 (2-9)	
2008	Odisha	3746 (2656-3904)		91 (64-95)		562 (250-1122)		14 (6-27)		177 (79-353)		4 (2-9)	
2009	Odisha	3462 (2455-3608)		84 (60-88)		518 (231-1037)		13 (6-25)		163 (73-326)		4 (2-8)	
2010	Odisha	3198 (2268-3333)		78 (55-81)		477 (214-958)		12 (5-23)		150 (67-301)		4 (2-7)	
2011	Odisha	2967 (2104-3092)		73 (52-76)		441 (173-914)		11 (4-22)		392 (154-814)		10 (4-20)	
2012	Odisha	2725 (1932-2840)		68 (48-70)		404 (159-839)		10 (4-21)		359 (142-747)		9 (4-19)	
2013	Odisha	2524 (1790-2631)		63 (45-66)		373 (147-778)		9 (4-19)		332 (131-692)		8 (3-17)	
2014	Odisha	2306 (1635-2403)		58 (41-61)		340 (135-710)		9 (3-18)		303 (120-632)		8 (3-16)	
2015	Odisha	2109 (1495-2198)	30 (21-31)	54 (38-56)	1 (1-1)	311 (123-650)	5 (2-10)	8 (3-17)	0 (0-0)	277 (110-578)	4 (2-9)	7 (3-15)	0 (0-0)
2000	Punjab	1877 (1331-1956)		77 (55-81)		305 (160-539)		13 (7-22)		272 (142-480)		11 (6-20)	
2001	Punjab	1752 (1242-1826)		72 (51-75)		285 (149-503)		12 (6-21)		254 (133-448)		10 (5-18)	
2002	Punjab	1631 (1156-1699)		67 (47-70)		265 (139-468)		11 (6-19)		236 (124-417)		10 (5-17)	
2003	Punjab	1516 (1075-1580)		62 (44-65)		247 (129-435)		10 (5-18)		220 (115-388)		9 (5-16)	
2004	Punjab	1405 (996-1464)		58 (41-60)		230 (120-404)		9 (5-17)		205 (107-359)		8 (4-15)	
2005	Punjab	1299 (921-1354)		53 (38-56)		215 (115-370)		9 (5-15)		191 (103-330)		8 (4-14)	
2006	Punjab	1197 (849-1248)		49 (35-51)		200 (111-339)		8 (5-14)		178 (99-301)		7 (4-12)	
2007	Punjab	1098 (779-1144)		45 (32-47)		185 (101-308)		8 (4-13)		164 (90-274)		7 (4-11)	
2008	Punjab	1004 (712-1047)		41 (29-43)		170 (93-282)		7 (4-12)		151 (82-251)		6 (3-10)	
2009	Punjab	912 (647-950)		38 (27-39)		154 (84-256)		6 (3-11)		138 (75-228)		6 (3-9)	

Spn = *Streptococcus pneumoniae*; npnm = non-pneumonia, non-meningitis; HIV = HIV-infected cases only; UR = uncertainty range

Year	State	Spn pneumonia deaths (UR)	Spn pneumonia deaths; HIV (UR)	Spn pneumonia mortality rate (UR)	Spn pneumonia mortality rate; HIV (UR)	Spn meningitis deaths (UR)	Spn meningitis deaths; HIV (UR)	Spn meningitis mortality rate (UR)	Spn meningitis mortality rate; HIV (UR)	Spn NPNM deaths (UR)	Spn NPNM deaths; HIV (UR)	Spn NPNM mortality rate (UR)	Spn NPNM mortality rate; HIV (UR)
2010	Punjab	827 (586-862)		35 (24-36)		140 (76-232)		6 (3-10)		125 (68-207)		5 (3-9)	
2011	Punjab	752 (533-784)		32 (22-33)		128 (69-211)		5 (3-9)		114 (62-188)		5 (3-8)	
2012	Punjab	679 (482-708)		29 (20-30)		116 (63-191)		5 (3-8)		103 (56-170)		4 (2-7)	
2013	Punjab	624 (443-650)		27 (19-28)		108 (58-176)		5 (2-8)		96 (51-156)		4 (2-7)	
2014	Punjab	574 (407-598)		25 (18-26)		101 (56-192)		4 (2-8)		89 (50-171)		4 (2-7)	
2015	Punjab	540 (383-563)	8 (5-8)	24 (17-25)	0 (0-0)	96 (53-180)	1 (1-3)	4 (2-8)	0 (0-0)	85 (47-160)	1 (1-2)	4 (2-7)	0 (0-0)
2000	Rajasthan	11109 (7877-11577)		135 (96-141)		1689 (779-3294)		21 (9-40)		531 (245-1036)		6 (3-13)	
2001	Rajasthan	10645 (7549-11094)		129 (91-134)		1618 (746-3157)		20 (9-38)		509 (235-993)		6 (3-12)	
2002	Rajasthan	10170 (7212-10599)		122 (87-128)		1547 (713-3016)		19 (9-36)		486 (224-948)		6 (3-11)	
2003	Rajasthan	9640 (6836-10047)		116 (82-121)		1468 (676-2859)		18 (8-34)		462 (213-899)		6 (3-11)	
2004	Rajasthan	9091 (6447-9475)		109 (78-114)		1388 (637-2696)		17 (8-32)		436 (200-848)		5 (2-10)	
2005	Rajasthan	8532 (6050-8892)		103 (73-107)		1308 (612-2518)		16 (7-30)		411 (193-792)		5 (2-10)	
2006	Rajasthan	7971 (5652-8307)		96 (68-100)		1226 (586-2341)		15 (7-28)		385 (184-736)		5 (2-9)	
2007	Rajasthan	7405 (5251-7717)		89 (63-93)		1141 (558-2163)		14 (7-26)		359 (175-680)		4 (2-8)	
2008	Rajasthan	6870 (4872-7160)		83 (59-86)		1058 (517-2007)		13 (6-24)		333 (163-631)		4 (2-8)	
2009	Rajasthan	6350 (4503-6618)		77 (55-80)		975 (429-1904)		12 (5-23)		868 (382-1695)		11 (5-21)	
2010	Rajasthan	5854 (4151-6101)		71 (51-74)		896 (396-1756)		11 (5-21)		798 (352-1563)		10 (4-19)	
2011	Rajasthan	5389 (3821-5616)		66 (47-69)		823 (364-1616)		10 (4-20)		733 (324-1439)		9 (4-18)	
2012	Rajasthan	4899 (3474-5105)		61 (43-63)		747 (331-1469)		9 (4-18)		665 (295-1308)		8 (4-16)	
2013	Rajasthan	4514 (3201-4704)		57 (40-59)		688 (305-1354)		9 (4-17)		613 (272-1205)		8 (3-15)	
2014	Rajasthan	4161 (2951-4337)		53 (37-55)		635 (281-1248)		8 (4-16)		565 (250-1111)		7 (3-14)	
2015	Rajasthan	3912 (2774-4077)	55 (39-57)	50 (35-52)	1 (0-1)	597 (264-1173)	9 (4-18)	8 (3-15)	0 (0-0)	531 (235-1044)	8 (4-16)	7 (3-13)	0 (0-0)
2000	Sikkim	31 (22-33)		51 (36-54)		5 (2-9)		8 (4-15)		4 (2-8)		7 (3-14)	
2001	Sikkim	29 (21-31)		49 (35-51)		5 (2-9)		8 (3-15)		4 (2-8)		7 (3-13)	
2002	Sikkim	27 (19-28)		46 (33-48)		4 (2-8)		7 (3-14)		4 (2-7)		6 (3-12)	
2003	Sikkim	25 (18-26)		43 (31-45)		4 (2-7)		7 (3-13)		3 (2-7)		6 (3-12)	
2004	Sikkim	23 (16-24)		41 (29-42)		3 (2-7)		6 (3-12)		3 (1-6)		5 (3-11)	
2005	Sikkim	21 (15-22)		38 (27-40)		3 (1-6)		6 (2-12)		3 (1-6)		5 (2-10)	
2006	Sikkim	19 (14-20)		36 (25-37)		3 (1-6)		5 (2-11)		3 (1-5)		5 (2-10)	
2007	Sikkim	18 (12-18)		34 (24-35)		3 (1-6)		5 (2-11)		2 (1-5)		4 (1-9)	
2008	Sikkim	16 (12-17)		32 (23-33)		2 (1-5)		5 (2-10)		2 (1-5)		4 (1-9)	
2009	Sikkim	16 (11-16)		31 (22-32)		2 (1-5)		5 (2-10)		2 (1-4)		4 (1-9)	
2010	Sikkim	15 (11-16)		31 (22-32)		2 (1-5)		5 (2-10)		2 (1-4)		4 (2-8)	
2011	Sikkim	14 (10-15)		31 (22-32)		2 (1-4)		5 (2-9)		2 (1-4)		4 (2-8)	
2012	Sikkim	14 (10-14)		30 (21-32)		2 (1-4)		5 (2-9)		2 (1-4)		4 (2-8)	
2013	Sikkim	14 (10-14)		31 (22-32)		2 (1-5)		5 (2-11)		2 (1-4)		4 (1-10)	
2014	Sikkim	13 (9-14)		31 (22-32)		2 (1-5)		5 (2-11)		2 (1-4)		4 (1-10)	
2015	Sikkim	13 (9-14)	0 (0-0)	31 (22-33)	0 (0-0)	2 (1-5)	0 (0-0)	5 (2-11)	0 (0-0)	2 (1-4)	0 (0-0)	4 (1-10)	0 (0-0)
2000	Tamil Nadu	2795 (1982-2913)		48 (34-50)		431 (198-830)		7 (3-14)		384 (177-739)		7 (3-13)	
2001	Tamil Nadu	2657 (1884-2769)		46 (32-47)		410 (189-790)		7 (3-14)		365 (168-703)		6 (3-12)	
2002	Tamil Nadu	2514 (1783-2620)		43 (30-45)		388 (178-747)		7 (3-13)		346 (159-665)		6 (3-11)	
2003	Tamil Nadu	2369 (1680-2469)		40 (29-42)		367 (168-704)		6 (3-12)		326 (150-627)		6 (3-11)	
2004	Tamil Nadu	2220 (1574-2313)		38 (27-39)		345 (158-660)		6 (3-11)		307 (140-587)		5 (2-10)	
2005	Tamil Nadu	2067 (1465-2154)		35 (25-36)		323 (153-610)		5 (3-10)		288 (136-543)		5 (2-9)	
2006	Tamil Nadu	1915 (1358-1996)		32 (23-34)		301 (147-561)		5 (2-9)		268 (131-499)		5 (2-8)	
2007	Tamil Nadu	1762 (1250-1837)		30 (21-31)		279 (141-512)		5 (2-9)		248 (126-456)		4 (2-8)	
2008	Tamil Nadu	1623 (1151-1691)		27 (19-28)		257 (130-471)		4 (2-8)		229 (116-420)		4 (2-7)	
2009	Tamil Nadu	1491 (1057-1554)		25 (18-26)		236 (116-436)		4 (2-7)		210 (103-388)		4 (2-7)	
2010	Tamil Nadu	1369 (971-1427)		23 (16-24)		216 (103-402)		4 (2-7)		193 (92-358)		3 (2-6)	
2011	Tamil Nadu	1258 (892-1311)		21 (15-22)		198 (77-448)		3 (1-8)		177 (68-399)		3 (1-7)	
2012	Tamil Nadu	1145 (812-1193)		20 (14-20)		180 (67-410)		3 (1-7)		160 (59-365)		3 (1-6)	
2013	Tamil Nadu	1055 (748-1099)		18 (13-19)		166 (62-377)		3 (1-7)		148 (55-336)		3 (1-6)	
2014	Tamil Nadu	972 (689-1013)		17 (12-18)		153 (57-348)		3 (1-6)		136 (50-310)		2 (1-5)	
2015	Tamil Nadu	926 (656-965)	13 (9-14)	16 (11-17)	0 (0-0)	145 (54-331)	2 (1-5)	3 (1-6)	0 (0-0)	129 (48-295)	2 (1-4)	2 (1-5)	0 (0-0)
2000	Tripura	160 (113-167)		48 (34-50)		24 (9-49)		7 (3-15)		21 (8-44)		6 (2-13)	
2001	Tripura	144 (102-150)		43 (30-45)		21 (8-45)		6 (2-13)		19 (7-40)		6 (2-12)	
2002	Tripura	131 (93-137)		38 (27-40)		19 (8-41)		6 (2-12)		17 (7-36)		5 (2-11)	
2003	Tripura	119 (85-124)		35 (25-36)		18 (7-37)		5 (2-11)		16 (6-33)		5 (2-10)	
2004	Tripura	108 (77-113)		31 (22-33)		16 (6-34)		5 (2-10)		14 (6-30)		4 (2-9)	
2005	Tripura	100 (71-104)		29 (20-30)		15 (6-31)		4 (2-9)		13 (5-27)		4 (2-8)	
2006	Tripura	92 (65-96)		26 (19-27)		14 (6-28)		4 (2-8)		12 (5-25)		3 (1-7)	
2007	Tripura	87 (61-90)		24 (17-25)		13 (6-26)		4 (2-7)		12 (5-23)		3 (1-7)	
2008	Tripura	80 (57-84)		22 (16-23)		12 (5-24)		3 (1-7)		11 (5-22)		3 (1-6)	
2009	Tripura	75 (53-78)		21 (15-22)		12 (5-22)		3 (1-6)		10 (5-20)		3 (1-6)	
2010	Tripura	71 (50-74)		20 (14-20)		11 (5-21)		3 (1-6)		10 (5-18)		3 (1-5)	
2011	Tripura	66 (47-69)		18 (13-19)		11 (5-19)		3 (1-5)		9 (5-17)		3 (1-5)	
2012	Tripura	60 (43-63)		17 (12-18)		10 (5-17)		3 (1-5)		9 (5-15)		2 (1-4)	
2013	Tripura	54 (38-57)		15 (11-16)		9 (5-16)		2 (1-4)		8 (4-14)		2 (1-4)	
2014	Tripura	47 (34-49)		13 (9-14)		8 (4-16)		2 (1-5)		7 (3-14)		2 (1-4)	
2015	Tripura	41 (29-42)	1 (0-1)	11 (8-12)	0 (0-0)	7 (3-14)	0 (0-0)	2 (1-4)	0 (0-0)	6 (3-12)	0 (0-0)	2 (1-3)	0 (0-0)
2000	Union Territory	105 (75-110)		38 (27-40)		17 (8-32)		6 (3-12)		15 (7-29)		5 (3-10)	
2001	Union Territory	100 (71-104)		36 (25-37)		16 (8-31)		6 (3-11)		14 (7-27)		5 (2-10)	
2002	Union Territory	95 (67-99)		33 (24-35)		15 (7-29)		5 (3-10)		14 (6-26)		5 (2-9)	
2003	Union Territory	91 (64-95)		32 (23-33)		15 (7-29)		5 (2-10)		13 (6-26)		5 (2-9)	
2004	Union Territory	87 (62-91)		30 (21-31)		14 (6-28)		5 (2-10)		13 (6-25)		4 (2-9)	
2005	Union Territory	82 (58-86)		28 (20-30)		14 (6-26)		5 (2-9)		12 (6-23)		4 (2-8)	
2006	Union Territory	78 (55-82)		27 (19-28)		13 (7-25)		5 (2-8)		12 (6-22)		4 (2-7)	
2007	Union Territory	74 (53-77)		25 (18-26)		13 (7-23)		4 (3-8)		12 (7-21)		4 (2-7)	
2008	Union Territory	71 (51-74)		24 (17-25)		13 (7-22)		4 (2-8)		11 (6-20)		4 (2-7)	
2009	Union Territory	68 (48-70)		23 (16-24)		12 (7-21)		4 (2-7)		11 (6-19)		4 (2-6)	
2010	Union Territory	65 (46-68)		22 (16-23)		12 (6-20)		4 (2-7)		11 (6-18)		4 (2-6)	
2011	Union Territory	63 (45-66)		21 (15-22)		12 (6-20)		4 (2-7)		10 (6-18)		4 (2-6)	
2012	Union Territory	60 (43-62)		20 (14-21)		11 (6-20)		4 (2-7)		10 (5-17)		3 (2-6)	
2013	Union Territory	58 (41-60)		20 (14-21)		11 (5-19)		4 (2-6)		9 (5-17)		3 (2-6)	
2014	Union Territory	54 (38-57)		19 (13-19)		10 (5-18)		3 (2-6)		9 (4-16)		3 (2-5)	
2015	Union Territory	50 (36-53)	1 (1-1)	17 (12-18)	0 (0-0)	9 (5-16)	0 (0-0)	3 (2-6)	0 (0-0)	8 (4-14)	0 (0-0)	3 (1-5)	0 (0-0)
2000	Uttar Pradesh	39399 (27938-41059)		167 (118-174)		6218 (3122-11386)		26 (13-48)		1955 (981-3580)		8 (4-15)	

Spn = *Streptococcus pneumoniae*; npnm = non-pneumonia, non-meningitis; HIV = HIV-infected cases only; UR = uncertainty range

Year	State	Spn pneumonia deaths (UR)	Spn pneumonia deaths; HIV (UR)	Spn pneumonia mortality rate (UR)	Spn pneumonia mortality rate; HIV (UR)	Spn meningitis deaths (UR)	Spn meningitis deaths; HIV (UR)	Spn meningitis mortality rate (UR)	Spn meningitis mortality rate; HIV (UR)	Spn NPNM deaths (UR)	Spn NPNM deaths; HIV (UR)	Spn NPNM mortality rate (UR)	Spn NPNM mortality rate; HIV (UR)
2001	Uttar Pradesh	37800 (26804-39394)		159 (113-166)		5966 (2995-10924)		25 (13-46)		1876 (942-3434)		8 (4-14)	
2002	Uttar Pradesh	36166 (25645-37690)		152 (108-159)		5707 (2866-10451)		24 (12-44)		1794 (901-3286)		8 (4-14)	
2003	Uttar Pradesh	34518 (24477-35973)		146 (103-152)		5442 (2735-9975)		23 (12-42)		1711 (860-3136)		7 (4-13)	
2004	Uttar Pradesh	32809 (23265-34192)		139 (98-145)		5165 (2600-9481)		22 (11-40)		1624 (817-2981)		7 (3-13)	
2005	Uttar Pradesh	31019 (21996-32327)		132 (93-137)		4871 (2426-8989)		21 (10-38)		1532 (763-2826)		6 (3-12)	
2006	Uttar Pradesh	29257 (20746-30490)		124 (88-130)		4583 (2260-8501)		19 (10-36)		1441 (710-2673)		6 (3-11)	
2007	Uttar Pradesh	27438 (19456-28595)		117 (83-122)		4288 (2093-7994)		18 (9-34)		1348 (658-2513)		6 (3-11)	
2008	Uttar Pradesh	25658 (18194-26739)		110 (78-115)		4000 (1957-7475)		17 (8-32)		1258 (615-2350)		5 (3-10)	
2009	Uttar Pradesh	23907 (16952-24914)		103 (73-108)		3716 (1823-6965)		16 (8-30)		1168 (573-2190)		5 (2-9)	
2010	Uttar Pradesh	22191 (15735-23126)		97 (69-101)		3438 (1693-6465)		15 (7-28)		1081 (532-2033)		5 (2-9)	
2011	Uttar Pradesh	20556 (14576-21423)		91 (64-94)		3170 (1411-6146)		14 (6-27)		2822 (1256-5471)		12 (6-24)	
2012	Uttar Pradesh	18786 (13321-19578)		84 (59-87)		2887 (1290-5617)		13 (6-25)		2570 (1148-5000)		11 (5-22)	
2013	Uttar Pradesh	17334 (12292-18065)		78 (56-82)		2658 (1190-5182)		12 (5-23)		2366 (1060-4614)		11 (5-21)	
2014	Uttar Pradesh	15889 (11267-16558)		73 (52-76)		2433 (1091-4750)		11 (5-22)		2166 (971-4229)		10 (4-19)	
2015	Uttar Pradesh	14665 (10399-15283)	206 (146-214)	68 (48-71)	1 (1-1)	2245 (1007-4384)	33 (15-65)	10 (5-20)	0 (0-0)	1999 (896-3903)	30 (13-58)	9 (4-18)	0 (0-0)
2000	Uttarakhand	879 (624-917)		84 (59-87)		138 (69-254)		13 (7-24)		43 (22-80)		4 (2-8)	
2001	Uttarakhand	842 (597-877)		80 (57-83)		132 (66-244)		13 (6-23)		42 (21-77)		4 (2-7)	
2002	Uttarakhand	804 (570-837)		76 (54-79)		126 (63-232)		12 (6-22)		39 (20-73)		4 (2-7)	
2003	Uttarakhand	765 (543-797)		72 (51-75)		119 (55-227)		11 (5-21)		106 (49-202)		10 (5-19)	
2004	Uttarakhand	726 (515-756)		69 (49-71)		112 (52-215)		11 (5-20)		99 (46-192)		9 (4-18)	
2005	Uttarakhand	685 (486-714)		65 (46-68)		104 (46-206)		10 (4-19)		93 (41-183)		9 (4-17)	
2006	Uttarakhand	646 (458-673)		61 (43-64)		97 (41-196)		9 (4-19)		87 (37-174)		8 (3-16)	
2007	Uttarakhand	606 (429-631)		57 (41-60)		91 (37-185)		9 (3-18)		81 (33-165)		8 (3-16)	
2008	Uttarakhand	567 (402-591)		54 (38-56)		85 (34-173)		8 (3-17)		75 (30-154)		7 (3-15)	
2009	Uttarakhand	526 (373-549)		50 (36-52)		78 (32-161)		7 (3-15)		70 (28-143)		7 (3-14)	
2010	Uttarakhand	485 (344-505)		47 (33-49)		72 (29-148)		7 (3-14)		64 (26-132)		6 (3-13)	
2011	Uttarakhand	441 (313-460)		43 (30-45)		66 (27-135)		6 (3-13)		58 (24-120)		6 (2-12)	
2012	Uttarakhand	392 (278-408)		38 (27-40)		58 (24-120)		6 (2-12)		52 (21-107)		5 (2-10)	
2013	Uttarakhand	348 (247-363)		35 (25-36)		52 (21-107)		5 (2-11)		46 (19-95)		5 (2-9)	
2014	Uttarakhand	307 (218-320)		31 (22-32)		46 (19-94)		5 (2-9)		41 (16-84)		4 (2-8)	
2015	Uttarakhand	272 (193-283)	4 (3-4)	27 (19-29)	0 (0-0)	40 (16-83)	1 (0-1)	4 (2-8)	0 (0-0)	36 (15-74)	1 (0-1)	4 (1-7)	0 (0-0)
2000	West Bengal	6561 (4652-6837)		75 (53-79)		1024 (485-1934)		12 (6-22)		912 (432-1722)		10 (5-20)	
2001	West Bengal	5984 (4243-6236)		69 (49-72)		934 (443-1764)		11 (5-20)		831 (394-1571)		10 (5-18)	
2002	West Bengal	5377 (3813-5604)		62 (44-64)		839 (398-1586)		10 (5-18)		747 (354-1412)		9 (4-16)	
2003	West Bengal	4845 (3435-5049)		56 (40-58)		757 (358-1429)		9 (4-16)		674 (319-1272)		8 (4-15)	
2004	West Bengal	4398 (3119-4584)		51 (36-53)		687 (325-1297)		8 (4-15)		612 (290-1155)		7 (3-13)	
2005	West Bengal	4015 (2847-4184)		47 (33-49)		628 (300-1182)		7 (3-14)		559 (267-1052)		7 (3-12)	
2006	West Bengal	3670 (2602-3824)		43 (30-45)		575 (276-1079)		7 (3-13)		512 (246-960)		6 (3-11)	
2007	West Bengal	3348 (2374-3489)		39 (28-41)		526 (254-982)		6 (3-12)		468 (226-875)		6 (3-10)	
2008	West Bengal	3023 (2144-3151)		36 (25-37)		476 (229-887)		6 (3-11)		424 (204-790)		5 (2-9)	
2009	West Bengal	2709 (1921-2823)		32 (23-34)		427 (208-793)		5 (2-9)		380 (185-706)		5 (2-8)	
2010	West Bengal	2502 (1774-2608)		30 (21-32)		396 (194-731)		5 (2-9)		352 (173-651)		4 (2-8)	
2011	West Bengal	2388 (1694-2489)		29 (21-30)		378 (187-697)		5 (2-9)		337 (167-620)		4 (2-8)	
2012	West Bengal	2284 (1619-2380)		28 (20-30)		363 (181-665)		5 (2-8)		323 (161-592)		4 (2-7)	
2013	West Bengal	2261 (1603-2356)		29 (20-30)		359 (179-658)		5 (2-8)		320 (159-586)		4 (2-7)	
2014	West Bengal	2255 (1599-2350)		29 (20-30)		359 (179-656)		5 (2-8)		319 (159-584)		4 (2-7)	
2015	West Bengal	2280 (1617-2376)	32 (23-33)	30 (21-31)	0 (0-0)	363 (181-664)	5 (3-10)	5 (2-9)	0 (0-0)	323 (161-591)	5 (2-9)	4 (2-8)	0 (0-0)

Spn = *Streptococcus pneumoniae*; npnm = non-pneumonia, non-meningitis; HIV = HIV-infected cases only; UR = uncertainty range

Webappendix 10: Hib deaths in India by state, year, and HIV status

		Pneumonia deaths				Meningitis deaths				NPNM deaths			
Year	State	Hib pneumonia deaths (UR)	Hib pneumonia deaths; HIV (UR)	Hib pneumonia mortality rate (UR)	Hib pneumonia mortality rate; HIV (UR)	Hib meningitis deaths (UR)	Hib meningitis deaths; HIV (UR)	Hib meningitis mortality rate (UR)	Hib mortality rate; HIV (UR)	Hib NPNM deaths (UR)	Hib NPNM deaths; HIV (UR)	Hib NPNM mortality rate (UR)	Hib NPNM mortality rate; HIV (UR)
2000	Andhra Pradesh	2768 (1947-3636)		36 (25-47)		942 (441-1396)		12 (6-18)		4 (2-6)		0 (0-0)	
2001	Andhra Pradesh	2714 (1909-3566)		35 (25-47)		924 (405-1396)		12 (5-18)		7 (3-11)		0 (0-0)	
2002	Andhra Pradesh	2639 (1856-3468)		35 (24-45)		898 (394-1357)		12 (5-18)		7 (3-11)		0 (0-0)	
2003	Andhra Pradesh	2551 (1794-3352)		34 (24-44)		868 (380-1312)		11 (5-17)		7 (3-10)		0 (0-0)	
2004	Andhra Pradesh	2447 (1721-3215)		33 (23-43)		833 (365-1258)		11 (5-17)		7 (3-10)		0 (0-0)	
2005	Andhra Pradesh	2327 (1636-3057)		31 (22-41)		792 (346-1198)		11 (5-16)		6 (3-10)		0 (0-0)	
2006	Andhra Pradesh	2206 (1552-2899)		30 (21-39)		752 (328-1137)		10 (4-15)		6 (3-9)		0 (0-0)	
2007	Andhra Pradesh	2065 (1452-2713)		28 (20-37)		704 (306-1065)		10 (4-14)		6 (2-8)		0 (0-0)	
2008	Andhra Pradesh	1924 (1353-2528)		26 (19-35)		657 (285-992)		9 (4-14)		5 (2-8)		0 (0-0)	
2009	Andhra Pradesh	1776 (1249-2334)		25 (17-32)		608 (259-924)		8 (4-13)		5 (2-7)		0 (0-0)	
2010	Andhra Pradesh	1616 (1137-2124)		23 (16-30)		554 (231-848)		8 (3-12)		4 (2-7)		0 (0-0)	
2011	Andhra Pradesh	1455 (1023-1912)		21 (15-27)		500 (204-769)		7 (3-11)		4 (2-6)		0 (0-0)	
2012	Andhra Pradesh	1291 (908-1697)		19 (13-25)		444 (178-688)		6 (3-10)		4 (1-5)		0 (0-0)	
2013	Andhra Pradesh	1158 (814-1521)		17 (12-23)		399 (160-617)		6 (2-9)		3 (1-5)		0 (0-0)	
2014	Andhra Pradesh	1042 (733-1369)		16 (11-21)		359 (144-555)		5 (2-8)		3 (1-4)		0 (0-0)	
2015	Andhra Pradesh	404 (284-530)	1 (1-1)	6 (4-8)	0 (0-0)	139 (56-215)	0 (0-1)	2 (1-3)	0 (0-0)	1 (0-2)	0 (0-0)	0 (0-0)	0 (0-0)
2000	Arunachal Pradesh	33 (23-43)		21 (15-27)		11 (4-18)		7 (2-12)		0 (0-0)		0 (0-0)	
2001	Arunachal Pradesh	31 (22-40)		19 (14-26)		11 (4-17)		7 (2-11)		0 (0-0)		0 (0-0)	
2002	Arunachal Pradesh	31 (22-40)		19 (14-25)		11 (4-17)		7 (2-11)		0 (0-0)		0 (0-0)	
2003	Arunachal Pradesh	31 (22-41)		20 (14-26)		11 (4-18)		7 (2-11)		0 (0-0)		0 (0-0)	
2004	Arunachal Pradesh	32 (22-41)		20 (14-26)		11 (4-18)		7 (2-11)		0 (0-0)		0 (0-0)	
2005	Arunachal Pradesh	32 (22-41)		20 (14-26)		11 (4-17)		7 (2-11)		0 (0-0)		0 (0-0)	
2006	Arunachal Pradesh	33 (23-44)		21 (15-27)		12 (5-18)		7 (3-11)		0 (0-0)		0 (0-0)	
2007	Arunachal Pradesh	32 (23-42)		20 (14-26)		11 (5-16)		7 (3-10)		0 (0-0)		0 (0-0)	
2008	Arunachal Pradesh	33 (23-43)		20 (14-27)		11 (5-17)		7 (3-10)		0 (0-0)		0 (0-0)	
2009	Arunachal Pradesh	33 (23-44)		21 (15-27)		12 (5-18)		7 (3-11)		0 (0-0)		0 (0-0)	
2010	Arunachal Pradesh	34 (24-44)		21 (15-28)		12 (4-18)		7 (3-11)		0 (0-0)		0 (0-0)	
2011	Arunachal Pradesh	33 (23-43)		21 (15-27)		12 (4-18)		7 (3-12)		0 (0-0)		0 (0-0)	
2012	Arunachal Pradesh	32 (23-42)		20 (14-27)		11 (4-18)		7 (2-12)		0 (0-0)		0 (0-0)	
2013	Arunachal Pradesh	32 (22-42)		20 (14-27)		11 (4-18)		7 (2-11)		0 (0-0)		0 (0-0)	
2014	Arunachal Pradesh	31 (22-41)		20 (14-26)		11 (4-18)		7 (2-11)		0 (0-0)		0 (0-0)	
2015	Arunachal Pradesh	28 (20-37)	0 (0-0)	18 (13-24)	0 (0-0)	10 (3-16)	0 (0-0)	6 (2-10)	0 (0-0)	0 (0-0)	0 (0-0)	0 (0-0)	0 (0-0)
2000	Assam	1754 (1234-2305)		51 (36-67)		612 (235-947)		18 (7-28)		2 (1-4)		0 (0-0)	
2001	Assam	1700 (1195-2233)		49 (34-64)		593 (228-917)		17 (7-26)		2 (1-4)		0 (0-0)	
2002	Assam	1662 (1169-2183)		47 (33-62)		580 (223-897)		17 (6-26)		2 (1-4)		0 (0-0)	
2003	Assam	1643 (1156-2159)		47 (33-61)		573 (220-887)		16 (6-25)		2 (1-4)		0 (0-0)	
2004	Assam	1638 (1152-2153)		46 (33-61)		571 (220-884)		16 (6-25)		2 (1-4)		0 (0-0)	
2005	Assam	1637 (1152-2152)		46 (32-61)		570 (222-880)		16 (6-25)		2 (1-4)		0 (0-0)	
2006	Assam	1639 (1153-2153)		46 (32-60)		570 (225-877)		16 (6-25)		2 (1-4)		0 (0-0)	
2007	Assam	1643 (1155-2158)		46 (32-60)		571 (228-876)		16 (6-24)		2 (1-4)		0 (0-0)	
2008	Assam	1650 (1160-2167)		46 (32-60)		573 (229-879)		16 (6-24)		2 (1-4)		0 (0-0)	
2009	Assam	1646 (1158-2162)		46 (32-60)		572 (229-877)		16 (6-24)		2 (1-4)		0 (0-0)	
2010	Assam	1644 (1157-2161)		46 (32-60)		571 (229-877)		16 (6-24)		2 (1-4)		0 (0-0)	
2011	Assam	1640 (1154-2155)		46 (32-60)		570 (228-874)		16 (6-24)		2 (1-4)		0 (0-0)	
2012	Assam	1625 (1143-2135)		46 (32-60)		565 (226-866)		16 (6-24)		2 (1-4)		0 (0-0)	
2013	Assam	1632 (1148-2144)		46 (32-61)		567 (227-870)		16 (6-25)		2 (1-4)		0 (0-0)	
2014	Assam	1621 (1140-2130)		46 (32-61)		563 (225-864)		16 (6-25)		2 (1-4)		0 (0-0)	
2015	Assam	568 (400-747)	1 (1-2)	16 (11-21)	0 (0-0)	198 (72-312)	1 (0-1)	6 (2-9)	0 (0-0)	2 (1-2)	0 (0-0)	0 (0-0)	0 (0-0)
2000	Bihar	6762 (4756-8884)		55 (39-72)		2296 (1084-3403)		19 (9-28)		9 (4-14)		0 (0-0)	
2001	Bihar	6647 (4675-8733)		53 (37-69)		2257 (1066-3345)		18 (8-27)		9 (4-14)		0 (0-0)	
2002	Bihar	6495 (4568-8534)		51 (36-67)		2206 (1041-3268)		17 (8-25)		9 (4-13)		0 (0-0)	
2003	Bihar	6329 (4452-8316)		49 (34-64)		2150 (1015-3185)		17 (8-24)		9 (4-13)		0 (0-0)	
2004	Bihar	6145 (4322-8074)		47 (33-61)		2089 (985-3092)		16 (7-23)		9 (4-13)		0 (0-0)	
2005	Bihar	5947 (4183-7814)		44 (31-58)		2024 (946-3003)		15 (7-22)		8 (4-12)		0 (0-0)	
2006	Bihar	5752 (4046-7558)		42 (30-56)		1961 (848-2975)		14 (6-22)		16 (7-24)		0 (0-0)	
2007	Bihar	5532 (3891-7269)		40 (28-53)		1888 (808-2874)		14 (6-21)		15 (6-23)		0 (0-0)	
2008	Bihar	5297 (3726-6960)		38 (27-50)		1809 (774-2751)		13 (6-20)		14 (6-22)		0 (0-0)	
2009	Bihar	5025 (3534-6603)		36 (25-47)		1717 (734-2610)		12 (5-19)		14 (6-21)		0 (0-0)	
2010	Bihar	4716 (3317-6196)		33 (23-44)		1611 (689-2449)		11 (5-17)		13 (5-20)		0 (0-0)	
2011	Bihar	4367 (3071-5737)		31 (22-40)		1492 (638-2268)		10 (4-16)		12 (5-18)		0 (0-0)	
2012	Bihar	3961 (2786-5204)		28 (20-36)		1353 (579-2057)		9 (4-14)		11 (5-16)		0 (0-0)	
2013	Bihar	3599 (2531-4729)		25 (18-33)		1230 (526-1869)		9 (4-13)		10 (4-15)		0 (0-0)	
2014	Bihar	3212 (2259-4220)		22 (16-29)		1097 (469-1668)		8 (3-12)		9 (4-13)		0 (0-0)	
2015	Bihar	479 (337-629)	1 (1-2)	3 (2-4)	0 (0-0)	164 (70-249)	0 (0-1)	1 (0-2)	0 (0-0)	1 (1-2)	0 (0-0)	0 (0-0)	0 (0-0)
2000	Chhattisgarh	1264 (889-1661)		45 (32-60)		440 (172-679)		16 (6-24)		2 (1-3)		0 (0-0)	
2001	Chhattisgarh	1252 (880-1644)		45 (31-59)		436 (170-672)		16 (6-24)		2 (1-3)		0 (0-0)	
2002	Chhattisgarh	1215 (855-1597)		43 (30-57)		423 (165-653)		15 (6-23)		2 (1-3)		0 (0-0)	
2003	Chhattisgarh	1176 (827-1545)		41 (29-55)		409 (160-632)		14 (6-22)		2 (1-3)		0 (0-0)	
2004	Chhattisgarh	1116 (785-1466)		39 (28-52)		388 (152-599)		14 (5-21)		2 (1-2)		0 (0-0)	
2005	Chhattisgarh	1061 (746-1394)		37 (26-49)		368 (149-564)		13 (5-20)		1 (1-2)		0 (0-0)	
2006	Chhattisgarh	1034 (727-1358)		36 (25-48)		358 (150-542)		13 (5-19)		1 (1-2)		0 (0-0)	
2007	Chhattisgarh	964 (678-1267)		34 (24-44)		333 (134-512)		12 (5-18)		3 (1-4)		0 (0-0)	
2008	Chhattisgarh	920 (647-1209)		32 (23-42)		317 (128-488)		11 (4-17)		3 (1-4)		0 (0-0)	
2009	Chhattisgarh	865 (609-1137)		30 (21-40)		298 (120-459)		10 (4-16)		2 (1-4)		0 (0-0)	
2010	Chhattisgarh	804 (566-1057)		28 (20-37)		277 (112-427)		10 (4-15)		2 (1-3)		0 (0-0)	
2011	Chhattisgarh	762 (536-1001)		27 (19-35)		262 (106-404)		9 (4-14)		2 (1-3)		0 (0-0)	
2012	Chhattisgarh	716 (503-941)		25 (18-33)		246 (100-380)		9 (4-14)		2 (1-3)		0 (0-0)	
2013	Chhattisgarh	698 (491-917)		25 (18-33)		240 (97-370)		9 (3-13)		2 (1-3)		0 (0-0)	
2014	Chhattisgarh	697 (490-915)		25 (18-33)		240 (97-370)		9 (4-13)		2 (1-3)		0 (0-0)	
2015	Chhattisgarh	224 (157-294)	1 (0-1)	8 (6-11)	0 (0-0)	77 (31-119)	0 (0-0)	3 (1-4)	0 (0-0)	1 (0-1)	0 (0-0)	0 (0-0)	0 (0-0)
2000	Delhi	397 (279-521)		25 (18-33)		137 (53-214)		9 (3-14)		1 (0-2)		0 (0-0)	
2001	Delhi	384 (270-505)		24 (17-32)		133 (51-207)		8 (3-13)		1 (0-2)		0 (0-0)	
2002	Delhi	370 (261-487)		23 (16-31)		128 (50-200)		8 (3-13)		1 (0-2)		0 (0-0)	
2003	Delhi	356 (250-468)		23 (16-30)		123 (48-192)		8 (3-12)		1 (0-2)		0 (0-0)	
2004	Delhi	340 (239-447)		22 (15-28)		117 (46-183)		7 (3-12)		1 (0-1)		0 (0-0)	

Year	State	Hib pneumonia deaths (UR)	Hib pneumonia deaths; HIV (UR)	Hib pneumonia mortality rate (UR)	Hib pneumonia mortality rate; HIV (UR)	Hib meningitis deaths (UR)	Hib meningitis deaths; HIV (UR)	Hib meningitis mortality rate (UR)	Hib mortality rate; HIV (UR)	Hib NPNM deaths (UR)	Hib NPNM deaths; HIV (UR)	Hib NPNM mortality rate (UR)	Hib NPNM mortality rate; HIV (UR)
2005	Delhi	323 (228-425)		20 (14-27)		111 (46-170)		7 (3-11)		1 (0-1)		0 (0-0)	
2006	Delhi	307 (216-403)		19 (14-26)		104 (47-155)		7 (3-10)		1 (0-1)		0 (0-0)	
2007	Delhi	288 (203-378)		18 (13-24)		97 (48-140)		6 (3-9)		1 (0-1)		0 (0-0)	
2008	Delhi	269 (190-354)		17 (12-23)		90 (45-131)		6 (3-8)		1 (0-1)		0 (0-0)	
2009	Delhi	250 (176-329)		16 (11-21)		83 (41-121)		5 (3-8)		1 (0-1)		0 (0-0)	
2010	Delhi	230 (162-302)		15 (10-19)		76 (38-111)		5 (2-7)		1 (0-1)		0 (0-0)	
2011	Delhi	208 (146-273)		14 (10-18)		68 (34-101)		4 (2-7)		1 (0-1)		0 (0-0)	
2012	Delhi	184 (130-242)		12 (9-16)		59 (16-90)		4 (1-6)		0 (0-1)		0 (0-0)	
2013	Delhi	84 (59-111)		6 (4-7)		26 (7-41)		2 (0-3)		0 (0-0)		0 (0-0)	
2014	Delhi	14 (10-19)		1 (1-1)		4 (1-7)		0 (0-0)		0 (0-0)		0 (0-0)	
2015	Delhi	12 (9-16)	0 (0-0)	1 (1-1)	0 (0-0)	4 (1-6)	0 (0-0)	0 (0-0)	0 (0-0)	0 (0-0)	0 (0-0)	0 (0-0)	0 (0-0)
2000	Goa	9 (6-11)		7 (5-10)		3 (1-4)		2 (1-4)		0 (0-0)		0 (0-0)	
2001	Goa	7 (5-9)		6 (4-8)		2 (1-4)		2 (0-3)		0 (0-0)		0 (0-0)	
2002	Goa	5 (4-7)		4 (3-6)		2 (0-3)		1 (0-2)		0 (0-0)		0 (0-0)	
2003	Goa	6 (4-8)		5 (4-7)		2 (1-3)		2 (0-3)		0 (0-0)		0 (0-0)	
2004	Goa	6 (5-9)		5 (4-7)		2 (1-3)		2 (0-3)		0 (0-0)		0 (0-0)	
2005	Goa	7 (5-9)		6 (4-7)		2 (1-3)		2 (0-3)		0 (0-0)		0 (0-0)	
2006	Goa	6 (4-8)		5 (4-7)		2 (0-3)		2 (0-3)		0 (0-0)		0 (0-0)	
2007	Goa	4 (3-5)		3 (2-4)		1 (0-2)		1 (0-2)		0 (0-0)		0 (0-0)	
2008	Goa	3 (2-4)		3 (2-4)		1 (0-2)		1 (0-2)		0 (0-0)		0 (0-0)	
2009	Goa	4 (3-5)		3 (2-4)		1 (0-2)		1 (0-2)		0 (0-0)		0 (0-0)	
2010	Goa	4 (3-5)		3 (2-4)		1 (0-2)		1 (0-2)		0 (0-0)		0 (0-0)	
2011	Goa	3 (2-4)		3 (2-3)		1 (0-1)		1 (0-1)		0 (0-0)		0 (0-0)	
2012	Goa	2 (1-3)		2 (1-2)		1 (0-1)		1 (0-1)		0 (0-0)		0 (0-0)	
2013	Goa	0 (0-0)		0 (0-0)		0 (0-0)		0 (0-0)		0 (0-0)		0 (0-0)	
2014	Goa	0 (0-0)		0 (0-0)		0 (0-0)		0 (0-0)		0 (0-0)		0 (0-0)	
2015	Goa	0 (0-0)	0 (0-0)	0 (0-0)	0 (0-0)	0 (0-0)	0 (0-0)	0 (0-0)	0 (0-0)	0 (0-0)	0 (0-0)	0 (0-0)	0 (0-0)
2000	Gujarat	2487 (1749-3268)		42 (29-55)		865 (342-1330)		14 (6-22)		4 (1-5)		0 (0-0)	
2001	Gujarat	2372 (1669-3117)		39 (28-52)		825 (327-1269)		14 (5-21)		3 (1-5)		0 (0-0)	
2002	Gujarat	2261 (1590-2971)		37 (26-49)		787 (311-1209)		13 (5-20)		3 (1-5)		0 (0-0)	
2003	Gujarat	2135 (1502-2805)		35 (25-46)		743 (269-1175)		12 (4-19)		6 (2-9)		0 (0-0)	
2004	Gujarat	1979 (1392-2600)		32 (23-43)		687 (250-1089)		11 (4-18)		5 (2-9)		0 (0-0)	
2005	Gujarat	1881 (1323-2472)		31 (22-40)		651 (249-1018)		11 (4-17)		5 (2-8)		0 (0-0)	
2006	Gujarat	1809 (1272-2376)		29 (21-39)		623 (252-959)		10 (4-16)		5 (2-8)		0 (0-0)	
2007	Gujarat	1691 (1190-2222)		28 (19-36)		580 (249-875)		9 (4-14)		5 (2-7)		0 (0-0)	
2008	Gujarat	1587 (1116-2085)		26 (18-34)		543 (234-821)		9 (4-13)		4 (2-7)		0 (0-0)	
2009	Gujarat	1477 (1039-1941)		24 (17-32)		504 (218-764)		8 (4-12)		4 (2-6)		0 (0-0)	
2010	Gujarat	1359 (956-1786)		22 (16-29)		464 (200-703)		8 (3-12)		4 (2-6)		0 (0-0)	
2011	Gujarat	1256 (883-1650)		21 (15-27)		429 (185-650)		7 (3-11)		3 (1-5)		0 (0-0)	
2012	Gujarat	1162 (817-1527)		19 (14-25)		397 (171-601)		7 (3-10)		3 (1-5)		0 (0-0)	
2013	Gujarat	406 (285-533)		7 (5-9)		138 (60-210)		2 (1-4)		1 (0-2)		0 (0-0)	
2014	Gujarat	132 (93-173)		2 (2-3)		45 (19-68)		1 (0-1)		0 (0-1)		0 (0-0)	
2015	Gujarat	125 (88-165)	0 (0-0)	2 (1-3)	0 (0-0)	43 (18-65)	0 (0-0)	1 (0-1)	0 (0-0)	0 (0-1)	0 (0-0)	0 (0-0)	0 (0-0)
2000	Haryana	1478 (1040-1942)		57 (40-75)		505 (229-754)		20 (9-29)		2 (1-3)		0 (0-0)	
2001	Haryana	1411 (993-1854)		54 (38-71)		482 (219-720)		19 (8-28)		2 (1-3)		0 (0-0)	
2002	Haryana	1338 (941-1758)		51 (36-67)		457 (208-683)		17 (8-26)		2 (1-3)		0 (0-0)	
2003	Haryana	1261 (887-1657)		48 (34-63)		430 (196-644)		16 (7-24)		2 (1-3)		0 (0-0)	
2004	Haryana	1177 (828-1547)		45 (31-59)		401 (170-614)		15 (6-23)		3 (1-5)		0 (0-0)	
2005	Haryana	1075 (756-1412)		41 (29-53)		365 (162-550)		14 (6-21)		3 (1-4)		0 (0-0)	
2006	Haryana	967 (680-1271)		36 (26-48)		327 (151-485)		12 (6-18)		3 (1-4)		0 (0-0)	
2007	Haryana	919 (646-1207)		35 (24-45)		309 (150-449)		12 (6-17)		2 (1-4)		0 (0-0)	
2008	Haryana	847 (596-1113)		32 (22-42)		284 (138-414)		11 (5-16)		2 (1-3)		0 (0-0)	
2009	Haryana	753 (530-989)		28 (20-37)		252 (122-370)		10 (5-14)		2 (1-3)		0 (0-0)	
2010	Haryana	726 (511-954)		27 (19-36)		243 (117-359)		9 (4-14)		2 (1-3)		0 (0-0)	
2011	Haryana	678 (477-891)		26 (18-34)		228 (108-337)		9 (4-13)		2 (1-3)		0 (0-0)	
2012	Haryana	591 (416-776)		23 (16-30)		199 (93-295)		8 (4-11)		2 (1-2)		0 (0-0)	
2013	Haryana	152 (107-199)		6 (4-8)		51 (24-76)		2 (1-3)		0 (0-1)		0 (0-0)	
2014	Haryana	62 (44-81)		2 (2-3)		21 (10-31)		1 (0-1)		0 (0-0)		0 (0-0)	
2015	Haryana	60 (42-79)	0 (0-0)	2 (2-3)	0 (0-0)	20 (9-30)	0 (0-0)	1 (0-1)	0 (0-0)	0 (0-0)	0 (0-0)	0 (0-0)	0 (0-0)
2000	Himachal Pradesh	262 (184-344)		41 (29-54)		89 (38-136)		14 (6-21)		1 (0-1)		0 (0-0)	
2001	Himachal Pradesh	241 (169-316)		38 (26-49)		82 (35-125)		13 (5-20)		1 (0-1)		0 (0-0)	
2002	Himachal Pradesh	221 (155-290)		34 (24-45)		75 (32-115)		12 (5-18)		1 (0-1)		0 (0-0)	
2003	Himachal Pradesh	202 (142-266)		32 (22-42)		69 (29-105)		11 (5-16)		1 (0-1)		0 (0-0)	
2004	Himachal Pradesh	186 (131-244)		29 (21-38)		63 (27-97)		10 (4-15)		1 (0-1)		0 (0-0)	
2005	Himachal Pradesh	170 (120-224)		27 (19-35)		58 (25-88)		9 (4-14)		0 (0-1)		0 (0-0)	
2006	Himachal Pradesh	156 (110-205)		25 (17-32)		53 (24-80)		8 (4-13)		0 (0-1)		0 (0-0)	
2007	Himachal Pradesh	143 (100-187)		23 (16-30)		48 (22-72)		8 (4-11)		0 (0-1)		0 (0-0)	
2008	Himachal Pradesh	131 (92-172)		21 (15-28)		44 (20-66)		7 (3-11)		0 (0-1)		0 (0-0)	
2009	Himachal Pradesh	120 (85-158)		19 (14-26)		40 (19-60)		7 (3-10)		0 (0-0)		0 (0-0)	
2010	Himachal Pradesh	110 (78-145)		18 (13-24)		37 (18-54)		6 (3-9)		0 (0-0)		0 (0-0)	
2011	Himachal Pradesh	102 (72-134)		17 (12-22)		34 (17-49)		6 (3-8)		0 (0-0)		0 (0-0)	
2012	Himachal Pradesh	94 (66-124)		16 (11-21)		31 (17-45)		5 (3-8)		0 (0-0)		0 (0-0)	
2013	Himachal Pradesh	91 (64-119)		15 (11-20)		30 (16-43)		5 (3-7)		0 (0-0)		0 (0-0)	
2014	Himachal Pradesh	89 (62-117)		15 (11-20)		29 (16-42)		5 (3-7)		0 (0-0)		0 (0-0)	
2015	Himachal Pradesh	82 (58-108)	0 (0-0)	14 (10-19)	0 (0-0)	27 (14-39)	0 (0-0)	5 (3-7)	0 (0-0)	0 (0-0)	0 (0-0)	0 (0-0)	0 (0-0)
2000	Jammu & Kashmir	308 (217-405)		29 (21-38)		100 (55-148)		9 (5-14)		1 (0-1)		0 (0-0)	
2001	Jammu & Kashmir	288 (202-378)		26 (18-34)		93 (52-138)		8 (5-12)		1 (0-1)		0 (0-0)	
2002	Jammu & Kashmir	268 (188-352)		23 (16-31)		87 (48-128)		8 (4-11)		1 (0-1)		0 (0-0)	
2003	Jammu & Kashmir	250 (176-328)		21 (15-27)		82 (45-120)		7 (4-10)		1 (0-1)		0 (0-0)	
2004	Jammu & Kashmir	234 (164-307)		19 (13-25)		77 (42-112)		6 (3-9)		1 (0-1)		0 (0-0)	
2005	Jammu & Kashmir	218 (154-287)		17 (12-22)		72 (37-104)		6 (3-8)		1 (0-1)		0 (0-0)	
2006	Jammu & Kashmir	204 (144-269)		15 (11-20)		68 (33-100)		5 (2-7)		1 (0-1)		0 (0-0)	
2007	Jammu & Kashmir	190 (134-250)		14 (10-18)		64 (30-96)		5 (2-7)		1 (0-1)		0 (0-0)	
2008	Jammu & Kashmir	177 (125-233)		12 (9-16)		60 (28-89)		4 (2-6)		0 (0-1)		0 (0-0)	
2009	Jammu & Kashmir	165 (116-216)		11 (8-15)		56 (26-83)		4 (2-6)		0 (0-1)		0 (0-0)	
2010	Jammu & Kashmir	152 (107-199)		10 (7-13)		51 (24-76)		3 (2-5)		0 (0-1)		0 (0-0)	
2011	Jammu & Kashmir	138 (97-182)		9 (6-12)		47 (22-70)		3 (1-4)		0 (0-1)		0 (0-0)	

Hib = *Haemophilus influenzae* type b; npnm = non-pneumonia, non-meningitis; HIV = HIV-infected cases only; UR = uncertainty range

Year	State	Hib pneumonia deaths (UR)	Hib pneumonia deaths; HIV (UR)	Hib pneumonia mortality rate (UR)	Hib pneumonia mortality rate; HIV (UR)	Hib meningitis deaths (UR)	Hib meningitis deaths; HIV (UR)	Hib meningitis mortality rate (UR)	Hib mortality rate; HIV (UR)	Hib NPNM deaths (UR)	Hib NPNM deaths; HIV (UR)	Hib NPNM mortality rate (UR)	Hib NPNM mortality rate; HIV (UR)
2012	Jammu & Kashmir	125 (88-164)		8 (5-10)		42 (19-63)		3 (1-4)		0 (0-1)		0 (0-0)	
2013	Jammu & Kashmir	41 (29-53)		2 (2-3)		14 (6-20)		1 (0-1)		0 (0-0)		0 (0-0)	
2014	Jammu & Kashmir	12 (9-16)		1 (1-1)		4 (2-6)		0 (0-0)		0 (0-0)		0 (0-0)	
2015	Jammu & Kashmir	12 (9-16)	0 (0-0)	1 (0-1)	0 (0-0)	4 (2-6)	0 (0-0)	0 (0-0)	0 (0-0)	0 (0-0)	0 (0-0)	0 (0-0)	0 (0-0)
2000	Jharkhand	1376 (968-1809)		37 (26-49)		482 (181-747)		13 (5-20)		2 (1-3)		0 (0-0)	
2001	Jharkhand	1366 (961-1794)		36 (26-48)		478 (164-764)		13 (4-20)		4 (1-6)		0 (0-0)	
2002	Jharkhand	1335 (939-1755)		35 (25-46)		468 (160-747)		12 (4-20)		4 (1-6)		0 (0-0)	
2003	Jharkhand	1320 (928-1734)		34 (24-45)		462 (158-739)		12 (4-19)		4 (1-6)		0 (0-0)	
2004	Jharkhand	1246 (876-1637)		32 (23-42)		436 (149-697)		11 (4-18)		3 (1-6)		0 (0-0)	
2005	Jharkhand	1133 (797-1489)		29 (20-38)		397 (137-633)		10 (3-16)		3 (1-5)		0 (0-0)	
2006	Jharkhand	1122 (789-1474)		28 (20-37)		392 (137-625)		10 (3-16)		3 (1-5)		0 (0-0)	
2007	Jharkhand	1037 (730-1363)		26 (18-34)		363 (127-576)		9 (3-14)		3 (1-5)		0 (0-0)	
2008	Jharkhand	925 (651-1216)		23 (16-30)		323 (114-514)		8 (3-13)		3 (1-4)		0 (0-0)	
2009	Jharkhand	817 (575-1074)		20 (14-27)		286 (100-454)		7 (2-11)		2 (1-4)		0 (0-0)	
2010	Jharkhand	816 (574-1073)		20 (14-26)		285 (100-453)		7 (2-11)		2 (1-4)		0 (0-0)	
2011	Jharkhand	764 (538-1004)		19 (13-25)		267 (94-424)		7 (2-10)		2 (1-3)		0 (0-0)	
2012	Jharkhand	694 (488-912)		17 (12-22)		243 (85-385)		6 (2-9)		2 (1-3)		0 (0-0)	
2013	Jharkhand	617 (434-811)		15 (11-20)		216 (76-343)		5 (2-8)		2 (1-3)		0 (0-0)	
2014	Jharkhand	539 (379-708)		13 (9-17)		188 (66-299)		5 (2-7)		2 (1-2)		0 (0-0)	
2015	Jharkhand	116 (82-153)	0 (0-0)	3 (2-4)	0 (0-0)	41 (14-65)	0 (0-0)	1 (0-2)	0 (0-0)	0 (0-1)	0 (0-0)	0 (0-0)	0 (0-0)
2000	Karnataka	1946 (1369-2557)		35 (24-46)		671 (268-1037)		12 (5-18)		5 (2-8)		0 (0-0)	
2001	Karnataka	1882 (1323-2472)		33 (23-44)		649 (259-1003)		11 (5-18)		5 (2-8)		0 (0-0)	
2002	Karnataka	1849 (1301-2430)		33 (23-43)		637 (255-985)		11 (4-17)		5 (2-8)		0 (0-0)	
2003	Karnataka	1819 (1279-2389)		32 (22-42)		626 (251-969)		11 (4-17)		5 (2-8)		0 (0-0)	
2004	Karnataka	1780 (1252-2338)		31 (22-41)		612 (245-948)		11 (4-17)		5 (2-8)		0 (0-0)	
2005	Karnataka	1723 (1212-2264)		30 (21-40)		591 (244-908)		10 (4-16)		5 (2-7)		0 (0-0)	
2006	Karnataka	1660 (1168-2182)		29 (20-38)		568 (242-864)		10 (4-15)		5 (2-7)		0 (0-0)	
2007	Karnataka	1571 (1105-2065)		27 (19-36)		536 (235-807)		9 (4-14)		4 (2-6)		0 (0-0)	
2008	Karnataka	1473 (1036-1936)		26 (18-34)		501 (220-756)		9 (4-13)		4 (2-6)		0 (0-0)	
2009	Karnataka	1364 (959-1792)		24 (17-32)		462 (208-693)		8 (4-12)		4 (2-6)		0 (0-0)	
2010	Karnataka	1236 (869-1624)		22 (15-29)		417 (193-621)		7 (3-11)		3 (2-5)		0 (0-0)	
2011	Karnataka	1096 (771-1440)		19 (14-26)		369 (175-544)		7 (3-10)		3 (1-4)		0 (0-0)	
2012	Karnataka	934 (657-1227)		17 (12-22)		313 (152-458)		6 (3-8)		2 (1-4)		0 (0-0)	
2013	Karnataka	258 (181-339)		5 (3-6)		86 (42-126)		2 (1-2)		1 (0-1)		0 (0-0)	
2014	Karnataka	55 (38-72)		1 (1-1)		18 (9-27)		0 (0-0)		0 (0-0)		0 (0-0)	
2015	Karnataka	42 (30-56)	0 (0-0)	1 (1-1)	0 (0-0)	14 (7-21)	0 (0-0)	0 (0-0)	0 (0-0)	0 (0-0)	0 (0-0)	0 (0-0)	0 (0-0)
2000	Kerala	170 (119-223)		5 (4-7)		55 (11-92)		2 (0-3)		0 (0-1)		0 (0-0)	
2001	Kerala	165 (116-217)		5 (4-7)		54 (11-89)		2 (0-3)		0 (0-1)		0 (0-0)	
2002	Kerala	160 (113-211)		5 (4-7)		52 (11-87)		2 (0-3)		0 (0-1)		0 (0-0)	
2003	Kerala	157 (110-206)		5 (4-7)		51 (11-85)		2 (0-3)		0 (0-1)		0 (0-0)	
2004	Kerala	154 (109-203)		5 (4-7)		50 (10-83)		2 (0-3)		0 (0-1)		0 (0-0)	
2005	Kerala	152 (107-200)		5 (4-7)		49 (11-80)		2 (0-3)		0 (0-1)		0 (0-0)	
2006	Kerala	152 (107-200)		5 (4-7)		48 (12-77)		2 (0-3)		0 (0-1)		0 (0-0)	
2007	Kerala	152 (107-200)		5 (4-7)		47 (13-73)		2 (0-2)		0 (0-1)		0 (0-0)	
2008	Kerala	154 (108-202)		5 (4-7)		47 (14-74)		2 (0-3)		0 (0-1)		0 (0-0)	
2009	Kerala	156 (110-205)		5 (4-7)		47 (14-75)		2 (0-3)		0 (0-1)		0 (0-0)	
2010	Kerala	158 (111-208)		6 (4-7)		48 (14-76)		2 (0-3)		0 (0-1)		0 (0-0)	
2011	Kerala	160 (113-211)		6 (4-8)		49 (14-78)		2 (1-3)		0 (0-1)		0 (0-0)	
2012	Kerala	25 (18-33)		1 (1-1)		8 (2-12)		0 (0-0)		0 (0-0)		0 (0-0)	
2013	Kerala	13 (9-17)		0 (0-1)		4 (1-6)		0 (0-0)		0 (0-0)		0 (0-0)	
2014	Kerala	13 (9-17)		1 (0-1)		4 (1-6)		0 (0-0)		0 (0-0)		0 (0-0)	
2015	Kerala	13 (9-17)	0 (0-0)	1 (0-1)	0 (0-0)	4 (1-6)	0 (0-0)	0 (0-0)	0 (0-0)	0 (0-0)	0 (0-0)	0 (0-0)	0 (0-0)
2000	Madhya Pradesh	6165 (4336-8101)		74 (52-97)		2156 (810-3349)		26 (10-40)		9 (3-14)		0 (0-0)	
2001	Madhya Pradesh	5896 (4147-7747)		70 (49-92)		2062 (774-3203)		24 (9-38)		8 (3-13)		0 (0-0)	
2002	Madhya Pradesh	5644 (3970-7416)		67 (47-88)		1973 (741-3066)		23 (9-36)		8 (3-12)		0 (0-0)	
2003	Madhya Pradesh	5401 (3799-7097)		64 (45-84)		1888 (709-2934)		22 (8-35)		8 (3-12)		0 (0-0)	
2004	Madhya Pradesh	5155 (3625-6773)		61 (43-80)		1799 (677-2800)		21 (8-33)		7 (3-11)		0 (0-0)	
2005	Madhya Pradesh	4914 (3456-6456)		58 (41-76)		1712 (662-2647)		20 (8-31)		7 (3-11)		0 (0-0)	
2006	Madhya Pradesh	4684 (3294-6155)		55 (39-72)		1629 (648-2501)		19 (8-29)		7 (3-10)		0 (0-0)	
2007	Madhya Pradesh	4429 (3115-5820)		52 (37-69)		1538 (629-2343)		18 (7-28)		6 (3-10)		0 (0-0)	
2008	Madhya Pradesh	4187 (2945-5501)		49 (35-65)		1451 (594-2215)		17 (7-26)		6 (2-9)		0 (0-0)	
2009	Madhya Pradesh	3950 (2778-5190)		47 (33-62)		1368 (561-2089)		16 (7-25)		6 (2-9)		0 (0-0)	
2010	Madhya Pradesh	3711 (2610-4876)		44 (31-58)		1285 (527-1963)		15 (6-23)		5 (2-8)		0 (0-0)	
2011	Madhya Pradesh	3492 (2456-4588)		42 (30-55)		1210 (496-1847)		15 (6-22)		5 (2-8)		0 (0-0)	
2012	Madhya Pradesh	3278 (2305-4307)		40 (28-52)		1136 (465-1734)		14 (6-21)		5 (2-7)		0 (0-0)	
2013	Madhya Pradesh	3140 (2209-4126)		38 (27-51)		1089 (410-1708)		13 (5-21)		9 (3-14)		0 (0-0)	
2014	Madhya Pradesh	2817 (1981-3701)		35 (25-46)		977 (368-1532)		12 (5-19)		8 (3-12)		0 (0-0)	
2015	Madhya Pradesh	316 (222-416)	1 (1-1)	4 (3-5)	0 (0-0)	110 (41-172)	0 (0-0)	1 (1-2)	0 (0-0)	1 (0-1)	0 (0-0)	0 (0-0)	0 (0-0)
2000	Maharashtra	2773 (1950-3643)		26 (18-34)		949 (402-1445)		9 (4-13)		8 (3-12)		0 (0-0)	
2001	Maharashtra	2592 (1823-3406)		24 (17-31)		887 (376-1351)		8 (3-12)		7 (3-11)		0 (0-0)	
2002	Maharashtra	2406 (1692-3161)		22 (15-29)		823 (349-1254)		8 (3-11)		7 (3-10)		0 (0-0)	
2003	Maharashtra	2220 (1561-2917)		20 (14-27)		759 (322-1157)		7 (3-11)		6 (3-9)		0 (0-0)	
2004	Maharashtra	2037 (1433-2676)		19 (13-25)		695 (295-1062)		6 (3-10)		6 (2-8)		0 (0-0)	
2005	Maharashtra	1861 (1309-2446)		17 (12-23)		634 (277-959)		6 (3-9)		5 (2-8)		0 (0-0)	
2006	Maharashtra	1696 (1193-2228)		16 (11-21)		576 (258-863)		5 (2-8)		5 (2-7)		0 (0-0)	
2007	Maharashtra	1527 (1074-2006)		14 (10-19)		517 (239-766)		5 (2-7)		4 (2-6)		0 (0-0)	
2008	Maharashtra	1380 (971-1814)		13 (9-17)		466 (216-692)		4 (2-6)		4 (2-6)		0 (0-0)	
2009	Maharashtra	1239 (871-1628)		12 (8-15)		417 (194-621)		4 (2-6)		3 (2-5)		0 (0-0)	
2010	Maharashtra	1099 (773-1444)		10 (7-14)		367 (172-551)		3 (2-5)		3 (1-4)		0 (0-0)	
2011	Maharashtra	992 (697-1303)		10 (7-12)		328 (155-497)		3 (1-5)		3 (1-4)		0 (0-0)	
2012	Maharashtra	878 (618-1154)		9 (6-11)		287 (67-454)		3 (1-4)		2 (1-4)		0 (0-0)	
2013	Maharashtra	783 (550-1028)		8 (5-10)		252 (60-404)		2 (1-4)		2 (0-3)		0 (0-0)	
2014	Maharashtra	690 (486-907)		7 (5-9)		220 (53-357)		2 (1-4)		2 (0-3)		0 (0-0)	
2015	Maharashtra	580 (408-762)	1 (1-2)	6 (4-8)	0 (0-0)	184 (44-300)	0 (0-1)	2 (0-3)	0 (0-0)	1 (0-2)	0 (0-0)	0 (0-0)	0 (0-0)
2000	Manipur	31 (22-41)		14 (10-18)		11 (4-17)		5 (2-8)		0 (0-0)		0 (0-0)	
2001	Manipur	29 (21-39)		12 (9-16)		10 (3-17)		4 (1-7)		0 (0-0)		0 (0-0)	
2002	Manipur	28 (20-37)		12 (8-15)		10 (3-16)		4 (1-7)		0 (0-0)		0 (0-0)	

Hib = *Haemophilus influenzae* type b; npnm = non-pneumonia, non-meningitis; HIV = HIV-infected cases only; UR = uncertainty range

Year	State	Hib pneumonia deaths (UR)	Hib pneumonia deaths; HIV (UR)	Hib pneumonia mortality rate (UR)	Hib pneumonia mortality rate; HIV (UR)	Hib meningitis deaths (UR)	Hib meningitis deaths; HIV (UR)	Hib meningitis mortality rate (UR)	Hib mortality rate; HIV (UR)	Hib NPNM deaths (UR)	Hib NPNM deaths; HIV (UR)	Hib NPNM mortality rate (UR)	Hib NPNM mortality rate; HIV (UR)
2003	Manipur	26 (19-35)		11 (8-14)		9 (1-16)		4 (0-6)		0 (0-0)		0 (0-0)	
2004	Manipur	26 (18-34)		10 (7-13)		9 (1-15)		4 (0-6)		0 (0-0)		0 (0-0)	
2005	Manipur	25 (18-33)		10 (7-13)		9 (1-15)		3 (0-6)		0 (0-0)		0 (0-0)	
2006	Manipur	25 (17-32)		9 (7-12)		9 (1-15)		3 (0-6)		0 (0-0)		0 (0-0)	
2007	Manipur	25 (17-32)		9 (6-12)		9 (1-15)		3 (0-6)		0 (0-0)		0 (0-0)	
2008	Manipur	25 (18-33)		9 (6-12)		9 (1-15)		3 (0-6)		0 (0-0)		0 (0-0)	
2009	Manipur	25 (18-33)		9 (6-12)		9 (1-15)		3 (0-5)		0 (0-0)		0 (0-0)	
2010	Manipur	26 (18-34)		9 (6-12)		9 (1-15)		3 (0-5)		0 (0-0)		0 (0-0)	
2011	Manipur	26 (18-34)		9 (6-12)		9 (1-15)		3 (0-5)		0 (0-0)		0 (0-0)	
2012	Manipur	26 (18-34)		9 (6-12)		9 (1-15)		3 (0-5)		0 (0-0)		0 (0-0)	
2013	Manipur	26 (18-34)		9 (6-12)		9 (1-15)		3 (0-5)		0 (0-0)		0 (0-0)	
2014	Manipur	25 (18-33)		9 (6-11)		9 (1-15)		3 (0-5)		0 (0-0)		0 (0-0)	
2015	Manipur	22 (16-29)	0 (0-0)	7 (5-10)	0 (0-0)	8 (1-13)	0 (0-0)	3 (0-4)	0 (0-0)	0 (0-0)	0 (0-0)	0 (0-0)	0 (0-0)
2000	Meghalaya	159 (112-209)		44 (31-58)		54 (25-81)		15 (7-22)		0 (0-0)		0 (0-0)	
2001	Meghalaya	165 (116-216)		44 (31-58)		56 (26-84)		15 (7-23)		0 (0-0)		0 (0-0)	
2002	Meghalaya	171 (120-225)		45 (32-59)		59 (27-88)		15 (7-23)		0 (0-0)		0 (0-0)	
2003	Meghalaya	178 (125-234)		46 (32-60)		61 (28-91)		16 (7-23)		0 (0-0)		0 (0-0)	
2004	Meghalaya	185 (130-243)		46 (33-61)		63 (29-94)		16 (7-24)		0 (0-0)		0 (0-0)	
2005	Meghalaya	191 (134-251)		47 (33-62)		65 (30-97)		16 (7-24)		0 (0-0)		0 (0-0)	
2006	Meghalaya	197 (139-259)		47 (33-62)		67 (32-99)		16 (8-24)		0 (0-0)		0 (0-0)	
2007	Meghalaya	202 (142-266)		48 (34-63)		69 (33-101)		16 (8-24)		0 (0-0)		0 (0-0)	
2008	Meghalaya	208 (146-274)		48 (34-63)		71 (34-104)		16 (8-24)		0 (0-0)		0 (0-0)	
2009	Meghalaya	211 (148-277)		48 (34-63)		72 (33-108)		16 (7-24)		0 (0-0)		0 (0-0)	
2010	Meghalaya	221 (155-290)		50 (35-65)		76 (33-115)		17 (7-26)		0 (0-0)		0 (0-0)	
2011	Meghalaya	228 (160-300)		50 (35-66)		79 (33-120)		17 (7-27)		0 (0-0)		0 (0-0)	
2012	Meghalaya	234 (164-307)		51 (36-67)		81 (32-125)		18 (7-27)		0 (0-1)		0 (0-0)	
2013	Meghalaya	242 (170-317)		52 (37-69)		84 (33-129)		18 (7-28)		0 (0-1)		0 (0-0)	
2014	Meghalaya	247 (174-325)		53 (37-69)		86 (34-132)		18 (7-28)		0 (0-1)		0 (0-0)	
2015	Meghalaya	231 (163-304)	1 (0-1)	49 (34-64)	0 (0-0)	80 (32-124)	0 (0-0)	17 (7-26)	0 (0-0)	0 (0-1)	0 (0-0)	0 (0-0)	0 (0-0)
2000	Mizoram	47 (33-62)		42 (29-55)		17 (6-26)		15 (5-23)		0 (0-0)		0 (0-0)	
2001	Mizoram	46 (32-60)		40 (28-52)		16 (6-25)		14 (5-22)		0 (0-0)		0 (0-0)	
2002	Mizoram	45 (32-59)		38 (27-50)		16 (6-25)		13 (5-21)		0 (0-0)		0 (0-0)	
2003	Mizoram	45 (31-59)		37 (26-49)		16 (6-24)		13 (5-20)		0 (0-0)		0 (0-0)	
2004	Mizoram	44 (31-58)		36 (25-48)		16 (6-24)		13 (5-20)		0 (0-0)		0 (0-0)	
2005	Mizoram	44 (31-58)		35 (25-47)		15 (6-24)		12 (5-19)		0 (0-0)		0 (0-0)	
2006	Mizoram	44 (31-58)		35 (25-46)		15 (6-24)		12 (5-19)		0 (0-0)		0 (0-0)	
2007	Mizoram	44 (31-58)		34 (24-45)		15 (6-24)		12 (5-19)		0 (0-0)		0 (0-0)	
2008	Mizoram	44 (31-58)		34 (24-44)		15 (6-24)		12 (4-18)		0 (0-0)		0 (0-0)	
2009	Mizoram	44 (31-58)		33 (23-44)		15 (6-24)		12 (4-18)		0 (0-0)		0 (0-0)	
2010	Mizoram	43 (31-57)		32 (23-43)		15 (6-23)		11 (4-17)		0 (0-0)		0 (0-0)	
2011	Mizoram	44 (31-58)		32 (23-43)		15 (6-23)		11 (4-17)		0 (0-0)		0 (0-0)	
2012	Mizoram	43 (31-57)		32 (22-42)		15 (6-23)		11 (4-17)		0 (0-0)		0 (0-0)	
2013	Mizoram	44 (31-57)		32 (22-42)		15 (6-23)		11 (4-17)		0 (0-0)		0 (0-0)	
2014	Mizoram	43 (31-57)		31 (22-41)		15 (6-23)		11 (4-17)		0 (0-0)		0 (0-0)	
2015	Mizoram	38 (27-50)	0 (0-0)	27 (19-36)	0 (0-0)	13 (5-21)	0 (0-0)	10 (4-15)	0 (0-0)	0 (0-0)	0 (0-0)	0 (0-0)	0 (0-0)
2000	Nagaland	32 (22-42)		15 (11-20)		11 (4-18)		5 (2-8)		0 (0-0)		0 (0-0)	
2001	Nagaland	34 (24-44)		16 (11-21)		12 (4-19)		6 (2-9)		0 (0-0)		0 (0-0)	
2002	Nagaland	36 (25-47)		17 (12-22)		13 (4-20)		6 (2-9)		0 (0-0)		0 (0-0)	
2003	Nagaland	39 (27-51)		18 (12-23)		14 (4-22)		6 (2-10)		0 (0-0)		0 (0-0)	
2004	Nagaland	41 (29-54)		19 (13-25)		14 (5-23)		7 (2-11)		0 (0-0)		0 (0-0)	
2005	Nagaland	43 (30-56)		20 (14-26)		15 (5-24)		7 (2-11)		0 (0-0)		0 (0-0)	
2006	Nagaland	44 (31-58)		20 (14-26)		16 (5-25)		7 (2-11)		0 (0-0)		0 (0-0)	
2007	Nagaland	45 (32-59)		20 (14-27)		16 (5-26)		7 (2-12)		0 (0-0)		0 (0-0)	
2008	Nagaland	46 (32-60)		21 (15-27)		16 (5-26)		7 (2-12)		0 (0-0)		0 (0-0)	
2009	Nagaland	45 (32-59)		20 (14-27)		16 (5-26)		7 (2-12)		0 (0-0)		0 (0-0)	
2010	Nagaland	47 (33-62)		21 (15-28)		17 (5-28)		8 (2-13)		0 (0-0)		0 (0-0)	
2011	Nagaland	47 (33-62)		22 (15-28)		17 (5-28)		8 (2-13)		0 (0-0)		0 (0-0)	
2012	Nagaland	47 (33-61)		21 (15-28)		17 (5-28)		8 (2-13)		0 (0-0)		0 (0-0)	
2013	Nagaland	46 (32-60)		21 (15-28)		16 (4-27)		8 (2-12)		0 (0-0)		0 (0-0)	
2014	Nagaland	44 (31-58)		20 (14-27)		16 (4-26)		7 (2-12)		0 (0-0)		0 (0-0)	
2015	Nagaland	40 (28-53)	0 (0-0)	19 (13-24)	0 (0-0)	14 (4-24)	0 (0-0)	7 (2-11)	0 (0-0)	0 (0-0)	0 (0-0)	0 (0-0)	0 (0-0)
2000	Odisha	2867 (2016-3767)		71 (50-93)		992 (408-1514)		24 (10-37)		4 (2-6)		0 (0-0)	
2001	Odisha	2707 (1904-3557)		66 (47-87)		937 (386-1430)		23 (9-35)		4 (2-6)		0 (0-0)	
2002	Odisha	2544 (1789-3342)		62 (44-81)		881 (362-1344)		21 (9-33)		4 (1-5)		0 (0-0)	
2003	Odisha	2398 (1687-3151)		58 (41-76)		830 (342-1267)		20 (8-31)		3 (1-5)		0 (0-0)	
2004	Odisha	2253 (1585-2961)		55 (38-72)		781 (321-1190)		19 (8-29)		3 (1-5)		0 (0-0)	
2005	Odisha	2112 (1486-2775)		51 (36-67)		732 (299-1118)		18 (7-27)		3 (1-5)		0 (0-0)	
2006	Odisha	1981 (1393-2603)		48 (34-63)		687 (279-1051)		17 (7-25)		3 (1-4)		0 (0-0)	
2007	Odisha	1833 (1289-2409)		44 (31-58)		636 (257-975)		15 (6-24)		3 (1-4)		0 (0-0)	
2008	Odisha	1700 (1196-2234)		41 (29-54)		590 (238-904)		14 (6-22)		2 (1-4)		0 (0-0)	
2009	Odisha	1570 (1104-2063)		38 (27-50)		545 (220-835)		13 (5-20)		2 (1-3)		0 (0-0)	
2010	Odisha	1445 (1017-1899)		35 (25-46)		502 (202-769)		12 (5-19)		2 (1-3)		0 (0-0)	
2011	Odisha	1335 (939-1754)		33 (23-43)		464 (172-730)		11 (4-18)		4 (1-6)		0 (0-0)	
2012	Odisha	1223 (860-1607)		30 (21-40)		425 (157-669)		11 (4-17)		3 (1-5)		0 (0-0)	
2013	Odisha	1129 (794-1483)		28 (20-37)		393 (145-618)		10 (4-15)		3 (1-5)		0 (0-0)	
2014	Odisha	1022 (719-1343)		26 (18-34)		355 (131-559)		9 (3-14)		3 (1-4)		0 (0-0)	
2015	Odisha	820 (577-1078)	2 (1-3)	21 (15-27)	0 (0-0)	285 (105-449)	1 (0-1)	7 (3-11)	0 (0-0)	2 (1-4)	0 (0-0)	0 (0-0)	0 (0-0)
2000	Punjab	903 (635-1186)		37 (26-49)		302 (146-444)		12 (6-18)		2 (1-4)		0 (0-0)	
2001	Punjab	839 (590-1103)		34 (24-45)		281 (136-413)		12 (6-17)		2 (1-3)		0 (0-0)	
2002	Punjab	777 (547-1021)		32 (22-42)		260 (126-382)		11 (5-16)		2 (1-3)		0 (0-0)	
2003	Punjab	720 (507-946)		29 (21-39)		241 (117-354)		10 (5-14)		2 (1-3)		0 (0-0)	
2004	Punjab	665 (468-874)		27 (19-36)		222 (108-327)		9 (4-13)		2 (1-3)		0 (0-0)	
2005	Punjab	612 (431-805)		25 (18-33)		204 (101-297)		8 (4-12)		2 (1-2)		0 (0-0)	
2006	Punjab	564 (396-741)		23 (16-30)		187 (96-269)		8 (4-11)		1 (1-2)		0 (0-0)	
2007	Punjab	515 (362-677)		21 (15-28)		170 (89-246)		7 (4-10)		1 (1-2)		0 (0-0)	
2008	Punjab	471 (331-619)		19 (14-26)		155 (82-225)		6 (3-9)		1 (1-2)		0 (0-0)	
2009	Punjab	431 (303-566)		18 (13-23)		142 (75-206)		6 (3-9)		1 (1-2)		0 (0-0)	

Hib = *Haemophilus influenzae* type b; npnm = non-pneumonia, non-meningitis; HIV = HIV-infected cases only; UR = uncertainty range

Year	State	Hib pneumonia deaths (UR)	Hib pneumonia deaths; HIV (UR)	Hib pneumonia mortality rate (UR)	Hib pneumonia mortality rate; HIV (UR)	Hib meningitis deaths (UR)	Hib meningitis deaths; HIV (UR)	Hib meningitis mortality rate (UR)	Hib mortality rate; HIV (UR)	Hib NPNM deaths (UR)	Hib NPNM deaths; HIV (UR)	Hib NPNM mortality rate (UR)	Hib NPNM mortality rate; HIV (UR)
2010	Punjab	394 (277-517)		16 (12-22)		129 (68-188)		5 (3-8)		1 (1-1)		0 (0-0)	
2011	Punjab	361 (254-474)		15 (11-20)		118 (62-172)		5 (3-7)		1 (0-1)		0 (0-0)	
2012	Punjab	330 (232-433)		14 (10-18)		107 (57-157)		5 (2-7)		1 (0-1)		0 (0-0)	
2013	Punjab	307 (216-403)		13 (9-17)		98 (53-147)		4 (2-6)		1 (0-1)		0 (0-0)	
2014	Punjab	287 (202-377)		12 (9-16)		90 (27-132)		4 (1-6)		1 (0-1)		0 (0-0)	
2015	Punjab	46 (32-60)	0 (0-0)	2 (1-3)	0 (0-0)	14 (4-21)	0 (0-0)	1 (0-1)	0 (0-0)	0 (0-0)	0 (0-0)	0 (0-0)	0 (0-0)
2000	Rajasthan	5099 (3586-6699)		62 (44-81)		1761 (738-2678)		21 (9-33)		7 (3-11)		0 (0-0)	
2001	Rajasthan	4892 (3441-6428)		59 (42-78)		1690 (708-2570)		20 (9-31)		7 (3-10)		0 (0-0)	
2002	Rajasthan	4679 (3291-6148)		56 (40-74)		1616 (677-2458)		19 (8-30)		7 (3-10)		0 (0-0)	
2003	Rajasthan	4447 (3128-5843)		53 (38-70)		1535 (644-2336)		18 (8-28)		6 (3-10)		0 (0-0)	
2004	Rajasthan	4207 (2959-5527)		51 (36-66)		1451 (609-2209)		17 (7-27)		6 (2-9)		0 (0-0)	
2005	Rajasthan	3952 (2780-5193)		48 (33-62)		1361 (581-2063)		16 (7-25)		6 (2-8)		0 (0-0)	
2006	Rajasthan	3702 (2604-4865)		45 (31-58)		1273 (553-1920)		15 (7-23)		5 (2-8)		0 (0-0)	
2007	Rajasthan	3437 (2417-4515)		41 (29-54)		1181 (522-1770)		14 (6-21)		5 (2-7)		0 (0-0)	
2008	Rajasthan	3191 (2244-4192)		39 (27-51)		1095 (485-1644)		13 (6-20)		4 (2-7)		0 (0-0)	
2009	Rajasthan	2960 (2082-3889)		36 (25-47)		1016 (418-1561)		12 (5-19)		8 (3-12)		0 (0-0)	
2010	Rajasthan	2731 (1921-3589)		33 (23-44)		938 (385-1441)		11 (5-18)		7 (3-11)		0 (0-0)	
2011	Rajasthan	2518 (1771-3308)		31 (22-41)		865 (355-1328)		11 (4-16)		7 (3-11)		0 (0-0)	
2012	Rajasthan	2303 (1620-3026)		29 (20-38)		791 (325-1215)		10 (4-15)		6 (3-10)		0 (0-0)	
2013	Rajasthan	2141 (1506-2814)		27 (19-35)		736 (302-1130)		9 (4-14)		6 (2-9)		0 (0-0)	
2014	Rajasthan	1867 (1313-2453)		24 (17-31)		641 (263-985)		8 (3-12)		5 (2-8)		0 (0-0)	
2015	Rajasthan	188 (132-247)	0 (0-1)	2 (2-3)	0 (0-0)	65 (27-99)	0 (0-0)	1 (0-1)	0 (0-0)	1 (0-1)	0 (0-0)	0 (0-0)	0 (0-0)
2000	Sikkim	14 (10-19)		23 (16-31)		5 (2-7)		8 (3-12)		0 (0-0)		0 (0-0)	
2001	Sikkim	13 (9-17)		22 (16-29)		5 (2-7)		8 (3-12)		0 (0-0)		0 (0-0)	
2002	Sikkim	12 (9-16)		21 (15-28)		4 (2-6)		7 (3-11)		0 (0-0)		0 (0-0)	
2003	Sikkim	11 (8-15)		20 (14-26)		4 (2-6)		7 (3-10)		0 (0-0)		0 (0-0)	
2004	Sikkim	10 (7-14)		19 (13-24)		4 (2-5)		6 (3-10)		0 (0-0)		0 (0-0)	
2005	Sikkim	10 (7-13)		17 (12-23)		3 (1-5)		6 (2-9)		0 (0-0)		0 (0-0)	
2006	Sikkim	9 (6-11)		16 (11-21)		3 (1-5)		6 (2-9)		0 (0-0)		0 (0-0)	
2007	Sikkim	8 (6-11)		15 (11-20)		3 (1-5)		5 (2-9)		0 (0-0)		0 (0-0)	
2008	Sikkim	8 (5-10)		15 (10-19)		3 (1-4)		5 (2-8)		0 (0-0)		0 (0-0)	
2009	Sikkim	7 (5-9)		14 (10-19)		3 (1-4)		5 (2-8)		0 (0-0)		0 (0-0)	
2010	Sikkim	7 (5-9)		14 (10-19)		2 (1-4)		5 (2-8)		0 (0-0)		0 (0-0)	
2011	Sikkim	7 (5-9)		15 (10-19)		2 (1-4)		5 (2-8)		0 (0-0)		0 (0-0)	
2012	Sikkim	7 (5-9)		15 (10-19)		2 (1-4)		5 (2-8)		0 (0-0)		0 (0-0)	
2013	Sikkim	7 (5-9)		15 (11-20)		2 (0-4)		5 (1-8)		0 (0-0)		0 (0-0)	
2014	Sikkim	7 (5-9)		16 (11-20)		2 (0-4)		5 (1-9)		0 (0-0)		0 (0-0)	
2015	Sikkim	6 (4-8)	0 (0-0)	14 (10-18)	0 (0-0)	2 (0-3)	0 (0-0)	5 (1-8)	0 (0-0)	0 (0-0)	0 (0-0)	0 (0-0)	0 (0-0)
2000	Tamil Nadu	1356 (954-1782)		23 (16-31)		464 (197-706)		8 (3-12)		4 (2-6)		0 (0-0)	
2001	Tamil Nadu	1294 (910-1700)		22 (16-29)		443 (188-674)		8 (3-12)		4 (2-5)		0 (0-0)	
2002	Tamil Nadu	1227 (863-1613)		21 (15-27)		420 (179-639)		7 (3-11)		3 (1-5)		0 (0-0)	
2003	Tamil Nadu	1160 (816-1525)		20 (14-26)		396 (169-604)		7 (3-10)		3 (1-5)		0 (0-0)	
2004	Tamil Nadu	1091 (767-1434)		18 (13-24)		372 (159-568)		6 (3-10)		3 (1-5)		0 (0-0)	
2005	Tamil Nadu	1017 (715-1336)		17 (12-23)		346 (151-523)		6 (3-9)		3 (1-4)		0 (0-0)	
2006	Tamil Nadu	945 (664-1241)		16 (11-21)		321 (144-480)		5 (2-8)		3 (1-4)		0 (0-0)	
2007	Tamil Nadu	867 (610-1140)		15 (10-19)		294 (136-435)		5 (2-7)		2 (1-3)		0 (0-0)	
2008	Tamil Nadu	798 (561-1048)		13 (9-18)		269 (125-400)		5 (2-7)		2 (1-3)		0 (0-0)	
2009	Tamil Nadu	734 (516-964)		12 (9-16)		247 (113-372)		4 (2-6)		2 (1-3)		0 (0-0)	
2010	Tamil Nadu	671 (472-882)		11 (8-15)		225 (101-343)		4 (2-6)		2 (1-3)		0 (0-0)	
2011	Tamil Nadu	611 (430-803)		10 (7-14)		203 (41-330)		3 (1-6)		2 (0-3)		0 (0-0)	
2012	Tamil Nadu	135 (95-177)		2 (2-3)		45 (9-73)		1 (0-1)		0 (0-1)		0 (0-0)	
2013	Tamil Nadu	54 (38-71)		1 (1-1)		18 (4-29)		0 (0-1)		0 (0-0)		0 (0-0)	
2014	Tamil Nadu	44 (31-57)		1 (1-1)		14 (3-24)		0 (0-0)		0 (0-0)		0 (0-0)	
2015	Tamil Nadu	35 (25-46)	0 (0-0)	1 (0-1)	0 (0-0)	12 (2-19)	0 (0-0)	0 (0-0)	0 (0-0)	0 (0-0)	0 (0-0)	0 (0-0)	0 (0-0)
2000	Tripura	72 (51-94)		22 (15-28)		25 (9-39)		8 (3-12)		0 (0-0)		0 (0-0)	
2001	Tripura	65 (46-85)		19 (14-25)		23 (8-36)		7 (2-11)		0 (0-0)		0 (0-0)	
2002	Tripura	59 (42-78)		17 (12-23)		21 (8-33)		6 (2-10)		0 (0-0)		0 (0-0)	
2003	Tripura	54 (38-71)		16 (11-21)		19 (7-30)		5 (2-9)		0 (0-0)		0 (0-0)	
2004	Tripura	49 (35-65)		14 (10-19)		17 (6-27)		5 (2-8)		0 (0-0)		0 (0-0)	
2005	Tripura	45 (32-60)		13 (9-17)		16 (6-25)		5 (2-7)		0 (0-0)		0 (0-0)	
2006	Tripura	42 (30-55)		12 (8-16)		15 (6-23)		4 (2-6)		0 (0-0)		0 (0-0)	
2007	Tripura	40 (28-52)		11 (8-15)		14 (5-21)		4 (2-6)		0 (0-0)		0 (0-0)	
2008	Tripura	37 (26-48)		10 (7-14)		13 (5-20)		4 (1-5)		0 (0-0)		0 (0-0)	
2009	Tripura	35 (24-46)		10 (7-13)		12 (5-18)		3 (1-5)		0 (0-0)		0 (0-0)	
2010	Tripura	33 (23-43)		9 (6-12)		11 (5-17)		3 (1-5)		0 (0-0)		0 (0-0)	
2011	Tripura	32 (22-42)		9 (6-12)		11 (5-16)		3 (1-4)		0 (0-0)		0 (0-0)	
2012	Tripura	29 (21-38)		8 (6-11)		10 (5-14)		3 (1-4)		0 (0-0)		0 (0-0)	
2013	Tripura	27 (19-36)		8 (5-10)		9 (4-13)		2 (1-4)		0 (0-0)		0 (0-0)	
2014	Tripura	24 (17-32)		7 (5-9)		8 (2-12)		2 (1-3)		0 (0-0)		0 (0-0)	
2015	Tripura	21 (15-27)	0 (0-0)	6 (4-8)	0 (0-0)	7 (2-10)	0 (0-0)	2 (0-3)	0 (0-0)	0 (0-0)	0 (0-0)	0 (0-0)	0 (0-0)
2000	Union Territory	49 (34-64)		18 (12-23)		16 (7-25)		6 (2-9)		0 (0-0)		0 (0-0)	
2001	Union Territory	47 (33-61)		17 (12-22)		15 (6-24)		5 (2-8)		0 (0-0)		0 (0-0)	
2002	Union Territory	44 (31-58)		16 (11-21)		15 (6-23)		5 (2-8)		0 (0-0)		0 (0-0)	
2003	Union Territory	43 (30-56)		15 (10-20)		14 (5-22)		5 (2-8)		0 (0-0)		0 (0-0)	
2004	Union Territory	41 (29-54)		14 (10-19)		13 (5-21)		5 (2-7)		0 (0-0)		0 (0-0)	
2005	Union Territory	39 (27-51)		13 (9-18)		13 (4-20)		4 (2-7)		0 (0-0)		0 (0-0)	
2006	Union Territory	37 (26-49)		13 (9-17)		12 (5-18)		4 (2-6)		0 (0-0)		0 (0-0)	
2007	Union Territory	36 (25-47)		12 (9-16)		11 (5-16)		4 (2-6)		0 (0-0)		0 (0-0)	
2008	Union Territory	34 (24-45)		12 (8-15)		10 (4-16)		4 (1-5)		0 (0-0)		0 (0-0)	
2009	Union Territory	33 (23-43)		11 (8-15)		10 (4-15)		3 (1-5)		0 (0-0)		0 (0-0)	
2010	Union Territory	31 (22-41)		11 (7-14)		9 (4-15)		3 (1-5)		0 (0-0)		0 (0-0)	
2011	Union Territory	30 (21-40)		10 (7-14)		9 (3-14)		3 (1-5)		0 (0-0)		0 (0-0)	
2012	Union Territory	29 (20-38)		10 (7-13)		8 (3-13)		3 (1-5)		0 (0-0)		0 (0-0)	
2013	Union Territory	22 (15-29)		7 (5-10)		6 (3-10)		2 (1-3)		0 (0-0)		0 (0-0)	
2014	Union Territory	20 (14-26)		7 (5-9)		6 (2-9)		2 (1-3)		0 (0-0)		0 (0-0)	
2015	Union Territory	7 (5-10)	0 (0-0)	3 (2-3)	0 (0-0)	2 (1-4)	0 (0-0)	1 (0-1)	0 (0-0)	0 (0-0)	0 (0-0)	0 (0-0)	0 (0-0)
2000	Uttar Pradesh	17458 (12279-22939)		74 (52-97)		5950 (2746-8862)		25 (12-37)		24 (11-36)		0 (0-0)	

Hib = *Haemophilus influenzae* type b; npnm = non-pneumonia, non-meningitis; HIV = HIV-infected cases only; UR = uncertainty range

Year	State	Hib pneumonia deaths (UR)	Hib pneumonia deaths; HIV (UR)	Hib pneumonia mortality rate (UR)	Hib pneumonia mortality rate; HIV (UR)	Hib meningitis deaths (UR)	Hib meningitis deaths; HIV (UR)	Hib meningitis mortality rate (UR)	Hib mortality rate; HIV (UR)	Hib NPNM deaths (UR)	Hib NPNM deaths; HIV (UR)	Hib NPNM mortality rate (UR)	Hib NPNM mortality rate; HIV (UR)
2001	Uttar Pradesh	16731 (11767-21983)		71 (50-93)		5702 (2632-8493)		24 (11-36)		23 (11-35)		0 (0-0)	
2002	Uttar Pradesh	15988 (11245-21007)		67 (47-88)		5449 (2515-8116)		23 (11-34)		22 (10-33)		0 (0-0)	
2003	Uttar Pradesh	15266 (10737-20059)		64 (45-85)		5205 (2401-7749)		22 (10-33)		21 (10-32)		0 (0-0)	
2004	Uttar Pradesh	14527 (10217-19087)		61 (43-81)		4956 (2285-7374)		21 (10-31)		20 (9-30)		0 (0-0)	
2005	Uttar Pradesh	13734 (9659-18045)		58 (41-77)		4689 (2142-6998)		20 (9-30)		19 (9-29)		0 (0-0)	
2006	Uttar Pradesh	12984 (9132-17060)		55 (39-73)		4437 (2008-6640)		19 (9-28)		18 (8-27)		0 (0-0)	
2007	Uttar Pradesh	12174 (8562-15996)		52 (37-68)		4164 (1866-6249)		18 (8-27)		17 (8-25)		0 (0-0)	
2008	Uttar Pradesh	11405 (8021-14985)		49 (34-64)		3904 (1749-5854)		17 (8-25)		16 (7-24)		0 (0-0)	
2009	Uttar Pradesh	10684 (7515-14039)		46 (33-61)		3659 (1638-5484)		16 (7-24)		15 (7-22)		0 (0-0)	
2010	Uttar Pradesh	9956 (7002-13082)		43 (31-57)		3411 (1526-5110)		15 (7-22)		14 (6-21)		0 (0-0)	
2011	Uttar Pradesh	9261 (6513-12168)		41 (29-54)		3174 (1320-4864)		14 (6-21)		25 (11-39)		0 (0-0)	
2012	Uttar Pradesh	8524 (5995-11201)		38 (27-50)		2923 (1215-4478)		13 (5-20)		23 (10-36)		0 (0-0)	
2013	Uttar Pradesh	7941 (5585-10434)		36 (25-47)		2724 (1132-4171)		12 (5-19)		22 (9-33)		0 (0-0)	
2014	Uttar Pradesh	7357 (5174-9667)		34 (24-44)		2524 (1049-3864)		12 (5-18)		20 (8-31)		0 (0-0)	
2015	Uttar Pradesh	6913 (4862-9083)	17 (12-23)	32 (22-42)	0 (0-0)	2372 (985-3631)	6 (3-10)	11 (5-17)	0 (0-0)	19 (8-29)	0 (0-0)	0 (0-0)	0 (0-0)
2000	Uttarakhand	389 (274-511)		37 (26-49)		133 (61-198)		13 (6-19)		1 (0-1)		0 (0-0)	
2001	Uttarakhand	372 (262-489)		35 (25-46)		127 (58-189)		12 (6-18)		1 (0-1)		0 (0-0)	
2002	Uttarakhand	355 (250-466)		34 (24-44)		121 (56-180)		11 (5-17)		0 (0-1)		0 (0-0)	
2003	Uttarakhand	338 (238-444)		32 (22-42)		116 (49-176)		11 (5-17)		1 (0-1)		0 (0-0)	
2004	Uttarakhand	321 (226-422)		30 (21-40)		110 (47-167)		10 (4-16)		1 (0-1)		0 (0-0)	
2005	Uttarakhand	303 (213-398)		29 (20-38)		104 (43-160)		10 (4-15)		1 (0-1)		0 (0-0)	
2006	Uttarakhand	286 (201-376)		27 (19-36)		99 (39-153)		9 (4-14)		1 (0-1)		0 (0-0)	
2007	Uttarakhand	268 (188-352)		25 (18-33)		93 (35-145)		9 (3-14)		1 (0-1)		0 (0-0)	
2008	Uttarakhand	251 (176-330)		24 (17-31)		87 (33-136)		8 (3-13)		1 (0-1)		0 (0-0)	
2009	Uttarakhand	234 (164-307)		22 (16-29)		81 (31-127)		8 (3-12)		1 (0-1)		0 (0-0)	
2010	Uttarakhand	215 (152-283)		21 (15-27)		75 (28-117)		7 (3-11)		1 (0-1)		0 (0-0)	
2011	Uttarakhand	196 (138-258)		19 (13-25)		68 (26-106)		7 (3-10)		1 (0-1)		0 (0-0)	
2012	Uttarakhand	174 (122-229)		17 (12-22)		60 (23-94)		6 (2-9)		0 (0-1)		0 (0-0)	
2013	Uttarakhand	155 (109-203)		15 (11-20)		54 (20-84)		5 (2-8)		0 (0-1)		0 (0-0)	
2014	Uttarakhand	136 (95-178)		14 (10-18)		47 (18-74)		5 (2-7)		0 (0-1)		0 (0-0)	
2015	Uttarakhand	20 (14-26)	0 (0-0)	2 (1-3)	0 (0-0)	7 (3-11)	0 (0-0)	1 (0-1)	0 (0-0)	0 (0-0)	0 (0-0)	0 (0-0)	0 (0-0)
2000	West Bengal	2965 (2085-3895)		34 (24-45)		1009 (442-1525)		12 (5-18)		8 (4-12)		0 (0-0)	
2001	West Bengal	2708 (1905-3559)		31 (22-41)		922 (404-1393)		11 (5-16)		7 (3-11)		0 (0-0)	
2002	West Bengal	2438 (1715-3203)		28 (20-37)		830 (364-1254)		10 (4-14)		7 (3-10)		0 (0-0)	
2003	West Bengal	2203 (1549-2894)		25 (18-33)		750 (329-1133)		9 (4-13)		6 (3-9)		0 (0-0)	
2004	West Bengal	2005 (1410-2634)		23 (16-31)		682 (299-1031)		8 (3-12)		5 (2-8)		0 (0-0)	
2005	West Bengal	1830 (1287-2404)		21 (15-28)		622 (274-939)		7 (3-11)		5 (2-7)		0 (0-0)	
2006	West Bengal	1676 (1179-2202)		20 (14-26)		570 (253-858)		7 (3-10)		5 (2-7)		0 (0-0)	
2007	West Bengal	1527 (1074-2007)		18 (13-24)		519 (231-779)		6 (3-9)		4 (2-6)		0 (0-0)	
2008	West Bengal	1383 (973-1817)		16 (12-22)		469 (209-706)		6 (2-8)		4 (2-6)		0 (0-0)	
2009	West Bengal	1251 (880-1643)		15 (11-20)		424 (191-636)		5 (2-8)		3 (2-5)		0 (0-0)	
2010	West Bengal	1166 (820-1532)		14 (10-19)		395 (179-591)		5 (2-7)		3 (1-5)		0 (0-0)	
2011	West Bengal	1125 (791-1478)		14 (10-18)		381 (174-569)		5 (2-7)		3 (1-5)		0 (0-0)	
2012	West Bengal	1091 (767-1434)		14 (10-18)		369 (169-550)		5 (2-7)		3 (1-4)		0 (0-0)	
2013	West Bengal	1094 (769-1438)		14 (10-18)		370 (170-551)		5 (2-7)		3 (1-4)		0 (0-0)	
2014	West Bengal	1102 (775-1447)		14 (10-19)		372 (171-555)		5 (2-7)		3 (1-4)		0 (0-0)	
2015	West Bengal	176 (124-231)	0 (0-1)	2 (2-3)	0 (0-0)	59 (27-88)	0 (0-0)	1 (0-1)	0 (0-0)	0 (0-1)	0 (0-0)	0 (0-0)	0 (0-0)

Hib = *Haemophilus influenzae* type b; npnm = non-pneumonia, non-meningitis; HIV = HIV-infected cases only; UR = uncertainty range

Webappendix 11: Pneumococcal cases in India by state, year, and HIV status

		Pneumonia cases				Severe pneumonia cases				Meningitis cases				NPNM cases			
Year	State	Spn pneumonia cases (UR)	Spn pneumonia cases; HIV (UR)	Spn pneumonia incidence rate (UR)	Spn pneumonia incidence rate; HIV (UR)	Spn severe pneumonia cases (UR)	Spn severe pneumonia cases; HIV (UR)	Spn severe pneumonia incidence rate (UR)	Spn severe pneumonia incidence rate; HIV (UR)	Spn meningitis cases (UR)	Spn meningitis cases; HIV (UR)	Spn meningitis incidence rate (UR)	Spn meningitis incidence rate; HIV (UR)	Spn NPNM cases (UR)	Spn NPNM cases; HIV (UR)	Spn NPNM incidence rate (UR)	Spn NPNM incidence rate; HIV (UR)
2000	Andhra Pradesh	322365 (278143-383201)		4210 (3632-5004)		132498 (99266-151126)		1730 (1296-1974)		2313 (1190-4231)		30 (16-55)		928 (477-1697)		12 (6-22)	
2001	Andhra Pradesh									2292 (1082-4310)		30 (14-56)		2603 (1228-4896)		34 (16-64)	
2002	Andhra Pradesh									2236 (1057-4214)		29 (14-55)		2540 (1201-4786)		33 (16-63)	
2003	Andhra Pradesh									2157 (1022-4072)		28 (13-54)		2450 (1160-4625)		32 (15-61)	
2004	Andhra Pradesh									2062 (978-3897)		27 (13-52)		2342 (1111-4427)		31 (15-59)	
2005	Andhra Pradesh									1944 (919-3678)		26 (12-49)		2208 (1044-4178)		30 (14-56)	
2006	Andhra Pradesh									1819 (859-3448)		25 (12-46)		2066 (975-3917)		28 (13-53)	
2007	Andhra Pradesh									1681 (792-3194)		23 (11-43)		1909 (900-3628)		26 (12-49)	
2008	Andhra Pradesh									1535 (726-2927)		21 (10-40)		1743 (825-3324)		24 (11-46)	
2009	Andhra Pradesh									1374 (633-2648)		19 (9-37)		1561 (719-3008)		22 (10-42)	
2010	Andhra Pradesh									1212 (545-2363)		17 (8-33)		1377 (620-2684)		19 (9-38)	
2011	Andhra Pradesh									1062 (467-2091)		15 (7-30)		1206 (530-2375)		17 (8-34)	
2012	Andhra Pradesh									918 (394-1825)		13 (6-27)		1043 (447-2073)		15 (6-30)	
2013	Andhra Pradesh									809 (348-1613)		12 (5-24)		919 (395-1832)		14 (6-27)	
2014	Andhra Pradesh									722 (311-1441)		11 (5-22)		820 (353-1636)		12 (5-25)	
2015	Andhra Pradesh	136860 (118086-162688)	1919 (1656-2281)	2091 (1804-2485)	29 (25-35)	56250 (42142-64159)	789 (591-900)	859 (644-980)	12 (9-14)	670 (289-1338)	10 (4-20)	10 (4-20)	0 (0-0)	761 (328-1519)	11 (5-23)	12 (5-23)	0 (0-0)
2000	Arunachal Pradesh	6848 (5908-8140)		4379 (3778-5205)		2811 (2106-3207)		1798 (1347-2051)		22 (8-47)		14 (5-30)		25 (9-54)		16 (6-34)	
2001	Arunachal Pradesh									20 (7-45)		13 (5-28)		23 (8-51)		15 (5-32)	
2002	Arunachal Pradesh									20 (7-45)		13 (5-28)		23 (8-51)		15 (5-32)	
2003	Arunachal Pradesh									21 (7-46)		13 (5-29)		24 (8-52)		15 (5-33)	
2004	Arunachal Pradesh									22 (8-48)		14 (5-30)		25 (9-54)		16 (5-34)	
2005	Arunachal Pradesh									23 (9-49)		15 (6-31)		26 (10-56)		17 (6-35)	
2006	Arunachal Pradesh									26 (11-53)		16 (7-33)		30 (13-61)		19 (8-38)	
2007	Arunachal Pradesh									27 (13-52)		17 (8-32)		30 (15-59)		19 (9-37)	
2008	Arunachal Pradesh									27 (13-53)		17 (8-33)		31 (15-60)		19 (9-37)	
2009	Arunachal Pradesh									26 (11-52)		16 (7-33)		30 (13-59)		18 (8-37)	
2010	Arunachal Pradesh									24 (10-49)		15 (6-31)		27 (11-56)		17 (7-35)	
2011	Arunachal Pradesh									21 (8-45)		13 (5-28)		24 (9-51)		15 (6-32)	
2012	Arunachal Pradesh									19 (7-42)		12 (4-26)		22 (7-47)		14 (5-30)	
2013	Arunachal Pradesh									18 (6-39)		11 (4-25)		20 (7-44)		13 (4-28)	
2014	Arunachal Pradesh									17 (6-37)		11 (4-24)		19 (7-42)		12 (4-27)	
2015	Arunachal Pradesh	2591 (2236-3080)	36 (31-43)	1674 (1445-1990)	23 (20-28)	1065 (798-1215)	15 (11-17)	688 (516-785)	10 (7-11)	15 (5-34)	0 (0-1)	10 (3-22)	0 (0-0)	18 (6-39)	0 (0-1)	11 (4-25)	0 (0-0)
2000	Assam	137674 (118788-163655)		4008 (3458-4765)		56599 (42404-64557)		1648 (1235-1880)		1184 (504-2431)		34 (15-71)		475 (202-975)		14 (6-28)	
2001	Assam									1143 (487-2348)		33 (14-68)		459 (195-942)		13 (6-27)	
2002	Assam									1113 (474-2285)		32 (14-65)		446 (190-917)		13 (5-26)	
2003	Assam									1095 (466-2246)		31 (13-64)		439 (187-901)		12 (5-26)	
2004	Assam									1087 (461-2226)		31 (13-63)		436 (185-893)		12 (5-25)	
2005	Assam									1085 (467-2210)		31 (13-62)		435 (187-886)		12 (5-25)	
2006	Assam									1085 (474-2196)		30 (13-61)		435 (190-881)		12 (5-25)	
2007	Assam									1089 (483-2191)		30 (13-61)		437 (194-879)		12 (5-25)	
2008	Assam									1092 (484-2193)		30 (13-61)		438 (194-880)		12 (5-24)	
2009	Assam									1082 (479-2171)		30 (13-60)		434 (192-871)		12 (5-24)	
2010	Assam									1074 (475-2154)		30 (13-60)		431 (190-864)		12 (5-24)	
2011	Assam									1064 (471-2134)		30 (13-60)		427 (189-856)		12 (5-24)	
2012	Assam									1051 (465-2110)		30 (13-59)		422 (187-846)		12 (5-24)	
2013	Assam									1054 (467-2117)		30 (13-60)		423 (187-849)		12 (5-24)	
2014	Assam									1048 (465-2109)		30 (13-60)		420 (187-846)		12 (5-24)	
2015	Assam	92971 (80217-110516)	1304 (1125-1550)	2649 (2286-3149)	37 (32-44)	38228 (28640-43602)	536 (402-611)	1089 (816-1242)	15 (11-17)	1047 (406-2173)	16 (6-32)	30 (12-62)	0 (0-1)	1189 (461-2468)	18 (7-37)	34 (13-70)	1 (0-1)
2000	Bihar	755665 (652004-898273)		6120 (5281-7275)		310782 (232837-354477)		2517 (1886-2871)		5970 (3055-10760)		48 (25-87)		2395 (1225-4316)		19 (10-35)	
2001	Bihar									5817 (2977-10483)		46 (24-83)		2333 (1194-4205)		19 (9-33)	
2002	Bihar									5629 (2881-10146)		44 (22-79)		2258 (1156-4070)		18 (9-32)	
2003	Bihar									5452 (2795-9845)		42 (21-76)		2187 (1121-3949)		17 (9-30)	
2004	Bihar									5279 (2717-9567)		40 (21-73)		2117 (1090-3837)		16 (8-29)	
2005	Bihar									5123 (2620-9358)		38 (20-70)		2055 (1051-3754)		15 (8-28)	
2006	Bihar									4993 (2314-9440)		37 (17-70)		5671 (2628-10722)		42 (19-79)	
2007	Bihar									4834 (2220-9217)		35 (16-67)		5490 (2522-10468)		40 (18-76)	
2008	Bihar									4648 (2144-8898)		33 (15-64)		5280 (2435-10107)		38 (18-73)	
2009	Bihar									4419 (2042-8475)		32 (15-60)		5019 (2319-9626)		36 (17-69)	
2010	Bihar									4168 (1926-7996)		30 (14-57)		4734 (2188-9082)		34 (15-64)	
2011	Bihar									3907 (1806-7495)		27 (13-53)		4437 (2051-8513)		31 (14-60)	
2012	Bihar									3597 (1662-6901)		25 (12-48)		4085 (1888-7838)		29 (13-55)	
2013	Bihar									3323 (1536-6376)		23 (11-45)		3774 (1745-7241)		26 (12-51)	
2014	Bihar									3020 (1396-5794)		21 (10-40)		3430 (1585-6581)		24 (11-46)	
2015	Bihar	549088 (473765-652711)	7699 (6643-9152)	3797 (3276-4513)	53 (46-63)	225728 (169114-257465)	3165 (2371-3610)	1561 (1169-1780)	22 (16-25)	2742 (1267-5260)	41 (19-78)	19 (9-36)	0 (0-1)	3114 (1439-5974)	46 (21-89)	22 (10-41)	0 (0-1)
2000	Chhattisgarh	167498 (144521-199108)		6019 (5193-7154)		68880 (51605-78565)		2475 (1854-2823)		824 (356-1676)		30 (13-60)		330 (143-672)		12 (5-24)	
2001	Chhattisgarh									816 (353-1660)		29 (13-59)		327 (141-666)		12 (5-24)	
2002	Chhattisgarh									795 (344-1617)		28 (12-57)		319 (138-649)		11 (5-23)	
2003	Chhattisgarh									776 (334-1573)		27 (12-55)		311 (134-631)		11 (5-22)	
2004	Chhattisgarh									751 (323-1519)		26 (11-53)		301 (129-609)		11 (5-21)	
2005	Chhattisgarh									741 (332-1478)		26 (12-52)		297 (133-593)		10 (5-21)	
2006	Chhattisgarh									755 (352-1484)		26 (12-52)		303 (141-595)		11 (5-21)	
2007	Chhattisgarh									741 (323-1475)		26 (11-52)		842 (367-1675)		29 (13-59)	
2008	Chhattisgarh									732 (319-1453)		26 (11-51)		831 (362-1650)		29 (13-58)	
2009	Chhattisgarh									696 (303-1381)		24 (11-48)		791 (344-1569)		28 (12-55)	
2010	Chhattisgarh									646 (281-1283)		23 (10-45)		734 (320-1457)		26 (11-51)	
2011	Chhattisgarh									605 (263-1201)		21 (9-42)		687 (299-1364)		24 (11-48)	
2012	Chhattisgarh									560 (244-1112)		20 (9-40)		636 (277-1263)		23 (10-45)	
2013	Chhattisgarh									539 (235-1069)		19 (8-38)		612 (266-1215)		22 (10-44)	
2014	Chhattisgarh									533 (232-1058)		19 (8-38)		605 (264-1202)		22 (10-43)	
2015	Chhattisgarh	82858 (71492-98495)	1162 (1002-1381)	3011 (2598-3580)	42 (36-50)	34070 (25525-38860)	478 (358-545)	1238 (928-1412)	17 (13-20)	548 (238-1087)	8 (4-16)	20 (9-40)	0 (0-1)	622 (271-1235)	9 (4-18)	23 (10-45)	0 (0-1)
2000	Delhi	64043 (55258-76130)		4083 (3523-4854)		26331 (19727-30033)		1679 (1258-1915)		288 (120-587)		18 (8-37)		328 (136-667)		21 (9-43)	
2001	Delhi									277 (115-564)		18 (7-36)		315 (131-641)		20 (8-41)	
2002	Delhi									267 (111-543)		17 (7-34)		303 (126-616)		19 (8-39)	
2003	Delhi									260 (107-525)		16 (7-33)		295 (122-597)		19 (8-38)	
2004	Delhi									257 (105-516)		16 (7-33)		292 (120-586)		18 (8-37)	
2005	Delhi									261 (117-505)		17 (7-32)		296 (133-574)		19 (8-36)	
2006	Delhi									267 (132-497)		17 (8-32)		304 (150-565)		19 (10-36)	
2007	Delhi									272 (150-484)		17 (10-31)		309 (171-550)		20 (11-35)	
2008	Delhi									269 (147-472)		17 (9-30)		306 (167-536)		19 (11-34)	
2009	Delhi									262 (141-455)		17 (9-29)		298 (160-516)		19 (10-33)	
2010	Delhi									254 (136-437)		16 (9-28)		289 (154-496)		19 (10-32)	
2011	Delhi									248 (131-422)		16 (9-27)		281 (149-479)		18 (10-31)	
2012	Delhi									244 (121-489)		16 (8-32)		277 (138-555)		18 (9-36)	
2013	Delhi									233 (115-462)		15 (8-31)		264 (130-525)		18 (9-35)	
2014	Delhi									212 (104-417)		14 (7-28)		241 (118-474)		16 (8-32)	
2015	Delhi	31176 (26899-37059)	437 (377-520)	2112 (1822-2511)	30 (26-35)	12813 (9599-14614)	180 (135-205)	868 (650-990)	12 (9-14)	187 (91-366)	3 (1-5)	13 (6-25)	0 (0-0)	213 (103-416)	3 (2-6)	14 (7-28)	0 (0-0)
2000	Goa	3930 (3391-4671)		3316 (2861-3942)		1613 (1209-1840)		1361 (1020-1553)		13 (6-26)		11 (5-22)		15 (7-30)		12 (6-25)	
2001	Goa									11 (5-22)		9 (4-18)		12 (6-25)		10 (5-21)	
2002	Goa									7 (3-15)		6 (3-13)		8 (4-17)		7 (3-15)	
2003	Goa									9 (4-19)		8 (4-16)		10 (5-21)		9 (4-18)	
2004	Goa									9 (4-19)		8 (4-16)		11 (5-22)		9 (4-18)	
2005	Goa									9 (4-19)		8 (3-16)		10 (5-22)		9 (4-19)	
2006	Goa									8 (3-17)		7 (3-14)		9 (4-19)		8 (3-16)	
2007	Goa									5 (2-11)		4 (2-10)		6 (2-13)		5 (2-11)	
2008	Goa									4 (2-10)		4 (2-8)		5 (2-11)		4 (2-9)	
2009	Goa									5 (2-11)		5 (2-10)		6 (2-13)		5 (2-11)	
2010	Goa									5 (2-11)		5 (2-10)		6 (3-13)		5 (2-11)	
2011	Goa									4 (2-9)		4 (2-8)		5 (2-10)		4 (2-9)	
2012	Goa									3 (2-7)		3 (1-6)		4 (2-7)		3 (2-7)	
2013	Goa									2 (1-5)		2 (1-4)		3 (1-6)		3 (1-5)	
2014	Goa									2 (1-4)		2 (1-3)		2 (1-4)		2 (1-4)	
2015	Goa	1761 (1519-2093)	25 (21-29)	1647 (1421-1957)	23 (20-27)	724 (542-826)	10 (8-12)	677 (507-772)	9 (7-11)	1 (1-3)	0 (0-0)	1 (1-2)	0 (0-0)	1 (1-3)	0 (0-0)	1 (1-3)	0 (0-0)
2000	Gujarat	255465 (220421-303676)		4274 (3688-5080)		105049 (78702-119819)		1757 (1317-2005)		1658 (727-3352)		28 (12-56)		665 (292-1344)		11 (5-22)	
2001	Gujarat									1591 (699-3223)		26 (12-53)		638 (280-1293)		11 (5-21)	
2002	Gujarat									1539 (677-3122)		25 (11-51)		617 (272-1252)		10 (4-21)	
2003	Gujarat									1492 (571-3117)		25 (9-51)		1695 (648-3540)		28 (11-58)	
2004	Gujarat									1442 (550-3004)		24 (9-49)		1638 (625-3412)		27 (10-56)	
2005	Gujarat									1454 (594-2966)		24 (10-48)		1651 (674-3368)		27 (11-55)	
2006	Gujarat									1486 (651-2956)		24 (11-48)		1688 (740-3357)		27 (12-55)	
2007	Gujarat									1473 (697-2849)		24 (11-46)		1672 (792-3235)		27 (13-53)	
2008	Gujarat									1437 (675-2757)		23 (11-45)		1632 (766-3131)		27 (12-51)	
2009	Gujarat									1361 (636-2599)		22 (10-42)		1545 (722-2952)		25 (12-48)	
2010	Gujarat									1264 (590-2411)		21 (10-39)		1435 (670-2738)		24 (11-45)	
2011	Gujarat									1175 (549-2241)		19 (9-37)		1335 (623-2546)		22 (10-42)	
2012	Gujarat									1095 (511-2088)		18 (8-35)		1243 (580-2371)		21 (10-39)	
2013	Gujarat									1044 (487-1991)		17 (8-33)		1186 (553-2262)		20 (9-38)	
2014	Gujarat									993 (464-1895)		17 (8-32)		1128 (527-2152)		19 (9-36)	
2015	Gujarat	219088 (189034-260434)	3072 (2651-3652)	3705 (3197-4404)	52 (45-62)	90023 (67445-102680)	1262 (946-1440)	1522 (1140-1736)	21 (16-24)	944 (441-1801)	14 (7-27)	16 (7-30)	0 (0-0)	1072 (501-2045)	16 (7-31)	18 (8-35)	0 (0-1)
2000	Haryana	100407 (86633-119356)		3893 (3359-4628)		41263 (30914-47065)		1600 (1199-1825)		1138 (564-2106)		44 (22-82)		457 (226-845)		18 (9-33)	

Spn = *Streptococcus pneumoniae*; npnm = non-pneumonia, non-meningitis; HIV = HIV-infected cases only; UR = uncertainty range

Year	State	Spn pneumonia cases (UR)	Spn pneumonia cases; HIV (UR)	Spn pneumonia incidence rate (UR)	Spn pneumonia incidence rate; HIV (UR)	Spn severe pneumonia cases (UR)	Spn severe pneumonia cases; HIV (UR)	Spn severe pneumonia incidence rate (UR)	Spn severe pneumonia incidence rate; HIV (UR)	Spn meningitis cases (UR)	Spn meningitis cases; HIV (UR)	Spn meningitis incidence rate (UR)	Spn meningitis incidence rate; HIV (UR)	Spn NPNM cases (UR)	Spn NPNM cases; HIV (UR)	Spn NPNM incidence rate (UR)	Spn NPNM incidence rate; HIV (UR)
2001	Haryana									1088 (540-2016)		42 (21-77)		437 (217-809)		17 (8-31)	
2002	Haryana									1042 (517-1932)		40 (20-74)		418 (208-775)		16 (8-30)	
2003	Haryana									1003 (498-1859)		38 (19-71)		402 (200-746)		15 (8-28)	
2004	Haryana									977 (438-1856)		37 (17-70)		1110 (498-2109)		42 (19-80)	
2005	Haryana									944 (449-1760)		36 (17-67)		1072 (510-1999)		41 (19-76)	
2006	Haryana									899 (454-1639)		34 (17-62)		1021 (515-1862)		39 (19-70)	
2007	Haryana									899 (484-1600)		34 (18-60)		1021 (550-1818)		38 (21-68)	
2008	Haryana									851 (455-1504)		32 (17-57)		966 (517-1708)		36 (19-64)	
2009	Haryana									760 (400-1345)		29 (15-51)		863 (454-1527)		33 (17-58)	
2010	Haryana									730 (379-1298)		28 (14-49)		829 (431-1474)		31 (16-56)	
2011	Haryana									679 (349-1215)		26 (13-46)		772 (396-1380)		29 (15-52)	
2012	Haryana									591 (300-1062)		23 (11-41)		671 (341-1207)		26 (13-46)	
2013	Haryana									596 (303-1073)		23 (12-41)		677 (344-1219)		26 (13-47)	
2014	Haryana									582 (296-1049)		23 (12-41)		661 (336-1191)		26 (13-46)	
2015	Haryana	65457 (56477-77809)	918 (792-1091)	2556 (2205-3038)	36 (31-43)	26910 (20161-30694)	377 (283-430)	1051 (787-1198)	15 (11-17)	588 (299-1059)	9 (4-16)	23 (12-41)	0 (0-1)	667 (340-1203)	10 (5-18)	26 (13-47)	0 (0-1)
2000	Himachal Pradesh	24292 (20960-28876)		3798 (3277-4515)		9981 (7478-11385)		1561 (1169-1780)		221 (102-427)		35 (16-67)		251 (115-484)		39 (18-76)	
2001	Himachal Pradesh									205 (94-394)		32 (15-61)		233 (107-448)		36 (17-70)	
2002	Himachal Pradesh									189 (87-365)		30 (14-57)		215 (99-414)		34 (15-65)	
2003	Himachal Pradesh									176 (81-339)		28 (13-53)		200 (92-385)		31 (14-60)	
2004	Himachal Pradesh									165 (75-317)		26 (12-50)		188 (86-360)		29 (13-57)	
2005	Himachal Pradesh									156 (74-296)		25 (12-47)		178 (84-336)		28 (13-53)	
2006	Himachal Pradesh									149 (72-278)		24 (11-44)		169 (82-316)		27 (13-50)	
2007	Himachal Pradesh									142 (71-261)		23 (11-42)		161 (81-297)		26 (13-47)	
2008	Himachal Pradesh									137 (67-249)		22 (11-40)		155 (77-282)		25 (12-45)	
2009	Himachal Pradesh									132 (68-235)		21 (11-38)		150 (77-267)		24 (12-43)	
2010	Himachal Pradesh									128 (68-222)		21 (11-36)		145 (78-253)		24 (13-41)	
2011	Himachal Pradesh									124 (69-211)		20 (11-35)		141 (79-240)		23 (13-40)	
2012	Himachal Pradesh									121 (66-200)		20 (11-33)		137 (75-228)		23 (12-38)	
2013	Himachal Pradesh									119 (64-196)		20 (11-33)		135 (73-222)		23 (12-38)	
2014	Himachal Pradesh									118 (63-193)		20 (11-33)		134 (72-219)		23 (12-38)	
2015	Himachal Pradesh	12921 (11149-15360)	181 (156-215)	2245 (1937-2669)	31 (27-37)	5317 (3983-6064)	75 (56-85)	924 (692-1054)	13 (10-15)	119 (64-195)	2 (1-3)	21 (11-34)	0 (0-1)	135 (73-222)	2 (1-3)	24 (13-39)	0 (0-1)
2000	Jammu & Kashmir	48920 (42209-58152)		4634 (3998-5508)		20117 (15071-22945)		1905 (1428-2173)		422 (220-672)		40 (21-64)		479 (250-763)		45 (24-72)	
2001	Jammu & Kashmir									396 (207-632)		36 (19-57)		450 (235-717)		41 (21-65)	
2002	Jammu & Kashmir									369 (193-590)		32 (17-51)		420 (220-670)		37 (19-58)	
2003	Jammu & Kashmir									340 (179-546)		28 (15-46)		386 (203-620)		32 (17-52)	
2004	Jammu & Kashmir									305 (163-497)		25 (13-40)		347 (185-565)		28 (15-46)	
2005	Jammu & Kashmir									269 (149-454)		21 (12-35)		305 (169-516)		24 (13-40)	
2006	Jammu & Kashmir									235 (124-412)		18 (9-31)		267 (140-468)		20 (10-35)	
2007	Jammu & Kashmir									208 (102-374)		15 (7-27)		236 (116-425)		17 (8-31)	
2008	Jammu & Kashmir									187 (93-340)		13 (6-24)		212 (105-386)		15 (7-27)	
2009	Jammu & Kashmir									169 (84-309)		11 (6-21)		192 (96-351)		13 (6-24)	
2010	Jammu & Kashmir									154 (77-282)		10 (5-18)		175 (87-320)		11 (6-21)	
2011	Jammu & Kashmir									139 (69-255)		9 (4-16)		158 (79-289)		10 (5-18)	
2012	Jammu & Kashmir									124 (62-226)		8 (4-14)		140 (70-257)		9 (4-16)	
2013	Jammu & Kashmir									111 (55-203)		7 (3-12)		126 (63-230)		8 (4-14)	
2014	Jammu & Kashmir									101 (50-184)		6 (3-11)		115 (57-210)		7 (3-12)	
2015	Jammu & Kashmir	29329 (25306-34864)	411 (355-489)	1665 (1437-1979)	23 (20-28)	12073 (9045-13770)	169 (127-193)	685 (513-782)	10 (7-11)	96 (48-175)	1 (1-3)	5 (3-10)	0 (0-0)	109 (54-199)	2 (1-3)	6 (3-11)	0 (0-0)
2000	Jharkhand	225360 (194446-267890)		6120 (5281-7275)		92684 (69438-105715)		2517 (1886-2871)		871 (366-1825)		24 (10-50)		349 (147-732)		9 (4-20)	
2001	Jharkhand									864 (313-1869)		23 (8-50)		982 (355-2123)		26 (9-57)	
2002	Jharkhand									844 (305-1827)		22 (8-48)		958 (347-2075)		25 (9-55)	
2003	Jharkhand									833 (302-1805)		22 (8-47)		946 (343-2050)		25 (9-53)	
2004	Jharkhand									789 (286-1709)		20 (7-44)		896 (325-1941)		23 (8-50)	
2005	Jharkhand									721 (264-1556)		18 (7-40)		818 (300-1768)		21 (8-45)	
2006	Jharkhand									717 (266-1544)		18 (7-39)		815 (302-1753)		21 (8-44)	
2007	Jharkhand									670 (251-1436)		17 (6-36)		761 (285-1631)		19 (7-41)	
2008	Jharkhand									602 (225-1290)		15 (6-32)		684 (256-1465)		17 (6-36)	
2009	Jharkhand									535 (200-1146)		13 (5-28)		608 (227-1302)		15 (6-32)	
2010	Jharkhand									541 (202-1159)		13 (5-29)		615 (230-1317)		15 (6-33)	
2011	Jharkhand									517 (193-1107)		13 (5-27)		587 (219-1257)		14 (5-31)	
2012	Jharkhand									481 (180-1029)		12 (4-25)		546 (204-1169)		13 (5-29)	
2013	Jharkhand									438 (164-939)		11 (4-23)		498 (186-1066)		12 (5-26)	
2014	Jharkhand									394 (147-844)		10 (4-21)		447 (167-958)		11 (4-24)	
2015	Jharkhand	141363 (121971-168041)	1982 (1710-2356)	3474 (2997-4129)	49 (42-58)	58105 (43532-66274)	815 (610-929)	1428 (1070-1628)	20 (15-23)	350 (131-750)	5 (2-11)	9 (3-18)	0 (0-0)	398 (149-852)	6 (2-13)	10 (4-21)	0 (0-0)
2000	Karnataka	249063 (214897-296066)		4435 (3827-5272)		102412 (76727-116811)		1824 (1366-2080)		1525 (656-3047)		27 (12-54)		1732 (745-3460)		31 (13-62)	
2001	Karnataka									1456 (626-2908)		26 (11-51)		1653 (711-3303)		29 (13-58)	
2002	Karnataka									1418 (610-2832)		25 (11-50)		1611 (693-3216)		28 (12-57)	
2003	Karnataka									1391 (597-2772)		24 (10-49)		1579 (678-3148)		28 (12-55)	
2004	Karnataka									1372 (586-2723)		24 (10-48)		1558 (666-3093)		27 (12-54)	
2005	Karnataka									1356 (602-2655)		24 (11-47)		1540 (683-3016)		27 (12-53)	
2006	Karnataka									1342 (618-2587)		23 (11-45)		1525 (702-2939)		27 (12-51)	
2007	Karnataka									1315 (630-2492)		23 (11-44)		1493 (715-2830)		26 (13-50)	
2008	Karnataka									1274 (605-2394)		22 (11-42)		1447 (687-2719)		25 (12-48)	
2009	Karnataka									1220 (594-2259)		21 (10-40)		1385 (675-2565)		24 (12-45)	
2010	Karnataka									1151 (576-2099)		20 (10-37)		1307 (654-2384)		23 (12-42)	
2011	Karnataka									1070 (551-1922)		19 (10-34)		1215 (626-2183)		22 (11-39)	
2012	Karnataka									957 (509-1696)		17 (9-30)		1087 (578-1926)		20 (10-35)	
2013	Karnataka									828 (438-1461)		15 (8-26)		941 (498-1659)		17 (9-30)	
2014	Karnataka									673 (355-1185)		12 (6-22)		764 (404-1345)		14 (7-25)	
2015	Karnataka	145701 (125714-173197)	2043 (1763-2429)	2682 (2314-3188)	38 (32-45)	59887 (44867-68306)	840 (629-958)	1102 (826-1257)	15 (12-18)	525 (277-923)	8 (4-14)	10 (5-17)	0 (0-0)	596 (315-1049)	9 (5-16)	11 (6-19)	0 (0-0)
2000	Kerala	102615 (88539-121981)		3221 (2779-3828)		42157 (31583-48084)		1323 (991-1509)		208 (80-468)		7 (3-15)		237 (91-531)		7 (3-17)	
2001	Kerala									201 (77-452)		6 (2-14)		228 (87-513)		7 (3-16)	
2002	Kerala									196 (75-439)		6 (2-14)		222 (85-499)		7 (3-16)	
2003	Kerala									194 (74-433)		6 (2-14)		220 (84-491)		7 (3-16)	
2004	Kerala									197 (74-435)		6 (2-14)		223 (84-494)		7 (3-16)	
2005	Kerala									206 (85-442)		7 (3-15)		234 (96-502)		8 (3-17)	
2006	Kerala									221 (100-456)		7 (3-15)		251 (114-518)		8 (4-17)	
2007	Kerala									236 (120-469)		8 (4-16)		268 (137-532)		9 (5-18)	
2008	Kerala									249 (125-485)		9 (4-17)		282 (142-551)		10 (5-19)	
2009	Kerala									254 (126-492)		9 (4-17)		289 (143-559)		10 (5-20)	
2010	Kerala									255 (125-493)		9 (4-18)		289 (142-559)		10 (5-20)	
2011	Kerala									253 (124-491)		9 (5-18)		288 (141-557)		11 (5-20)	
2012	Kerala									250 (122-485)		9 (5-18)		284 (139-551)		11 (5-21)	
2013	Kerala									251 (123-487)		10 (5-19)		285 (139-553)		11 (5-21)	
2014	Kerala									250 (122-486)		10 (5-19)		284 (139-552)		11 (5-22)	
2015	Kerala	27092 (23376-32205)	380 (328-452)	1085 (936-1290)	15 (13-18)	11137 (8344-12702)	156 (117-178)	446 (334-509)	6 (5-7)	253 (124-490)	4 (2-7)	10 (5-20)	0 (0-0)	287 (140-557)	4 (2-8)	11 (6-22)	0 (0-0)
2000	Madhya Pradesh	504250 (435078-599412)		6019 (5193-7154)		207363 (155355-236517)		2475 (1854-2823)		3775 (1573-7851)		45 (19-94)		1514 (631-3149)		18 (8-38)	
2001	Madhya Pradesh									3614 (1506-7514)		43 (18-89)		1449 (604-3014)		17 (7-36)	
2002	Madhya Pradesh									3472 (1446-7216)		41 (17-85)		1393 (580-2894)		16 (7-34)	
2003	Madhya Pradesh									3346 (1392-6944)		39 (16-82)		1342 (558-2785)		16 (7-33)	
2004	Madhya Pradesh									3241 (1344-6704)		38 (16-79)		1300 (539-2689)		15 (6-32)	
2005	Madhya Pradesh									3168 (1353-6473)		37 (16-76)		1271 (543-2596)		15 (6-31)	
2006	Madhya Pradesh									3101 (1366-6252)		37 (16-74)		1244 (548-2508)		15 (6-30)	
2007	Madhya Pradesh									3015 (1372-5997)		36 (16-71)		1209 (550-2406)		14 (6-28)	
2008	Madhya Pradesh									2901 (1315-5750)		34 (16-68)		1164 (528-2306)		14 (6-27)	
2009	Madhya Pradesh									2756 (1247-5451)		33 (15-65)		1105 (500-2186)		13 (6-26)	
2010	Madhya Pradesh									2608 (1181-5161)		31 (14-62)		1046 (474-2070)		12 (6-25)	
2011	Madhya Pradesh									2477 (1123-4911)		30 (13-59)		994 (451-1970)		12 (5-24)	
2012	Madhya Pradesh									2353 (1070-4680)		29 (13-57)		944 (429-1877)		11 (5-23)	
2013	Madhya Pradesh									2285 (915-4690)		28 (11-57)		2596 (1039-5326)		32 (13-65)	
2014	Madhya Pradesh									2202 (884-4532)		27 (11-56)		2501 (1004-5148)		31 (12-64)	
2015	Madhya Pradesh	342550 (295559-407195)	4803 (4144-5710)	4271 (3685-5077)	60 (52-71)	140802 (105488-160599)	1974 (1479-2252)	1755 (1315-2002)	25 (18-28)	2131 (857-4395)	32 (13-66)	27 (11-55)	0 (0-1)	2420 (974-4991)	36 (15-74)	30 (12-62)	0 (0-1)
2000	Maharashtra	480040 (414189-570632)		4415 (3809-5248)		197311 (147824-225052)		1815 (1360-2070)		2376 (1087-4584)		22 (10-42)		2698 (1235-5207)		25 (11-48)	
2001	Maharashtra									2217 (1015-4277)		20 (9-39)		2518 (1152-4858)		23 (11-45)	
2002	Maharashtra									2063 (944-3979)		19 (9-36)		2344 (1072-4519)		21 (10-41)	
2003	Maharashtra									1921 (877-3696)		18 (8-34)		2181 (996-4198)		20 (9-39)	
2004	Maharashtra									1797 (817-3444)		17 (8-32)		2041 (928-3912)		19 (9-36)	
2005	Maharashtra									1695 (798-3205)		16 (7-30)		1925 (907-3641)		18 (8-34)	
2006	Maharashtra									1597 (779-2974)		15 (7-28)		1814 (885-3377)		17 (8-31)	
2007	Maharashtra									1488 (754-2730)		14 (7-25)		1690 (856-3101)		16 (8-29)	
2008	Maharashtra									1376 (693-2511)		13 (6-23)		1562 (788-2852)		15 (7-27)	
2009	Maharashtra									1265 (635-2300)		12 (6-22)		1437 (722-2612)		14 (7-25)	
2010	Maharashtra									1165 (583-2108)		11 (6-20)		1323 (662-2394)		13 (6-23)	
2011	Maharashtra									1107 (551-1991)		11 (5-19)		1257 (626-2262)		12 (6-22)	
2012	Maharashtra									1048 (458-2247)		10 (4-22)		1190 (521-2552)		12 (5-25)	
2013	Maharashtra									970 (421-2066)		10 (4-20)		1102 (479-2346)		11 (5-23)	
2014	Maharashtra									869 (375-1841)		9 (4-18)		987 (426-2091)		10 (4-21)	
2015	Maharashtra	315333 (272077-374843)	4421 (3815-5256)	3178 (2742-3777)	45 (38-53)	129622 (97112-147847)	1818 (1362-2073)	1306 (979-1490)	18 (14-21)	774 (333-1634)	12 (5-24)	8 (3-16)	0 (0-0)	879 (379-1856)	13 (6-28)	9 (4-19)	0 (0-0)
2000	Manipur	9160 (7903-10888)		4008 (3458-4764)		3763 (2819-4292)		1646 (1234-1878)		22 (8-49)		10 (3-21)		25 (9-55)		11 (4-24)	
2001	Manipur									22 (8-47)		9 (3-20)		24 (9-54)		10 (4-23)	
2002	Manipur									21 (7-46)		9 (3-19)		24 (8-52)		10 (3-22)	
2003	Manipur									20 (5-54)		8 (2-22)		23 (6-61)		9 (2-25)	

Spn = *Streptococccus pneumoniae*; npnm = non-pneumonia, non-meningitis; HIV = HIV-infected cases only; UR = uncertainty range

Year	State	Spn pneumonia cases (UR)	Spn pneumonia cases; HIV (UR)	Spn pneumonia incidence rate (UR)	Spn pneumonia incidence rate; HIV (UR)	Spn severe pneumonia cases (UR)	Spn severe pneumonia cases; HIV (UR)	Spn severe pneumonia incidence rate (UR)	Spn severe pneumonia incidence rate; HIV (UR)	Spn meningitis cases (UR)	Spn meningitis cases; HIV (UR)	Spn meningitis incidence rate (UR)	Spn meningitis incidence rate; HIV (UR)	Spn NPNM cases (UR)	Spn NPNM cases; HIV (UR)	Spn NPNM incidence rate (UR)	Spn NPNM incidence rate; HIV (UR)
2004	Manipur									20 (5-52)		8 (2-21)		23 (6-59)		9 (2-24)	
2005	Manipur									19 (5-51)		8 (2-20)		22 (5-58)		9 (2-23)	
2006	Manipur									19 (4-50)		7 (2-19)		21 (5-57)		8 (2-22)	
2007	Manipur									19 (4-50)		7 (2-19)		21 (5-57)		8 (2-21)	
2008	Manipur									19 (4-50)		7 (2-18)		22 (5-57)		8 (2-21)	
2009	Manipur									20 (5-52)		7 (2-19)		22 (5-59)		8 (2-21)	
2010	Manipur									21 (5-54)		7 (2-19)		23 (6-61)		8 (2-22)	
2011	Manipur									21 (6-55)		8 (2-19)		24 (7-63)		9 (2-22)	
2012	Manipur									22 (6-56)		8 (2-19)		25 (7-64)		9 (2-22)	
2013	Manipur									22 (6-57)		8 (2-19)		25 (7-65)		9 (2-22)	
2014	Manipur									22 (6-56)		7 (2-19)		25 (7-63)		8 (2-21)	
2015	Manipur	3242 (2797-3854)	45 (39-54)	1082 (933-1286)	15 (13-18)	1332 (998-1520)	19 (14-21)	445 (333-507)	6 (5-7)	21 (6-54)	0 (0-1)	7 (2-18)	0 (0-0)	24 (7-61)	0 (0-1)	8 (2-20)	0 (0-0)
2000	Meghalaya	14454 (12472-17182)		4000 (3451-4755)		5940 (4450-6775)		1644 (1232-1875)		150 (74-277)		41 (20-77)		60 (30-111)		17 (8-31)	
2001	Meghalaya									156 (77-288)		42 (21-78)		62 (31-116)		17 (8-31)	
2002	Meghalaya									162 (80-300)		43 (21-79)		65 (32-121)		17 (8-32)	
2003	Meghalaya									169 (83-313)		43 (21-80)		68 (33-125)		17 (9-32)	
2004	Meghalaya									177 (87-326)		44 (22-82)		71 (35-131)		18 (9-33)	
2005	Meghalaya									185 (93-338)		46 (23-83)		74 (37-136)		18 (9-33)	
2006	Meghalaya									193 (99-349)		46 (24-84)		77 (40-140)		19 (10-34)	
2007	Meghalaya									198 (104-356)		47 (25-84)		79 (42-143)		19 (10-34)	
2008	Meghalaya									199 (105-360)		46 (24-83)		80 (42-144)		18 (10-33)	
2009	Meghalaya									192 (96-356)		44 (22-81)		77 (39-143)		18 (9-33)	
2010	Meghalaya									189 (90-361)		42 (20-81)		76 (36-145)		17 (8-32)	
2011	Meghalaya									185 (84-362)		41 (19-80)		74 (34-145)		16 (7-32)	
2012	Meghalaya									182 (79-363)		40 (17-79)		73 (32-146)		16 (7-32)	
2013	Meghalaya									184 (81-370)		40 (17-80)		74 (32-149)		16 (7-32)	
2014	Meghalaya									188 (82-378)		40 (18-81)		75 (33-152)		16 (7-32)	
2015	Meghalaya	11151 (9622-13256)	156 (135-186)	2351 (2028-2795)	33 (28-39)	4585 (3435-5230)	64 (48-73)	967 (724-1103)	14 (10-15)	193 (85-390)	3 (1-6)	41 (18-82)	1 (0-1)	78 (34-156)	1 (1-2)	16 (7-33)	0 (0-0)
2000	Mizoram	3966 (3422-4714)		3519 (3037-4184)		1629 (1221-1858)		1446 (1083-1649)		34 (14-71)		30 (12-63)		14 (5-28)		12 (5-25)	
2001	Mizoram									33 (13-69)		28 (11-59)		13 (5-28)		11 (5-24)	
2002	Mizoram									32 (13-67)		27 (11-57)		13 (5-27)		11 (4-23)	
2003	Mizoram									32 (13-67)		26 (11-55)		13 (5-27)		11 (4-22)	
2004	Mizoram									31 (13-66)		26 (10-54)		13 (5-26)		10 (4-22)	
2005	Mizoram									31 (13-65)		25 (10-53)		13 (5-26)		10 (4-21)	
2006	Mizoram									31 (13-65)		25 (10-52)		13 (5-26)		10 (4-21)	
2007	Mizoram									32 (13-66)		25 (10-51)		13 (5-26)		10 (4-20)	
2008	Mizoram									32 (13-66)		24 (10-50)		13 (5-26)		10 (4-20)	
2009	Mizoram									32 (14-66)		24 (10-50)		13 (5-26)		10 (4-20)	
2010	Mizoram									32 (14-65)		24 (10-49)		13 (6-26)		10 (4-20)	
2011	Mizoram									33 (14-66)		24 (11-49)		13 (6-27)		10 (4-20)	
2012	Mizoram									33 (15-66)		24 (11-49)		13 (6-27)		10 (4-20)	
2013	Mizoram									33 (15-67)		24 (11-49)		13 (6-27)		10 (4-20)	
2014	Mizoram									33 (15-67)		24 (11-48)		13 (6-27)		10 (4-19)	
2015	Mizoram	1240 (1070-1474)	17 (15-21)	890 (768-1058)	12 (11-15)	509 (382-581)	7 (5-8)	366 (274-417)	5 (4-6)	33 (13-68)	0 (0-1)	24 (9-49)	0 (0-1)	37 (15-77)	1 (0-1)	27 (11-56)	0 (0-1)
2000	Nagaland	8088 (6978-9614)		3804 (3282-4522)		3323 (2490-3791)		1563 (1171-1783)		23 (8-50)		11 (4-24)		26 (9-57)		12 (4-27)	
2001	Nagaland									24 (8-53)		11 (4-25)		27 (10-61)		13 (4-28)	
2002	Nagaland									26 (9-57)		12 (4-26)		29 (10-65)		13 (5-30)	
2003	Nagaland									27 (9-60)		13 (4-28)		31 (11-69)		14 (5-31)	
2004	Nagaland									28 (10-63)		13 (5-29)		32 (11-72)		15 (5-33)	
2005	Nagaland									29 (10-65)		13 (4-29)		33 (11-73)		15 (5-33)	
2006	Nagaland									29 (9-65)		13 (4-29)		33 (11-74)		15 (5-33)	
2007	Nagaland									28 (9-64)		13 (4-29)		32 (10-73)		14 (5-33)	
2008	Nagaland									28 (9-64)		12 (4-29)		31 (10-72)		14 (4-33)	
2009	Nagaland									26 (8-61)		12 (4-27)		30 (9-69)		13 (4-31)	
2010	Nagaland									26 (8-62)		12 (4-28)		30 (9-70)		14 (4-32)	
2011	Nagaland									26 (7-60)		12 (3-27)		29 (8-69)		13 (4-31)	
2012	Nagaland									24 (7-58)		11 (3-27)		28 (8-66)		13 (4-30)	
2013	Nagaland									23 (7-56)		11 (3-26)		27 (8-64)		12 (3-29)	
2014	Nagaland									22 (6-53)		10 (3-24)		25 (7-60)		12 (3-28)	
2015	Nagaland	4250 (3667-5052)	60 (51-71)	1978 (1707-2351)	28 (24-33)	1747 (1309-1993)	25 (18-28)	813 (609-928)	11 (9-13)	21 (6-49)	0 (0-1)	10 (3-23)	0 (0-0)	23 (7-56)	0 (0-1)	11 (3-26)	0 (0-0)
2000	Odisha	193600 (167042-230136)		4769 (4114-5668)		79555 (59602-90740)		1960 (1468-2235)		1937 (879-3819)		48 (22-94)		777 (353-1532)		19 (9-38)	
2001	Odisha									1830 (831-3608)		45 (20-88)		734 (333-1447)		18 (8-35)	
2002	Odisha									1721 (781-3395)		42 (19-83)		690 (313-1362)		17 (8-33)	
2003	Odisha									1622 (737-3200)		39 (18-78)		651 (295-1284)		16 (7-31)	
2004	Odisha									1521 (691-3003)		37 (17-73)		610 (277-1205)		15 (7-29)	
2005	Odisha									1423 (642-2819)		34 (16-68)		571 (258-1131)		14 (6-27)	
2006	Odisha									1329 (596-2642)		32 (14-64)		533 (239-1060)		13 (6-26)	
2007	Odisha									1230 (547-2453)		30 (13-59)		494 (220-984)		12 (5-24)	
2008	Odisha									1146 (511-2289)		28 (12-55)		460 (205-918)		11 (5-22)	
2009	Odisha									1066 (476-2133)		26 (12-52)		427 (191-856)		10 (5-21)	
2010	Odisha									995 (446-1999)		24 (11-49)		399 (179-802)		10 (4-20)	
2011	Odisha									938 (369-1945)		23 (9-48)		1066 (419-2210)		26 (10-54)	
2012	Odisha									873 (344-1815)		22 (9-45)		991 (390-2061)		25 (10-51)	
2013	Odisha									815 (322-1698)		20 (8-42)		925 (365-1928)		23 (9-48)	
2014	Odisha									748 (295-1559)		19 (7-39)		849 (336-1771)		21 (8-45)	
2015	Odisha	112067 (96694-133217)	1571 (1356-1868)	2848 (2457-3386)	40 (34-47)	46074 (34518-52552)	646 (484-737)	1171 (877-1336)	16 (12-19)	684 (270-1426)	10 (4-21)	17 (7-36)	0 (0-1)	776 (307-1620)	12 (5-24)	20 (8-41)	0 (0-1)
2000	Punjab	106762 (92116-126910)		4406 (3801-5237)		43878 (32873-50047)		1811 (1357-2065)		958 (502-1693)		40 (21-70)		1088 (571-1922)		45 (24-79)	
2001	Punjab									894 (469-1580)		37 (19-65)		1016 (533-1794)		42 (22-74)	
2002	Punjab									835 (438-1474)		34 (18-60)		949 (497-1674)		39 (20-69)	
2003	Punjab									784 (410-1380)		32 (17-56)		890 (465-1568)		36 (19-64)	
2004	Punjab									741 (385-1298)		30 (16-53)		841 (437-1474)		34 (18-60)	
2005	Punjab									705 (379-1217)		29 (16-50)		801 (431-1382)		33 (18-57)	
2006	Punjab									672 (374-1141)		28 (15-47)		764 (425-1296)		31 (17-53)	
2007	Punjab									636 (348-1061)		26 (14-44)		722 (396-1205)		30 (16-49)	
2008	Punjab									593 (323-984)		24 (13-41)		674 (367-1118)		28 (15-46)	
2009	Punjab									543 (295-899)		23 (12-37)		617 (335-1021)		26 (14-42)	
2010	Punjab									492 (268-816)		21 (11-34)		559 (304-927)		23 (13-39)	
2011	Punjab									457 (249-757)		19 (10-32)		520 (282-860)		22 (12-36)	
2012	Punjab									434 (235-714)		18 (10-30)		493 (266-811)		21 (11-34)	
2013	Punjab									424 (227-692)		18 (10-30)		482 (258-786)		21 (11-34)	
2014	Punjab									430 (240-820)		19 (10-36)		489 (272-932)		21 (12-41)	
2015	Punjab	45222 (39018-53756)	634 (547-754)	1983 (1711-2358)	28 (24-33)	18608 (13941-21224)	261 (195-298)	816 (611-931)	11 (9-13)	440 (242-827)	7 (4-12)	19 (11-36)	0 (0-1)	500 (275-940)	7 (4-14)	22 (12-41)	0 (0-1)
2000	Rajasthan	494142 (426356-587396)		6006 (5182-7139)		203221 (152252-231793)		2470 (1850-2817)		3586 (1654-6994)		44 (20-85)		1438 (663-2805)		17 (8-34)	
2001	Rajasthan									3436 (1585-6702)		41 (19-81)		1378 (636-2688)		17 (8-32)	
2002	Rajasthan									3289 (1516-6413)		40 (18-77)		1319 (608-2572)		16 (7-31)	
2003	Rajasthan									3134 (1443-6104)		38 (17-73)		1257 (579-2448)		15 (7-29)	
2004	Rajasthan									2989 (1373-5805)		36 (17-70)		1199 (551-2328)		14 (7-28)	
2005	Rajasthan									2852 (1335-5492)		34 (16-66)		1144 (536-2203)		14 (6-27)	
2006	Rajasthan									2729 (1304-5211)		33 (16-63)		1094 (523-2090)		13 (6-25)	
2007	Rajasthan									2605 (1273-4940)		31 (15-59)		1045 (511-1981)		13 (6-24)	
2008	Rajasthan									2485 (1215-4713)		30 (15-57)		997 (487-1890)		12 (6-23)	
2009	Rajasthan									2360 (1039-4610)		29 (13-56)		2681 (1180-5236)		33 (14-64)	
2010	Rajasthan									2216 (978-4341)		27 (12-53)		2517 (1111-4931)		31 (14-60)	
2011	Rajasthan									2062 (913-4050)		25 (11-50)		2343 (1036-4600)		29 (13-57)	
2012	Rajasthan									1885 (835-3707)		23 (10-46)		2141 (949-4210)		27 (12-52)	
2013	Rajasthan									1737 (770-3416)		22 (10-43)		1973 (874-3880)		25 (11-49)	
2014	Rajasthan									1601 (709-3149)		20 (9-40)		1819 (806-3576)		23 (10-45)	
2015	Rajasthan	301982 (260557-358972)	4234 (3653-5033)	3859 (3329-4587)	54 (47-64)	124154 (93015-141610)	1741 (1304-1986)	1586 (1189-1809)	22 (17-25)	1506 (667-2960)	22 (10-44)	19 (9-38)	0 (0-1)	1710 (758-3362)	26 (11-50)	22 (10-43)	0 (0-1)
2000	Sikkim	1977 (1706-2350)		3253 (2807-3867)		812 (609-927)		1337 (1001-1525)		13 (6-25)		21 (10-40)		14 (7-28)		24 (11-46)	
2001	Sikkim									12 (5-23)		20 (9-38)		14 (6-26)		23 (10-44)	
2002	Sikkim									11 (5-21)		19 (9-36)		12 (6-24)		21 (10-41)	
2003	Sikkim									10 (5-19)		17 (8-33)		11 (5-22)		19 (9-38)	
2004	Sikkim									8 (4-17)		15 (7-30)		10 (5-19)		17 (8-34)	
2005	Sikkim									7 (3-15)		13 (5-26)		8 (3-17)		15 (6-30)	
2006	Sikkim									6 (2-13)		11 (4-24)		7 (3-14)		13 (5-27)	
2007	Sikkim									5 (2-11)		10 (3-22)		6 (2-13)		11 (4-25)	
2008	Sikkim									5 (2-10)		9 (3-20)		5 (2-12)		11 (4-23)	
2009	Sikkim									5 (2-10)		9 (3-20)		5 (2-11)		11 (4-23)	
2010	Sikkim									5 (2-10)		10 (4-21)		5 (2-11)		11 (4-23)	
2011	Sikkim									5 (2-10)		11 (4-22)		6 (2-12)		12 (5-25)	
2012	Sikkim									5 (2-11)		11 (5-23)		6 (3-12)		13 (6-26)	
2013	Sikkim									6 (2-13)		13 (4-30)		6 (2-15)		14 (5-34)	
2014	Sikkim									6 (2-14)		13 (5-32)		7 (2-16)		15 (5-36)	
2015	Sikkim	350 (302-417)	5 (4-6)	843 (728-1003)	12 (10-14)	144 (108-164)	2 (2-2)	346 (259-395)	5 (4-6)	6 (2-14)	0 (0-0)	14 (5-34)	0 (0-1)	7 (2-16)	0 (0-0)	16 (5-39)	0 (0-1)
2000	Tamil Nadu	169832 (146535-201883)		2936 (2533-3490)		69746 (52253-79552)		1206 (903-1375)		1140 (524-2195)		20 (9-38)		1295 (595-2493)		22 (10-43)	
2001	Tamil Nadu									1084 (498-2087)		19 (9-36)		1232 (566-2371)		21 (10-41)	
2002	Tamil Nadu									1029 (473-1979)		18 (8-34)		1169 (537-2248)		20 (9-38)	
2003	Tamil Nadu									978 (448-1878)		17 (8-32)		1111 (509-2132)		19 (9-36)	
2004	Tamil Nadu									933 (426-1784)		16 (7-30)		1059 (484-2026)		18 (8-34)	
2005	Tamil Nadu									892 (421-1684)		15 (7-28)		1013 (479-1913)		17 (8-32)	
2006	Tamil Nadu									851 (416-1584)		14 (7-27)		966 (472-1799)		16 (8-30)	

Spn = *Streptococccus pneumoniae*; npnm = non-pneumonia, non-meningitis; HIV = HIV-infected cases only; UR = uncertainty range

Year	State	Spn pneumonia cases (UR)	Spn pneumonia cases; HIV (UR)	Spn pneumonia incidence rate (UR)	Spn pneumonia incidence rate; HIV (UR)	Spn severe pneumonia cases (UR)	Spn severe pneumonia cases; HIV (UR)	Spn severe pneumonia incidence rate (UR)	Spn severe pneumonia incidence rate; HIV (UR)	Spn meningitis cases (UR)	Spn meningitis cases; HIV (UR)	Spn meningitis incidence rate (UR)	Spn meningitis incidence rate; HIV (UR)	Spn NPNM cases (UR)	Spn NPNM cases; HIV (UR)	Spn NPNM incidence rate (UR)	Spn NPNM incidence rate; HIV (UR)
2007	Tamil Nadu									800 (406-1470)		13 (7-25)		909 (461-1670)		15 (8-28)	
2008	Tamil Nadu									753 (381-1381)		13 (6-23)		856 (433-1568)		14 (7-26)	
2009	Tamil Nadu									706 (347-1302)		12 (6-22)		802 (394-1479)		14 (7-25)	
2010	Tamil Nadu									665 (318-1236)		11 (5-21)		755 (361-1404)		13 (6-24)	
2011	Tamil Nadu									636 (246-1435)		11 (4-24)		723 (280-1630)		12 (5-28)	
2012	Tamil Nadu									596 (221-1354)		10 (4-23)		676 (251-1537)		12 (4-26)	
2013	Tamil Nadu									560 (208-1274)		10 (4-22)		636 (236-1447)		11 (4-25)	
2014	Tamil Nadu									522 (194-1188)		9 (3-21)		593 (220-1350)		10 (4-24)	
2015	Tamil Nadu	75992 (65567-90333)	1066 (919-1267)	1330 (1147-1581)	19 (16-22)	31226 (23394-35616)	438 (328-499)	546 (409-623)	8 (6-9)	495 (184-1129)	7 (3-17)	9 (3-20)	0 (0-0)	563 (209-1282)	8 (3-19)	10 (4-22)	0 (0-0)
2000	Tripura	15138 (13061-17995)		4557 (3932-5417)		6217 (4658-7091)		1871 (1402-2134)		51 (20-107)		15 (6-32)		58 (22-121)		17 (7-36)	
2001	Tripura									46 (18-96)		14 (5-28)		52 (20-109)		15 (6-32)	
2002	Tripura									42 (16-88)		12 (5-26)		47 (18-100)		14 (5-29)	
2003	Tripura									38 (15-80)		11 (4-23)		43 (17-91)		13 (5-26)	
2004	Tripura									35 (14-74)		10 (4-21)		40 (16-84)		12 (4-24)	
2005	Tripura									34 (13-69)		10 (4-20)		38 (15-79)		11 (4-23)	
2006	Tripura									32 (13-65)		9 (4-19)		36 (15-74)		10 (4-21)	
2007	Tripura									31 (14-63)		9 (4-18)		36 (15-72)		10 (4-20)	
2008	Tripura									31 (13-61)		9 (4-17)		35 (15-69)		10 (4-19)	
2009	Tripura									30 (14-59)		8 (4-16)		35 (16-67)		10 (4-19)	
2010	Tripura									31 (15-57)		9 (4-16)		35 (17-65)		10 (5-18)	
2011	Tripura									31 (16-57)		9 (4-16)		36 (18-65)		10 (5-18)	
2012	Tripura									31 (17-56)		9 (5-15)		36 (19-63)		10 (5-18)	
2013	Tripura									31 (16-54)		9 (5-15)		35 (19-61)		10 (5-17)	
2014	Tripura									30 (15-61)		8 (4-17)		34 (17-70)		10 (5-20)	
2015	Tripura	7876 (6795-9362)	110 (95-131)	2205 (1903-2621)	31 (27-37)	3240 (2427-3695)	45 (34-52)	907 (679-1034)	13 (10-15)	28 (13-56)	0 (0-1)	8 (4-16)	0 (0-0)	32 (15-64)	0 (0-1)	9 (4-18)	0 (0-0)
2000	Union Territory	10882 (9390-12936)		3914 (3377-4652)		4472 (3350-5101)		1608 (1205-1834)		54 (26-103)		20 (9-37)		62 (30-117)		22 (11-42)	
2001	Union Territory									52 (25-99)		19 (9-35)		60 (29-112)		21 (10-40)	
2002	Union Territory									51 (25-96)		18 (9-34)		58 (28-109)		20 (10-38)	
2003	Union Territory									50 (23-99)		18 (8-35)		57 (26-113)		20 (9-39)	
2004	Union Territory									50 (23-99)		17 (8-34)		57 (26-113)		20 (9-39)	
2005	Union Territory									51 (24-99)		18 (8-34)		58 (28-113)		20 (10-39)	
2006	Union Territory									54 (28-100)		18 (10-34)		61 (32-114)		21 (11-39)	
2007	Union Territory									58 (34-102)		20 (12-35)		66 (39-116)		22 (13-39)	
2008	Union Territory									62 (36-104)		21 (12-35)		70 (40-118)		24 (14-40)	
2009	Union Territory									63 (35-102)		21 (12-35)		72 (40-116)		24 (13-39)	
2010	Union Territory									62 (34-99)		21 (11-34)		71 (38-113)		24 (13-38)	
2011	Union Territory									60 (33-98)		20 (11-33)		68 (38-111)		23 (13-38)	
2012	Union Territory									57 (31-94)		19 (11-32)		65 (35-107)		22 (12-36)	
2013	Union Territory									54 (29-89)		18 (10-30)		61 (33-101)		21 (11-34)	
2014	Union Territory									49 (26-81)		17 (9-28)		55 (30-92)		19 (10-31)	
2015	Union Territory	5637 (4864-6701)	79 (68-94)	1925 (1661-2288)	27 (23-32)	2316 (1735-2642)	32 (24-37)	791 (593-902)	11 (8-13)	43 (23-73)	1 (0-1)	15 (8-25)	0 (0-0)	49 (26-83)	1 (0-1)	17 (9-28)	0 (0-0)
2000	Uttar Pradesh	1397037 (1205394-1660684)		5911 (5101-7027)		574511 (430420-655285)		2431 (1821-2773)		14874 (7467-27234)		63 (32-115)		5966 (2995-10923)		25 (13-46)	
2001	Uttar Pradesh									14271 (7164-26129)		60 (30-110)		5724 (2874-10480)		24 (12-44)	
2002	Uttar Pradesh									13638 (6849-24978)		57 (29-105)		5470 (2747-10019)		23 (12-42)	
2003	Uttar Pradesh									12977 (6522-23785)		55 (28-100)		5205 (2616-9540)		22 (11-40)	
2004	Uttar Pradesh									12257 (6170-22501)		52 (26-95)		4916 (2475-9025)		21 (10-38)	
2005	Uttar Pradesh									11480 (5718-21183)		49 (24-90)		4605 (2294-8497)		20 (10-36)	
2006	Uttar Pradesh									10717 (5284-19879)		46 (22-85)		4299 (2119-7974)		18 (9-34)	
2007	Uttar Pradesh									9959 (4860-18565)		43 (21-79)		3995 (1949-7446)		17 (8-32)	
2008	Uttar Pradesh									9306 (4553-17390)		40 (20-75)		3732 (1826-6975)		16 (8-30)	
2009	Uttar Pradesh									8737 (4286-16373)		38 (19-71)		3504 (1719-6567)		15 (7-28)	
2010	Uttar Pradesh									8233 (4054-15483)		36 (18-68)		3302 (1626-6210)		14 (7-27)	
2011	Uttar Pradesh									7796 (3471-15113)		34 (15-67)		8854 (3942-17166)		39 (17-76)	
2012	Uttar Pradesh									7245 (3237-14095)		32 (14-63)		8229 (3676-16009)		37 (16-71)	
2013	Uttar Pradesh									6759 (3027-13180)		31 (14-60)		7677 (3438-14970)		35 (16-68)	
2014	Uttar Pradesh									6233 (2795-12171)		29 (13-56)		7079 (3175-13824)		32 (15-63)	
2015	Uttar Pradesh	868357 (749238-1032232)	12176 (10506-14474)	4018 (3467-4777)	56 (49-67)	356899 (267387-407077)	5004 (3749-5708)	1652 (1237-1884)	23 (17-26)	5753 (2580-11233)	86 (38-168)	27 (12-52)	0 (0-1)	6534 (2930-12759)	97 (44-190)	30 (14-59)	0 (0-1)
2000	Uttarakhand	62043 (53532-73752)		5911 (5101-7027)		25514 (19115-29102)		2431 (1821-2773)		332 (167-612)		32 (16-58)		133 (67-245)		13 (6-23)	
2001	Uttarakhand									322 (162-594)		31 (15-56)		129 (65-238)		12 (6-23)	
2002	Uttarakhand									311 (157-575)		29 (15-54)		125 (63-231)		12 (6-22)	
2003	Uttarakhand									298 (137-571)		28 (13-54)		339 (156-648)		32 (15-61)	
2004	Uttarakhand									281 (130-542)		27 (12-51)		319 (148-615)		30 (14-58)	
2005	Uttarakhand									258 (114-509)		24 (11-48)		293 (130-578)		28 (12-55)	
2006	Uttarakhand									235 (99-472)		22 (9-45)		266 (113-536)		25 (11-51)	
2007	Uttarakhand									212 (86-434)		20 (8-41)		241 (97-493)		23 (9-47)	
2008	Uttarakhand									194 (79-398)		18 (7-38)		220 (89-452)		21 (8-43)	
2009	Uttarakhand									178 (72-367)		17 (7-35)		202 (82-416)		19 (8-40)	
2010	Uttarakhand									164 (66-337)		16 (6-32)		186 (75-382)		18 (7-37)	
2011	Uttarakhand									149 (60-307)		14 (6-30)		169 (69-348)		16 (7-34)	
2012	Uttarakhand									132 (54-272)		13 (5-27)		150 (61-309)		15 (6-30)	
2013	Uttarakhand									118 (48-242)		12 (5-24)		133 (54-275)		13 (5-27)	
2014	Uttarakhand									103 (42-213)		10 (4-21)		118 (48-242)		12 (5-24)	
2015	Uttarakhand	24309 (20975-28897)	341 (294-405)	2459 (2122-2923)	34 (30-41)	10000 (7492-11406)	140 (105-160)	1011 (758-1154)	14 (11-16)	92 (37-189)	1 (1-3)	9 (4-19)	0 (0-0)	104 (42-214)	2 (1-3)	11 (4-22)	0 (0-0)
2000	West Bengal	429118 (370253-510101)		4930 (4254-5860)		176361 (132128-201156)		2026 (1518-2311)		2815 (1335-5319)		32 (15-61)		3198 (1516-6041)		37 (17-69)	
2001	West Bengal									2568 (1217-4851)		29 (14-56)		2916 (1383-5510)		33 (16-63)	
2002	West Bengal									2309 (1095-4362)		27 (13-50)		2623 (1243-4954)		30 (14-57)	
2003	West Bengal									2084 (988-3936)		24 (11-45)		2367 (1122-4470)		27 (13-52)	
2004	West Bengal									1899 (899-3583)		22 (10-42)		2157 (1021-4070)		25 (12-47)	
2005	West Bengal									1744 (832-3281)		20 (10-38)		1981 (944-3726)		23 (11-43)	
2006	West Bengal									1606 (771-3011)		19 (9-35)		1824 (876-3420)		21 (10-40)	
2007	West Bengal									1477 (714-2760)		17 (8-32)		1677 (811-3135)		20 (10-37)	
2008	West Bengal									1345 (649-2509)		16 (8-30)		1528 (737-2850)		18 (9-34)	
2009	West Bengal									1218 (592-2262)		15 (7-27)		1383 (673-2569)		17 (8-31)	
2010	West Bengal									1136 (557-2101)		14 (7-25)		1291 (633-2387)		16 (8-29)	
2011	West Bengal									1095 (542-2017)		13 (7-25)		1244 (615-2291)		15 (8-28)	
2012	West Bengal									1056 (527-1937)		13 (7-24)		1200 (599-2200)		15 (7-27)	
2013	West Bengal									1051 (524-1925)		13 (7-24)		1193 (595-2186)		15 (8-28)	
2014	West Bengal									1050 (523-1923)		13 (7-25)		1193 (594-2184)		15 (8-28)	
2015	West Bengal	195096 (168333-231914)	2736 (2360-3252)	2530 (2183-3007)	35 (31-42)	80214 (60096-91492)	1125 (843-1283)	1040 (779-1186)	15 (11-17)	1063 (529-1946)	16 (8-29)	14 (7-25)	0 (0-0)	1208 (601-2210)	18 (9-33)	16 (8-29)	0 (0-0)

Spn = *Streptococccus pneumoniae*; npnm = non-pneumonia, non-meningitis; HIV = HIV-infected cases only; UR = uncertainty range

Webappendix 12: Hib cases in India by state, year, and HIV status

		Pneumonia cases				Severe pneumonia cases				Meningitis cases				NPNM Cases			
Year	State	Hib pneumonia cases (UR)	Hib pneumonia cases, HIV (UR)	Hib pneumonia incidence rate (UR)	Hib pneumonia incidence rate, HIV (UR)	Hib severe pneumonia cases (UR)	Hib severe pneumonia cases; HIV (UR)	Hib severe pneumonia incidence rate (UR)	Hib severe pneumonia incidence rate; HIV (UR)	Hib meningitis cases (UR)	Hib meningitis cases; HIV (UR)	Hib meningitis incidence rate (UR)	Hib meningitis incidence rate; HIV (UR)	Hib NPNM cases (UR)	Hib NPNM cases; HIV (UR)	Hib NPNM incidence rate (UR)	Hib NPNM incidence rate; HIV (UR)
2000	Andhra Pradesh	208573 (190431-340787)		2724 (2487-4450)		41073 (24592-82901)		536 (321-1083)		3073 (1441-4556)		40 (19-59)		529 (248-784)		7 (3-10)	
2001	Andhra Pradesh									3066 (1344-4633)		40 (18-61)		1033 (453-1562)		14 (6-20)	
2002	Andhra Pradesh									3011 (1319-4549)		39 (17-60)		1015 (445-1534)		13 (6-20)	
2003	Andhra Pradesh									2930 (1284-4426)		39 (17-58)		988 (433-1492)		13 (6-20)	
2004	Andhra Pradesh									2824 (1237-4265)		38 (16-57)		952 (417-1438)		13 (6-19)	
2005	Andhra Pradesh									2680 (1171-4050)		36 (16-54)		903 (395-1365)		12 (5-18)	
2006	Andhra Pradesh									2529 (1103-3824)		34 (15-52)		852 (372-1289)		11 (5-17)	
2007	Andhra Pradesh									2345 (1020-3546)		32 (14-48)		791 (344-1196)		11 (5-16)	
2008	Andhra Pradesh									2152 (934-3250)		30 (13-45)		725 (315-1095)		10 (4-15)	
2009	Andhra Pradesh									1941 (826-2952)		27 (11-41)		654 (278-995)		9 (4-14)	
2010	Andhra Pradesh									1719 (717-2630)		24 (10-37)		579 (242-887)		8 (3-12)	
2011	Andhra Pradesh									1508 (616-2320)		22 (9-33)		508 (208-782)		7 (3-11)	
2012	Andhra Pradesh									1310 (525-2028)		19 (8-29)		442 (177-684)		6 (3-10)	
2013	Andhra Pradesh									1161 (465-1795)		17 (7-27)		391 (157-605)		6 (2-9)	
2014	Andhra Pradesh									1038 (415-1604)		16 (6-24)		350 (140-541)		5 (2-8)	
2015	Andhra Pradesh	36978 (33761-60418)	93 (85-153)	565 (516-923)	1 (1-2)	7282 (4360-14697)	18 (11-37)	111 (67-224)	0 (0-1)	401 (161-620)	1 (0-2)	6 (2-9)	0 (0-0)	135 (54-209)	0 (0-1)	2 (1-3)	0 (0-0)
2000	Arunachal Pradesh	4431 (4045-7239)		2833 (2587-4629)		872 (522-1759)		557 (334-1125)		24 (8-39)		16 (5-25)		8 (3-13)		5 (2-8)	
2001	Arunachal Pradesh									23 (8-37)		15 (5-23)		8 (3-12)		5 (2-8)	
2002	Arunachal Pradesh									23 (8-37)		15 (5-23)		8 (3-12)		5 (2-8)	
2003	Arunachal Pradesh									24 (8-39)		15 (5-24)		8 (3-13)		5 (2-8)	
2004	Arunachal Pradesh									25 (9-41)		16 (5-26)		9 (3-14)		5 (2-9)	
2005	Arunachal Pradesh									27 (10-43)		17 (6-27)		9 (3-15)		6 (2-9)	
2006	Arunachal Pradesh									32 (12-49)		20 (8-30)		11 (4-16)		7 (3-10)	
2007	Arunachal Pradesh									32 (14-48)		20 (9-30)		11 (5-16)		7 (3-10)	
2008	Arunachal Pradesh									33 (15-50)		21 (9-31)		11 (5-17)		7 (3-10)	
2009	Arunachal Pradesh									33 (13-50)		20 (8-31)		11 (4-17)		7 (3-10)	
2010	Arunachal Pradesh									30 (11-47)		19 (7-30)		10 (4-16)		6 (2-10)	
2011	Arunachal Pradesh									27 (10-44)		17 (6-27)		9 (3-15)		6 (2-9)	
2012	Arunachal Pradesh									25 (8-40)		16 (5-26)		8 (3-14)		5 (2-9)	
2013	Arunachal Pradesh									24 (8-38)		15 (5-25)		8 (3-13)		5 (2-8)	
2014	Arunachal Pradesh									23 (8-37)		15 (5-24)		8 (3-13)		5 (2-8)	
2015	Arunachal Pradesh	1535 (1401-2507)	4 (4-6)	992 (905-1620)	3 (2-4)	302 (181-610)	1 (0-2)	195 (117-394)	0 (0-1)	21 (7-33)	0 (0-0)	13 (4-21)	0 (0-0)	7 (2-11)	0 (0-0)	4 (1-7)	0 (0-0)
2000	Assam	89076 (81328-145541)		2593 (2368-4237)		17545 (10505-35413)		511 (306-1031)		1334 (512-2063)		39 (15-60)		230 (88-355)		7 (3-10)	
2001	Assam									1292 (496-1999)		37 (14-58)		223 (85-344)		6 (2-10)	
2002	Assam									1265 (486-1957)		36 (14-56)		218 (84-337)		6 (2-10)	
2003	Assam									1256 (483-1943)		36 (14-55)		216 (83-335)		6 (2-9)	
2004	Assam									1262 (485-1953)		36 (14-55)		217 (84-336)		6 (2-10)	
2005	Assam									1276 (497-1969)		36 (14-55)		220 (86-339)		6 (2-10)	
2006	Assam									1294 (510-1990)		36 (14-56)		223 (88-343)		6 (2-10)	
2007	Assam									1312 (525-2012)		37 (15-56)		226 (90-346)		6 (3-10)	
2008	Assam									1328 (531-2038)		37 (15-57)		229 (91-351)		6 (3-10)	
2009	Assam									1331 (532-2042)		37 (15-57)		229 (92-352)		6 (3-10)	
2010	Assam									1332 (533-2043)		37 (15-57)		229 (92-352)		6 (3-10)	
2011	Assam									1328 (531-2038)		37 (15-57)		229 (92-351)		6 (3-10)	
2012	Assam									1321 (528-2027)		37 (15-57)		227 (91-349)		6 (3-10)	
2013	Assam									1332 (533-2044)		38 (15-58)		229 (92-352)		6 (3-10)	
2014	Assam									1331 (532-2042)		38 (15-58)		229 (92-352)		7 (3-10)	
2015	Assam	21225 (19379-34679)	54 (49-88)	605 (552-988)	2 (1-2)	4181 (2504-8439)	11 (6-21)	119 (71-240)	0 (0-1)	471 (173-744)	1 (0-2)	13 (5-21)	0 (0-0)	159 (58-251)	0 (0-1)	5 (2-7)	0 (0-0)
2000	Bihar	488921 (446395-798849)		3960 (3615-6470)		96340 (57683-194450)		780 (467-1575)		7149 (3375-10594)		58 (27-86)		1231 (581-1824)		10 (5-15)	
2001	Bihar									7027 (3318-10413)		56 (26-83)		1210 (571-1793)		10 (5-14)	
2002	Bihar									6859 (3238-10164)		53 (25-79)		1181 (558-1750)		9 (4-14)	
2003	Bihar									6706 (3165-9932)		52 (24-76)		1155 (545-1710)		9 (4-13)	
2004	Bihar									6550 (3089-9694)		50 (23-73)		1128 (532-1669)		9 (4-13)	
2005	Bihar									6397 (2990-9491)		48 (22-71)		1102 (515-1634)		8 (4-12)	
2006	Bihar									6276 (2714-9525)		46 (20-70)		2116 (915-3211)		16 (7-24)	
2007	Bihar									6093 (2608-9273)		44 (19-68)		2054 (879-3126)		15 (6-23)	
2008	Bihar									5870 (2511-8928)		42 (18-64)		1979 (847-3009)		14 (6-22)	
2009	Bihar									5586 (2388-8491)		40 (17-61)		1883 (805-2862)		13 (6-20)	
2010	Bihar									5236 (2239-7960)		37 (16-56)		1765 (755-2683)		13 (5-19)	
2011	Bihar									4848 (2073-7370)		34 (15-52)		1634 (699-2484)		11 (5-17)	
2012	Bihar									4398 (1880-6685)		31 (13-47)		1482 (634-2254)		10 (4-16)	
2013	Bihar									3996 (1709-6075)		28 (12-42)		1347 (576-2048)		9 (4-14)	
2014	Bihar									3566 (1525-5421)		25 (11-38)		1202 (514-1827)		8 (4-13)	
2015	Bihar	59162 (54016-96665)	149 (136-244)	409 (374-668)	1 (1-2)	11653 (6977-23519)	29 (18-59)	81 (48-163)	0 (0-0)	532 (227-808)	1 (1-2)	4 (2-6)	0 (0-0)	179 (77-272)	0 (0-1)	1 (1-2)	0 (0-0)
2000	Chhattisgarh	108373 (98947-177070)		3894 (3555-6363)		21352 (12785-43097)		767 (459-1549)		986 (385-1520)		35 (14-55)		170 (66-262)		6 (2-9)	
2001	Chhattisgarh									976 (381-1504)		35 (14-54)		168 (66-259)		6 (2-9)	
2002	Chhattisgarh									951 (371-1466)		34 (13-52)		164 (64-253)		6 (2-9)	
2003	Chhattisgarh									929 (363-1435)		33 (13-51)		160 (63-247)		6 (2-9)	
2004	Chhattisgarh									904 (354-1397)		32 (12-49)		156 (61-241)		5 (2-8)	
2005	Chhattisgarh									896 (363-1372)		31 (13-48)		154 (62-236)		5 (2-8)	
2006	Chhattisgarh									919 (385-1394)		32 (13-49)		158 (66-240)		6 (2-8)	
2007	Chhattisgarh									905 (365-1392)		32 (13-49)		305 (123-469)		11 (4-16)	
2008	Chhattisgarh									899 (363-1385)		31 (13-48)		303 (122-467)		11 (4-16)	
2009	Chhattisgarh									866 (350-1334)		30 (12-47)		292 (118-450)		10 (4-16)	
2010	Chhattisgarh									812 (328-1252)		29 (12-44)		274 (111-422)		10 (4-15)	
2011	Chhattisgarh									769 (311-1186)		27 (11-42)		259 (105-400)		9 (4-14)	
2012	Chhattisgarh									723 (292-1115)		26 (10-40)		244 (99-376)		9 (4-13)	
2013	Chhattisgarh									705 (285-1087)		25 (10-39)		238 (96-366)		9 (3-13)	
2014	Chhattisgarh									704 (284-1085)		25 (10-39)		237 (96-366)		9 (3-13)	
2015	Chhattisgarh	16638 (15191-27185)	42 (38-69)	605 (552-988)	2 (1-2)	3278 (1963-6616)	8 (5-17)	119 (71-240)	0 (0-1)	226 (91-348)	1 (0-1)	8 (3-13)	0 (0-0)	76 (31-117)	0 (0-0)	3 (1-4)	0 (0-0)
2000	Delhi	41437 (37833-67703)		2642 (2412-4317)		8162 (4887-16475)		520 (312-1050)		373 (144-581)		24 (9-37)		126 (49-196)		8 (3-12)	
2001	Delhi									362 (140-563)		23 (9-36)		122 (47-190)		8 (3-12)	
2002	Delhi									351 (136-547)		22 (9-35)		118 (46-184)		7 (3-12)	
2003	Delhi									345 (134-538)		22 (8-34)		116 (45-181)		7 (3-11)	
2004	Delhi									345 (134-541)		22 (8-34)		116 (45-182)		7 (3-12)	
2005	Delhi									353 (148-541)		22 (9-34)		119 (50-182)		8 (3-12)	
2006	Delhi									366 (165-546)		23 (10-35)		123 (56-184)		8 (4-12)	
2007	Delhi									374 (184-539)		24 (12-34)		126 (62-182)		8 (4-12)	
2008	Delhi									373 (184-540)		24 (12-34)		126 (62-182)		8 (4-12)	
2009	Delhi									366 (182-534)		23 (12-34)		123 (61-180)		8 (4-12)	
2010	Delhi									355 (179-524)		23 (12-34)		120 (60-177)		8 (4-11)	
2011	Delhi									346 (176-516)		22 (11-34)		117 (59-174)		8 (4-11)	
2012	Delhi									340 (92-521)		22 (6-34)		115 (31-176)		8 (2-12)	
2013	Delhi									169 (47-263)		11 (3-17)		57 (16-89)		4 (1-6)	
2014	Delhi									31 (9-49)		2 (1-3)		10 (3-16)		1 (0-1)	
2015	Delhi	2107 (1924-3443)	5 (5-9)	143 (130-233)	0 (0-1)	415 (248-837)	1 (1-2)	28 (17-57)	0 (0-0)	27 (8-44)	0 (0-0)	2 (1-3)	0 (0-0)	9 (3-15)	0 (0-0)	1 (0-1)	0 (0-0)
2000	Goa	2543 (2321-4154)		2145 (1959-3505)		500 (299-1010)		422 (253-852)		17 (4-28)		14 (4-23)		6 (1-9)		5 (1-8)	
2001	Goa									14 (4-23)		12 (3-19)		5 (1-8)		4 (1-7)	
2002	Goa									10 (3-16)		8 (2-14)		3 (1-5)		3 (1-5)	
2003	Goa									12 (3-20)		10 (3-17)		4 (1-7)		3 (1-6)	
2004	Goa									12 (3-20)		10 (3-17)		4 (1-7)		4 (1-6)	
2005	Goa									12 (3-20)		10 (3-17)		4 (1-7)		4 (1-6)	
2006	Goa									10 (2-17)		9 (2-14)		3 (1-6)		3 (1-5)	
2007	Goa									7 (2-11)		6 (1-10)		2 (1-4)		2 (0-3)	
2008	Goa									6 (1-10)		5 (1-8)		2 (0-3)		2 (0-3)	
2009	Goa									7 (2-11)		6 (1-10)		2 (1-4)		2 (0-3)	
2010	Goa									7 (2-11)		6 (1-10)		2 (1-4)		2 (0-3)	
2011	Goa									5 (1-9)		5 (1-8)		2 (0-3)		2 (0-3)	
2012	Goa									4 (1-7)		4 (1-6)		1 (0-2)		1 (0-2)	
2013	Goa									0 (0-1)		0 (0-1)		0 (0-0)		0 (0-0)	
2014	Goa									0 (0-0)		0 (0-0)		0 (0-0)		0 (0-0)	
2015	Goa	84 (77-138)	0 (0-0)	79 (72-129)	0 (0-0)	17 (10-34)	0 (0-0)	16 (9-31)	0 (0-0)	0 (0-0)	0 (0-0)	0 (0-0)	0 (0-0)	0 (0-0)	0 (0-0)	0 (0-0)	0 (0-0)
2000	Gujarat	165288 (150911-270064)		2765 (2525-4518)		32564 (19498-65727)		545 (326-1100)		1992 (788-3062)		33 (13-51)		343 (136-527)		6 (2-9)	
2001	Gujarat									1915 (757-2943)		32 (13-49)		330 (130-507)		5 (2-8)	
2002	Gujarat									1853 (733-2849)		31 (12-47)		319 (126-491)		5 (2-8)	
2003	Gujarat									1801 (653-2851)		30 (11-47)		607 (220-961)		10 (4-16)	
2004	Gujarat									1744 (634-2765)		29 (10-45)		588 (214-932)		10 (3-15)	
2005	Gujarat									1758 (673-2748)		29 (11-45)		593 (227-926)		10 (4-15)	
2006	Gujarat									1802 (729-2772)		29 (12-45)		607 (246-934)		10 (4-15)	
2007	Gujarat									1784 (766-2690)		29 (12-44)		601 (258-907)		10 (4-15)	
2008	Gujarat									1742 (750-2635)		28 (12-43)		587 (253-888)		10 (4-14)	
2009	Gujarat									1655 (714-2508)		27 (12-41)		558 (241-845)		9 (4-14)	
2010	Gujarat									1533 (662-2325)		25 (11-38)		517 (223-784)		8 (4-13)	
2011	Gujarat									1417 (611-2148)		23 (10-35)		478 (206-724)		8 (3-12)	
2012	Gujarat									1311 (566-1988)		22 (9-33)		442 (191-670)		7 (3-11)	
2013	Gujarat									458 (198-694)		8 (3-12)		154 (67-234)		3 (1-4)	
2014	Gujarat									149 (64-226)		3 (1-4)		50 (22-76)		1 (0-1)	
2015	Gujarat	17958 (16396-29342)	45 (41-74)	304 (277-496)	1 (1-1)	3535 (2117-7136)	9 (5-18)	60 (36-121)	0 (0-0)	141 (61-214)	0 (0-1)	2 (1-4)	0 (0-0)	48 (21-72)	0 (0-0)	1 (0-1)	0 (0-0)
2000	Haryana	64964 (59314-106145)		2519 (2300-4116)		12791 (7659-25817)		496 (297-1001)		1468 (667-2193)		57 (26-85)		253 (115-378)		10 (4-15)	

Hib = *Haemophilus influenzae* type b; npnm = non-pneumonia, non-meningitis; HIV = HIV-infected cases only; UR = uncertainty range

Year	State	Hib pneumonia cases (UR)	Hib pneumonia cases, HIV (UR)	Hib pneumonia incidence rate (UR)	Hib pneumonia incidence rate, HIV (UR)	Hib severe pneumonia cases (UR)	Hib severe pneumonia cases; HIV (UR)	Hib severe pneumonia incidence rate (UR)	Hib severe pneumonia incidence rate; HIV (UR)	Hib meningitis cases (UR)	Hib meningitis cases; HIV (UR)	Hib meningitis incidence rate (UR)	Hib meningitis incidence rate; HIV (UR)	Hib NPNM cases (UR)	Hib NPNM cases; HIV (UR)	Hib NPNM incidence rate (UR)	Hib NPNM incidence rate; HIV (UR)
2001	Haryana									1411 (641-2108)		54 (25-81)		243 (110-363)		9 (4-14)	
2002	Haryana									1359 (618-2030)		52 (24-77)		234 (106-350)		9 (4-13)	
2003	Haryana									1318 (599-1971)		50 (23-75)		227 (103-339)		9 (4-13)	
2004	Haryana									1295 (550-1984)		49 (21-75)		437 (185-669)		17 (7-25)	
2005	Haryana									1259 (558-1900)		48 (21-72)		424 (188-640)		16 (7-24)	
2006	Haryana									1208 (560-1793)		46 (21-68)		407 (189-604)		15 (7-23)	
2007	Haryana									1211 (589-1761)		46 (22-66)		408 (198-594)		15 (7-22)	
2008	Haryana									1148 (560-1674)		43 (21-63)		387 (189-564)		15 (7-21)	
2009	Haryana									1027 (497-1507)		39 (19-57)		346 (168-508)		13 (6-19)	
2010	Haryana									983 (471-1448)		37 (18-55)		331 (159-488)		13 (6-18)	
2011	Haryana									907 (430-1340)		34 (16-51)		306 (145-452)		12 (6-17)	
2012	Haryana									781 (367-1158)		30 (14-44)		263 (124-390)		10 (5-15)	
2013	Haryana									199 (94-295)		8 (4-11)		67 (32-100)		3 (1-4)	
2014	Haryana									81 (38-120)		3 (1-5)		27 (13-40)		1 (0-2)	
2015	Haryana	4417 (4032-7216)	11 (10-18)	172 (157-282)	0 (0-1)	870 (521-1756)	2 (1-4)	34 (20-69)	0 (0-0)	78 (37-116)	0 (0-0)	3 (1-5)	0 (0-0)	26 (12-39)	0 (0-0)	1 (0-2)	0 (0-0)
2000	Himachal Pradesh	15717 (14350-25680)		2457 (2244-4015)		3094 (1853-6245)		484 (290-976)		287 (122-437)		45 (19-68)		97 (41-147)		15 (6-23)	
2001	Himachal Pradesh									264 (112-402)		41 (17-63)		89 (38-136)		14 (6-21)	
2002	Himachal Pradesh									243 (103-370)		38 (16-58)		82 (35-125)		13 (5-19)	
2003	Himachal Pradesh									225 (95-342)		35 (15-54)		76 (32-115)		12 (5-18)	
2004	Himachal Pradesh									209 (89-320)		33 (14-50)		71 (30-108)		11 (5-17)	
2005	Himachal Pradesh									197 (86-298)		31 (14-47)		66 (29-100)		10 (5-16)	
2006	Himachal Pradesh									186 (83-280)		29 (13-44)		63 (28-94)		10 (4-15)	
2007	Himachal Pradesh									176 (81-263)		28 (13-42)		59 (27-89)		9 (4-14)	
2008	Himachal Pradesh									169 (78-252)		27 (12-40)		57 (26-85)		9 (4-14)	
2009	Himachal Pradesh									163 (77-240)		26 (12-39)		55 (26-81)		9 (4-13)	
2010	Himachal Pradesh									158 (78-230)		26 (13-37)		53 (26-78)		9 (4-13)	
2011	Himachal Pradesh									153 (78-220)		25 (13-36)		52 (26-74)		9 (4-12)	
2012	Himachal Pradesh									149 (79-215)		25 (13-36)		50 (27-73)		8 (4-12)	
2013	Himachal Pradesh									147 (78-213)		25 (13-36)		49 (26-72)		8 (4-12)	
2014	Himachal Pradesh									146 (78-213)		25 (13-37)		49 (26-72)		8 (5-12)	
2015	Himachal Pradesh	7604 (6942-12424)	19 (18-31)	1321 (1206-2159)	3 (3-5)	1499 (898-3026)	4 (2-8)	260 (156-526)	1 (0-1)	136 (73-199)	0 (0-1)	24 (13-35)	0 (0-0)	46 (25-67)	0 (0-0)	8 (4-12)	0 (0-0)
2000	Jammu & Kashmir	31652 (28899-51716)		2998 (2737-4898)		6236 (3734-12587)		591 (354-1192)		543 (300-801)		51 (28-76)		183 (101-270)		17 (10-26)	
2001	Jammu & Kashmir									506 (280-747)		46 (25-68)		171 (94-252)		15 (9-23)	
2002	Jammu & Kashmir									467 (258-689)		41 (22-60)		157 (87-232)		14 (8-20)	
2003	Jammu & Kashmir									426 (235-626)		36 (20-52)		144 (79-211)		12 (7-18)	
2004	Jammu & Kashmir									380 (208-555)		31 (17-45)		128 (70-187)		10 (6-15)	
2005	Jammu & Kashmir									332 (171-479)		26 (13-37)		112 (58-161)		9 (4-13)	
2006	Jammu & Kashmir									290 (142-427)		22 (11-32)		98 (48-144)		7 (4-11)	
2007	Jammu & Kashmir									255 (118-382)		18 (9-28)		86 (40-129)		6 (3-9)	
2008	Jammu & Kashmir									229 (106-342)		16 (7-24)		77 (36-115)		5 (2-8)	
2009	Jammu & Kashmir									209 (96-311)		14 (6-21)		70 (32-105)		5 (2-7)	
2010	Jammu & Kashmir									191 (88-285)		13 (6-19)		64 (30-96)		4 (2-6)	
2011	Jammu & Kashmir									174 (80-260)		11 (5-16)		59 (27-88)		4 (2-6)	
2012	Jammu & Kashmir									157 (72-235)		10 (4-14)		53 (24-79)		3 (2-5)	
2013	Jammu & Kashmir									51 (24-76)		3 (1-5)		17 (8-26)		1 (0-2)	
2014	Jammu & Kashmir									16 (7-23)		1 (0-1)		5 (2-8)		0 (0-0)	
2015	Jammu & Kashmir	2174 (1985-3552)	5 (5-9)	123 (113-202)	0 (0-1)	429 (257-865)	1 (1-2)	24 (15-49)	0 (0-0)	15 (7-23)	0 (0-0)	1 (0-1)	0 (0-0)	5 (2-8)	0 (0-0)	0 (0-0)	0 (0-0)
2000	Jharkhand	145810 (133127-238239)		3960 (3615-6470)		28731 (17203-57990)		780 (467-1575)		1044 (392-1619)		28 (11-44)		180 (67-279)		5 (2-8)	
2001	Jharkhand									1044 (357-1669)		28 (10-45)		352 (120-563)		9 (3-15)	
2002	Jharkhand									1027 (351-1641)		27 (9-43)		346 (118-553)		9 (3-15)	
2003	Jharkhand									1022 (349-1632)		27 (9-43)		344 (118-550)		9 (3-14)	
2004	Jharkhand									973 (333-1554)		25 (9-40)		328 (112-524)		8 (3-14)	
2005	Jharkhand									891 (307-1421)		23 (8-36)		300 (104-479)		8 (3-12)	
2006	Jharkhand									890 (310-1416)		23 (8-36)		300 (104-477)		8 (3-12)	
2007	Jharkhand									830 (291-1317)		21 (7-33)		280 (98-444)		7 (2-11)	
2008	Jharkhand									744 (261-1181)		19 (7-29)		251 (88-398)		6 (2-10)	
2009	Jharkhand									659 (231-1047)		16 (6-26)		222 (78-353)		6 (2-9)	
2010	Jharkhand									659 (231-1046)		16 (6-26)		222 (78-353)		5 (2-9)	
2011	Jharkhand									617 (217-980)		15 (5-24)		208 (73-330)		5 (2-8)	
2012	Jharkhand									560 (197-890)		14 (5-22)		189 (66-300)		5 (2-7)	
2013	Jharkhand									498 (175-791)		12 (4-19)		168 (59-267)		4 (1-7)	
2014	Jharkhand									435 (153-691)		11 (4-17)		147 (51-233)		4 (1-6)	
2015	Jharkhand	22649 (20679-37007)	57 (52-93)	557 (508-909)	1 (1-2)	4460 (2671-9003)	11 (7-23)	110 (66-221)	0 (0-1)	94 (33-149)	0 (0-0)	2 (1-4)	0 (0-0)	32 (11-50)	0 (0-0)	1 (0-1)	0 (0-0)
2000	Karnataka	161146 (147130-263297)		2870 (2620-4689)		31747 (19008-64077)		565 (338-1141)		1930 (771-2983)		34 (14-53)		651 (260-1006)		12 (5-18)	
2001	Karnataka									1866 (746-2884)		33 (13-51)		629 (251-972)		11 (4-17)	
2002	Karnataka									1840 (736-2845)		32 (13-50)		620 (248-959)		11 (4-17)	
2003	Karnataka									1827 (731-2826)		32 (13-50)		616 (246-953)		11 (4-17)	
2004	Karnataka									1822 (730-2822)		32 (13-50)		614 (246-951)		11 (4-17)	
2005	Karnataka									1816 (749-2789)		32 (13-49)		612 (253-940)		11 (4-16)	
2006	Karnataka									1814 (771-2759)		32 (13-48)		612 (260-930)		11 (5-16)	
2007	Karnataka									1784 (783-2686)		31 (14-47)		602 (264-906)		11 (5-16)	
2008	Karnataka									1736 (764-2621)		30 (13-46)		585 (258-884)		10 (5-15)	
2009	Karnataka									1669 (752-2503)		29 (13-44)		563 (254-844)		10 (4-15)	
2010	Karnataka									1568 (724-2334)		28 (13-41)		529 (244-787)		9 (4-14)	
2011	Karnataka									1439 (681-2123)		26 (12-38)		485 (230-716)		9 (4-13)	
2012	Karnataka									1262 (613-1844)		23 (11-33)		425 (206-622)		8 (4-11)	
2013	Karnataka									354 (172-519)		6 (3-9)		119 (58-175)		2 (1-3)	
2014	Karnataka									76 (37-111)		1 (1-2)		26 (12-37)		0 (0-1)	
2015	Karnataka	9605 (8770-15694)	24 (22-40)	177 (161-289)	0 (0-1)	1892 (1133-3818)	5 (3-10)	35 (21-70)	0 (0-0)	59 (29-86)	0 (0-0)	1 (1-2)	0 (0-0)	20 (10-29)	0 (0-0)	0 (0-1)	0 (0-0)
2000	Kerala	66393 (60618-108480)		2084 (1902-3405)		13068 (7825-26376)		410 (246-828)		256 (53-424)		8 (2-13)		86 (18-143)		3 (1-4)	
2001	Kerala									249 (51-411)		8 (2-13)		84 (17-139)		3 (1-4)	
2002	Kerala									244 (51-404)		8 (2-13)		82 (17-136)		3 (1-4)	
2003	Kerala									244 (51-405)		8 (2-13)		82 (17-137)		3 (1-4)	
2004	Kerala									251 (52-419)		8 (2-14)		84 (18-141)		3 (1-5)	
2005	Kerala									266 (61-437)		9 (2-14)		90 (21-147)		3 (1-5)	
2006	Kerala									289 (74-466)		10 (2-16)		98 (25-157)		3 (1-5)	
2007	Kerala									315 (90-490)		11 (3-17)		106 (30-165)		4 (1-6)	
2008	Kerala									337 (98-532)		12 (3-18)		114 (33-179)		4 (1-6)	
2009	Kerala									352 (102-560)		12 (4-20)		119 (34-189)		4 (1-7)	
2010	Kerala									359 (104-573)		13 (4-21)		121 (35-193)		4 (1-7)	
2011	Kerala									363 (105-580)		13 (4-21)		122 (35-195)		4 (1-7)	
2012	Kerala									57 (16-91)		2 (1-3)		19 (6-31)		1 (0-1)	
2013	Kerala									28 (8-45)		1 (0-2)		10 (3-15)		0 (0-1)	
2014	Kerala									29 (8-46)		1 (0-2)		10 (3-15)		0 (0-1)	
2015	Kerala	1342 (1226-2193)	3 (3-6)	54 (49-88)	0 (0-0)	264 (158-534)	1 (0-1)	11 (6-21)	0 (0-0)	29 (8-47)	0 (0-0)	1 (0-2)	0 (0-0)	10 (3-16)	0 (0-0)	0 (0-1)	0 (0-0)
2000	Madhya Pradesh	326254 (297877-533067)		3894 (3555-6363)		64281 (38488-129742)		767 (459-1549)		4529 (1701-7035)		54 (20-84)		780 (293-1211)		9 (3-14)	
2001	Madhya Pradesh									4331 (1626-6727)		51 (19-80)		746 (280-1158)		9 (3-14)	
2002	Madhya Pradesh									4158 (1562-6459)		49 (18-76)		716 (269-1112)		8 (3-13)	
2003	Madhya Pradesh									4010 (1507-6233)		47 (18-73)		691 (259-1073)		8 (3-13)	
2004	Madhya Pradesh									3889 (1463-6051)		46 (17-71)		670 (252-1042)		8 (3-12)	
2005	Madhya Pradesh									3800 (1469-5874)		45 (17-69)		654 (253-1012)		8 (3-12)	
2006	Madhya Pradesh									3726 (1481-5721)		44 (17-67)		642 (255-985)		8 (3-12)	
2007	Madhya Pradesh									3616 (1479-5510)		43 (17-65)		623 (255-949)		7 (3-11)	
2008	Madhya Pradesh									3479 (1425-5309)		41 (17-63)		599 (245-914)		7 (3-11)	
2009	Madhya Pradesh									3312 (1357-5057)		39 (16-60)		570 (234-871)		7 (3-10)	
2010	Madhya Pradesh									3134 (1284-4785)		37 (15-57)		540 (221-824)		6 (3-10)	
2011	Madhya Pradesh									2973 (1218-4538)		36 (15-55)		512 (210-781)		6 (3-9)	
2012	Madhya Pradesh									2826 (1157-4312)		34 (14-52)		487 (199-743)		6 (2-9)	
2013	Madhya Pradesh									2748 (1035-4310)		34 (13-53)		927 (349-1453)		11 (4-18)	
2014	Madhya Pradesh									2494 (939-3909)		31 (12-48)		841 (316-1318)		10 (4-16)	
2015	Madhya Pradesh	24288 (22175-39684)	61 (56-100)	303 (276-495)	1 (1-1)	4783 (2864-9654)	12 (7-24)	60 (36-120)	0 (0-0)	283 (106-443)	1 (0-1)	4 (1-6)	0 (0-0)	95 (36-149)	0 (0-0)	1 (0-2)	0 (0-0)
2000	Maharashtra	310589 (283575-507473)		2856 (2608-4667)		61165 (36622-123453)		563 (337-1135)		3031 (1284-4618)		28 (12-42)		1022 (433-1557)		9 (4-14)	
2001	Maharashtra									2834 (1200-4318)		26 (11-40)		955 (405-1455)		9 (4-13)	
2002	Maharashtra									2638 (1118-4021)		24 (10-37)		889 (377-1355)		8 (3-12)	
2003	Maharashtra									2456 (1041-3746)		23 (10-34)		828 (351-1263)		8 (3-12)	
2004	Maharashtra									2295 (975-3507)		21 (9-32)		774 (329-1182)		7 (3-11)	
2005	Maharashtra									2158 (942-3266)		20 (9-30)		727 (317-1101)		7 (3-10)	
2006	Maharashtra									2030 (912-3043)		19 (8-28)		684 (307-1026)		6 (3-9)	
2007	Maharashtra									1884 (871-2792)		17 (8-26)		635 (294-941)		6 (3-9)	
2008	Maharashtra									1739 (805-2583)		16 (8-24)		586 (272-871)		5 (3-8)	
2009	Maharashtra									1607 (747-2395)		15 (7-23)		542 (252-807)		5 (2-8)	
2010	Maharashtra									1486 (696-2229)		14 (7-21)		501 (234-751)		5 (2-7)	
2011	Maharashtra									1425 (673-2156)		14 (6-21)		480 (227-727)		5 (2-7)	
2012	Maharashtra									1375 (321-2176)		13 (3-21)		464 (108-734)		4 (1-7)	
2013	Maharashtra									1308 (309-2096)		13 (3-21)		441 (104-707)		4 (1-7)	
2014	Maharashtra									1210 (289-1959)		12 (3-20)		408 (97-660)		4 (1-7)	
2015	Maharashtra	190972 (174362-312031)	482 (441-788)	1924 (1757-3144)	5 (4-8)	37611 (22520-75914)	95 (57-192)	379 (227-765)	1 (1-2)	1049 (252-1710)	3 (1-5)	11 (3-17)	0 (0-0)	354 (85-576)	1 (0-2)	4 (1-6)	0 (0-0)
2000	Manipur	5926 (5411-9683)		2593 (2367-4237)		1167 (698-2355)		510 (306-1030)		23 (8-37)		10 (3-16)		8 (3-13)		3 (1-6)	
2001	Manipur									22 (7-36)		10 (3-15)		8 (3-12)		3 (1-5)	
2002	Manipur									22 (7-35)		9 (3-14)		7 (2-12)		3 (1-5)	
2003	Manipur									21 (3-36)		9 (1-15)		7 (1-12)		3 (0-5)	

Hib = *Haemophilus influenzae* type b; npnm = non-pneumonia, non-meningitis; HIV = HIV-infected cases only; UR = uncertainty range

Year	State	Hib pneumonia cases (UR)	Hib pneumonia cases, HIV (UR)	Hib pneumonia incidence rate (UR)	Hib pneumonia incidence rate, HIV (UR)	Hib severe pneumonia cases (UR)	Hib severe pneumonia cases; HIV (UR)	Hib severe pneumonia incidence rate (UR)	Hib severe pneumonia incidence rate; HIV (UR)	Hib meningitis cases (UR)	Hib meningitis cases; HIV (UR)	Hib meningitis incidence rate (UR)	Hib meningitis incidence rate; HIV (UR)	Hib NPNM cases (UR)	Hib NPNM cases; HIV (UR)	Hib NPNM incidence rate (UR)	Hib NPNM incidence rate; HIV (UR)
2004	Manipur									21 (3-35)		8 (1-14)		7 (1-12)		3 (0-5)	
2005	Manipur									20 (2-35)		8 (1-14)		7 (1-12)		3 (0-5)	
2006	Manipur									20 (2-34)		8 (1-13)		7 (1-12)		3 (0-4)	
2007	Manipur									20 (2-34)		7 (1-13)		7 (1-12)		2 (0-4)	
2008	Manipur									20 (2-35)		7 (1-13)		7 (1-12)		2 (0-4)	
2009	Manipur									21 (3-36)		8 (1-13)		7 (1-12)		3 (0-4)	
2010	Manipur									22 (3-38)		8 (1-14)		7 (1-13)		3 (0-5)	
2011	Manipur									23 (3-40)		8 (1-14)		8 (1-14)		3 (0-5)	
2012	Manipur									24 (3-41)		8 (1-14)		8 (1-14)		3 (0-5)	
2013	Manipur									25 (4-42)		8 (1-14)		8 (1-14)		3 (0-5)	
2014	Manipur									25 (4-42)		8 (1-14)		8 (1-14)		3 (0-5)	
2015	Manipur	1909 (1743-3119)	5 (4-8)	637 (582-1041)	2 (1-3)	376 (225-759)	1 (1-2)	125 (75-253)	0 (0-1)	22 (3-37)	0 (0-0)	7 (1-12)	0 (0-0)	7 (1-13)	0 (0-0)	2 (0-4)	0 (0-0)
2000	Meghalaya	9352 (8539-15281)		2588 (2363-4229)		1841 (1103-3717)		510 (305-1029)		157 (71-235)		43 (20-65)		27 (12-40)		7 (3-11)	
2001	Meghalaya									163 (74-243)		44 (20-65)		28 (13-42)		8 (3-11)	
2002	Meghalaya									169 (77-253)		44 (20-67)		29 (13-44)		8 (3-11)	
2003	Meghalaya									177 (80-265)		45 (21-68)		31 (14-46)		8 (4-12)	
2004	Meghalaya									186 (84-279)		47 (21-70)		32 (15-48)		8 (4-12)	
2005	Meghalaya									196 (91-292)		48 (22-72)		34 (16-50)		8 (4-12)	
2006	Meghalaya									205 (97-304)		49 (23-73)		35 (17-52)		9 (4-13)	
2007	Meghalaya									211 (101-310)		50 (24-73)		36 (17-53)		9 (4-13)	
2008	Meghalaya									213 (102-312)		49 (24-72)		37 (18-54)		8 (4-12)	
2009	Meghalaya									206 (94-307)		47 (21-70)		35 (16-53)		8 (4-12)	
2010	Meghalaya									203 (88-307)		46 (20-69)		35 (15-53)		8 (3-12)	
2011	Meghalaya									199 (82-303)		44 (18-67)		34 (14-52)		8 (3-12)	
2012	Meghalaya									195 (77-300)		43 (17-66)		34 (13-52)		7 (3-11)	
2013	Meghalaya									196 (78-302)		42 (17-65)		34 (13-52)		7 (3-11)	
2014	Meghalaya									199 (79-305)		42 (17-65)		34 (14-53)		7 (3-11)	
2015	Meghalaya	6581 (6009-10753)	17 (15-27)	1387 (1267-2267)	4 (3-6)	1296 (776-2617)	3 (2-7)	273 (164-552)	1 (0-1)	185 (73-284)	0 (0-1)	39 (15-60)	0 (0-0)	32 (13-49)	0 (0-0)	7 (3-10)	0 (0-0)
2000	Mizoram	2566 (2343-4192)		2277 (2079-3721)		505 (302-1019)		448 (268-905)		33 (12-52)		30 (11-46)		6 (2-9)		5 (2-8)	
2001	Mizoram									32 (12-50)		28 (10-44)		6 (2-9)		5 (2-8)	
2002	Mizoram									32 (12-50)		27 (10-42)		5 (2-9)		5 (2-7)	
2003	Mizoram									32 (12-49)		26 (10-41)		5 (2-8)		5 (2-7)	
2004	Mizoram									32 (12-49)		26 (9-40)		5 (2-8)		4 (2-7)	
2005	Mizoram									32 (12-49)		25 (9-40)		5 (2-8)		4 (2-7)	
2006	Mizoram									32 (12-50)		25 (9-39)		6 (2-9)		4 (2-7)	
2007	Mizoram									32 (12-50)		25 (9-39)		6 (2-9)		4 (2-7)	
2008	Mizoram									33 (12-51)		25 (9-39)		6 (2-9)		4 (2-7)	
2009	Mizoram									33 (13-52)		25 (10-39)		6 (2-9)		4 (2-7)	
2010	Mizoram									34 (13-52)		25 (10-39)		6 (2-9)		4 (2-7)	
2011	Mizoram									35 (14-53)		26 (10-39)		6 (2-9)		4 (2-7)	
2012	Mizoram									35 (14-54)		26 (10-39)		6 (2-9)		4 (2-7)	
2013	Mizoram									36 (14-55)		26 (11-40)		6 (2-9)		5 (2-7)	
2014	Mizoram									36 (14-55)		26 (11-40)		6 (2-9)		4 (2-7)	
2015	Mizoram	712 (650-1163)	2 (2-3)	511 (467-835)	1 (1-2)	140 (84-283)	0 (0-1)	101 (60-203)	0 (0-1)	32 (12-51)	0 (0-0)	23 (9-36)	0 (0-0)	11 (4-17)	0 (0-0)	8 (3-12)	0 (0-0)
2000	Nagaland	5233 (4778-8550)		2461 (2247-4022)		1030 (617-2079)		485 (290-978)		23 (8-37)		11 (4-18)		8 (3-13)		4 (1-6)	
2001	Nagaland									25 (8-39)		11 (4-18)		8 (3-13)		4 (1-6)	
2002	Nagaland									26 (9-42)		12 (4-19)		9 (3-14)		4 (1-7)	
2003	Nagaland									28 (9-45)		13 (4-21)		9 (3-15)		4 (1-7)	
2004	Nagaland									29 (10-47)		13 (4-21)		10 (3-16)		5 (1-7)	
2005	Nagaland									30 (10-48)		14 (4-22)		10 (3-16)		5 (1-7)	
2006	Nagaland									30 (9-48)		14 (4-22)		10 (3-16)		5 (1-7)	
2007	Nagaland									29 (9-48)		13 (4-22)		10 (3-16)		4 (1-7)	
2008	Nagaland									29 (9-47)		13 (4-21)		10 (3-16)		4 (1-7)	
2009	Nagaland									28 (8-45)		13 (4-21)		9 (3-15)		4 (1-7)	
2010	Nagaland									28 (8-46)		13 (4-21)		9 (3-16)		4 (1-7)	
2011	Nagaland									28 (8-45)		13 (4-21)		9 (3-15)		4 (1-7)	
2012	Nagaland									27 (7-44)		12 (3-20)		9 (2-15)		4 (1-7)	
2013	Nagaland									26 (7-43)		12 (3-20)		9 (2-14)		4 (1-7)	
2014	Nagaland									25 (7-41)		11 (3-19)		8 (2-14)		4 (1-6)	
2015	Nagaland	2606 (2379-4258)	7 (6-11)	1213 (1107-1982)	3 (3-5)	513 (307-1036)	1 (1-3)	239 (143-482)	1 (0-1)	22 (6-37)	0 (0-0)	10 (3-17)	0 (0-0)	8 (2-12)	0 (0-0)	4 (1-6)	0 (0-0)
2000	Odisha	125261 (114366-204664)		3085 (2817-5041)		24661 (14766-49776)		607 (364-1226)		2427 (999-3703)		60 (25-91)		418 (172-638)		10 (4-16)	
2001	Odisha									2291 (943-3497)		56 (23-85)		395 (162-602)		10 (4-15)	
2002	Odisha									2152 (886-3283)		52 (22-80)		371 (152-565)		9 (4-14)	
2003	Odisha									2025 (833-3089)		49 (20-75)		349 (143-532)		8 (3-13)	
2004	Odisha									1895 (779-2890)		46 (19-70)		326 (134-498)		8 (3-12)	
2005	Odisha									1765 (722-2697)		43 (17-65)		304 (124-464)		7 (3-11)	
2006	Odisha									1644 (668-2517)		40 (16-61)		283 (115-433)		7 (3-10)	
2007	Odisha									1513 (610-2319)		37 (15-56)		261 (105-399)		6 (3-10)	
2008	Odisha									1403 (566-2150)		34 (14-52)		242 (97-370)		6 (2-9)	
2009	Odisha									1303 (525-1996)		32 (13-49)		224 (90-344)		5 (2-8)	
2010	Odisha									1213 (489-1857)		30 (12-45)		209 (84-320)		5 (2-8)	
2011	Odisha									1139 (421-1792)		28 (10-44)		384 (142-604)		9 (3-15)	
2012	Odisha									1056 (390-1661)		26 (10-41)		356 (132-560)		9 (3-14)	
2013	Odisha									983 (363-1545)		25 (9-39)		331 (122-521)		8 (3-13)	
2014	Odisha									893 (330-1405)		23 (8-35)		301 (111-473)		8 (3-12)	
2015	Odisha	64286 (58695-105037)	162 (148-265)	1634 (1492-2670)	4 (4-7)	12663 (7582-25558)	32 (19-65)	322 (193-650)	1 (0-2)	717 (265-1127)	2 (1-3)	18 (7-29)	0 (0-0)	242 (89-380)	1 (0-1)	6 (2-10)	0 (0-0)
2000	Punjab	69076 (63068-112863)		2851 (2603-4658)		13602 (8144-27454)		561 (336-1133)		1243 (602-1826)		51 (25-75)		419 (203-615)		17 (8-25)	
2001	Punjab									1155 (559-1697)		47 (23-70)		389 (188-572)		16 (8-23)	
2002	Punjab									1074 (520-1578)		44 (21-65)		362 (175-532)		15 (7-22)	
2003	Punjab									1004 (487-1477)		41 (20-60)		339 (164-498)		14 (7-20)	
2004	Punjab									946 (459-1394)		39 (19-57)		319 (155-470)		13 (6-19)	
2005	Punjab									897 (447-1307)		37 (18-54)		302 (151-441)		12 (6-18)	
2006	Punjab									854 (437-1229)		35 (18-50)		288 (147-414)		12 (6-17)	
2007	Punjab									804 (424-1164)		33 (17-48)		271 (143-392)		11 (6-16)	
2008	Punjab									751 (396-1089)		31 (16-45)		253 (134-367)		10 (6-15)	
2009	Punjab									692 (365-1005)		29 (15-42)		233 (123-339)		10 (5-14)	
2010	Punjab									632 (333-918)		26 (14-38)		213 (112-309)		9 (5-13)	
2011	Punjab									592 (312-863)		25 (13-36)		200 (105-291)		8 (4-12)	
2012	Punjab									568 (302-836)		24 (13-36)		192 (102-282)		8 (4-12)	
2013	Punjab									563 (303-839)		24 (13-36)		190 (102-283)		8 (4-12)	
2014	Punjab									581 (177-850)		25 (8-37)		196 (60-286)		9 (3-12)	
2015	Punjab	4815 (4396-7867)	12 (11-20)	211 (193-345)	1 (0-1)	949 (568-1916)	2 (1-5)	42 (25-84)	0 (0-0)	101 (31-150)	0 (0-0)	4 (1-7)	0 (0-0)	34 (11-51)	0 (0-0)	1 (0-2)	0 (0-0)
2000	Rajasthan	319714 (291905-522381)		3886 (3548-6349)		62997 (37719-127151)		766 (458-1545)		4438 (1859-6749)		54 (23-82)		764 (320-1162)		9 (4-14)	
2001	Rajasthan									4259 (1784-6475)		51 (22-78)		733 (307-1115)		9 (4-13)	
2002	Rajasthan									4081 (1710-6206)		49 (21-75)		703 (294-1069)		8 (4-13)	
2003	Rajasthan									3899 (1635-5932)		47 (20-71)		671 (281-1022)		8 (3-12)	
2004	Rajasthan									3729 (1564-5678)		45 (19-68)		642 (269-978)		8 (3-12)	
2005	Rajasthan									3562 (1521-5399)		43 (18-65)		613 (262-930)		7 (3-11)	
2006	Rajasthan									3418 (1485-5153)		41 (18-62)		589 (256-887)		7 (3-11)	
2007	Rajasthan									3261 (1442-4889)		39 (17-59)		561 (248-842)		7 (3-10)	
2008	Rajasthan									3112 (1377-4669)		38 (17-56)		536 (237-804)		6 (3-10)	
2009	Rajasthan									2966 (1219-4558)		36 (15-55)		1000 (411-1536)		12 (5-19)	
2010	Rajasthan									2788 (1146-4283)		34 (14-52)		940 (386-1444)		11 (5-18)	
2011	Rajasthan									2599 (1068-3991)		32 (13-49)		876 (360-1345)		11 (4-17)	
2012	Rajasthan									2390 (982-3670)		30 (12-46)		806 (331-1237)		10 (4-15)	
2013	Rajasthan									2222 (913-3412)		28 (11-43)		749 (308-1150)		9 (4-14)	
2014	Rajasthan									1938 (796-2975)		25 (10-38)		653 (268-1003)		8 (3-13)	
2015	Rajasthan	19340 (17658-31600)	49 (45-80)	247 (226-404)	1 (1-1)	3810 (2281-7689)	10 (6-19)	49 (29-98)	0 (0-0)	195 (80-300)	1 (0-1)	2 (1-4)	0 (0-0)	66 (27-101)	0 (0-0)	1 (0-1)	0 (0-0)
2000	Sikkim	1279 (1168-2090)		2105 (1922-3439)		252 (151-508)		414 (248-836)		16 (7-24)		26 (11-39)		5 (2-8)		9 (4-13)	
2001	Sikkim									15 (6-22)		24 (10-37)		5 (2-7)		8 (3-13)	
2002	Sikkim									13 (6-20)		23 (10-35)		5 (2-7)		8 (3-12)	
2003	Sikkim									12 (5-18)		21 (9-32)		4 (2-6)		7 (3-11)	
2004	Sikkim									10 (4-16)		18 (8-28)		3 (1-5)		6 (3-9)	
2005	Sikkim									9 (3-14)		16 (6-25)		3 (1-5)		5 (2-8)	
2006	Sikkim									7 (3-12)		14 (5-22)		2 (1-4)		5 (2-7)	
2007	Sikkim									6 (2-10)		12 (4-20)		2 (1-3)		4 (1-7)	
2008	Sikkim									6 (2-10)		12 (4-19)		2 (1-3)		4 (1-6)	
2009	Sikkim									6 (2-9)		12 (4-19)		2 (1-3)		4 (1-6)	
2010	Sikkim									6 (2-9)		12 (4-20)		2 (1-3)		4 (2-7)	
2011	Sikkim									6 (2-10)		14 (5-21)		2 (1-3)		5 (2-7)	
2012	Sikkim									7 (3-11)		15 (6-23)		2 (1-4)		5 (2-8)	
2013	Sikkim									7 (1-12)		17 (3-28)		3 (0-4)		6 (1-9)	
2014	Sikkim									8 (1-13)		18 (3-30)		3 (0-4)		6 (1-10)	
2015	Sikkim	194 (177-317)	0 (0-1)	467 (426-763)	1 (1-2)	38 (23-77)	0 (0-0)	92 (55-185)	0 (0-0)	7 (1-12)	0 (0-0)	17 (3-29)	0 (0-0)	2 (0-4)	0 (0-0)	6 (1-10)	0 (0-0)
2000	Tamil Nadu	109883 (100325-179538)		1900 (1734-3104)		21621 (12945-43638)		374 (224-754)		1492 (635-2272)		26 (11-39)		503 (214-766)		9 (4-13)	
2001	Tamil Nadu									1424 (606-2168)		24 (10-37)		480 (204-731)		8 (3-13)	
2002	Tamil Nadu									1355 (576-2063)		23 (10-35)		457 (194-695)		8 (3-12)	
2003	Tamil Nadu									1292 (550-1968)		22 (9-33)		436 (185-664)		7 (3-11)	
2004	Tamil Nadu									1236 (527-1887)		21 (9-32)		417 (178-636)		7 (3-11)	
2005	Tamil Nadu									1184 (518-1790)		20 (9-30)		399 (175-603)		7 (3-10)	
2006	Tamil Nadu									1132 (509-1695)		19 (9-29)		382 (172-572)		6 (3-10)	

Hib = *Haemophilus influenzae* type b; npnm = non-pneumonia, non-meningitis; HIV = HIV-infected cases only; UR = uncertainty range

Year	State	Hib pneumonia cases (UR)	Hib pneumonia cases, HIV (UR)	Hib pneumonia incidence rate (UR)	Hib pneumonia incidence rate, HIV (UR)	Hib severe pneumonia cases (UR)	Hib severe pneumonia cases; HIV (UR)	Hib severe pneumonia incidence rate (UR)	Hib severe pneumonia incidence rate; HIV (UR)	Hib meningitis cases (UR)	Hib meningitis cases; HIV (UR)	Hib meningitis incidence rate (UR)	Hib meningitis incidence rate; HIV (UR)	Hib NPNM cases (UR)	Hib NPNM cases; HIV (UR)	Hib NPNM incidence rate (UR)	Hib NPNM incidence rate; HIV (UR)
2007	Tamil Nadu									1062 (490-1572)		18 (8-26)		358 (165-530)		6 (3-9)	
2008	Tamil Nadu									998 (462-1483)		17 (8-25)		337 (156-500)		6 (3-8)	
2009	Tamil Nadu									936 (427-1410)		16 (7-24)		316 (144-475)		5 (2-8)	
2010	Tamil Nadu									879 (396-1342)		15 (7-23)		296 (134-452)		5 (2-8)	
2011	Tamil Nadu									834 (170-1353)		14 (3-23)		281 (57-456)		5 (1-8)	
2012	Tamil Nadu									189 (37-312)		3 (1-5)		64 (13-105)		1 (0-2)	
2013	Tamil Nadu									77 (15-128)		1 (0-2)		26 (5-43)		0 (0-1)	
2014	Tamil Nadu									63 (13-105)		1 (0-2)		21 (4-35)		0 (0-1)	
2015	Tamil Nadu	4455 (4067-7279)	11 (10-18)	78 (71-127)	0 (0-0)	877 (525-1770)	2 (1-4)	15 (9-31)	0 (0-0)	51 (10-85)	0 (0-0)	1 (0-1)	0 (0-0)	17 (3-29)	0 (0-0)	0 (0-0)	0 (0-0)
2000	Tripura	9794 (8943-16003)		2948 (2692-4817)		1927 (1154-3890)		580 (347-1171)		61 (22-97)		18 (7-29)		21 (8-33)		6 (2-10)	
2001	Tripura									55 (20-87)		16 (6-26)		19 (7-29)		6 (2-9)	
2002	Tripura									51 (18-80)		15 (5-23)		17 (6-27)		5 (2-8)	
2003	Tripura									47 (17-74)		14 (5-21)		16 (6-25)		5 (2-7)	
2004	Tripura									43 (16-69)		13 (5-20)		15 (5-23)		4 (2-7)	
2005	Tripura									41 (15-64)		12 (4-18)		14 (5-22)		4 (1-6)	
2006	Tripura									39 (15-61)		11 (4-17)		13 (5-21)		4 (1-6)	
2007	Tripura									39 (15-60)		11 (4-17)		13 (5-20)		4 (1-6)	
2008	Tripura									38 (15-59)		11 (4-16)		13 (5-20)		4 (1-6)	
2009	Tripura									38 (16-58)		11 (4-16)		13 (5-20)		4 (2-5)	
2010	Tripura									39 (17-58)		11 (5-16)		13 (6-20)		4 (2-5)	
2011	Tripura									40 (19-60)		11 (5-17)		14 (6-20)		4 (2-6)	
2012	Tripura									41 (20-60)		11 (6-17)		14 (7-20)		4 (2-6)	
2013	Tripura									42 (21-62)		12 (6-17)		14 (7-21)		4 (2-6)	
2014	Tripura									42 (11-64)		12 (3-18)		14 (4-21)		4 (1-6)	
2015	Tripura	4809 (4391-7857)	12 (11-20)	1346 (1229-2200)	3 (3-6)	948 (567-1913)	2 (1-5)	265 (159-536)	1 (0-1)	38 (10-59)	0 (0-0)	11 (3-17)	0 (0-0)	13 (3-20)	0 (0-0)	4 (1-6)	0 (0-0)
2000	Union Territory	7041 (6429-11504)		2532 (2312-4137)		1386 (830-2798)		499 (299-1006)		69 (27-105)		25 (10-38)		23 (9-35)		8 (3-13)	
2001	Union Territory									66 (26-102)		24 (9-36)		22 (9-34)		8 (3-12)	
2002	Union Territory									65 (25-100)		23 (9-35)		22 (9-34)		8 (3-12)	
2003	Union Territory									64 (21-101)		22 (7-35)		22 (7-34)		8 (2-12)	
2004	Union Territory									65 (21-102)		22 (7-36)		22 (7-35)		8 (2-12)	
2005	Union Territory									66 (22-104)		23 (8-36)		22 (7-35)		8 (3-12)	
2006	Union Territory									71 (26-108)		24 (9-37)		24 (9-37)		8 (3-13)	
2007	Union Territory									76 (30-110)		26 (10-38)		26 (10-37)		9 (3-13)	
2008	Union Territory									82 (35-124)		28 (12-42)		28 (12-42)		9 (4-14)	
2009	Union Territory									84 (38-133)		28 (13-45)		28 (13-45)		10 (4-15)	
2010	Union Territory									83 (38-135)		28 (13-46)		28 (13-45)		9 (4-15)	
2011	Union Territory									80 (35-129)		27 (12-44)		27 (12-43)		9 (4-15)	
2012	Union Territory									76 (32-122)		26 (11-41)		25 (11-41)		9 (4-14)	
2013	Union Territory									63 (29-101)		21 (10-34)		21 (10-34)		7 (3-12)	
2014	Union Territory									56 (26-90)		19 (9-31)		19 (9-30)		6 (3-10)	
2015	Union Territory	1026 (937-1677)	3 (2-4)	350 (320-572)	1 (1-1)	202 (121-408)	1 (0-1)	69 (41-139)	0 (0-0)	17 (7-26)	0 (0-0)	6 (2-9)	0 (0-0)	6 (2-9)	0 (0-0)	2 (1-3)	0 (0-0)
2000	Uttar Pradesh	903893 (825274-1476875)		3825 (3492-6249)		178093 (106633-359458)		754 (451-1521)		17774 (8203-26473)		75 (35-112)		3061 (1413-4559)		13 (6-19)	
2001	Uttar Pradesh									17034 (7862-25370)		72 (33-107)		2933 (1354-4369)		12 (6-18)	
2002	Uttar Pradesh									16259 (7503-24214)		68 (32-102)		2800 (1292-4170)		12 (5-18)	
2003	Uttar Pradesh									15477 (7140-23042)		65 (30-97)		2665 (1230-3968)		11 (5-17)	
2004	Uttar Pradesh									14635 (6748-21776)		62 (29-92)		2520 (1162-3750)		11 (5-16)	
2005	Uttar Pradesh									13707 (6261-20456)		58 (27-87)		2360 (1078-3522)		10 (5-15)	
2006	Uttar Pradesh									12826 (5803-19194)		55 (25-82)		2209 (999-3305)		9 (4-14)	
2007	Uttar Pradesh									11916 (5341-17882)		51 (23-76)		2052 (920-3079)		9 (4-13)	
2008	Uttar Pradesh									11155 (4997-16728)		48 (21-72)		1921 (860-2881)		8 (4-12)	
2009	Uttar Pradesh									10530 (4714-15782)		46 (20-68)		1813 (812-2718)		8 (4-12)	
2010	Uttar Pradesh									9961 (4458-14924)		43 (19-65)		1715 (768-2570)		7 (3-11)	
2011	Uttar Pradesh									9471 (3938-14514)		42 (17-64)		3193 (1328-4893)		14 (6-22)	
2012	Uttar Pradesh									8866 (3685-13580)		40 (16-61)		2989 (1242-4578)		13 (6-20)	
2013	Uttar Pradesh									8350 (3470-12787)		38 (16-58)		2815 (1170-4310)		13 (5-19)	
2014	Uttar Pradesh									7783 (3234-11917)		36 (15-55)		2624 (1090-4017)		12 (5-18)	
2015	Uttar Pradesh	561834 (512966-917982)	1419 (1296-2319)	2600 (2374-4248)	7 (6-11)	110635 (66243-223303)	280 (167-564)	512 (307-1033)	1 (1-3)	7313 (3039-11198)	20 (8-30)	34 (14-52)	0 (0-0)	2465 (1024-3775)	7 (3-10)	11 (5-17)	0 (0-0)
2000	Uttarakhand	40142 (36651-65589)		3825 (3492-6249)		7909 (4736-15964)		754 (451-1521)		397 (182-592)		38 (17-56)		68 (31-102)		7 (3-10)	
2001	Uttarakhand									384 (176-572)		36 (17-54)		66 (30-99)		6 (3-9)	
2002	Uttarakhand									370 (170-551)		35 (16-52)		64 (29-95)		6 (3-9)	
2003	Uttarakhand									356 (152-540)		34 (14-51)		120 (51-182)		11 (5-17)	
2004	Uttarakhand									335 (143-507)		32 (13-48)		113 (48-171)		11 (5-16)	
2005	Uttarakhand									308 (126-472)		29 (12-45)		104 (42-159)		10 (4-15)	
2006	Uttarakhand									280 (110-435)		27 (10-41)		94 (37-147)		9 (4-14)	
2007	Uttarakhand									253 (96-397)		24 (9-38)		85 (32-134)		8 (3-13)	
2008	Uttarakhand									232 (88-363)		22 (8-35)		78 (30-122)		7 (3-12)	
2009	Uttarakhand									213 (81-334)		20 (8-32)		72 (27-113)		7 (3-11)	
2010	Uttarakhand									196 (74-307)		19 (7-30)		66 (25-103)		6 (2-10)	
2011	Uttarakhand									178 (68-279)		17 (7-27)		60 (23-94)		6 (2-9)	
2012	Uttarakhand									158 (60-248)		16 (6-24)		53 (20-83)		5 (2-8)	
2013	Uttarakhand									141 (53-220)		14 (5-22)		47 (18-74)		5 (2-7)	
2014	Uttarakhand									123 (47-193)		12 (5-19)		42 (16-65)		4 (2-7)	
2015	Uttarakhand	2622 (2394-4284)	7 (6-11)	265 (242-433)	1 (1-1)	517 (309-1043)	1 (1-3)	52 (31-105)	0 (0-0)	18 (7-28)	0 (0-0)	2 (1-3)	0 (0-0)	6 (2-10)	0 (0-0)	1 (0-1)	0 (0-0)
2000	West Bengal	277643 (253494-453642)		3190 (2912-5212)		54670 (32734-110345)		628 (376-1268)		3431 (1503-5182)		39 (17-60)		1157 (507-1747)		13 (6-20)	
2001	West Bengal									3134 (1373-4734)		36 (16-54)		1057 (463-1596)		12 (5-18)	
2002	West Bengal									2823 (1237-4265)		32 (14-49)		952 (417-1438)		11 (5-17)	
2003	West Bengal									2555 (1120-3861)		29 (13-44)		861 (377-1301)		10 (4-15)	
2004	West Bengal									2335 (1023-3528)		27 (12-41)		787 (345-1189)		9 (4-14)	
2005	West Bengal									2143 (945-3233)		25 (11-38)		723 (318-1090)		8 (4-13)	
2006	West Bengal									1977 (877-2977)		23 (10-35)		667 (295-1004)		8 (3-12)	
2007	West Bengal									1817 (810-2730)		21 (10-32)		612 (273-920)		7 (3-11)	
2008	West Bengal									1660 (741-2496)		20 (9-30)		560 (250-841)		7 (3-10)	
2009	West Bengal									1516 (681-2275)		18 (8-27)		511 (230-767)		6 (3-9)	
2010	West Bengal									1428 (647-2138)		17 (8-26)		481 (218-721)		6 (3-9)	
2011	West Bengal									1392 (635-2079)		17 (8-25)		469 (214-701)		6 (3-9)	
2012	West Bengal									1361 (625-2027)		17 (8-25)		459 (211-683)		6 (3-8)	
2013	West Bengal									1371 (630-2043)		17 (8-26)		462 (212-689)		6 (3-9)	
2014	West Bengal									1384 (636-2063)		18 (8-26)		467 (214-695)		6 (3-9)	
2015	West Bengal	19672 (17961-32142)	50 (45-81)	255 (233-417)	1 (1-1)	3875 (2320-7821)	10 (6-20)	50 (30-101)	0 (0-0)	221 (102-329)	1 (0-1)	3 (1-4)	0 (0-0)	74 (34-111)	0 (0-0)	1 (0-1)	0 (0-0)

Hib = *Haemophilus influenzae* type b; npnm = non-pneumonia, non-meningitis; HIV = HIV-infected cases only; UR = uncertainty range

Webappendix 13: Pneumococcal pneumonia mortality sensitivity results in India by state and year

		Base case			VT AOM vaccine efficacy adjustment			VT pneumococcal pneumonia efficacy adjustment (from Dutch elderly trial)		
Year	State	Spn pna deaths	Spn pna deaths_lb	Spn pna deaths_ub	Spn pna deaths	Spn pna deaths_lb	Spn pna deaths_ub	Spn pna deaths	Spn pna deaths_lb	Spn pna deaths_ub
2000	Andhra Pradesh	6780	4582	7980	9542	6250	10951	10786	6562	11553
2001	Andhra Pradesh	7093	4645	8751	9782	6269	11644	10503	6390	11249
2002	Andhra Pradesh	6849	4487	8454	9447	6056	11249	10147	6173	10868
2003	Andhra Pradesh	6562	4300	8102	9052	5804	10781	9725	5916	10415
2004	Andhra Pradesh	6237	4089	7704	8604	5518	10251	9246	5625	9903
2005	Andhra Pradesh	5891	3861	7280	8128	5212	9686	8736	5314	9356
2006	Andhra Pradesh	5540	3629	6848	7644	4900	9111	8216	4998	8800
2007	Andhra Pradesh	5163	3383	6387	7125	4567	8497	7661	4661	8205
2008	Andhra Pradesh	4785	3137	5923	6604	4235	7880	7105	4322	7609
2009	Andhra Pradesh	4380	2860	5439	6047	3867	7232	6511	3961	6973
2010	Andhra Pradesh	3965	2580	4939	5476	3493	6564	5901	3590	6320
2011	Andhra Pradesh	3559	2309	4447	4917	3129	5908	5303	3226	5680
2012	Andhra Pradesh	3140	2030	3934	4339	2754	5224	4683	2849	5016
2013	Andhra Pradesh	2800	1812	3511	3870	2458	4662	4179	2542	4476
2014	Andhra Pradesh	2513	1626	3151	3473	2206	4184	3751	2282	4017
2015	Andhra Pradesh	2337	1513	2932	3231	2052	3893	3490	2123	3738
2000	Arunachal Pradesh	99	63	128	138	86	169	150	91	161
2001	Arunachal Pradesh	93	59	120	129	81	159	141	86	151
2002	Arunachal Pradesh	93	59	119	128	80	158	140	85	150
2003	Arunachal Pradesh	94	59	121	130	81	160	142	86	152
2004	Arunachal Pradesh	93	59	120	130	81	159	141	86	151
2005	Arunachal Pradesh	92	59	118	128	80	156	139	84	149
2006	Arunachal Pradesh	97	63	122	134	85	161	145	88	155
2007	Arunachal Pradesh	92	61	114	127	82	152	137	83	147
2008	Arunachal Pradesh	93	61	115	128	83	153	138	84	148
2009	Arunachal Pradesh	93	60	117	129	82	155	139	85	149
2010	Arunachal Pradesh	92	59	116	127	80	154	138	84	148
2011	Arunachal Pradesh	88	56	113	122	77	150	133	81	142
2012	Arunachal Pradesh	84	53	109	117	73	144	127	77	136
2013	Arunachal Pradesh	81	51	104	112	70	138	122	74	131

Spn = *Streptococcus pneumoniae* ; pna = pneumonia; hiv = HIV-infected deaths only; lb = lower bound; ub = upper bound

Spn pneumonia mortality sensitivity results in India by state and year

Year	State	Spn pna deaths	Spn pna deaths_lb	Spn pna deaths_ub	Spn pna deaths	Spn pna deaths_lb	Spn pna deaths_ub	Spn pna deaths	Spn pna deaths_lb	Spn pna deaths_ub
2014	Arunachal Pradesh	77	49	100	107	67	132	116	71	125
2015	Arunachal Pradesh	72	46	93	100	63	124	109	67	117
2000	Assam	5014	3325	6050	7078	4571	8270	8061	4904	8634
2001	Assam	4843	3211	5843	6837	4415	7988	7786	4737	8339
2002	Assam	4709	3122	5681	6647	4292	7766	7570	4605	8108
2003	Assam	4616	3060	5569	6516	4207	7612	7420	4514	7947
2004	Assam	4547	3013	5484	6418	4143	7496	7307	4445	7826
2005	Assam	4489	2979	5407	6336	4094	7394	7212	4387	7724
2006	Assam	4432	2945	5330	6254	4045	7290	7115	4329	7621
2007	Assam	4399	2928	5283	6207	4019	7228	7060	4295	7561
2008	Assam	4376	2911	5254	6173	3997	7187	7020	4271	7519
2009	Assam	4318	2873	5185	6092	3944	7093	6928	4215	7420
2010	Assam	4280	2847	5138	6038	3908	7029	6865	4177	7353
2011	Assam	4242	2822	5092	5984	3874	6966	6804	4139	7288
2012	Assam	4173	2776	5010	5887	3811	6854	6695	4073	7170
2013	Assam	4164	2771	5001	5875	3804	6841	6682	4065	7157
2014	Assam	4117	2740	4945	5809	3762	6765	6608	4020	7077
2015	Assam	4359	2784	5532	6032	3793	7331	6532	3974	6996
2000	Bihar	18419	12431	21620	25905	16951	29672	29235	17786	31312
2001	Bihar	17945	12111	21063	25239	16515	28908	28483	17328	30506
2002	Bihar	17382	11732	20403	24447	15997	28002	27590	16785	29550
2003	Bihar	16778	11328	19701	23600	15446	27038	26640	16207	28532
2004	Bihar	16138	10903	18961	22704	14867	26023	25640	15598	27462
2005	Bihar	15504	10468	18245	21818	14280	25036	24656	15000	26407
2006	Bihar	15990	10441	19762	22055	14103	26286	23687	14410	25370
2007	Bihar	15318	9992	18974	21136	13505	25232	22722	13823	24336
2008	Bihar	14627	9550	18134	20188	12908	24116	21717	13211	23259
2009	Bihar	13856	9051	17186	19126	12233	22854	20581	12520	22042
2010	Bihar	13082	8546	16227	18058	11550	21580	19433	11822	20813
2011	Bihar	12263	8011	15211	16928	10827	20228	18216	11082	19510
2012	Bihar	11290	7375	14005	15585	9968	18624	16771	10203	17962
2013	Bihar	10431	6814	12939	14399	9210	17207	15495	9426	16595

Spn = *Streptococcus pneumoniae* ; pna = pneumonia; hiv = HIV-infected deaths only; lb = lower bound; ub = upper bound

Spn pneumonia mortality sensitivity results in India by state and year

Year	State	Spn pna deaths	Spn pna deaths_lb	Spn pna deaths_ub	Spn pna deaths	Spn pna deaths_lb	Spn pna deaths_ub	Spn pna deaths	Spn pna deaths_lb	Spn pna deaths_ub
2014	Bihar	9479	6192	11758	13085	8369	15636	14081	8566	15081
2015	Bihar	8606	5622	10675	11880	7598	14196	12784	7777	13692
2000	Chhattisgarh	3406	2261	4101	4807	3107	5608	5471	3328	5860
2001	Chhattisgarh	3374	2240	4063	4762	3078	5556	5420	3297	5805
2002	Chhattisgarh	3277	2175	3946	4625	2989	5395	5263	3202	5637
2003	Chhattisgarh	3164	2100	3809	4465	2885	5208	5080	3091	5441
2004	Chhattisgarh	2990	1983	3597	4219	2725	4919	4798	2919	5139
2005	Chhattisgarh	2835	1888	3399	4000	2591	4652	4546	2766	4869
2006	Chhattisgarh	2746	1837	3281	3873	2517	4493	4400	2677	4712
2007	Chhattisgarh	2738	1775	3430	3784	2407	4556	4086	2486	4377
2008	Chhattisgarh	2597	1683	3252	3589	2282	4319	3875	2357	4150
2009	Chhattisgarh	2414	1565	3023	3336	2121	4015	3602	2191	3857
2010	Chhattisgarh	2220	1439	2780	3069	1951	3693	3313	2015	3548
2011	Chhattisgarh	2078	1347	2602	2872	1826	3456	3100	1886	3320
2012	Chhattisgarh	1924	1247	2409	2659	1691	3200	2871	1746	3074
2013	Chhattisgarh	1851	1199	2317	2558	1626	3078	2761	1680	2957
2014	Chhattisgarh	1831	1186	2292	2530	1609	3045	2731	1661	2925
2015	Chhattisgarh	1881	1219	2356	2600	1653	3129	2807	1708	3006
2000	Delhi	1061	683	1338	1467	929	1775	1587	966	1700
2001	Delhi	1020	657	1286	1410	893	1706	1526	928	1634
2002	Delhi	975	628	1229	1348	853	1631	1458	887	1562
2003	Delhi	928	597	1169	1283	812	1551	1387	844	1486
2004	Delhi	879	565	1105	1215	768	1467	1312	798	1405
2005	Delhi	831	541	1035	1148	732	1375	1236	752	1324
2006	Delhi	784	518	964	1082	697	1284	1161	706	1244
2007	Delhi	736	495	893	1013	663	1191	1084	659	1161
2008	Delhi	687	460	830	945	616	1108	1008	613	1080
2009	Delhi	635	424	766	873	568	1022	930	565	996
2010	Delhi	582	388	701	800	520	935	851	518	911
2011	Delhi	529	352	635	726	471	847	771	469	826
2012	Delhi	470	309	600	645	415	788	683	416	732
2013	Delhi	416	273	529	570	366	695	602	366	645

Spn = *Streptococcus pneumoniae* ; pna = pneumonia; hiv = HIV-infected deaths only; lb = lower bound; ub = upper bound

Spn pneumonia mortality sensitivity results in India by state and year

Year	State	Spn pna deaths	Spn pna deaths_lb	Spn pna deaths_ub	Spn pna deaths	Spn pna deaths_lb	Spn pna deaths_ub	Spn pna deaths	Spn pna deaths_lb	Spn pna deaths_ub
2014	Delhi	357	234	453	489	313	595	516	314	553
2015	Delhi	303	198	385	415	266	505	438	266	469
2000	Goa	23	15	29	31	20	39	33	20	36
2001	Goa	19	12	25	26	17	32	28	17	30
2002	Goa	13	9	17	18	12	23	19	12	21
2003	Goa	17	11	22	23	15	28	24	15	26
2004	Goa	17	11	22	24	15	29	25	15	27
2005	Goa	18	11	23	24	15	30	26	16	28
2006	Goa	16	10	20	21	14	27	23	14	25
2007	Goa	11	7	14	15	9	18	16	10	17
2008	Goa	9	6	12	13	8	16	13	8	14
2009	Goa	10	7	14	14	9	18	15	9	16
2010	Goa	10	7	13	14	9	18	15	9	16
2011	Goa	8	5	10	11	7	13	12	7	12
2012	Goa	6	4	7	8	5	10	9	5	9
2013	Goa	4	3	5	6	4	7	6	4	7
2014	Goa	3	2	4	4	3	5	5	3	5
2015	Goa	2	1	3	3	2	4	3	2	4
2000	Gujarat	6676	4438	8027	9421	6095	10980	10718	6521	11480
2001	Gujarat	6355	4226	7644	8969	5804	10455	10207	6209	10932
2002	Gujarat	6050	4024	7279	8539	5527	9956	9719	5913	10410
2003	Gujarat	6108	3894	7764	8453	5310	10287	9158	5572	9809
2004	Gujarat	5653	3602	7182	7822	4912	9515	8472	5154	9073
2005	Gujarat	5382	3459	6793	7444	4704	9011	8053	4899	8624
2006	Gujarat	5172	3356	6479	7149	4550	8606	7721	4697	8270
2007	Gujarat	4854	3185	6030	6705	4303	8021	7228	4397	7741
2008	Gujarat	4560	2987	5655	6296	4035	7522	6778	4123	7259
2009	Gujarat	4235	2771	5246	5845	3743	6978	6288	3825	6734
2010	Gujarat	3908	2556	4839	5393	3453	6437	5800	3528	6212
2011	Gujarat	3634	2377	4500	5015	3211	5985	5393	3281	5776
2012	Gujarat	3384	2214	4191	4671	2990	5575	5023	3056	5380
2013	Gujarat	3228	2111	3997	4455	2852	5317	4791	2915	5131

Spn = *Streptococcus pneumoniae* ; pna = pneumonia; hiv = HIV-infected deaths only; lb = lower bound; ub = upper bound

Spn pneumonia mortality sensitivity results in India by state and year

Year	State	Spn pna deaths	Spn pna deaths_lb	Spn pna deaths_ub	Spn pna deaths	Spn pna deaths_lb	Spn pna deaths_ub	Spn pna deaths	Spn pna deaths_lb	Spn pna deaths_ub
2014	Gujarat	3071	2009	3804	4239	2714	5059	4559	2773	4883
2015	Gujarat	2919	1910	3615	4029	2580	4809	4333	2636	4641
2000	Haryana	3729	2508	4401	5249	3426	6036	5936	3611	6357
2001	Haryana	3541	2382	4180	4984	3253	5732	5637	3429	6037
2002	Haryana	3336	2245	3939	4696	3066	5402	5312	3232	5689
2003	Haryana	3119	2099	3683	4391	2867	5051	4966	3021	5319
2004	Haryana	3106	2017	3845	4283	2728	5112	4599	2798	4926
2005	Haryana	2820	1848	3472	3888	2493	4620	4170	2537	4467
2006	Haryana	2525	1671	3087	3479	2247	4114	3725	2266	3990
2007	Haryana	2398	1605	2911	3302	2151	3884	3530	2147	3781
2008	Haryana	2210	1477	2679	3042	1979	3574	3249	1976	3479
2009	Haryana	1962	1307	2379	2700	1752	3173	2882	1753	3087
2010	Haryana	1899	1262	2306	2613	1693	3075	2791	1698	2989
2011	Haryana	1789	1187	2177	2463	1594	2902	2632	1601	2819
2012	Haryana	1572	1041	1916	2165	1399	2553	2314	1408	2478
2013	Haryana	1595	1057	1944	2197	1420	2591	2349	1429	2516
2014	Haryana	1562	1035	1904	2151	1391	2538	2301	1400	2464
2015	Haryana	1580	1047	1926	2175	1406	2567	2327	1415	2492
2000	Himachal Pradesh	702	458	872	970	620	1160	1044	635	1118
2001	Himachal Pradesh	650	424	807	897	573	1073	965	587	1034
2002	Himachal Pradesh	600	391	744	828	529	990	891	542	954
2003	Himachal Pradesh	554	361	687	765	488	914	823	501	881
2004	Himachal Pradesh	512	334	635	706	451	844	760	462	814
2005	Himachal Pradesh	473	310	584	652	418	777	701	426	751
2006	Himachal Pradesh	437	287	538	602	387	716	646	393	692
2007	Himachal Pradesh	403	266	494	555	358	658	595	362	638
2008	Himachal Pradesh	372	246	455	513	330	607	549	334	588
2009	Himachal Pradesh	343	228	417	472	305	556	504	307	540
2010	Himachal Pradesh	316	211	382	435	283	510	463	282	496
2011	Himachal Pradesh	293	197	352	403	264	470	428	261	459
2012	Himachal Pradesh	272	182	325	374	244	434	397	241	425
2013	Himachal Pradesh	261	175	311	358	233	415	380	231	407

Spn = *Streptococcus pneumoniae* ; pna = pneumonia; hiv = HIV-infected deaths only; lb = lower bound; ub = upper bound

Spn pneumonia mortality sensitivity results in India by state and year

Year	State	Spn pna deaths	Spn pna deaths_lb	Spn pna deaths_ub	Spn pna deaths	Spn pna deaths_lb	Spn pna deaths_ub	Spn pna deaths	Spn pna deaths_lb	Spn pna deaths_ub
2014	Himachal Pradesh	255	170	303	350	228	405	370	225	397
2015	Himachal Pradesh	257	172	305	353	229	408	373	227	400
2000	Jammu & Kashmir	858	569	1011	1176	760	1353	1240	754	1328
2001	Jammu & Kashmir	807	535	951	1106	715	1272	1166	709	1249
2002	Jammu & Kashmir	758	503	894	1039	672	1196	1096	667	1174
2003	Jammu & Kashmir	712	473	842	977	633	1126	1032	628	1105
2004	Jammu & Kashmir	669	446	792	917	596	1060	971	591	1040
2005	Jammu & Kashmir	626	421	750	860	562	1001	914	556	978
2006	Jammu & Kashmir	585	390	708	805	522	944	858	522	919
2007	Jammu & Kashmir	546	359	666	751	483	887	803	488	860
2008	Jammu & Kashmir	508	335	621	700	451	827	749	455	802
2009	Jammu & Kashmir	469	309	573	646	416	764	691	421	740
2010	Jammu & Kashmir	428	283	524	590	381	698	632	385	677
2011	Jammu & Kashmir	387	256	474	534	344	632	572	348	612
2012	Jammu & Kashmir	344	227	421	474	305	560	507	309	543
2013	Jammu & Kashmir	309	204	378	425	274	503	456	277	488
2014	Jammu & Kashmir	281	185	344	387	249	458	414	252	444
2015	Jammu & Kashmir	267	176	326	368	237	435	394	239	422
2000	Jharkhand	3690	2446	4470	5213	3365	6108	5947	3618	6369
2001	Jharkhand	3884	2463	4984	5382	3368	6596	5850	3559	6266
2002	Jharkhand	3768	2390	4837	5221	3268	6400	5677	3454	6080
2003	Jharkhand	3697	2346	4747	5124	3207	6282	5571	3389	5967
2004	Jharkhand	3470	2202	4456	4809	3010	5896	5230	3181	5601
2005	Jharkhand	3147	2000	4037	4362	2733	5343	4742	2885	5079
2006	Jharkhand	3107	1976	3981	4305	2700	5270	4679	2847	5012
2007	Jharkhand	2879	1833	3684	3988	2504	4878	4334	2637	4642
2008	Jharkhand	2574	1639	3294	3566	2238	4361	3875	2357	4150
2009	Jharkhand	2282	1453	2921	3162	1985	3867	3436	2090	3680
2010	Jharkhand	2307	1469	2952	3197	2006	3909	3473	2113	3720
2011	Jharkhand	2203	1403	2819	3052	1915	3732	3316	2017	3552
2012	Jharkhand	2049	1304	2621	2838	1781	3470	3084	1876	3303
2013	Jharkhand	1869	1190	2391	2589	1625	3166	2813	1711	3013

Spn = *Streptococcus pneumoniae* ; pna = pneumonia; hiv = HIV-infected deaths only; lb = lower bound; ub = upper bound

Spn pneumonia mortality sensitivity results in India by state and year

Year	State	Spn pna deaths	Spn pna deaths_lb	Spn pna deaths_ub	Spn pna deaths	Spn pna deaths_lb	Spn pna deaths_ub	Spn pna deaths	Spn pna deaths_lb	Spn pna deaths_ub
2014	Jharkhand	1679	1069	2149	2327	1460	2845	2528	1538	2707
2015	Jharkhand	1492	950	1909	2067	1297	2528	2246	1366	2406
2000	Karnataka	5330	3449	6687	7368	4680	8879	7959	4842	8524
2001	Karnataka	5088	3293	6383	7033	4467	8476	7597	4622	8137
2002	Karnataka	4942	3197	6199	6831	4338	8230	7377	4488	7901
2003	Karnataka	4803	3106	6022	6639	4215	7997	7168	4360	7677
2004	Karnataka	4652	3006	5828	6428	4078	7738	6936	4220	7429
2005	Karnataka	4473	2905	5580	6178	3935	7415	6660	4052	7133
2006	Karnataka	4278	2794	5313	5907	3777	7065	6360	3869	6812
2007	Karnataka	4039	2653	4992	5574	3580	6643	5994	3646	6419
2008	Karnataka	3779	2477	4661	5212	3343	6203	5597	3405	5994
2009	Karnataka	3492	2298	4289	4813	3096	5711	5161	3140	5528
2010	Karnataka	3182	2103	3893	4385	2829	5186	4695	2856	5028
2011	Karnataka	2865	1902	3489	3945	2554	4651	4219	2566	4518
2012	Karnataka	2494	1665	3026	3433	2232	4036	3667	2231	3927
2013	Karnataka	2124	1416	2574	2923	1899	3433	3119	1898	3341
2014	Karnataka	1711	1140	2073	2354	1529	2764	2512	1528	2690
2015	Karnataka	1331	887	1611	1831	1188	2149	1952	1188	2091
2000	Kerala	484	307	639	667	417	836	715	435	766
2001	Kerala	467	296	617	644	403	807	690	420	739
2002	Kerala	452	286	596	622	389	779	667	406	714
2003	Kerala	438	277	577	604	377	755	646	393	692
2004	Kerala	426	269	560	587	366	733	627	381	671
2005	Kerala	417	266	543	574	361	712	611	372	655
2006	Kerala	412	267	530	565	360	696	600	365	643
2007	Kerala	408	269	519	559	361	682	592	360	634
2008	Kerala	406	267	515	557	358	676	587	357	629
2009	Kerala	404	265	511	554	355	672	583	355	624
2010	Kerala	403	264	509	552	354	669	581	353	622
2011	Kerala	402	263	508	550	353	668	579	353	621
2012	Kerala	398	261	504	545	349	662	574	349	615
2013	Kerala	400	262	507	548	351	666	577	351	618

Spn = *Streptococcus pneumoniae* ; pna = pneumonia; hiv = HIV-infected deaths only; lb = lower bound; ub = upper bound

Spn pneumonia mortality sensitivity results in India by state and year

Year	State	Spn pna deaths	Spn pna deaths_lb	Spn pna deaths_ub	Spn pna deaths	Spn pna deaths_lb	Spn pna deaths_ub	Spn pna deaths	Spn pna deaths_lb	Spn pna deaths_ub
2014	Kerala	399	261	506	547	350	664	576	350	617
2015	Kerala	403	264	510	552	354	670	581	354	623
2000	Madhya Pradesh	16532	10942	20001	23346	15056	27330	26608	16187	28497
2001	Madhya Pradesh	15824	10473	19144	22345	14410	26159	25467	15493	27276
2002	Madhya Pradesh	15163	10035	18343	21411	13807	25064	24401	14845	26134
2003	Madhya Pradesh	14505	9597	17542	20480	13204	23970	23336	14197	24994
2004	Madhya Pradesh	13831	9146	16719	19526	12584	22844	22240	13530	23820
2005	Madhya Pradesh	13199	8753	15910	18629	12031	21751	21207	12901	22713
2006	Madhya Pradesh	12573	8362	15109	17741	11482	20668	20182	12278	21615
2007	Madhya Pradesh	11920	7954	14281	16815	10909	19546	19116	11630	20474
2008	Madhya Pradesh	11278	7521	13503	15906	10315	18482	18075	10996	19359
2009	Madhya Pradesh	10619	7079	12711	14976	9710	17397	17015	10351	18223
2010	Madhya Pradesh	9980	6653	11946	14075	9126	16350	15991	9728	17126
2011	Madhya Pradesh	9398	6268	11253	13255	8596	15402	15063	9164	16133
2012	Madhya Pradesh	8813	5880	10558	12432	8066	14451	14133	8598	15137
2013	Madhya Pradesh	9030	5789	11420	12492	7879	15143	13518	8224	14478
2014	Madhya Pradesh	8597	5515	10879	11895	7506	14426	12877	7834	13792
2015	Madhya Pradesh	8239	5288	10430	11400	7196	13831	12346	7511	13223
2000	Maharashtra	7569	4938	9404	10450	6677	12503	11252	6845	12051
2001	Maharashtra	7062	4607	8774	9750	6230	11665	10498	6387	11244
2002	Maharashtra	6554	4275	8141	9048	5781	10824	9741	5926	10433
2003	Maharashtra	6049	3944	7511	8350	5333	9986	8987	5467	9625
2004	Maharashtra	5560	3622	6898	7674	4898	9171	8254	5021	8840
2005	Maharashtra	5108	3344	6311	7046	4515	8396	7571	4606	8109
2006	Maharashtra	4665	3072	5739	6433	4139	7640	6904	4200	7394
2007	Maharashtra	4226	2799	5175	5825	3765	6895	6244	3799	6687
2008	Maharashtra	3830	2534	4685	5277	3408	6242	5652	3439	6054
2009	Maharashtra	3424	2264	4184	4717	3044	5575	5049	3071	5407
2010	Maharashtra	3025	1998	3693	4167	2687	4921	4456	2711	4773
2011	Maharashtra	2710	1788	3304	3731	2405	4403	3987	2426	4270
2012	Maharashtra	2360	1523	3071	3247	2058	4026	3465	2108	3711
2013	Maharashtra	2051	1321	2664	2820	1786	3492	3006	1829	3219

Spn = *Streptococcus pneumoniae* ; pna = pneumonia; hiv = HIV-infected deaths only; lb = lower bound; ub = upper bound

Spn pneumonia mortality sensitivity results in India by state and year

Year	State	Spn pna deaths	Spn pna deaths_lb	Spn pna deaths_ub	Spn pna deaths	Spn pna deaths_lb	Spn pna deaths_ub	Spn pna deaths	Spn pna deaths_lb	Spn pna deaths_ub
2014	Maharashtra	1753	1128	2274	2410	1524	2981	2566	1561	2748
2015	Maharashtra	1515	974	1964	2082	1316	2574	2216	1348	2373
2000	Manipur	101	64	131	141	88	173	153	93	164
2001	Manipur	97	61	125	134	84	165	146	89	157
2002	Manipur	92	58	119	128	80	158	139	85	149
2003	Manipur	88	54	121	122	74	158	133	81	142
2004	Manipur	84	51	116	117	71	152	127	77	136
2005	Manipur	82	50	114	114	69	148	124	75	133
2006	Manipur	80	49	111	111	68	145	121	74	130
2007	Manipur	80	49	111	111	67	144	121	73	129
2008	Manipur	80	49	111	111	67	144	121	74	130
2009	Manipur	80	49	111	111	68	144	121	74	130
2010	Manipur	81	50	112	112	68	145	122	74	131
2011	Manipur	81	50	112	113	69	146	123	75	131
2012	Manipur	81	50	111	112	69	144	122	74	130
2013	Manipur	80	49	110	111	68	143	121	73	129
2014	Manipur	78	48	106	108	66	139	117	71	125
2015	Manipur	75	46	102	104	64	134	113	69	121
2000	Meghalaya	492	331	582	693	452	797	784	477	840
2001	Meghalaya	512	344	605	721	471	829	815	496	873
2002	Meghalaya	533	358	629	750	489	863	848	516	909
2003	Meghalaya	553	371	652	778	507	895	879	535	942
2004	Meghalaya	571	383	673	803	524	923	908	552	972
2005	Meghalaya	588	396	692	828	541	950	935	569	1001
2006	Meghalaya	604	408	710	850	556	974	959	584	1027
2007	Meghalaya	618	418	724	869	570	995	981	597	1051
2008	Meghalaya	634	430	744	892	585	1021	1007	613	1079
2009	Meghalaya	639	430	754	900	588	1035	1018	619	1090
2010	Meghalaya	666	446	791	939	611	1084	1064	647	1139
2011	Meghalaya	688	459	821	970	629	1125	1100	669	1179
2012	Meghalaya	705	468	846	995	643	1158	1130	688	1210
2013	Meghalaya	732	486	879	1033	668	1203	1174	714	1258

Spn = *Streptococcus pneumoniae* ; pna = pneumonia; hiv = HIV-infected deaths only; lb = lower bound; ub = upper bound

Spn pneumonia mortality sensitivity results in India by state and year

Year	State	Spn pna deaths	Spn pna deaths_lb	Spn pna deaths_ub	Spn pna deaths	Spn pna deaths_lb	Spn pna deaths_ub	Spn pna deaths	Spn pna deaths_lb	Spn pna deaths_ub
2014	Meghalaya	755	502	907	1065	689	1240	1211	737	1297
2015	Meghalaya	781	519	938	1101	713	1283	1253	762	1342
2000	Mizoram	153	101	186	216	139	254	247	150	264
2001	Mizoram	148	98	180	209	135	245	239	145	255
2002	Mizoram	145	96	176	205	132	241	234	142	251
2003	Mizoram	143	95	174	202	130	237	231	140	247
2004	Mizoram	141	93	172	200	129	234	228	139	244
2005	Mizoram	140	92	169	197	127	231	225	137	241
2006	Mizoram	139	92	168	196	126	230	223	136	239
2007	Mizoram	138	91	167	195	126	229	222	135	238
2008	Mizoram	137	91	166	194	125	227	221	134	236
2009	Mizoram	136	90	164	191	123	224	218	133	233
2010	Mizoram	133	89	161	188	122	220	214	130	230
2011	Mizoram	134	89	161	189	122	220	215	131	230
2012	Mizoram	132	88	159	186	121	217	212	129	227
2013	Mizoram	132	88	158	186	121	217	212	129	227
2014	Mizoram	130	86	156	183	119	213	208	127	223
2015	Mizoram	136	87	172	188	118	228	204	124	218
2000	Nagaland	107	67	138	148	92	182	161	98	173
2001	Nagaland	113	72	147	157	98	194	171	104	184
2002	Nagaland	121	77	157	168	105	207	183	111	196
2003	Nagaland	129	82	167	179	112	221	195	119	209
2004	Nagaland	137	86	177	190	118	234	207	126	221
2005	Nagaland	142	89	184	197	123	243	215	131	230
2006	Nagaland	145	91	189	201	125	249	220	134	235
2007	Nagaland	147	92	192	204	127	253	223	136	239
2008	Nagaland	149	93	194	206	128	256	225	137	241
2009	Nagaland	145	91	190	201	125	251	220	134	236
2010	Nagaland	151	94	198	209	129	261	229	139	245
2011	Nagaland	150	93	197	208	128	260	228	139	244
2012	Nagaland	146	91	193	203	125	254	222	135	238
2013	Nagaland	142	88	187	197	121	246	216	131	231

Spn = *Streptococcus pneumoniae* ; pna = pneumonia; hiv = HIV-infected deaths only; lb = lower bound; ub = upper bound

Spn pneumonia mortality sensitivity results in India by state and year

Year	State	Spn pna deaths	Spn pna deaths_lb	Spn pna deaths_ub	Spn pna deaths	Spn pna deaths_lb	Spn pna deaths_ub	Spn pna deaths	Spn pna deaths_lb	Spn pna deaths_ub
2014	Nagaland	134	83	176	186	114	232	203	124	218
2015	Nagaland	125	78	165	174	107	217	190	116	204
2000	Odisha	7395	4931	8846	10428	6762	12108	11844	7206	12686
2001	Odisha	6988	4660	8359	9854	6390	11442	11192	6809	11987
2002	Odisha	6578	4386	7868	9275	6015	10770	10535	6409	11284
2003	Odisha	6210	4141	7429	8757	5679	10169	9948	6052	10654
2004	Odisha	5845	3899	6994	8243	5346	9573	9364	5697	10029
2005	Odisha	5502	3667	6587	7759	5030	9015	8816	5363	9442
2006	Odisha	5172	3444	6197	7294	4726	8480	8289	5043	8878
2007	Odisha	4815	3204	5773	6791	4398	7899	7719	4696	8267
2008	Odisha	4484	2985	5378	6326	4097	7359	7191	4375	7702
2009	Odisha	4143	2759	4971	5845	3787	6802	6646	4043	7118
2010	Odisha	3825	2549	4592	5397	3498	6283	6139	3735	6576
2011	Odisha	3800	2431	4819	5258	3312	6388	5695	3465	6100
2012	Odisha	3488	2233	4427	4828	3042	5868	5232	3183	5603
2013	Odisha	3230	2068	4101	4471	2818	5435	4846	2948	5190
2014	Odisha	2950	1890	3746	4083	2574	4965	4427	2693	4742
2015	Odisha	2697	1728	3426	3734	2354	4541	4048	2463	4336
2000	Punjab	2454	1633	2975	3376	2190	3967	3603	2192	3859
2001	Punjab	2290	1524	2777	3151	2044	3703	3363	2046	3602
2002	Punjab	2132	1419	2585	2934	1903	3447	3130	1904	3353
2003	Punjab	1984	1319	2403	2729	1769	3205	2911	1771	3117
2004	Punjab	1841	1223	2227	2531	1640	2970	2698	1641	2889
2005	Punjab	1705	1139	2054	2343	1525	2741	2494	1517	2671
2006	Punjab	1575	1059	1888	2163	1414	2521	2299	1398	2462
2007	Punjab	1447	970	1726	1987	1296	2307	2108	1282	2257
2008	Punjab	1325	887	1579	1819	1185	2110	1928	1173	2065
2009	Punjab	1204	805	1434	1652	1076	1916	1750	1065	1875
2010	Punjab	1091	730	1300	1498	976	1738	1587	966	1700
2011	Punjab	993	664	1183	1363	888	1581	1444	878	1546
2012	Punjab	899	600	1069	1233	802	1428	1304	794	1397
2013	Punjab	828	552	982	1134	737	1312	1198	729	1283

Spn = *Streptococcus pneumoniae* ; pna = pneumonia; hiv = HIV-infected deaths only; lb = lower bound; ub = upper bound

Spn pneumonia mortality sensitivity results in India by state and year

Year	State	Spn pna deaths	Spn pna deaths_lb	Spn pna deaths_ub	Spn pna deaths	Spn pna deaths_lb	Spn pna deaths_ub	Spn pna deaths	Spn pna deaths_lb	Spn pna deaths_ub
2014	Punjab	764	513	961	1046	683	1264	1102	671	1180
2015	Punjab	721	482	903	987	643	1189	1036	631	1110
2000	Rajasthan	13329	8901	15907	18790	12198	21781	21326	12974	22841
2001	Rajasthan	12773	8530	15244	18006	11689	20872	20436	12433	21888
2002	Rajasthan	12203	8149	14563	17203	11167	19940	19524	11877	20910
2003	Rajasthan	11570	7724	13804	16309	10586	18902	18507	11259	19822
2004	Rajasthan	10916	7285	13018	15385	9983	17825	17453	10618	18693
2005	Rajasthan	10251	6855	12202	14445	9387	16713	16380	9965	17543
2006	Rajasthan	9582	6422	11384	13501	8788	15598	15303	9309	16389
2007	Rajasthan	8904	5983	10560	12544	8181	14475	14215	8648	15225
2008	Rajasthan	8261	5552	9798	11639	7591	13431	13189	8024	14126
2009	Rajasthan	8193	5314	10218	11315	7199	13575	12191	7417	13057
2010	Rajasthan	7548	4899	9420	10426	6637	12515	11239	6837	12037
2011	Rajasthan	6944	4509	8671	9593	6109	11520	10345	6294	11080
2012	Rajasthan	6311	4099	7882	8719	5553	10472	9404	5721	10072
2013	Rajasthan	5816	3778	7263	8035	5117	9650	8666	5272	9282
2014	Rajasthan	5361	3482	6695	7406	4717	8895	7988	4860	8556
2015	Rajasthan	5040	3274	6294	6963	4435	8363	7510	4569	8043
2000	Sikkim	40	26	50	56	36	67	60	37	64
2001	Sikkim	38	25	47	52	33	63	56	34	60
2002	Sikkim	35	23	44	48	31	58	52	32	56
2003	Sikkim	32	21	40	45	29	53	48	29	51
2004	Sikkim	29	19	37	41	26	49	44	27	47
2005	Sikkim	27	17	34	37	24	45	40	24	43
2006	Sikkim	25	16	31	34	21	41	37	22	39
2007	Sikkim	22	14	29	31	19	38	34	21	36
2008	Sikkim	21	13	27	29	18	36	32	19	34
2009	Sikkim	20	13	25	27	17	34	30	18	32
2010	Sikkim	19	12	24	26	17	32	29	17	31
2011	Sikkim	18	12	23	26	16	31	28	17	30
2012	Sikkim	18	12	22	25	16	30	27	16	28
2013	Sikkim	17	11	23	24	15	30	26	16	28

Spn = *Streptococcus pneumoniae* ; pna = pneumonia; hiv = HIV-infected deaths only; lb = lower bound; ub = upper bound

Spn pneumonia mortality sensitivity results in India by state and year

Year	State	Spn pna deaths	Spn pna deaths_lb	Spn pna deaths_ub	Spn pna deaths	Spn pna deaths_lb	Spn pna deaths_ub	Spn pna deaths	Spn pna deaths_lb	Spn pna deaths_ub
2014	Sikkim	17	11	23	23	15	30	25	15	27
2015	Sikkim	17	11	23	23	14	29	25	15	27
2000	Tamil Nadu	3610	2357	4482	4984	3186	5960	5365	3264	5746
2001	Tamil Nadu	3433	2241	4262	4739	3029	5667	5101	3103	5463
2002	Tamil Nadu	3248	2120	4032	4484	2866	5361	4826	2936	5169
2003	Tamil Nadu	3062	1997	3799	4226	2700	5051	4547	2766	4870
2004	Tamil Nadu	2872	1872	3560	3963	2531	4734	4262	2593	4564
2005	Tamil Nadu	2677	1754	3306	3693	2367	4399	3967	2414	4249
2006	Tamil Nadu	2484	1636	3056	3426	2205	4068	3677	2237	3938
2007	Tamil Nadu	2289	1517	2804	3155	2040	3736	3383	2058	3623
2008	Tamil Nadu	2109	1397	2583	2907	1879	3441	3116	1896	3337
2009	Tamil Nadu	1937	1276	2377	2670	1719	3166	2862	1741	3066
2010	Tamil Nadu	1778	1166	2188	2452	1573	2912	2629	1599	2816
2011	Tamil Nadu	1633	1037	2158	2252	1411	2823	2416	1470	2587
2012	Tamil Nadu	1485	938	1967	2048	1278	2573	2198	1337	2354
2013	Tamil Nadu	1368	864	1812	1887	1177	2370	2025	1232	2169
2014	Tamil Nadu	1260	796	1670	1738	1085	2184	1865	1135	1998
2015	Tamil Nadu	1201	758	1591	1656	1033	2080	1777	1081	1903
2000	Tripura	204	131	260	283	178	345	307	187	329
2001	Tripura	184	118	234	255	160	310	276	168	296
2002	Tripura	168	107	214	232	146	283	252	153	270
2003	Tripura	153	98	194	211	133	257	229	139	245
2004	Tripura	139	89	176	192	121	234	208	127	223
2005	Tripura	128	82	162	177	112	215	192	117	206
2006	Tripura	118	76	149	164	104	198	177	108	190
2007	Tripura	111	72	140	154	98	186	166	101	178
2008	Tripura	103	67	129	143	91	172	154	94	165
2009	Tripura	97	63	121	134	85	160	144	88	154
2010	Tripura	91	60	113	126	81	150	135	82	145
2011	Tripura	86	57	106	119	77	141	127	78	136
2012	Tripura	79	53	95	108	71	127	116	70	124
2013	Tripura	71	47	86	98	63	115	104	63	112

Spn = *Streptococcus pneumoniae* ; pna = pneumonia; hiv = HIV-infected deaths only; lb = lower bound; ub = upper bound

Spn pneumonia mortality sensitivity results in India by state and year

Year	State	Spn pna deaths	Spn pna deaths_lb	Spn pna deaths_ub	Spn pna deaths	Spn pna deaths_lb	Spn pna deaths_ub	Spn pna deaths	Spn pna deaths_lb	Spn pna deaths_ub
2014	Tripura	62	41	80	86	55	105	91	55	98
2015	Tripura	54	35	69	74	47	90	78	48	84
2000	Union Territory	137	90	171	189	121	226	202	123	217
2001	Union Territory	131	86	162	180	115	215	192	117	206
2002	Union Territory	124	81	154	171	109	204	183	111	196
2003	Union Territory	119	77	150	163	104	198	174	106	187
2004	Union Territory	114	74	143	156	99	189	167	101	179
2005	Union Territory	108	71	135	148	95	179	158	96	169
2006	Union Territory	103	68	128	142	92	169	150	91	161
2007	Union Territory	99	67	121	136	89	161	143	87	153
2008	Union Territory	96	64	117	131	85	154	137	83	147
2009	Union Territory	91	60	110	124	80	146	130	79	139
2010	Union Territory	88	58	106	120	77	141	125	76	133
2011	Union Territory	85	56	104	116	75	137	121	74	130
2012	Union Territory	81	53	100	110	71	131	115	70	123
2013	Union Territory	78	51	96	106	69	127	111	68	119
2014	Union Territory	73	48	90	100	64	119	104	63	112
2015	Union Territory	68	44	83	92	59	110	97	59	104
2000	Uttar Pradesh	47572	32041	56025	66940	43735	76857	75635	46013	81007
2001	Uttar Pradesh	45642	30741	53752	64224	41960	73739	72567	44146	77721
2002	Uttar Pradesh	43666	29411	51427	61445	40146	70550	69428	42237	74360
2003	Uttar Pradesh	41672	28072	49085	58641	38317	67337	66266	40314	70973
2004	Uttar Pradesh	39597	26681	46654	55725	36419	64001	62984	38317	67457
2005	Uttar Pradesh	37422	25185	44141	52671	34391	60543	59548	36227	63778
2006	Uttar Pradesh	35281	23716	41663	49663	32399	57133	56165	34168	60154
2007	Uttar Pradesh	33075	22207	39102	46563	30351	53610	52674	32045	56416
2008	Uttar Pradesh	30915	20766	36565	43529	28381	50131	49256	29965	52755
2009	Uttar Pradesh	28792	19349	34069	40544	26444	46710	45894	27920	49154
2010	Uttar Pradesh	26709	17960	31624	37618	24546	43357	42601	25916	45626
2011	Uttar Pradesh	26549	17244	33040	36654	23345	43909	39462	24007	42265
2012	Uttar Pradesh	24243	15759	30195	33478	21335	40128	36064	21940	38626
2013	Uttar Pradesh	22358	14541	27861	30879	19686	37026	33277	20244	35640

Spn = *Streptococcus pneumoniae* ; pna = pneumonia; hiv = HIV-infected deaths only; lb = lower bound; ub = upper bound

Spn pneumonia mortality sensitivity results in India by state and year

Year	State	Spn pna deaths	Spn pna deaths_lb	Spn pna deaths_ub	Spn pna deaths	Spn pna deaths_lb	Spn pna deaths_ub	Spn pna deaths	Spn pna deaths_lb	Spn pna deaths_ub
2014	Uttar Pradesh	20487	13329	25538	28298	18045	33939	30502	18556	32669
2015	Uttar Pradesh	18909	12302	23571	26119	16655	31325	28153	17127	30153
2000	Uttarakhand	1061	715	1251	1494	976	1716	1688	1027	1808
2001	Uttarakhand	1015	684	1197	1429	934	1643	1616	983	1731
2002	Uttarakhand	969	653	1143	1364	891	1568	1543	938	1652
2003	Uttarakhand	990	646	1227	1366	873	1631	1469	894	1573
2004	Uttarakhand	937	613	1163	1294	828	1547	1393	848	1492
2005	Uttarakhand	883	573	1103	1220	777	1465	1316	800	1409
2006	Uttarakhand	830	536	1043	1147	727	1384	1240	754	1328
2007	Uttarakhand	777	498	981	1075	678	1302	1162	707	1245
2008	Uttarakhand	726	467	919	1005	635	1218	1088	662	1165
2009	Uttarakhand	674	433	853	933	590	1131	1010	615	1082
2010	Uttarakhand	621	399	786	860	543	1042	931	566	997
2011	Uttarakhand	566	363	716	783	494	949	848	516	908
2012	Uttarakhand	502	323	635	694	439	842	752	458	805
2013	Uttarakhand	446	287	565	618	390	749	669	407	716
2014	Uttarakhand	393	253	497	544	344	659	589	358	631
2015	Uttarakhand	348	224	440	481	304	584	521	317	558
2000	West Bengal	8496	5570	10494	11722	7517	13963	12595	7662	13490
2001	West Bengal	7749	5080	9571	10690	6856	12735	11487	6988	12303
2002	West Bengal	6964	4565	8601	9608	6161	11445	10323	6280	11057
2003	West Bengal	6275	4113	7749	8657	5551	10311	9301	5658	9961
2004	West Bengal	5697	3734	7035	7860	5039	9360	8443	5137	9043
2005	West Bengal	5203	3413	6418	7176	4605	8541	7708	4689	8255
2006	West Bengal	4757	3124	5863	6561	4213	7804	7045	4286	7545
2007	West Bengal	4341	2854	5346	5987	3848	7116	6427	3910	6883
2008	West Bengal	3923	2578	4828	5409	3475	6427	5804	3531	6216
2009	West Bengal	3517	2314	4323	4849	3118	5756	5201	3164	5571
2010	West Bengal	3250	2141	3990	4480	2884	5313	4804	2922	5145
2011	West Bengal	3104	2047	3806	4278	2756	5069	4585	2789	4911
2012	West Bengal	2969	1961	3637	4092	2639	4844	4384	2667	4696
2013	West Bengal	2940	1942	3600	4051	2613	4796	4340	2640	4649

Spn = *Streptococcus pneumoniae* ; pna = pneumonia; hiv = HIV-infected deaths only; lb = lower bound; ub = upper bound

Spn pneumonia mortality sensitivity results in India by state and year

Year	State	Spn pna deaths	Spn pna deaths_lb	Spn pna deaths_ub	Spn pna deaths	Spn pna deaths_lb	Spn pna deaths_ub	Spn pna deaths	Spn pna deaths_lb	Spn pna deaths_ub
2014	West Bengal	2932	1936	3591	4041	2606	4783	4329	2633	4636
2015	West Bengal	2965	1958	3631	4086	2635	4836	4377	2663	4687

Spn = *Streptococcus pneumoniae* ; pna = pneumonia; hiv = HIV-infected deaths only; lb = lower bound; ub = upper bound
